# Fully synthetic Mincle-dependent self-adjuvanting cancer vaccines elicit robust humoral and T cell-dependent immune responses and protect mice from tumor development†

**DOI:** 10.1039/d1sc05736g

**Published:** 2021-12-01

**Authors:** Xiang Luo, Qinghai Lian, Wenwei Li, Liqing Chen, Renyu Zhang, Deying Yang, Lingqiang Gao, Xiaoxiao Qi, Zhongqiu Liu, Guochao Liao

**Affiliations:** Joint Laboratory for Translational Cancer Research of Chinese Medicine of the Ministry of Education of the People's Republic of China, International Institute for Translational Chinese Medicine, Guangzhou University of Chinese Medicine Guangzhou 510006 China liuzq@gzucm.edu.cn liao@gzucm.edu.cn

## Abstract

A new strategy based on a macrophage-inducible C-type lectin (Mincle) agonist was established to construct synthetic cancer vaccines. Using sialyl-Tn (STn) as a model antigen, four conjugates with the Mincle agonist as a built-in adjuvant were designed and synthesized through a facile and efficient method. All conjugates could induce BMDMs to produce inflammatory cytokines in a Mincle-dependent manner and were found to elicit robust humoral and T cell-dependent immune responses alone in mice. The corresponding antibodies could recognize, bind and exhibit complement-dependent cytotoxicity to STn-positive cancer cells, leading to tumor cell lysis. Moreover, all conjugates could effectively inhibit tumor growth and prolong the mice survival time *in vivo*, with therapeutic effects better than STn-CRM197/Al. Notably, compared to conventional glycoprotein conjugate vaccines, these fully synthetic conjugate vaccines do not cause “epitope suppression.” Mincle ligands thus hold great potential as a platform for the development of new vaccine carriers with self-adjuvanting properties for cancer treatment. Preliminary structure–activity relationship analysis shows that a vaccine containing one STn antigen carried by vizantin exhibits the best efficacy, providing support for further optimization and additional investigation into Mincle agonists as the carrier of self-adjuvanting cancer vaccines.

## Abstract 2

A new strategy based on a Macrophage-inducible C-type lectin (Mincle) agonist was established to construct synthetic cancer vaccines.

## Graphical abstract

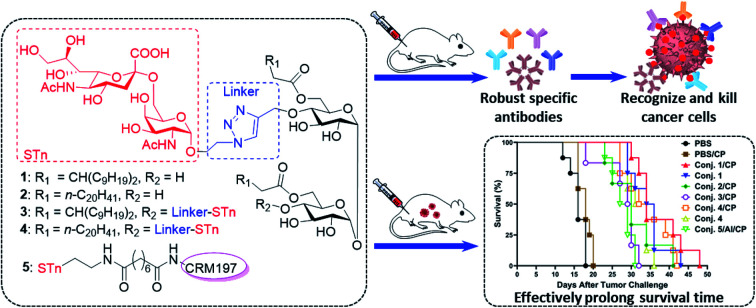

## Introduction

Vaccination has become an effective strategy for cancer treatment due to its low side effects and high specificity.^1^ Tumor-associated carbohydrate antigens (TACAs), which are overexpressed on the surface of various cancer cells and highly associated with tumor metastasis and signal transduction, have been regarded as particularly promising targets for therapeutic cancer vaccines.^2,3^ However, TACAs are weakly immunogenic and T cell-independent, and thus cannot elicit robust enough immune responses for effective cancer therapy by themselves. To induce T cell-mediated and long-lasting antibody responses, which are critical for cancer immunotherapy, TACAs are conventionally used to covalently conjugate with immunogenic carrier proteins such as KLH, CRM197 and TT.^4–7^ This strategy can not only increase the immunogenicity of carbohydrates but can also convert them from being T cell-independent to T cell-dependent, and thus has been widely used in carbohydrate-based antitumor vaccines.^2,8,9^ Over the years, several carbohydrate–protein anticancer vaccines including monovalent and polyvalent vaccines have been developed and applied in preclinical/clinical trials, such as GM2-KLH/QS-21 and Globo H-CRM197/C34.^10–14^

Although TACA–protein vaccination strategies have yielded encouraging results, some limitations prevent their further development. First, the conjugation sites and equivalents of TACAs to carrier proteins are random and uncontrollable, which makes it very difficult to maintain consistency in physical, chemical, and immunological properties across sample batches, affecting their efficacy.^15^ Second, a carrier protein may induce high antibody responses to itself, leading to “epitope suppression” and impairing the specific response to TACAs.^16–18^ Third, cold chain transportation is needed to prevent protein carrier degradation or aggregation. In addition, an external adjuvant is usually co-administrated to provoke adaptive immune responses, which may lead to serious side effects.^19^ To address such defects, fully synthetic self-adjuvanting carbohydrate-based vaccines with small molecule carriers were designed and synthesized. These vaccines have well-defined molecular chemical structures that can be fully characterized with standard methods, contributing to quality control and structure–activity relationship (SAR) analysis. Meanwhile, carrier proteins and additional adjuvants are not needed. This could avoid protein-induced “epitope suppression” and adjuvant-induced side effects. Moreover, self-adjuvanting vaccine constructs allow antigen presenting cells (APCs) to simultaneously take up antigens and adjuvants to promote an antigen-specific immune response, eliciting more specific immune responses.

A carrier molecule (built-in adjuvant) that efficiently stimulates the immune system to recognize TACAs and induces increased levels of antibodies is essential for the development of fully synthetic carbohydrate-based vaccines. Generally, the ligand of Toll-like receptors (such as Pam3Cys,^20–22^ UPam,^23,24^ and monophosphoryl lipid A^25–27^), zwitterionic polysaccharides,^28–30^ and an invariant natural killer T (iNKT) cell agonist (such as KRN7000)^31–34^ are usually used as the delivery carriers. In the past decade, many fully synthetic carbohydrate-based vaccines, which could elicit high TACA-specific IgG antibodies and exhibit effective complement-dependent cytotoxicity, have been explored.^35–37^ However, developing a qualified therapeutic TACA vaccine that could protect patients from tumor development, which is extremely challenging, is highly desired.

**Fig. 1:**
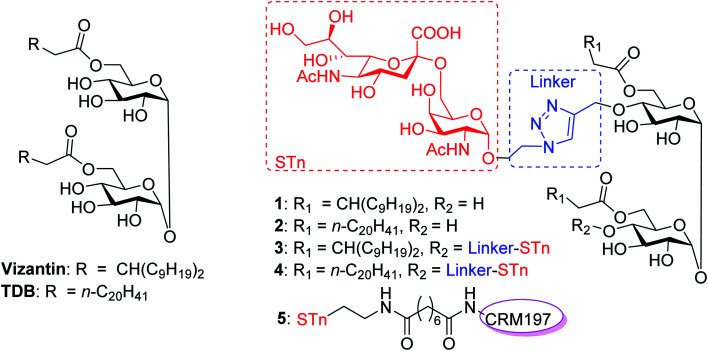
Structures of vizantin, TDB and designed STn conjugates.

The macrophage-inducible C-type lectin (Mincle, also called Clec4e or Clecsf9), which belongs to C-type lectin domain family 4, is a transmembrane C-type lectin receptor (CLR) expressed on activated macrophages and dendritic cells (DCs).^38^ The activation of Mincle can initiate the FcRγ-Syk-Card9-Bcl10-Malt1 signaling cascade, leading to the activation of nuclear factor kappa B (NF-κB) cells and production of pro-inflammatory cytokines, chemokines and growth factors, thereby eliciting responses through T helper 1 (Th1)/Th17 immune cells.^39–41^ In this context, the ligand of Mincle is regarded as a promising adjuvant to be co-administered with vaccine antigens. Over the past decade, many glycolipids have been isolated or synthesized as Mincle activators.^42–47^ For example, 6,6′-bis-*O*-(3-nonyldodecanoyl)-*α*,*α*′-trehalose (vizantin,^42^Fig. 1) and trehalose-6,6′-dibehenate (TDB)^48^ have proven to be useful and nontoxic Mincle ligands that induce strong Th17 and Th1 immune responses, and TDB has entered clinical studies as a vaccine adjuvant for both tuberculosis (TB) and HIV when formulated in the CAF01 liposome system.^48,49^ Based on these encouraging successes of Mincle ligands and our previous study on fully synthetic cancer vaccines,^50–53^ we envisioned that a Mincle agonist could be used as both a carrier molecule and a built-in adjuvant to create new and effective synthetic self-adjuvanting carbohydrate-based vaccines.

The Sialyl Thomsen-nouveau (STn, Neu5Acα2-6GalNAcα-*O*-Ser/Thr) antigen, an *O*-linked mucin-related TACA, is overexpressed in human carcinomas including breast, colorectal, pancreas, lung, prostate, ovarian and gastric cancers. The overexpression of the STn antigen is associated with increased tumor growth, tissue invasion, and metastasis of cancer.^54^ In addition, STn is usually correlated with poor prognosis in cancer patients because high levels of STn expression in cancer show rapid aggressiveness.^55^ Indeed, STn has been considered one of the most promising targets for therapeutic cancer vaccine development. In the past few decades, many STn based vaccines have been designed, such as STn-KLH/QS21,^4^ STn-KRN7000,^31^ and STn-CRM197/FA.^56^ Among these, the conjugation vaccine of STn-KLH (Theratope^®^) was approved for clinical trials to treat colorectal and breast cancer. Unfortunately, it failed to display decreased disease progression or increased overall survival in phase III trials.^57^ Although this therapeutic cancer vaccine failed, it provided valuable information and insight into the development of new anti-STn vaccines. (1) The STn antigen had an acceptable safety profile for the development of antitumor vaccines; and (2) the utilization of an effective carrier to activate T cell-dependent immunity for the sufficient enhancement of STn immunogenicity may be possible to make the vaccine effective.

In this context, we were the first to propose a new self-adjuvanting carbohydrate-based vaccine design using the Mincle agonist as a carrier molecule and built-in adjuvant. For this purpose, STn, a selective TACA for a cancer vaccine, was coupled with promising Mincle agonists vizantin and TDB to produce designed conjugates STn-vizantin (1) and STn-TDB (2), respectively (Fig. 1). A monovalent cluster of cancer antigens may be more efficient than a single one;^58,59^ thus, conjugates containing a cluster of two STn antigens were also designed (3 and 4). In addition, the linker between the TACA and carrier is also critical to self-adjuvanting cancer vaccines.^60^ 1,2,3-Triazole, which has been safely utilized for the construction of fully synthetic conjugate vaccines, was selected as the linker.^26^ The evaluation of immune effects and the anti-tumor efficacy of resultant conjugates 1–4 were examined in mice, and their results were compared with a semisynthetic glycoprotein of STn-CRM197 (5) with an external adjuvant.^56^

## Results and discussion

### Preparation of glycoconjugates 1–4

**Scheme 1:**
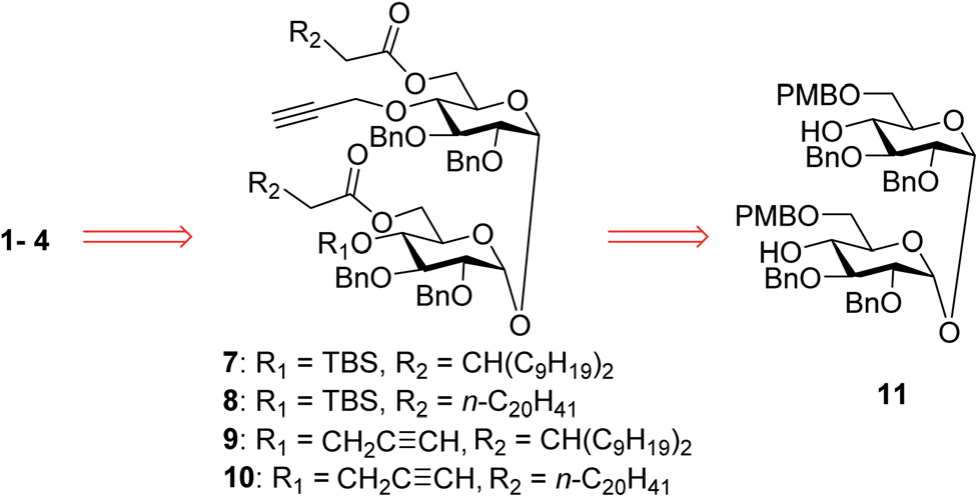
Retrosynthetic analysis of the target conjugates 1–4.

In our synthetic design for target molecules 1–4 (Scheme 1), the key step was to selectively construct 6,6′-diester trehalose derivatives containing one (7 and 8) or two (9 and 10) propargyl groups that could further couple with an STn antigen derivative equipped azide group by a click reaction, which has the advantages of superior stability, high yield and mild reaction conditions. The global deprotection of derivatives 7–10 also affords vizantin or TDB derivatives, which could be explored as independent vaccine adjuvants. In return, 7–10 could be obtained from the common intermediate 11, which could be obtained from readily available *α*,*α*′-d-trehalose. The choice of protecting groups is important during the preparation of 7–10. In our design, the 6,6′-*O*-positions of 11 were differently protected from 2,3,2′,3′-*O*-positions to enable regioselective deprotection and follow-up acylation. In addition, to selectively generate conjugates with a single antigen, a *tert*-butyldimethylsilyl (TBS) group, which can easily be removed later, was used to protect the 4′-*O*-position of intermediate 11.

**Scheme 2:**
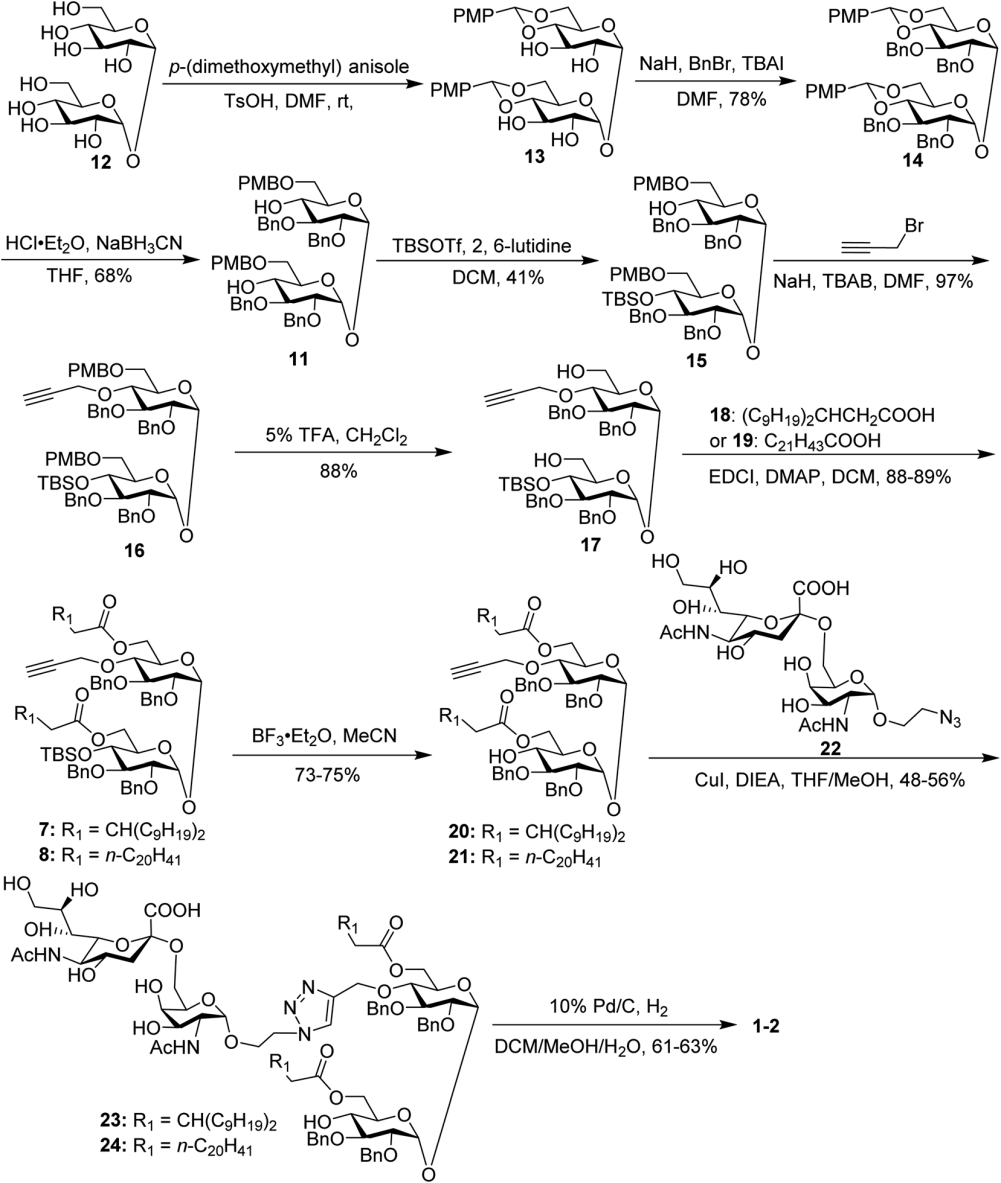
Synthesis of conjugates 1–2.

The synthesis of the target glycoconjugates 1–4 started from commercially available *α*,*α*′-d-trehalose (12), as shown in Scheme 2. Initially, *p*-methylphenyl (PMP) protected intermediate 13 was obtained according to a reported procedure.^61^ Substrate 13 reacted with benzyl bromide (BnBr) in the presence of sodium hydride (NaH) and tetrabutylammonium iodide (TBAI) using anhydrous DMF as solvent to give tetrabenzyl trehalose (14). Subsequently, the benzylidene ring in 11 was regioselectively opened with NaBH_3_CN and HCl·Et_2_O to expose hydroxyl groups at the 4,4′-position. For conjugates containing a single STn antigen, compound 11 was treated with *tert*-butyldimethylsilyl trifluoromethanesulfonate (TBSOTf) to selectively deliver a single hydroxyl-exposed intermediate (15). Then, compound 15 was reacted with 2-propynyl bromide in the presence of NaH and tetrabutylammonium bromide (TBAB) to insert a propargyl group in preparation for the follow-up click reaction. The 6,6′-*O*-PMB groups in 16 were removed under 5% trifluoroacetic acid (TFA) conditions to give the hydroxyl group exposed intermediate 17. Lipid acids 18 and 19 were synthesized through the described procedures using commercially available raw materials (see the ESI†).^42^ Then, lipid installation was performed using 1-(3-dimethylaminopropyl)-3-ethylcarbodiimide hydrochloride (EDCI) and 4-dimethylaminopyridine (DMAP) as promoters to generate the key intermediates 7 and 8, respectively. The TBS groups at the 4′-*O*-position of 7 and 8 were deprotected by BF_3_·Et_2_O to produce trehalose diester derivatives 20 and 21 for conjugation with the antigen.

The conjugates of trehalose diester derivatives and the STn antigen were then assembled. An azide group-equipped STn derivative, 22, was prepared from the start material *N*-acetyl-d-galactosamine using a similar approach to that in the literature (see the ESI†).^62,63^ Then, intermediates 20 and 21 were coupled with 22 through a click reaction catalyzed by cuprous iodide (CuI) in the presence of *N*,*N*-diisopropylethylamine (DIEA) to afford the desired products 23 and 24, respectively, which were carefully characterized by ^1^H, ^13^C, 2D NMR and HRMS. Finally, all benzyl groups in products 23 and 24 were removed through hydrogenolysis under a H_2_ atmosphere using Pd as the catalyst, leading to target conjugates 1 and 2.

**Scheme 3:**
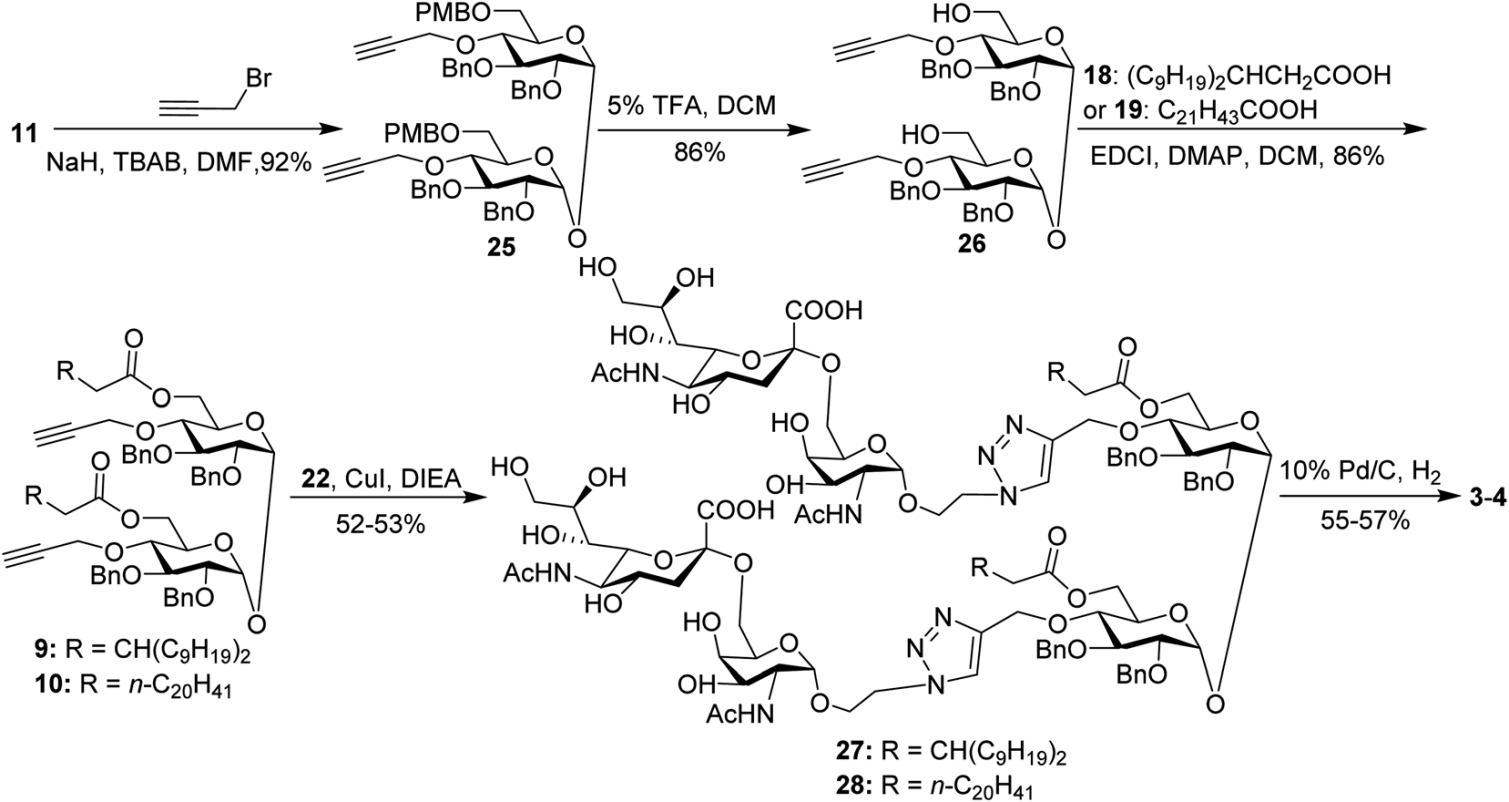
Synthesis of the target conjugates 3–4.

For conjugates containing two STn antigens, intermediate 11 was directly reacted with 5 equivalents of 2-propynyl bromide in the presence of NaH and TBAB to give intermediate 25 bearing two propargyl groups (Scheme 3). After removing PMB groups at the 6,6′-*O*-position of compound 25, acylation was performed to give the key intermediates 9 and 10. Finally, the desired products 3 and 4 were obtained through a click reaction, followed by hydrogenolysis.

### Preparation of STn-CRM197 and STn-HSA

**Scheme 4:**
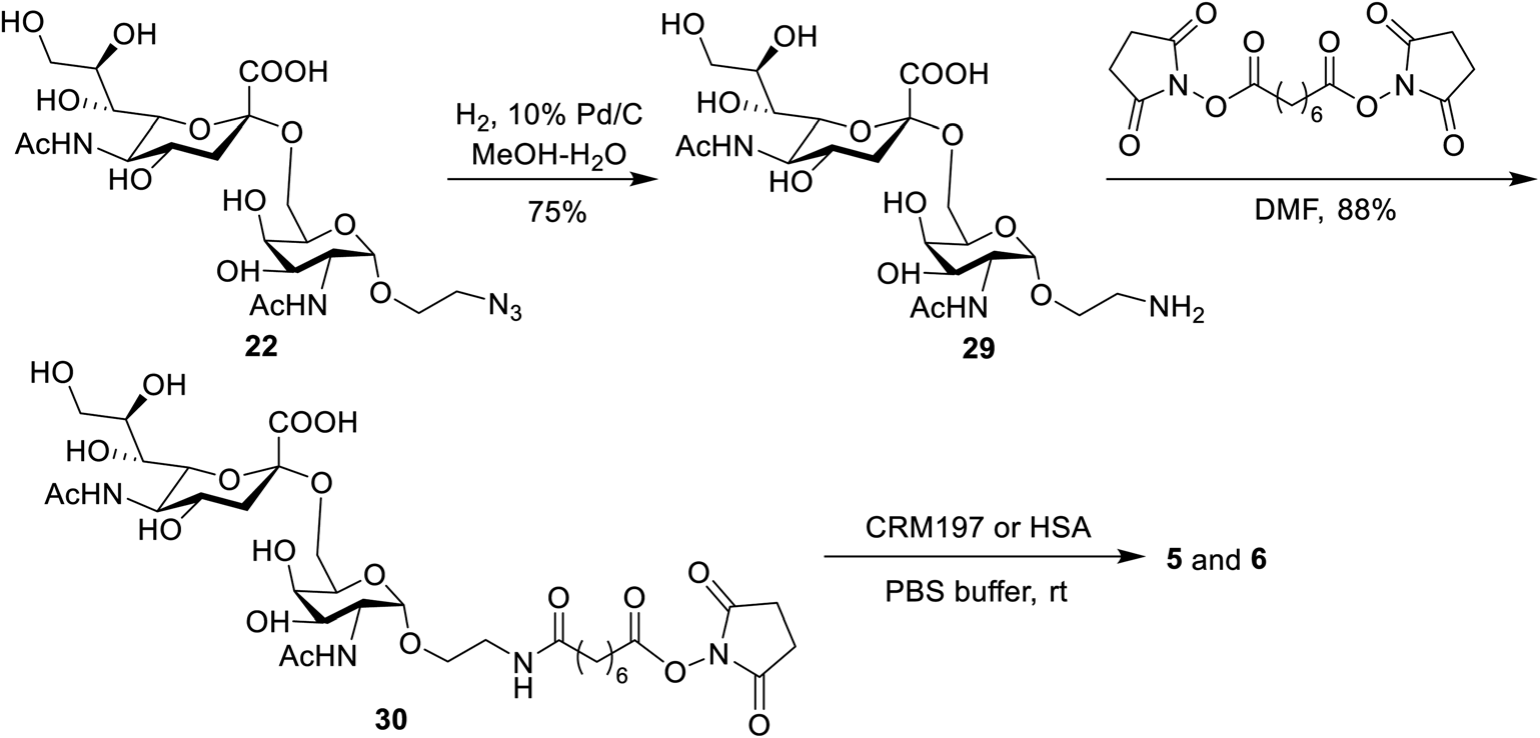
Synthesis of STn-CRM197 and STn-HAS.

The conjugates of STn-CRM197 (5) and STn-HSA (6), which were used as the positive control and coating antigen for enzyme-linked immunosorbent assays (ELISA) of STn-specific antibodies, respectively, were also prepared (Scheme 4). Previous work reported that the triazolyl moiety used as the linker in the protein–carbohydrate vaccine may have a major impact on immunological activity.^60^ Thus, a bifunctional suberic acyl was selected as the linker; this could not only enabled powerful coupling reactions but also avoided a negative impact on the immunological properties of the resulting glycoconjugates.^64^ In this transformation, the azide group in STn derivative 22 was first reduced to the free amine (29), which was subsequently coupled with disuccinimidyl suberic acid to afford the activated ester (30) in an 88% yield. The treatment of intermediate 30 with CRM197 or HSA in 0.1 M PBS buffer (pH = 7.8), followed by purification with dialysis and lyophilization, afforded the desired products 5 and 6, respectively. The two conjugates were identified by SDS-PAGE. An obvious increase in the molecular weight of the glycoconjugate compared to the protein alone proved successful conjugation between STn and the protein. The epitope ratio of conjugate 5 was analyzed using the Svennerholm method.^65,66^ This revealed that conjugate 5 contained 9.6% STn, indicating that the coupling reactions were effective and the antigen loading levels were in the desired range (5–20%) for glycoconjugate vaccines (see the ESI†). The carbohydrate loading of conjugate 6, which was analyzed by MALDI-TOF MS, demonstrated that it was suitable for use as a coating antigen for ELISA.

### Binding affinity of conjugates 1–4 to hMincle

**Fig. 2:**
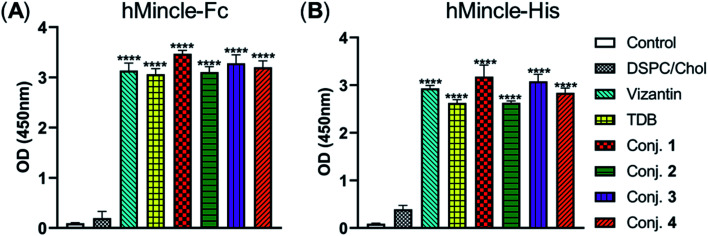
The binding affinity of conjugates 1–4 to hMincle-Fc and hMincle-His proteins. Data are representative of three independent experiments performed in triplicate (mean ± SD); ****P < 0.0001.

The binding affinity of 1–4 with soluble Mincle-Fc and Mincle-His proteins, respectively, was firstly determined to test whether the target conjugates can recognize and bind to human Mincle. The Mincle ligands TDB and vizantin were synthesized from trehalose according to a literature reported protocol with 7 steps^42^ (see the ESI†) and used as the positive control. Plates coated with vizantin, TDB or conjugates 1–4 (0.1 nmol per well) were incubated with hMincle-Fc or hMincle-His, and the ligand bound protein was detected *via* ELISA. As the conjugates will be immunized in a form of liposomes containing 1,2-distearoyl-*sn*-glycero-3-phosphocholine (DSPC) and cholesterol (Chol), the binding ability of the mixture of DSPC and cholesterol was also tested. As shown in Fig. 2, the mixture of DSPC and cholesterol alone did not show significant binding to hMincle-Fc or hMincle-His. All target conjugates could recognize and bind to both hMincle-Fc and hMincle-His proteins in comparison with the control, and their binding affinity was comparable to TDB and vizantin. Meanwhile, there was no statistically significant difference of 1–4 in the ability to bind to hMincle-Fc or hMincle-His protein. These results reveal that the introduction of the STn antigen into the 4- or/and 4′-*O*-position did not significantly affect the binding ability of vizantin or TDB to hMincle protein.

### Abilities of conjugates 1–4 to induce the production of inflammatory cytokines

**Fig. 3:**
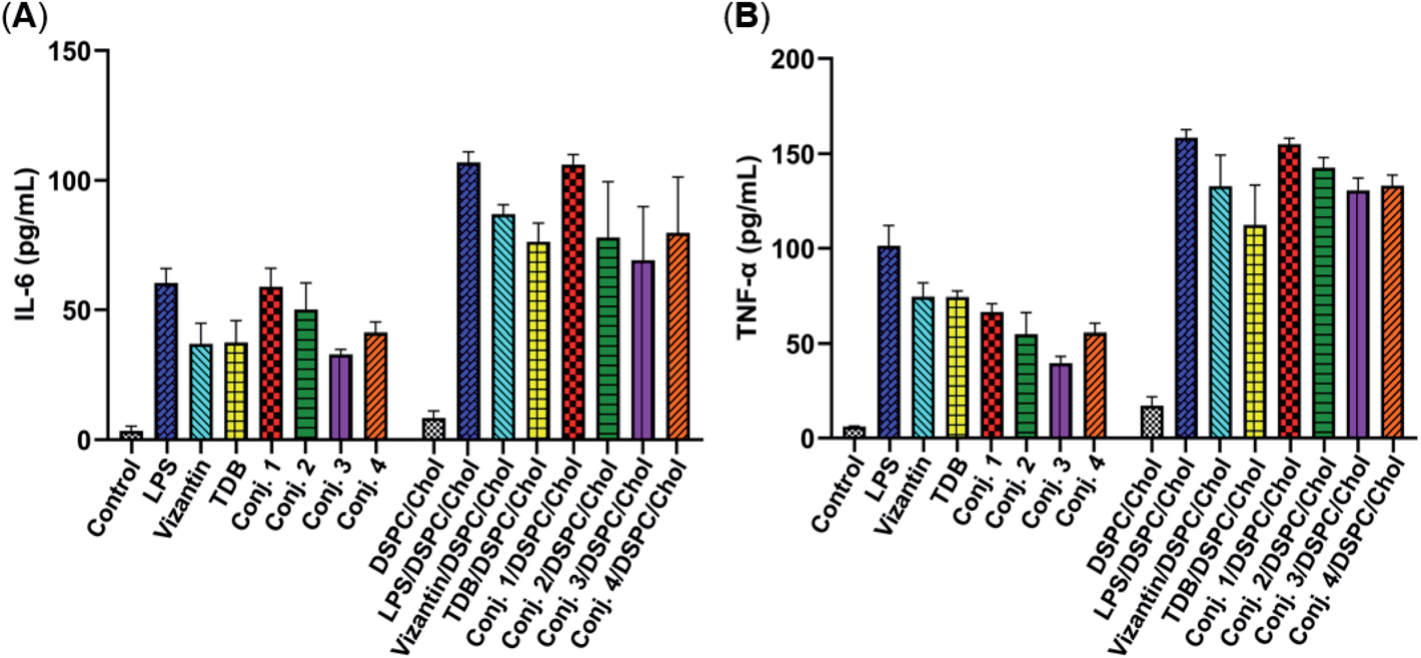
The capabilities of conjugates 1–4 to induce BMDMs to produce inflammatory cytokines IL-6 and TNF-α. Harvested BMDMs (1 × 10^4^ per well) were stimulated using vizantin, TDB or conjugates 1–4 coated plates (1 nmol per well) or LPS (100 ng per well). Cytokine production was measured by ELISA from the supernatant collected after 24 h. Data are representative of three independent experiments performed in triplicate (mean ± SD).

Then, the abilities of conjugates 1–4 to induce bone marrow derived macrophages (BMDMs) to produce inflammatory cytokines IL-6 and TNF-α were examined. This experiment has been widely used for the functional evaluation of Mincle ligands.^43,67^ As depicted in Fig. 3, the stimulation of BMDMs with conjugates 1–4 led to significant production of IL-6 and TNF-α. The levels of IL-6 induced by vizantin, TDB and conjugates 1–4 follow the order 1 > 2 > 4 ∼ vizantin ∼ TDB ∼ 3. All conjugates resulted in a lower production of TNF-α than vizantin and TDB, and the efficiency follows the order vizantin ∼ TDB > 1 > 2 > 4 ∼ 3. In addition, the efficiency of the above compounds that were co-administrated with DSPC and cholesterol was also measured. The mixture of DSPC and cholesterol alone did not significantly promote the production of IL-6 and TNF-α. When co-administered with DSPC and cholesterol, the production of IL-6 and TNF-α induced by all compounds significantly increased. These results revealed that administration in the form of liposomes could improve the immunogenicity of vizantin, TDB and conjugates 1–4. Collectively, all these studies suggest that conjugates 1–4 could induce BMDMs to produce inflammatory cytokines in a Mincle-dependent manner, and their efficiencies are comparable to those of Mincle ligands TDB and vizantin.

### Immunological evaluation of conjugates 1–5

The immunological evaluation of target conjugates 1–5 was performed with 6–8 week-old female BALB/c mice. The target conjugates were administrated in the form of liposomes prepared by sonication of a mixture of the conjugates 1–4, DSPC and cholesterol (molar ratios: DSPC/cholesterol/1–4 = 65 : 50 : 10) to improve their solubility and immunogenicity. As glycoprotein conjugate 5 would be more effective in the presence of an external adjuvant, it was administrated as an emulsion with a clinically used alum adjuvant (Al). In this experiment, conjugate 5 was first dissolved in PBS buffer (pH = 9.6) and then thoroughly mixed with alum before use.

For mouse immunization, conjugates 1–4 (0.1 mL liposome containing 10 μg of STn) were each administrated to a group of six mice through subcutaneous (s.c.) injection. A dose of conjugate 5 (0.1 mL emulsion containing 2 μg of STn), which had proved to elicit strong immune responses with an external adjuvant,^56^ was administrated with Al *via* the same protocol. Afterward, mice received booster doses on days 14, 21 and 28 by s.c. injection of the same conjugate and using the same immunization protocol as on day 1. Each mouse was bled from the eye socket on day 0 before the initial immunization (blank controls) and on days 21, 27 and 38 after the first injection. The blood samples were treated to prepare antisera with reference to the standard protocol for the analysis of STn-specific antibodies by ELISA with STn-HSA conjugate 6 as the capture reagent. The total antibodies (kappa, IgG and IgM) and IgG antibody isotypes including IgG1, IgG2a, IgG2b and IgG3 titers were determined.

**Fig. 4:**
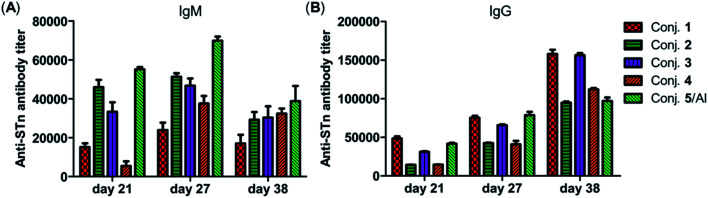
The IgM antibody (A) and IgG antibody (B) titers of pooled days 21, 27, and 38 sera derived from mice immunized with conjugate 1–5.

The total IgM and IgG antibody titers of the pooled sera on days 21, 27, and 38 derived from each group of mice immunized with conjugates 1–5 are shown in Fig. 4. It is evident that all conjugates elicited highly STn-specific Ig M titers, considered as the default antibody produced by B cells in response to a foreign antigen. High IgG antibody titers of the antisera on day 21 were also observed, and increased further after booster immunizations, suggesting a reinforcement of immune response against conjugates 1–5. After four immunizations, all IgM titers decreased, and IgG titers increased correspondingly, revealing that efficient conversion of IgM to IgG had occurred and that long-term secondary immune responses had been triggered. The different levels of the IgG antibody titer on day 38 preliminarily suggest that the immunogenicity of the tested conjugates follows the order 1 ∼ 3 > 4 > 2 ∼ 5/Al. These results reveal that vizantin and TDB can be used as novel carriers for the development of cancer vaccines and that vizantin may be a more effective carrier than TDB and CRM197.

**Fig. 5:**
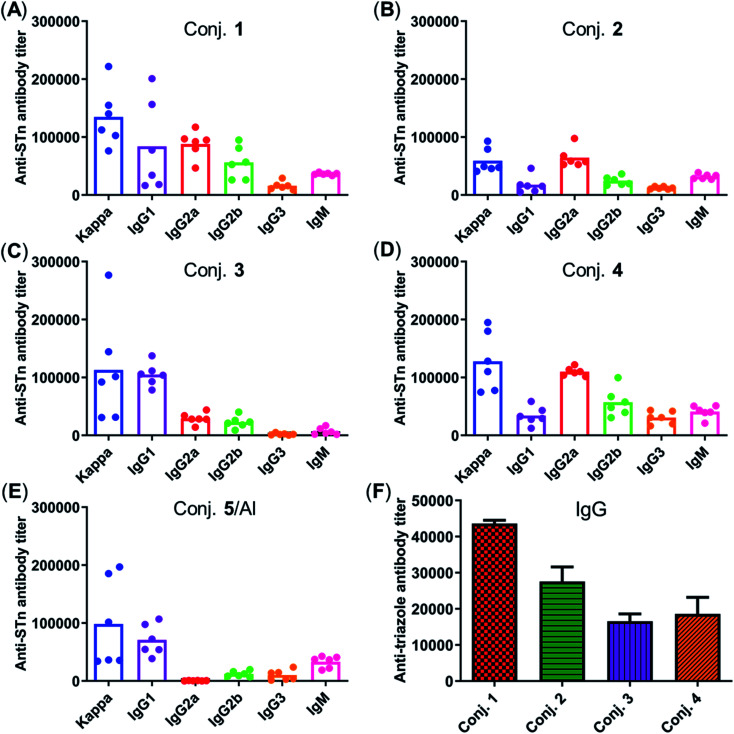
(A–E) The titers of total antibody and antibody isotypes in individual antiserum collected from mice immunized with conjugates 1–5 on day 38. Each dot represents the result of a mouse, and the bar represents the average titer for each group. (F) Titers of IgG antibody reactive to triazole-HSA in the pooled day 38 sera obtained with conjugates 1–4.

Fig. 5 depicts the ELISA results for the overall total (kappa) and various subclasses of antibody titers in day 38 antisera of each mouse immunized with conjugates 1–5 and for the group average. The high titers of kappa and IgG indicate that all conjugates provoked a strong STn-specific and T-cell dependent immune response desirable for cancer immunotherapy. The assessment of antibody isotypes revealed that mice immunized with conjugates 1–4 exhibited mainly IgG1, IgG2a and IgG2b antibodies, suggesting that conjugates 1–4 could stimulate both Th1 and Th2 immune responses. The average levels of kappa, IgG1 and IgG2a of STn-vizantin (1) were significantly higher than those of STn-TDB (2) and STn-CRM197/Al, indicating that vizantin as a built-in adjuvant can more effectively improve the immunogenicity of STn than TDB and CRM197. This result also preliminarily suggests that a trehalose derivative containing two shorter lipid chains may exhibit more effective immunostimulatory activities as a carrier. The ELISA result of (STn)_2_-vizantin (3) was similar to that of STn-vizantin (1), revealing that a vizantin-carried conjugate containing a cluster of two STn antigens was not more efficient than a single STn antigen. Interestingly, for TDB carried conjugates, (STn)_2_–TDB did elicit stronger immune responses than those with STn–TDB (2).

The immune response effects of the linker between STn and the carrier in these glycoconjugates were determined. Triazole-HSA (S17, see the ESI†), which was synthesized using a similar synthesis procedure to STn–HSA, was used as the capture reagent to test the reactivity of day 38 antisera induced by conjugates 1–4. The anti-triazole IgG antibody titers produced by antisera 1–4 were 43 612, 27 551, 16 546, and 18 608, respectively (Fig. 5F), and much lower than those of the corresponding anti-STn antibody. These results demonstrate that the triazolyl linker does not have a substantial influence on the immunological properties of target conjugates.

**Fig. 6:**
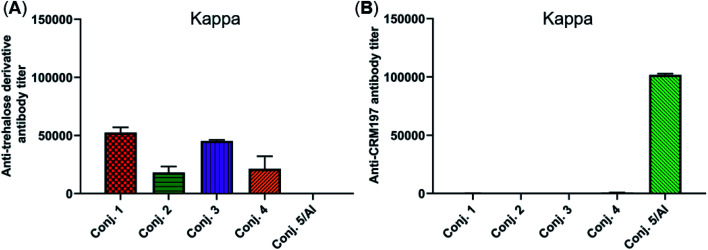
Titers of IgG antibody reactive to the trehalose derivative (A) or carrier protein (B) in the pooled day 38 sera obtained with conjugates 1–5. For ELISA, after the plates were coated with the corresponding substrate, they were treated with the antisera, and the antibodies bound to the plates were analyzed.

The reactivity of the day 38 antisera induced by the vaccine carrier of conjugates 1–5 was also investigated by means of ELISA. All antisera induced by conjugates 1–4 had a degree of reactions with the trehalose derivative (S5, see the ESI†), with total antibody titers of 52 502, 18 130, 45 260, and 21 367, respectively (Fig. 6A). They are much lower than those of the corresponding anti-STn antibody (118 295, 53 462, 116 510, and 51 895). In contrast, antisera induced by conjugate 5 exhibited strong reactivity to the CRM197 protein, and the CRM197-specific antibody titer was higher than that of the corresponding STn (101 725 *vs.* 82 747). These results demonstrate that CRM197 may inhibit the production of antibodies against the STn epitope, and conjugate 1–4 can avoid the influence of the immunogenicity of the carrier molecules as much as possible.

**Fig. 7:**
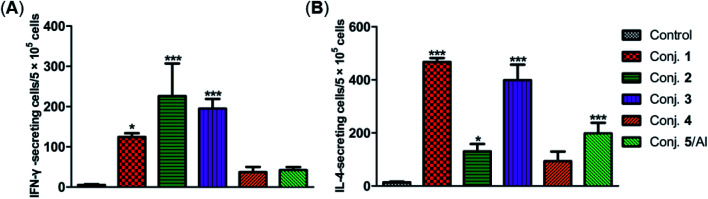
Number of IFN-γ (A) and IL-4 (B) secreted cells in the spleen of mice harvested on day 38 and stimulated *in vitro* with conjugates 1–5. Data are presented as mean ± SD; *p < 0.05; **p < 0.01.

The secretion of gamma interferon (IFN-γ) and interleukin 4 (IL-4) provoked by conjugates 1–5 was evaluated by ELISpot assay. Mice immunized with conjugates 1–5 developed distinctly higher numbers of IFN-γ and IL-4 spots as to the control, as shown in Fig. 7. Conjugate 2 elicited the highest expression level of IFN-γ, and conjugate 1 induced the highest level of IL-4. It is known that IFN-γ and IL-4 are secreted by Th1- and Th2-type cells, respectively. The increased IFN-γ expression thus indicates the activation of Th1 cells that can activate macrophages and mediate an IgG antibody switch. The increased expression of IL-4 suggested the activation of Th2 cells, which contribute to enhanced B cell immune responses and antibody conversion to IgG1. These results reveal that all conjugates can induce Th1 and Th2 immune responses, which is consistent with findings from ELISA. The IFN-γ/IL-4 ratios of conjugates 1–5 in our experiments were 0.265, 1.725, 0.489, 0.40, and 0.21, respectively, indicating that conjugates 1, 3, 4 and 5/Al caused a mainly humoral immune response, while conjugate 2 elicited a predominately cellular immune response.

### Capabilities of antiserum binding to cancer cells

The ability of antisera induced by conjugates 1–5 to recognize and bind to target cancer cells was subsequently detected by fluorescence-activated cell sorting (FACS) technology. Human breast cancer cell line MCF-7 and mouse colon cancer cell line CT-26, both of which are known to overexpress the STn antigen on their surface,^30,68^ were employed in these studies. STn negative cancer cell line B16-F10 (ref. 12) was used as a control. In these experiments, cancer cell lines were individually treated with either normal mouse serum (the negative control) or antisera obtained from mice vaccinated with conjugates 1–5. Then, tumor cells were cultured with a fluorescein isothiocyanate (FITC)-labeled goat anti-mouse IgG antibody, followed by FACS analysis.

**Fig. 8:**
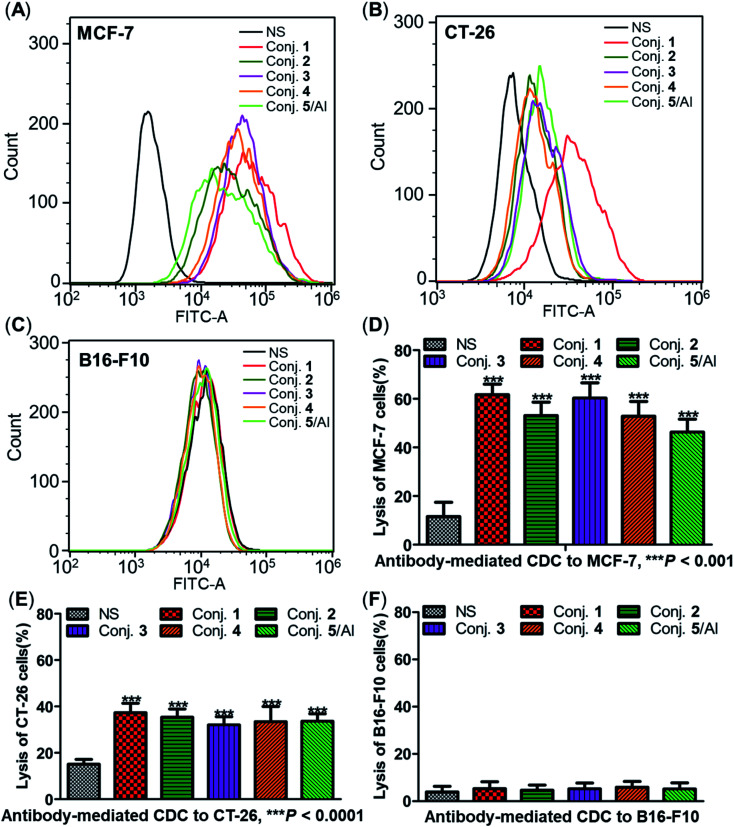
FACS analysis of IgG binding against STn positive or negative cancer cells (A–C) and lysis of cancer cells through antibody-mediated complement-dependent cytotoxicity (D–F). FACS assay results of the binding between MCF-7 (A), CT-26 (B) or B16-F10 (C) cancer cells and normal mouse serum (black), pooled mouse antisera induced by conjugate 1 (red), 2 (dark green), 3 (purple), 4 (orange) and 5 (light green) on day 38. Results of antibody-mediated CDC to MCF-7 (D), CT-26 (E) and B16-F10 (F) cells presented as cell lysis rates. ***Significant difference compared to normal mouse serum, *P* < 0.001.

Significantly positive fluorescent peak shifts were observed in MCF-7 and CT-26 cancer cells treated with antisera as compared to cells incubated with normal serum (Fig. 8A and B). In contrast, the fluorescent profiles of B16-F10 cancer cells treated with normal serum and the corresponding antisera did not differ noticeably (Fig. 8C). These FACS results indicate that all antisera induced by conjugates 1–5 could specifically recognize and bind to STn positive cancer cells, while the effect on STn negative cells was negligible. Moreover, the median fluorescence intensity (MFI) of MCF-7 and CT-26 cells treated with anti-1 serum was higher than that of the others (MCF-7: 33 071 *vs.* 21 436, 26 351, 19 808 and 11 911 for conjugate 1*vs.* conjugates 2–5; CT-26: 42 705 *vs.* 15 464, 18 643, 14 873 and 18 336), indicating stronger binding affinity of antibodies in anti-1 serum. These results matched the ELISA results, and provide further evidence supporting the conclusion that conjugate 1 induces significantly stronger immunological responses in mice than other conjugates.

### Antibody-mediated complement-dependent cytotoxicity (CDC) to cancer cells

The three cancer cell lines were also employed to further explore the potential anticancer activities of the antisera obtained from mice immunized with conjugates 1–5 using antibody-mediated complement-dependent cytotoxicity (CDC) experiments. Cancer cells were initially incubated with normal mouse serum or the antisera described above, followed by rabbit complement serum. The percent of cell lysis induced by sera was detected by the lactate dehydrogenase (LDH) assay.

All antisera showed significant cytotoxicity against MCF-7 and CT-26 cells compared to the normal mouse sera (NS), based on cell lysis rates (Fig. 8D and E). All antisera exhibited higher cytotoxicity against human breast cancer cell line MCF-7 (62%, 53%, 60%, 53% and 46% from conjugate 1 to 5) than the corresponding mouse colon cancer cell CT-26 (37%, 35%, 32%, 33% and 34%) under the same conditions, suggesting that the antisera had a better affinity for MCF-7. Nearly no lysis was observed with B16-F10 cancer cells using NS or antisera (Fig. 8F), due to the absence of surface STn antigens. Collectively, antisera induced by conjugates 1–5 mediated effective and specific CDC to tumor cells that express STn antigens on their surface, while showing negligible toxicity toward STn negative cells.

### Tumor challenge studies

Inspired by the strength and diversity of anti-STn responses induced by conjugates 1–4, the immunotherapeutic efficacy was evaluated in terms of inhibiting tumor growth and prolonging the survival of tumor-bearing animals. Nine groups of female BALB/c mice (6–8 weeks) were employed to test against subcutaneous CT-26 tumors. One group only received PBS as the control. Six groups were intravenously injected with a low dose of the chemotherapeutic drug cyclophosphamide (CP, 100 mg kg^−1^) 1 day before vaccination, and received PBS, 1–4 (0.1 mL liposome containing 10 μg of STn), and 5/Al (0.1 mL emulsion containing 2 μg of STn) on day 1. The CP was to reduce T-regulatory cells and enhance the immune response.^68^ The remaining groups only received 1 and 4. Each group was boosted three times on days 14, 21 and 28 by s.c. injection of the same dose. One week after the fourth immunization, CT-26 tumor cells (1.5 × 10^5^ cells) were injected subcutaneously into the armpit of the mice. The tumor volume and mouse survival time were recorded for up to 50 days after the tumor challenge.

**Fig. 9:**
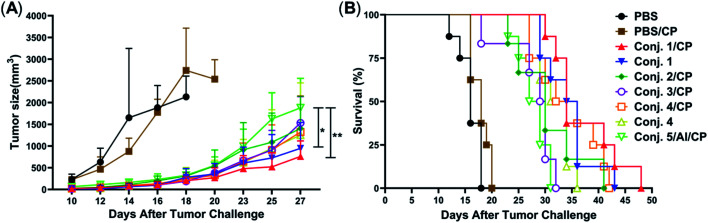
Evaluation of the immunotherapeutic efficacy of conjugates 1–5. (A) Tumor sizes with time. Data are presented as mean ± SD, *p < 0.05, **p < 0.01. (B) The survival rate with time. For each group, *n* = 8 mice, except for 2/CP and 3/CP group, where *n* = 6.

The tumor volume of both PBS groups (those that received CP or not) increased rapidly and all mice died within 20 days (Fig. 9). There was no significant difference between the group receiving PBS/CP and the group receiving CP alone, indicating that CP treatment alone does not obviously impact the proliferation of CT-26 cells at the dose administered. Compared to PBS groups, the tumor size in all groups receiving conjugate doses was reduced significantly (Fig. 9A). The decrease of the tumor growth rates of mice vaccinated with conjugates followed the order 1/CP > 1 > 2/CP ∼ 3/CP ∼ 4/CP ∼ 4 > 5/Al/CP, indicating that conjugates with or without CP can effectively inhibit tumor growth. Meanwhile, unlike the PBS groups, almost all mice were alive on day 20 in treatment groups (Fig. 9B), and all groups still had a survival rate of more than 50% on day 30. The average survival time of immunized groups followed the order 1/CP > 4/CP ∼ 1 > 2/CP ∼ 4 > 3/CP ∼ 5/Al/CP, indicating that conjugates with or without CP can effectively prolong the survival time of mice receiving tumor challenge. This result also reveals that the combination of CP and conjugates may provide higher protection than conjugates alone. All of the above results suggest that our designed conjugates are more effective than glycoprotein vaccine 5 with an Al adjuvant *in vivo*. In addition, conjugate 1, which showed the highest protection among all conjugates tested, has strong potential as a vaccine candidate.

## Conclusions

Fully synthetic self-adjuvanting TACA-based vaccines are promising agents for cancer immunotherapy. In the development of functional conjugate cancer vaccines, an essential component is the carrier molecule, which can stimulate the relevant immune system and elicit the most appropriate type of immunity to kill cancer cells. Here, we demonstrate for the first time that Mincle ligands have attractive immunostimulant and adjuvant activities as a carrier molecule for the preparation of potent fully synthetic carbohydrate-based cancer vaccines. Using the weakly immunogenic STn as the model antigen, four conjugates of STn–vizantin, STn–TDB, (STn)_2_–vizantin and (STn)_2_–TDB were successfully designed and prepared through a facile and efficient method. Compared to glycoprotein vaccine, these fully synthetic vaccines had the advantages of well-defined structures, convenient characterization and easy quality control.

All conjugates 1–4 can recognize and bind to hMincle protein, inducing BMDMs to produce inflammatory cytokines. Their efficiencies are comparable to those of Mincle ligands TDB and vizantin. Immunological evaluation demonstrated that like semisynthetic glycoprotein vaccine 5/Al, all our synthetic conjugates could effectively induce strong and consistent T cell-dependent immunity with switching from IgM to IgG in mice, as is desirable for therapeutic cancer vaccines. The difference is that conjugates 1–4 were self-adjuvanting, and thus were administrated without the use of an external adjuvant. These characteristics would not only simplify their clinical use but also contribute to stabilizing their properties. The assessment of antibody isotypes and ELISpot assays suggest that conjugates 1–4 could stimulate both Th1 and Th2 immune responses, indicating both activations of humoral and cellular immune responses. The IFN-γ/IL-4 ratios of the conjugates revealed that conjugates 1, 3, and 4 elicit a predominantly humoral immunity response. In contrast, conjugate 2 elicits a predominantly cellular immunity response. Notably, a strong immune response of conjugate 5 against CRM197 was observed by ELISA (the CRM197-specific antibody titer was 1.23-fold higher than the STn-specific antibody titer), indicating that the carrier molecule CRM197 protein might suppress the immune response to the carbohydrate antigen. However, our designed vaccines did not have this problem as the triazolyl linker and carrier molecule-specific antibody titer were both much lower than the STn-specific antibody titer. FACS studies and CDC experiments showed that all antibodies induced by conjugates 1–4 could specifically recognize, bind and generate complement-dependent cytotoxicity against STn positive cancer cells, leading to tumor cell lysis, while having negligible toxicity on STn negative cells. More importantly, all conjugates could effectively reduce the tumor volume and prolong the survival time *in vivo*, and all their therapeutic effects were better than that of STn-CRM197/Al. Consequently, all immunological results have suggested that Mincle ligands are a promising platform for the development of new carbohydrate-based vaccine carriers with self-adjuvanting properties for the treatment of cancer, and it is worth additional investigation and development.

Overall, immunological results show that conjugate 1 has the best therapeutic effect, indicating that vizantin as a built-in adjuvant can more effectively improve the immunogenicity of STn than TDB and CRM197. This result also revealed that the lipid structure and length had a significant impact on the immunology of the Mincle agonist, and the trehalose derivative containing two shorter lipid chains exhibited more effective immunostimulatory activities. For conjugates carried by vizantin, a vaccine containing a single STn antigen (conjugate 1) showed more effectiveness than those containing two STn antigens (conjugate 3). In contrast, the TDB-carried conjugate containing two STn antigens (conjugate 4) showed more efficacy than that with only one (conjugate 2). These results provide support for further optimization and additional investigation into Mincle agonists as self-adjuvanting cancer treatments.

In summary, a new strategy based on the Mincle agonist to construct synthetic cancer vaccines was first established. The Mincle agonist carried vaccines could induce BMDMs to produce inflammatory cytokines in a Mincle-dependent manner. All conjugates obviously elicit robust humoral and T cell-dependent immune responses and effectively protect mice from tumor challenge alone. Compared to conventional glycoprotein conjugate vaccines, these fully synthetic conjugate vaccines will not cause “epitope suppression” and do not require an external adjuvant. This vaccine approach provides a new direction for the design, application and promotion of fully synthetic vaccines.

## Experimental section

### General information

All starting materials and reagents were obtained commercially and used without further purification unless otherwise specified. 4 Å molecular sieves were flame-dried under vacuum and cooled to rt under a N_2_ atmosphere immediately before use. The reactions were monitored by thin-layer chromatography (TLC) on glass-packed precoated silica gel plates and visualized with a UV detector or charring with 10% H_2_SO_4_ in EtOH (v/v). The purification of products was accomplished by flash column chromatography on silica gel (200–300 mesh). NMR spectra were recorded on a Bruker Avance III 400 or Avance II 600 spectrometer (^1^H at 400 or 600 MHz, ^13^C at 100 or 150 MHz) with chemical shifts reported in ppm using TMS as the internal standard. Signal splitting patterns are described as singlet (s), doublet (d), triplet (t), quartet (q), or multiplet (m), with coupling constants (*J*) in hertz. MALDI-TOF mass spectrometry was performed with a Bruker Ultraflex instrument by applying the matrix of 2,5-dihydroxybenzoic acid (DHB). The high resolution electron spray ionization mass spectra (HR-ESI-MS) were obtained using a Waters Micromass-LCT Premier-XE mass spectrometer.

### Materials, reagents, and animals

DSPC, cholesterol and CP were purchased from Sigma Aldrich. Alum adjuvant was purchased from Thermo Fisher. Human Mincle-Fc and hMincle-his proteins were purchased from SinoBiological and Novoprotein, respectively. MCF-7, CT-26 and B16-F10 cancer cells were purchased from American Type Culture Collection (ATCC). Minimum Eagle's medium (MEM), RPMI medium 1640 and fetal bovine serum (FBS) were purchased from Gibico. Trypsin–EDTA was purchased from Invitrogen. HRP-linked goat anti-mouse kappa, IgM, IgG1, IgG2a, IgG2b, and IgG3 antibodies were purchased from Abcam. The FITC-labeled goat anti-mouse IgG antibody and LDH Cytotoxicity Detection Kit were purchased from Beyotime Biotechnology. Rabbit complements were purchased from Merck. Female BALB/c mice used for immunological studies were purchased from Southern Medical University (Guangzhou, China).

### Synthesis of compound 23

To a mixture of compound 20 (40 mg, 29 μmol), 22 (17.9 mg, 30.8 μmol), and CuI (53.3 mg, 0.28 mmol) in THF (2 mL) and MeOH (2 mL), DIEA was added (50 μL, 0.28 mmol). After the reaction mixture was stirred at rt for 24 h, it was diluted with MeOH and filtered. The filtrate was concentrated in a vacuum, and the residue was purified by silica gel column chromatography using MeOH/DCM (1 : 20, v/v) as the eluent to give the desired product as a white solid (27.4 mg, 48%). ^1^H NMR (600 MHz, CD_3_OD/CDCl_3_) *δ* 7.55 (s, 1H), 7.40–7.24 (m, 20H), 5.21–3.44 (m, 37H, 8H of Ar–CH_2_, 29H of sugar and linker), 2.76–2.72 (m, 1H), 2.1 (s, 4H), 2.05 (m, 6H, –NHAc), 1.84–1.77 (m, 4H), 1.62–1.58 (m, 1H), 1.46–1.43 (m, 4H), 1.26 (s, 64H, CH_2_ of lipid), 0.89 (t, *J* = 9.6 Hz, 12H, CH_3_ of lipid). ^13^C NMR (150 MHz, CD_3_OD/CDCl_3_) *δ* 180.70, 178.54, 178.11, 177.62, 177.53, 177.17, 148.97, 142.50, 142.43, 141.72, 141.35, 132.40, 132.30, 132.28, 131.89, 131.83, 131.59, 131.48, 131.44, 103.97, 101.67, 97.75, 97.67, 97.38, 97.31, 85.06, 84.96, 83.40, 82.73, 81.91, 79.36, 77.05, 76.83, 74.19, 74.07, 72.98, 72.68, 72.39, 71.63, 70.74, 70.23, 69.95, 69.87, 68.52, 66.92, 66.29, 64.33, 58.25, 57.96, 56.59, 46.25, 46.18, 43.01, 42.94, 38.87, 38.79, 37.56, 35.75, 33.76, 33.48, 33.19, 30.32, 27.08, 26.51, 26.01, 22.19, 20.86, 17.86, 15.95, 14.78, 14.75. HRMS (ESI-TOF) *m*/*z*: [M + H]^+^ calcd for C_106_H_164_N_5_O_27_, 1939.1608; found, 1939.1629.

### Compound 24

The synthesis of compound 24 was similar to that of 23 except for using compound 21 instead of 20. White solid, yield: 56%. ^1^H NMR (600 MHz, CD_3_OD/CDCl_3_) *δ* 7.65 (s, 1H), 7.38–7.28 (m, 20H), 5.22–3.43 (m, 42H, 8H of Ar–CH_2_, 34H of sugar and linker), 2.76–2.72 (m, 1H), 2.1 (s, 4H), 2.08–1.98 (m, 6H, –NHAc), 1.80–1.62 (m, 2H), 1.60–1.50 (m, 4H), 1.26 (s, 72H, CH_2_ of lipid), 0.89 (t, *J* = 9.6 Hz, 6H, CH_3_ of lipid). ^13^C NMR (150 MHz, CD_3_OD/CDCl_3_) *δ* 176.83, 174.67, 174.47, 173.96, 173.92, 173.91, 173.90, 173.87, 138.58, 137.84, 137.50, 128.54, 128.44, 128.03, 127.76, 100.11, 97.84, 93.95, 93.60, 81.22, 81.10, 79.40, 78.78, 78.71, 77.98, 75.54, 75.49, 75.33, 74.89, 73.16, 72.91, 70.32, 70.12, 69.12, 66.01, 63.08, 62.54, 54.03, 42.28, 39.82, 34.13, 31.90, 29.67, 29.51, 29.44, 29.33, 29.24, 29.12, 24.88, 24.81, 23.22, 22.65, 14.00, 10.89. HRMS (ESI-TOF) *m*/*z*: [M + H]^+^ calcd for C_108_H_168_N_5_O_27_, 1967.1921; found, 1967.1908.

### Compound 27

The synthesis of compound 27 was similar to that of 23. White solid, yield: 52%. ^1^H NMR (400 MHz, CD_3_OD/CDCl_3_) *δ* 7.63 (s, 2H), 7.38–7.24 (m, 20H), 5.19–3.49 (m, 64H, 8H of Ar–CH_2_, 56H of sugar and linker), 2.1 (d, 4H), 1.96–2.07 (m, 12H, –NHAc), 1.76–1.90 (m, 4H), 1.26 (s, 64H, CH_2_ of lipid), 0.89 (t, *J* = 9.6 Hz, 12H, CH_3_ of lipid); HRMS (ESI-TOF) *m*/*z*: [M + 2H]^2+^ calcd for C_130_H_202_N_10_O_41_, 1279.7009; found, 1279.7037.

### Compound 28

The synthesis of compound 28 was similar to that of 23. White solid, yield: 53%. ^1^H NMR (600 MHz, CD_3_OD/CDCl_3_) *δ* 7.74 (s, 1H), 7.67 (s, 1H), 7.38–7.24 (m, 20H), 5.19–3.49 (m, 62H, 8H of Ar–CH_2_, 54H of sugar and linker), 2.56–2.52 (m, 2H), 2.1 (s, 4H), 2.08–1.98 (m, 12H, –NHAc), 1.80–1.62 (m, 2H), 1.60–1.50 (m, 4H), 1.26 (s, 72H, CH_2_ of lipid), 0.89 (t, *J* = 9.6 Hz, 6H, CH_3_ of lipid). ^13^C NMR (150 MHz, CD_3_OD/CDCl_3_) *δ* 180.84, 180.65, 178.61, 178.05, 177.95, 149.37, 142.37, 142.17, 141.75, 141.56, 132.60, 132.26, 132.14, 131.64, 131.43, 131.23, 130.08, 104.04, 103.98, 102.13, 101.67, 100.05, 98.99, 85.51, 83.82, 83.02, 82.79, 79.61, 78.31, 77.17, 76.73, 76.41, 76.28, 73.97, 73.84, 73.69, 73.19, 73.05, 72.45, 72.34, 72.11, 71.39, 70.72, 69.66, 68.77, 68.45, 68.22, 66.52, 66.25, 64.29, 58.08, 53.67, 53.22, 52.97, 44.20, 43.69, 38.04, 37.98, 35.75, 33.49, 33.32, 33.29, 33.17, 33.08, 33.04, 32.93, 28.75, 27.17, 26.88, 26.48, 25.91, 17.69, 4.68, 3.44. HRMS (ESI-TOF) *m*/*z*: [M + 2H]^2+^ calcd for C_132_H_206_N_10_O_41_, 1293.7165; found, 1293.7167.

### Compound 1

A mixture of compound 23 (30 mg, 15.5 μmol) and 10% Pd/C (30 mg) in DCM/MeOH/H_2_O (3 : 3 : 0.1, v/v, 20 mL) was stirred under a hydrogen atmosphere at rt for 24 h. Then, the reaction mixture was diluted with DCM and filtered through a Celite pad. The filtrate was washed with water, and the organic layer was concentrated in a vacuum to give 1 as a white solid (14.9 mg, 61% yield). ^1^H NMR (600 MHz, CD_3_OD/CDCl_3_) *δ* 8.12 (s, 1H), 5.10–2.67 (m, 27H, 27H of sugar and linker), 2.32–2.28 (m, 4H), 2.32–2.28 (m, 4H), 2.05 (s, 6H, –NHAc), 1.85 (s, 2H), 1.63–1.60 (m, 1H), 1.46–1.40 (m, 4H), 1.26 (s, 64H, CH_2_ of lipid), 0.89 (t, *J* = 9.6 Hz, 12H, CH_3_ of lipid). HRMS (ESI-TOF) *m*/*z*: [M + H]^+^ calcd for C_78_H_140_N_5_O_27_, 1578.9730; found, 1578.9726.

### Compound 2

The synthesis of compound 2 was similar to that of 1 except for using compound 24 instead of 23. White solid, yield: 63%. ^1^H NMR (600 MHz, CD_3_OD/CDCl_3_) *δ* 8.12 (s, 1H), 5.11–3.43 (m, 35H, 35H of sugar and linker), 2.72–2.70 (m, 1H), 2.41–2.38 (m, 4H), 2.07 (s, 6H, –NHAc), 1.80–1.78 (m, 1H), 1.60–1.62 (m, 4H), 1.26 (s, 72H, CH_2_ of lipid), 0.89 (t, *J* = 9.6 Hz, 6H, CH_3_ of lipid). HRMS (ESI-TOF) *m*/*z*: [M + H]^+^ calcd for C_80_H_144_N_5_O_27_, 1607.0043; found, 1607.0044.

### Compound 3

The synthesis of compound 3 was similar to that of 1. White solid, yield: 55%. ^1^H NMR (600 MHz, CD_3_OD/CDCl_3_) *δ* 7.63 (s, 2H), 5.19–3.49 (m, 54H of sugar and linker), 2.66–2.57 (m, 2H), 2.1 (s, 4H), 1.93–1.75 (m, 12H, –NHAc), 1.69–1.62 (m, 2H), 1.55–1.48 (m, 2H), 1.21 (s, 64H, CH_2_ of lipid), 0.89 (t, *J* = 9.6 Hz, 12H, CH_3_ of lipid). HRMS (ESI-TOF) *m*/*z*: [M + Na]^+^ calcd for C_102_H_176_N_10_NaO_41_, 2220.1887; found, 2220.1807.

### Compound 4

The synthesis of compound 4 was similar to that of 1. White solid, yield: 57%. ^1^H NMR (600 MHz, CD_3_OD/CDCl_3_) *δ* 8.09 (s, 2H), 5.10–3.00 (m, 54H of sugar and linker), 2.76–2.72 (m, 2H), 2.41–2.37 (t, 4H), 2.05 (s, 12H, –NHAc), 1.85–1.75 (m, 2H),1.66–1.62 (m, 4H), 1.26 (s, 72H, CH_2_ of lipid), 0.89 (t, *J* = 9.6 Hz, 6H, CH_3_ of lipid). HRMS (ESI-TOF) *m*/*z*: [M + 2H]^2+^ C_104_H_182_N_10_O_41_, 1113.6226; found, 1113.6251.

### General procedure for the preparation of STn-CRM197 and STn-HSA

A mixture of compound 22 (25 mg, 43 μmol) and Pd/C (10%, 25 mg) in MeOH/H_2_O (1 : 1, v/v, 6 mL) was stirred under a hydrogen atmosphere at rt for 24 h. The reaction mixture was diluted with MeOH and filtered through a Celite pad. The filtrate was concentrated in a vacuum to give compound 29 as a white solid (17.9 mg, 75% yield). Then, a solution of compound 29 (2.0 mg, 3.6 μmol) and bis(2,5-dioxopyrrolidin-1-yl) octanedioate (20.0 mg, 54.3 μmol) in DMF (0.5 mL) was stirred at rt for 5 h. After removing the solvent, the crude product was washed with EA 6 times to give 30, which was used directly for the next step without purification.

A solution of the activated oligosaccharide 30 and CRM197 or HSA (5 mg) in 0.4 mL of 0.1 M PBS buffer was gently stirred at rt for 2.5 days. The mixture was purified on a Biogel A 0.5 column with 0.1 M PBS buffer as the eluent. The combined fractions containing the glycoconjugate indicated by the bicinchoninic acid (BCA) assay for proteins were dialyzed in distilled water for 2 days, and then lyophilized to obtain the desired conjugates 5 and 6 as white solids.

### Human Mincle binding assay

Conjugates 1–4, vizantin, and TDB were dissolved in MeOH (10 μg mL^−1^) and added to 96-well plates (100 μL per well). The solvents were evaporated at room temperature. The coated plates were incubated with 100 μL hMincle-Fc or hMincle-His protein [1 μg mL^−1^ in binding buffer (20 mMTris–HCl, 150 mM NaCl, 1 mM CaCl_2_, 2 mM MgCl_2_, and pH = 7.0)] for 1.5 h at 37 °C. After washing with PBST, HRP-rabbit anti-human IgG Fc or HRP-anti His was added, and the plates were incubated for another 1 h. Finally, a colorimetric substrate 3,3′,5,5′-tetramethylbenzidine (TMB) was added and the OD value at 450 nm was measured.

### Cytokine of murine bone-marrow derived macrophage analysis

The preparation of bone-marrow derived macrophages of BALB/c mice was according to a previously described procedure.^43^ The harvested BMDMs (1 × 10^4^ cells per well) were seeded in a 96-well plate and incubated at 37 °C overnight. Then, LPS (100 ng per well), vizantin, TDB or conjugates 1–4 (1 nmol per well) were added. After incubation for 24 h, TNF-α and IL-6 levels were determined *via* ELISA according to the manufacturer's instructions.

### Preparation of vaccine liposomes

The preparation of vaccine liposomal formulation was similar to a literature reported protocol.^69^ Briefly, a mixture of the conjugate, DSPC, and cholesterol in a molar ratio of 10 : 65 : 50 was dissolved in a mixture of CH_2_Cl_2_ and MeOH (1 : 1, v/v). The solvents were removed under reduced pressure through rotary evaporation, and a thin lipid film was formed on the vial wall. The film was hydrated with HEPES buffer (pH 7.5) and shaken on a vortex mixer. Finally, the mixture was sonicated at rt for 20 min to give the liposomal formulation of conjugate 1, 2, 3, and 4, respectively. The average diameter of 1, 2, 3, and 4 was 700.3 ± 192.0 (SD), 717.0 ± 62.9, 890.5 ± 94.4, and 789.5 ± 51.6 nm, respectively. The polydispersity index (PDI) of 1, 2, 3, and 4 is around 0.2200, 0.2150, 0.3060, and 0.111, respectively. The emulsion of STn-CRM197 with an alum adjuvant was prepared according to the protocol reported in the literature.^70^ Generally, conjugate 5 was dissolved in PBS buffer and thoroughly mixed with an alum adjuvant following the manufacturer's instructions.

### Mouse immunization

Each group of six female BALB/c mice (6–8 weeks old) was immunized subcutaneously with 0.1 mL liposome solution containing 10 μg of STn or 0.1 mL emulsion containing 2 μg of STn on day 1. Then, the mice were boosted 3 times on day 14, day 21 and day 28 after the initial immunization by s.c. injection of the same vaccine and using the same immunization protocol. Blood samples were collected from the tail vein of each mouse on day 0 before the initial immunization and on days 27 and 38 after the first injection. Then, the blood samples were clotted to obtain antisera and stored at −80 °C before use. The animal protocol for this study was approved by the Guangzhou University of Traditional Chinese Medicine Animal Care and Use Committee, and all of these mice were maintained under specific pathogen-free conditions (License number: SYXK (Guangzhou) 2019-0144).

### ELISA protocol

The conjugate of STn-HSA 6 (2 μg mL^−1^, 100 μL) was dissolved in 0.1 M carbonate buffer (pH = 9.6), and then was added to each well of a 96-well microtiter plate. After incubation at 4 °C overnight, followed by at 37 °C for 1 h, the plate was washed with PBS buffer containing 0.05% Tween-20 (PBST) 3 times, and treated with a blocking buffer (1% BSA in PBS) at rt for 1 h. Afterward, a pooled or an individual antiserum, which was diluted with serial half-log dilutions from 1 : 300 to 1 : 656 100 in PBS, was added to the coated plates (100 μL per well) and incubated at 37 °C for 2 h. After washing with PBST, the plates were incubated with a 1 : 1000 diluted solution of HRP-linked goat anti-mouse kappa, IgG, and IgM, and 1 : 2000 diluted solutions of IgG1, IgG2a, IgG2b, and IgG3 antibodies, respectively. The plates were shaken at 400 rpm for 1 h and subsequently washed with PBS. 100 μL TMB solution was added to the plates and incubated in the dark for 20 min. Finally, 100 μL 0.5 M H_2_SO_4_ solution was added and the optical density (OD) value was detected using a microplate reader at 450 nm wavelength with 570 nm as a reference. For titer analysis, the best fit line was obtained with the OD value as the ordinate and the natural logarithm of the serum dilutions was set as the abscissa. The antibody titer was defined as the dilution number when the OD value was 0.2.

### Protocols for cytokine assay

IFN-γ and IL-4 were detected by using ELISpot kits (DAKEWE, Cat#: 2210005, Cat#: 2210402). The 96-well plate was coated with a monoclonal antibody specific for mouse IFN-γ or IL-4 at 4 °C overnight. After washing with PBS, 200 μL RPMI-1640 was added to the plates. Splenocytes from immunized mice were seeded into the plate (5 × 10^5^ cells per well), and incubated with the corresponding conjugate (0.1 μg of STn per well) at 37 °C for 24 h. Then, the plate was washed with washing butter six times and incubated with 1 : 100 dilution of biotinylated anti-mouse IFN-γ or IL-4 antibodies at 37 °C for 1 h. After washing, 1 : 100 dilution of streptavidin–alkaline phosphatase was added and incubated at 37 °C for 1 h again. The plate was washed and a substrate solution of 3-amino-9-ethylcarbazole (AEC) chromogen was added and left in the dark for 30 minutes. The reaction was quenched with ddH_2_O, and the plate was dried with air. The number of spots was counted using an Immunospot Analyzer.

### Protocols for FACS

The cell samples used for FACS analyses were prepared according to a reported protocol.^53^ MCF-7 cancer cells were incubated in MEM containing 10% FBS and CT-26 and B16-F10 cells were incubated in RPMI-1640 containing 10% FBS. The cells were harvested with trypsin–EDTA solution. A suspension of a 5.0 × 10^5^ target cell was incubated with 50 μL 1 : 10 dilution of normal mouse serum or anti-serums at 4 °C for 1 h. After washing with FACS buffer three times, 50 μL 1 : 50 dilution of FITC-labeled goat anti-mouse IgG antibody was added to the cell suspension and incubated at 4 °C for 1 h. The resulting cells were collected and washed with FACS buffer three times. Percent positive cells and MFI of stained cells were recorded using a FACS flow cytometer. Data were processed and analyzed with FlowJo software.

### Protocols for CDC

CDC was determined using a commercially available LDH cytotoxicity detection kit following the manufacturer's instructions. Targeted cancer cells (1.0 × 10^4^ cells per well) were seeded in a 96-well plate and incubated at 37 °C overnight. After washing, the plates were incubated with 100 μL of 1 : 20 dilution normal mouse serum or a day 38 antiserum at 37 °C for 2 h. The wells were washed twice and then incubated with 100 μL of 1 : 10 dilution rabbit complement serum at 37 °C for 1 h. For low control (spontaneous LDH release), no antiserum was added. For high control (maximum LDH release), rabbit complement serum was replaced with 100 μL of 5% Triton X-100. After incubation, 20 μL of supernatant from each well was carefully transferred to another 96-well plate containing 80 μL of DPBS. Then, 100 μL of the LDH cytotoxicity detection reagent was added to each well and the plate was incubated for 30 min in the dark. The optical absorption (*A*) of each well was read at 490 nm wavelength using a microplate reader. The percentage of cell lysis was calculated according to the following equation:
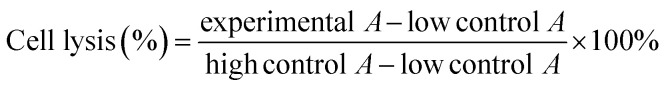
where “experimental *A*” is the optical absorption at 490 nm of cells lysed by the treatment of antiserum, “low control *A*” is the optical absorption of cells lysed without serum treatment, and “high control *A*” is the optical absorption of cells lysed with 5% Triton X-100 solution.

### Protocols for tumor immunotherapy

Nine groups of female BALB/c mice (6–8 weeks) were used for testing against subcutaneous CT-26 tumors. One group only received PBS as the control. Six groups were intravenously injected with a low dose of the chemotherapeutic drug cyclophosphamide (CP, 100 mg kg^−1^) 1 day before vaccination, and received PBS, 1–4 (0.1 mL liposome containing 10 μg of STn), and 5/Al (0.1 mL emulsion containing 2 μg of STn) on day 1. The remaining groups only received 1 and 4. Each group was boosted three times on days 14, 21 and 28 by s.c. injection of the same dose. One week after the fourth immunization, CT-26 tumor cells (1.5 × 10^5^ cells) were injected subcutaneously into the armpit of the mice. The tumor volume and mouse survival time were recorded for up to 50 days after the tumor challenge. The length, width and height of each tumor were measured using a digital slide caliper, and the tumor volume was calculated by using the formula: π/6 × length × width^2^.

## Author contributions

G. Liao conceived the project and designed the experiments. Q. Lian performed the sample preparation, characterization and biological experiments. L. Chen and X. Qi performed the hMincle binding assay and cytokine of murine bone-marrow derived macrophage test. R. Zhang synthesized the Mincle ligands vizantin and TDB. W. Li, D. Yang and L. Gao synthesized the self-adjuvanting conjugates and glycoprotein conjugates. X. Luo and G. Liao wrote the paper and prepared the manuscript and ESI.† X. Luo, Q. Lian and W. Li contributed equally. G. Liao, Z. Liu and X. Luo contributed to funding acquisition and supervision. All authors discussed the results and commented on the manuscript.

## Conflicts of interest

There are no conflicts to declare.

## Supplementary Material

SC-012-D1SC05736G-s001

## References

[cit1] Schumacher T. N., Schreiber R. D. (2015). Science.

[cit2] Feng D., Shaikh A. S., Wang F. (2016). ACS Chem. Biol..

[cit3] Dube D. H., Bertozzi C. R. (2005). Nat. Rev. Drug Discovery.

[cit4] Holmberg L. A., Sandmaier B. M. (2004). Expert Rev. Vaccines.

[cit5] Huang Y. L., Hung J. T., Cheung S. K. C., Lee H. Y., Chu K. C., Li S. T., Lin Y. C., Ren C. T., Cheng T. J. R., Hsu T. L., Yu A. L., Wu C. Y., Wong C. H. (2013). Proc. Natl. Acad. Sci. U. S. A..

[cit6] van der Heiden M., Duizendstra A., Berbers G. A. M., Boots A. M. H., Buisman A. M. (2017). Vaccine.

[cit7] Eggermont A. M. M., Suciu S., Rutkowski P., Marsden J., Santinami M., Corrie P., Aamdal S., Ascierto P. A., Patel P. M., Kruit W. H., Bastholt L., Borgognoni L., Bernengo M. G., Davidson N., Polders L., Praet M., Spatz A. (2013). J. Clin. Oncol..

[cit8] Kightlinger W., Warfel K. F., Delisa M. P., Jewett M. C. (2020). ACS Synth. Biol..

[cit9] Astronomo R. D., Burton D. R. (2010). Nat. Rev. Drug Discovery.

[cit10] Wang J., Mamuti M., Wang H. (2020). ACS Biomater. Sci. Eng..

[cit11] Lee H. Y., Chen C. Y., Tsai T. I., Li S. T., Lin K. H., Cheng Y. Y., Ren C. T., Cheng T. J. R., Wu C. Y., Wong C. H. (2014). J. Am. Chem. Soc..

[cit12] Du J. J., Wang C. W., Xu W. B., Zhang L., Tang Y. K., Zhou S. H., Gao X. F., Yang G. F., Guo J. (2020). iScience.

[cit13] Gaidzik N., Kaiser A., Kowalczyk D., Westerlind U., Gerlitzki B., Sinn H. P., Schmitt E., Kunz H. (2011). Angew. Chem., Int. Ed..

[cit14] Ragupathi G., Koide F., Livingstoi P. O., Cho Y. S., Endo A., Wan Q., Spassova M. K., Keding S. J., Allen J., Ouerfelli O., Wilson R. M., Danishefsky S. J. (2006). J. Am. Chem. Soc..

[cit15] Adamo R., Nilo A., Castagner B., Boutureira O., Berti F., Bernardes G. J. L. (2013). Chem. Sci..

[cit16] Schutze M. P., Leclerc C., Vogel F. R., Chedid L. (1987). Cell. Immunol..

[cit17] Woodruff M. C., Kim E. H., Luo W., Pulendran B. (2018). Cell Rep..

[cit18] Herzenberg L. A., Tokuhisa T., Herzenberg L. A. (2012). Nature.

[cit19] Gilewski T., Ragupathi G., Bhuta S., Williams L. J., Musselli C., Zhang X. F., Bencsath K. P., Panageas K. S., Chin J., Hudis C. A., Norton L., Houghton A. N., Livingston P. O., Danishefsky S. J. (2001). Proc. Natl. Acad. Sci. U. S. A..

[cit20] Ingale S., Wolfert M. A., Gaekwad J., Buskas T., Boons G. J. (2007). Nat. Chem. Biol..

[cit21] Sarkar S., Lombardo S. A., Herner D. N., Talan R. S., Wall K. A., Sucheck S. J. (2010). J. Am. Chem. Soc..

[cit22] Wilkinson B. L., Day S., Malins L. R., Apostolopoulos V., Payne R. J. (2011). Angew. Chem..

[cit23] Zom G. G., Welters M. J. P., Loof N. M., Goedemans R., Lougheed S., Valentijn R. R. P. M., Zandvliet M. L., Meeuwenoord N. J., Melief C. J. M., de Gruijl T. D., Van der Marel G. A., Filippov D. V., Ossendorp F., Van der Burg S. H. (2016). Oncotarget.

[cit24] van den Ende T. C., Heuts J. M. M., Gential G. P. P., Visser M., van de Graaff M. J., Ho N. I., Jiskoot W., Valentijn A. R. P. M., Meeuwenoord N. J., Overkleeft H. S., Codée J. D. C., van der Burg S. H., Verdegaal E. M. E., van der Marel G. A., Ossendorp F., Filippov D. V. (2020). ChemBioChem.

[cit25] Gao J., Guo Z. (2018). Med. Res. Rev..

[cit26] Wang Q., Zhou Z., Tang S., Guo Z. (2012). ACS Chem. Biol..

[cit27] Li Q., Guo Z. (2018). Molecules.

[cit28] Ghosh S., Trabbic K. R., Shi M., Nishat S., Eradi P., Kleski K. A., Andreana P. R. (2020). Chem. Sci..

[cit29] De Silva R. A., Wang Q., Chidley T., Appulage D. K., Andreana P. R. (2009). J. Am. Chem. Soc..

[cit30] Shi M., Kleski K. A., Trabbic K. R., Bourgault J. P., Andreana P. R. (2016). J. Am. Chem. Soc..

[cit31] Yin X. G., Chen X. Z., Sun W. M., Geng X. S., Zhang X. K., Wang J., Ji P. P., Zhou Z. Y., Baek D. J., Yang G. F., Liu Z., Guo J. (2017). Org. Lett..

[cit32] Broecker F., Götze S., Hudon J., Rathwell D. C. K., Pereira C. L., Stallforth P., Anish C., Seeberger P. H. (2018). J. Med. Chem..

[cit33] Yin X. G., Lu J., Wang J., Zhang R. Y., Wang X. F., Liao C. M., Liu X. P., Liu Z., Guo J. (2021). J. Med. Chem..

[cit34] Painter G. F., Burn O. K., Hermans I. F. (2021). Mol. Immunol..

[cit35] Micoli F., Del Bino L., Alfini R., Carboni F., Romano M. R., Adamo R. (2019). Expert Rev. Vaccines.

[cit36] Pifferi C., Fuentes R., Fernández-Tejada A. (2021). Nat. Rev. Chem..

[cit37] Jones L. H. (2015). Nat. Chem..

[cit38] Matsumoto M., Tanaka T., Kaisho T., Sanjo H., Copeland N. G., Gil- bert D. J., Jenkins N. A., Akira S. (1999). J. Immunol..

[cit39] Yamasaki S., Ishikawa E., Sakuma M., Hara H., Ogata K., Saito T. (2008). Nat. Immunol..

[cit40] Lobato-Pascual A., Saether P. C., Fossum S., Dissen E., Daws M. R. (2013). Eur. J. Immunol..

[cit41] Furukawa A., Kamishikiryo J., Mori D., Toyonaga K., Okabe Y., Toji A., Kanda R., Miyake Y., Ose T., Yamasaki S., Maenaka K. (2013). Proc. Natl. Acad. Sci. U. S. A..

[cit42] Yamamoto H., Oda M., Nakano M., Watanabe N., Yabiku K., Shibutani M., Inoue M., Imagawa H., Nagahama M., Himeno S., Setsu K., Sakurai J., Nishizawa M. (2013). J. Med. Chem..

[cit43] Foster A. J., Nagata M., Lu X., Lynch A. T., Omahdi Z., Ishikawa E., Yamasaki S., Timmer M. S. M., Stocker B. L. (2018). J. Med. Chem..

[cit44] Kallerup R. S., Madsen C. M., Schiøth M. L., Franzyk H., Rose F., Christensen D., Korsholm K. S., Foged C. (2015). Eur. J. Pharm. Biopharm..

[cit45] Smith D. G. M., Hosono Y., Nagata M., Yamasaki S., Williams S. J. (2020). Chem. Commun..

[cit46] Rasheed O. K., Buhl C., Evans J. T., Ryter K. T. (2021). ChemMedChem.

[cit47] Ryter K. T., Ettenger G., Rasheed O. K., Buhl C., Child R., Miller S. M., Holley D., Smith A. J., Evans J. T. (2020). J. Med. Chem..

[cit48] Ostrop J., Jozefowski K., Zimmermann S., Hofmann K., Strasser E., Lepenies B., Lang R. (2015). J. Immunol..

[cit49] Hong H., Zhou Z., Zhou K., Liu S., Guo Z., Wu Z. (2019). Chem. Sci..

[cit50] Gao L., Lian Q., Ma L., Su S., Yang M., Fang Y., Liu Z., Luo X., Liao G. (2021). Chin. Chem. Lett..

[cit51] Zhou Z., Mandal S. S., Liao G., Guo J., Guo Z. (2017). Sci. Rep..

[cit52] Zeng L., Liao Z., Li W., Yuan Q., Wu P., Gu Z., Liu Z., Liao G. (2020). Chin. Chem. Lett..

[cit53] Zhou Z., Liao G., Mandal S. S., Suryawanshi S., Guo Z. (2015). Chem. Sci..

[cit54] Davidson B., Berner A., Nesland J. M., Risberg B., Kristensen G. B., Tropé C. G., Bryne M. (2000). Hum. Pathol..

[cit55] Munkley J. (2016). Int. J. Mol. Sci..

[cit56] Song C., Zheng X. J., Guo H., Cao Y., Zhang F., Li Q., Ye X. S., Zhou Y. (2019). Glycoconjugate J..

[cit57] Miles D., Roché H., Martin M., Perren T. J., Cameron D. A., Glaspy J., Dodwell D., Parker J., Mayordomo J., Tres A., Murray J. L., Ibrahim N. K. (2011). Oncologist.

[cit58] Gilewski T. A., Ragupathi G., Dickler M., Powell S., Bhuta S., Panageas K., Koganty R. R., Chin-Eng J., Hudis C., Norton L., Houghton A. N., Livingston P. O. (2007). Clin. Cancer Res..

[cit59] Chang T. C., Manabe Y., Fujimoto Y., Ohshima S., Kametani Y., Kabayama K., Nimura Y., Lin C. C., Fukase K. (2018). Angew. Chem., Int. Ed..

[cit60] Buskas T., Li Y., Boons G. J. (2004). Chem.–Eur. J..

[cit61] Sarpe V. A., Kulkarni S. S. (2013). Org. Biomol. Chem..

[cit62] Wu J., Guo Z. (2006). Bioconjugate Chem..

[cit63] Wang Q., Ekanayaka S. A., Wu J., Zhang J., Guo Z. (2008). Bioconjugate Chem..

[cit64] Liao G., Zhou Z., Burgula S., Liao J., Yuan C., Wu Q., Guo Z. (2015). Bioconjugate Chem..

[cit65] Zinov’ev D. V., Sole P. (2004). Probl. Peredachi Inf.i.

[cit66] Svennerholm L. (1957). Biochim. Biophys. Acta.

[cit67] Khan A., Kodar K., Timmer M. S. M., Stocker B. L. (2018). Tetrahedron.

[cit68] Song C., Zheng X. J., Liu C. C., Zhou Y., Ye X. S. (2017). Oncotarget.

[cit69] Zhou Z., Mondal M., Liao G., Guo Z. (2014). Org. Biomol. Chem..

[cit70] Pan Y., Chefalo P., Nagy N., Harding C., Guo Z. (2005). J. Med. Chem..

