# Supplementary material for: Fully synthetic Mincle-dependent self-adjuvanting cancer vaccines elicit robust humoral and T cell-dependent immune responses and protect mice from tumor development
Source: Chem Sci. 2021 Dec 1;12(48):15998–6013. doi: 10.1039/d1sc05736g (PMC8672726; doi:10.1039/d1sc05736g)
Supplement: SC-012-D1SC05736G-s001 [file SC-012-D1SC05736G-s001.pdf]

Electronic Supplementary Material (ESI) for Chemical Science.
This journal is © The Royal Society of Chemistry 2021

Supporting Information

Fully synthetic Mincle-dependent self-adjuvanting cancer vaccines elicit robust humoral and T cell-dependent immune responses and protect mice from tumor development

Xiang Luo,‡ Qinghai Lian,‡ Wenwei Li,‡ Liqing Chen, Renyu Zhang, Deying Yang, Lingqiang Gao, Xiaoxiao Qi, Zhongqiu Liu,* Guochao Liao*

Joint Laboratory for Translational Cancer Research of Chinese Medicine of the Ministry of Education of the People's Republic of China, International Institute for Translational Chinese Medicine, Guangzhou University of Chinese Medicine, Guangzhou 510006, China

‡ These authors contributed equally to this work

Table of contents

I. General Information S1

II. Preparation and Characterization of Conjugates 1 and 2 ....S1

III. Preparation and Characterization of STn S13

IV. Preparation and Characterization of Conjugates 3 and 4....................................S16

V. Analysis of Sialic Acid Loading of STn-CRM197 and STn-HSA S21

VI. Synthesis of Triazole-HSA Conjugate..S25

VII. Synthesis of Mincle Ligands Vizantin and TDB...S26

VIII. Binding affinity of conjugates 1-4 to hMincle S28

IX. Abilities of conjugates 1-4 to induce the production of TNF-α and IL-6...........S28

X. Liposomes Size Analysis of the Conjugates 1-4...S29

XI. Calculated Antibody Titers of ELISA Experiments S32

XII. ELISpot Assay...S35

XIII. FACS Analyses S36

XIV. Antibody-Mediated Complement-Dependent Cytotoxicity (CDC).................S36

XV. Tumor Challenge Studies S37

References...S38

NMR and MS Spectra of Synthesized Compounds S39

I. General Information

All starting materials and reagents were obtained commercially and used without further purification unless otherwise specified. Molecular sieves 4 Å were flame-dried under vacuum and cooled to rt under N_2 atmosphere immediately before use. The reactions were monitored by thin-layer chromatography (TLC) on glass-packed precoated silica gel plates and visualized by a UV detector or charring with 10% H_2SO_4 in EtOH (v/v). Purification of products was accomplished by flash column chromatography on silica gel (200-300 mesh). NMR spectra were recorded on a Bruker Avance III 400 or Avance II 600 spectrometer (^{1}H at 400 or 600 MHz, ^{13}C at 100 or 150 MHz) with chemical shifts reported in ppm using TMS as the internal standard. Signal splitting patterns are described as singlet (s), doublet (d), triplet (t), quartet (q), or multiplet (m), with coupling constants (*J*) in hertz. MALDI-TOF mass spectrometry was obtained with a Bruker Ultraflex instrument by applying the matrix of 2,5-dihydroxybenzoic acid (DHB). The high resolution electron spray ionization mass spectra (HR-ESI-MS) were obtained using a Waters Micromass-LCTPremier-XE mass spectrometer.

II. Preparation and Characterization of Conjugates 1 and 2

4,6,4′,6′-di-*O*-(*p*-methoxybenzylidene)-*α*,*α*-*D*-trehalose (13)

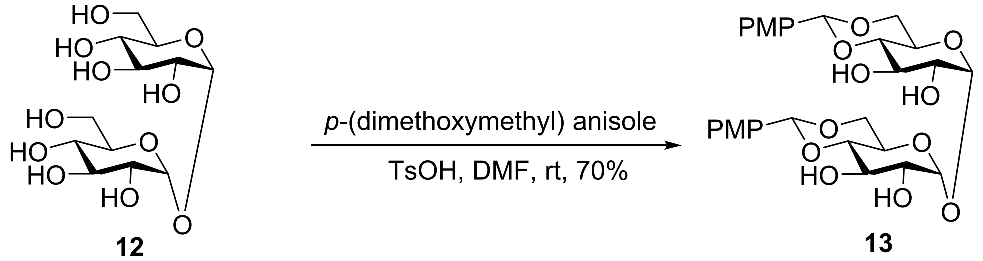

The synthesis of compound **13** was according to published procedure.[1] White solid, 70% yield. ^{1}H NMR (400 MHz，CD_3OD) δ 7.40 (d，*J* = 8.8 Hz, 4H)，6.87 (d，*J* = 8.8 Hz, 4H), 5.51 (s, 2H, PhCH), 5.11 (d, *J* = 3.8 Hz, 2H, H-1 and H-1′), 4.19 – 4.17 (m, 2H, H5 and H5′), 4.12 - 4.06 (m, 2H, H-3 and H-3′), 4.01 (t, *J* = 9.6 Hz, 2H, H-4

and H-4′), 3.77 (s, 6H), 3.69 (t, *J* = 10.4 Hz, 2H), 3.61 (dd, *J* = 9.2, 4.0 Hz, 2H), 3.45 (t, *J* = 9.6 Hz, 2H), 3.31 - 3.29 (m, 2H). HRMS (ESI-TOF) *m/z*: $[M + H]^+$ calcd for $C_{28}H_{35}O_{13}$, 579.2072; Found, 579.2067.

2,3,2′,3′-Tetra-*O*-benzyl-4,6,4′,6′-di-*O*-(*p*-methoxybenzylidene)-*α*,*α*-*D*-trehalose (14)

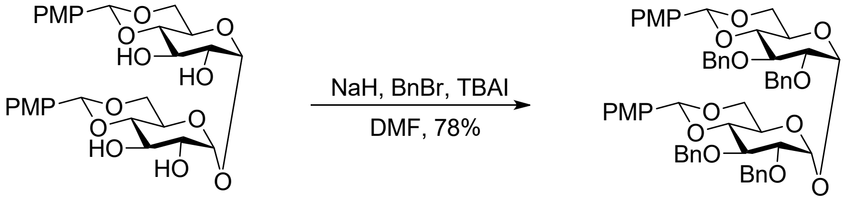

13 **14**

NaH (60% in oil, 1.64 g, 30.5 mmol) was added to a solution of compound **13** (11.9 g, 20.5 mmol) in anhydrous DMF (60 mL) at 0 °C under N_2 atmosphere. Then, benzyl bromide (14.6 mL, 123 mmol) and TBAI (751.2 mg, 2.03 mmol) were added and stirred for 3 h. After reaction for 18 h at rt, the reaction was quenched by MeOH, and filtered. The mixture was diluted with ethyl acetate (300 mL), and washed with saturated brine. The organic layer was dried over anhydrous Na_2SO_4, and concentrated under reduced pressure. The crude product was purified by recrystallization (ethyl acetate (EA): petroleum ether (PE) = 4:1) to furnish compound **14** as a white solid (14.9 g, 78%). ^{1}H NMR (400 MHz, $CDCl_3$) δ 7.51 – 7.24 (m, 28H), 5.55 (s, 2H, PhCH), 5.11 (d, *J* = 3.8 Hz, 2H, H-1 and H-1′), 4.96 (d, *J* =11.1 Hz, 2H, $PhCH_2O$)，4.85(d, *J* =11.1 Hz, 2H, $PhCH_2O$)，4.83(d, *J* = 12.0 Hz, 2H, $PhCH_2O$), 4.72 (d, *J* = 12.0 Hz, 2H, $PhCH_2O$), 4.27 (dt, *J* = 10.0, 4.8 Hz, 2H, H5 and H5′), 4.14 (t, *J* = 9.4 Hz, 2H, H-3 and H3′), 4.12 – 4.10 (m, 2H, H-4 and H-4′), 3.83 (s, 6H), 3.69 – 3.59 (m, 6H). HRMS (ESI-TOF) *m/z*: $[M + H]^+$ calcd for $C_{56}H_{59}O_{13}$, 939.3950; Found, 939.3933.

2,3,2′,3′-Tetra-*O*-benzyl-6,6′-di-*O*-(*p*-methoxybenzylidene)-*α*,*α*-*D*-trehalose (11)

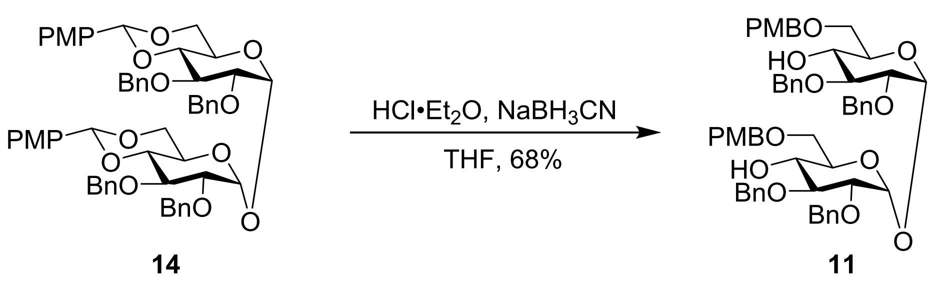

To a mixture of compound **14** (2.0 g, 2.13 mmol), $NaBH_3CN$（2.68 g, 42.6 mmol）and a small number of methyl orange indicator in THF (45 mL) was added a solution of HCl•Et_2O with a drop wise manner at 0 °C under N_2 atmosphere. The reaction mixture was stirred for 6 h, quenched by ice water, and extracted with ethyl acetate. The organic layers were combined, dried with anhydrous Na_2SO_4, and concentrated in vacuo. The crude product was purified by silica gel column chromatography (PE/ES = 5 : 1) to afford compound **11** (1.3 g, 68%). ^{1}H NMR (400 MHz, $CDCl_3$) δ 7.37 – 7.16 (m, 24H), 6.82 (d, *J* = 6.4 Hz, 4H), 5.20 (d, *J* = 3.6 Hz, 2H), 5.00 (d, *J* = 11.2 Hz, 2H), 4.80 (d, *J* = 11.2 Hz, 2H), 4.70 - 4.60 (q, *J* = 12.0 Hz, 4H), 4.40 (q, *J* = 12.0 Hz, 4H) , 4.11 (t, *J* = 3.6 Hz, 1H), 4.09 (t, *J* = 3.6 Hz, 1H), 3.87 (t, *J* = 9.2 Hz, 2H), 3.77 (s, 6H), 3.64 (t, *J* = 9.2 Hz, 2H)，3.53 (dd, *J* = 9.6, 3.6 Hz, 2H), 3.51 – 3.43 (qd, *J* = 10.4, 4.0 Hz, 4H). ^{13}C NMR (100 MHz, $CDCl_3$) δ 159.23, 138.85, 138.02, 130.05, 129.40, 128.55, 128.42, 128.01, 127.78, 127.69, 127.60, 113.78, 94.01, 81.05, 78.94, 75.29, 73.30, 72.54, 71.00, 70.68, 69.06, 55.29. HRMS (ESI-TOF) *m/z*: [M + COOH]$^-$ calcd for $C_{57}H_{63}O_{15}$, 987.4172; Found, 987.4177.

2,3,2′,3′-Tetra-*O*-benzyl-4-*O*-tert-butyldimethylsilyl-6,6′-di-*O*-(*p*-methoxybenzylidene)-*α*,*α*-*D*-trehalose (15)

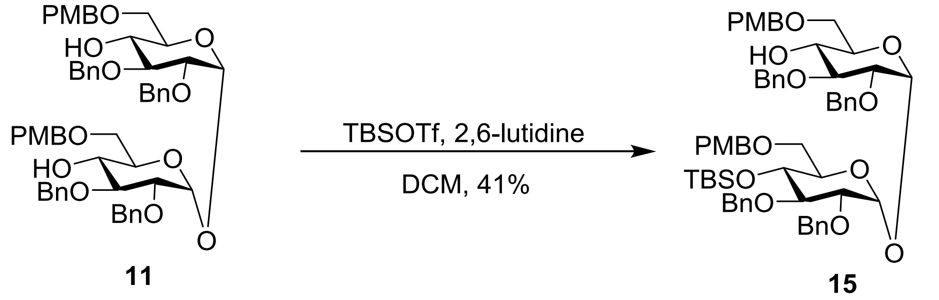

To a solution of compound **11** (270 mg, 0.29 mmol) in CH_2Cl_2 (5 mL) was added 2,6-lutidine (0.15 mL, 0.95 mmol) under nitrogen atmosphere. Then, TBSOTf (0.14 mL,

0.57 mmol) was added slowly at 0 °C. The reaction mixture was stirred at rt for 30 min, and quenched by saturated $NaHCO_3$ (30 ml). The mixture was extracted with CH_2Cl_2 (3 × 40 mL), and the organic layers were combined, dried over anhydrous Na_2SO_4, and concentrated in vacuo. The crude product was purified by silica gel column chromatography (PE/EA = 5:1) to afford compound **15** (123.8 mg, 41%). ^1H NMR (400 MHz, $CDCl_3$) δ 7.56 – 7.09 (m, 24H), 6.84 – 6.82 (d, J = 6.0 Hz, 4H), 5.32 (s, 1H), 5.26 (s, 1H), 5.09 (d, J = 11.6 Hz), 5.02 (d, J = 11.2 Hz, 1H), 4.85 (d, J = 11.2 Hz, 1H), 4.76 - 4.75 (m, 2H), 4.63 (d, J = 12.0 Hz, 2H), 4.54 (d, J = 12.0 Hz, 1H), 4.45 – 4.32 (m, 4H), 4.10 – 4.03 (m, 2H), 3.94 (t, J = 9.2 Hz, 1H), 3.85 –3.81 (m, 1H), 3.80 (s, 6H), 3.67 - 3.58 (m, 4H), 3.51 – 3.43 (m, 4H), 2.43 (s, 1H), 0.85 (s, 9H), -0.02 (s, 6H). ^{13}C NMR (100 MHz, $CDCl_3$) δ 159.26, 159.17, 139.28, 138.90, 138.25, 138.01, 130.28, 130.12, 129.42, 129.39, 129.33, 128.55, 128.49, 128.45, 128.40, 128.36, 128.14, 128.12, 127.80, 127.64, 127.59, 127.48, 127.04, 127.00, 113.81, 113.75, 93.43, 93.20, 81.22, 80.66, 79.92, 79.44, 75.06, 74.65, 73.32, 73.07, 72.60, 72.50, 72.21, 71.05, 70.87, 70.66, 69.17, 68.69, 55.30, 55.28, 26.18, 18.23, -3.52, -4.83. HRMS (ESI-TOF) *m/z*: $[M + Na]^+$ calcd for $C_{62}H_7O_{13}NaSi$, 1079.4947; Found, 1079.4951.

2,3,2′,3′-Tetra-*O*-benzyl-4-*O*-tert-butyldimethylsilyl-4′-*O*-(2-propyny)-6,6′-di-*O*-(*p*-methoxybenzylidene)-*α*,*α*-*D*-trehalose (16)

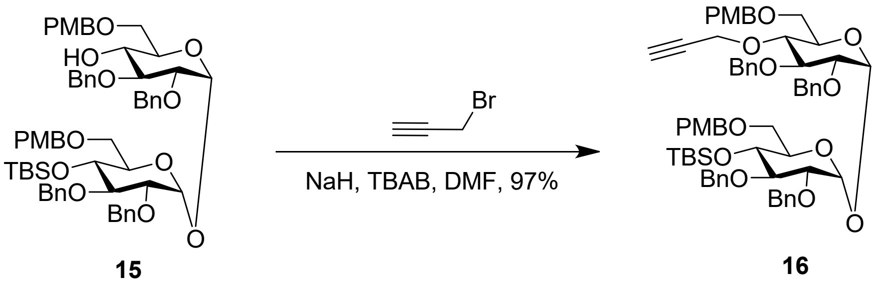

A mixture of **15** (1.0 g, 0.93 mmol) and 4 Å molecular sieves in anhydrous DMF was stirred at rt for 2 h. Then, NaH (60% in oil, 112 mg, 2.8 mmol) was added at 0 °C under nitrogen atmosphere. After reaction for 15 minutes, propargyl bromide (146 mL, 1.86 mmol) and TBAB were added and stirred at rt for 4 h. The reaction was quenched by MeOH, and diluted with CH_2Cl_2. Then, the mixture was washed with saturated $NaHCO_3$, brine, and filtered. The filter cake was purified by silica gel

column chromatography using PE/EA (6:1, v/v) as eluent to give the desired product (987 mg, 97% yield). ^{1}H NMR (400 MHz, $CDCl_3$) δ 7.40 – 7.27 (m, 20H), 7.21 – 7.18 (m, 4H), 6.84 – 6.81 (m, 4H), 5.26 (dd, *J* = 22.4 Hz, 2.8 Hz, 2H), 5.12 (d, *J* = 11.6 Hz, 1H), 4.18 (d, *J* = 11.6 Hz, 2H), 4.71 - 4.81 (m, 2H), 4.65 (d, *J* = 8.0 Hz, 1H), 4.61 (d, *J* = 8.0 Hz, 1H), 4.55 (d, *J* = 12.0 Hz, 1H), 4.50 – 4.30 (m, 5H), 4.21 – 4.02 (m, 4H), 3.80 - 3.79 (m, 7H), 3.69 (t, *J* = 9.6 Hz, 1H), 3.61 - 3.48 (m, 4H), 3.46 – 3.37 (m, 3H), 2.43 (s, 1H), 0.85 (s, 9H), 0.01 (dd, *J* = 3.2 Hz, 6H). ^{13}C NMR (100 MHz, $CDCl_3$) δ 164.14, 164.01, 144.21, 143.64, 143.15, 142.91, 135.14, 134.97, 134.45, 134.19, 133.33, 133.31, 133.24, 133.12, 133.00, 132.58, 132.50, 132.43, 132.34, 131.90, 131.84, 118.67, 118.59, 98.57, 98.51, 86.13, 86.07, 85.10, 84.74, 84.50, 82.40, 80.41, 79.54, 79.11, 78.03, 77.89, 77.60, 77.39, 77.01, 75.67, 75.20, 73.45, 72.86, 64.94, 60.21, 60.20, 31.03, 23.09, 1.32, 0.00. HRMS (ESI-TOF) *m/z*: $[M + Na]^+$ calcd for $C_{65}H_{78}O_{13}NaSi$, 1117.5104; Found,1117.5107.

2,3,2′,3′-Tetra-*O*-benzyl-4-*O*-tert-butyldimethylsilyl-4′-*O*-(2-propyny)-*α*,*α*-*D*-trehalose (17)

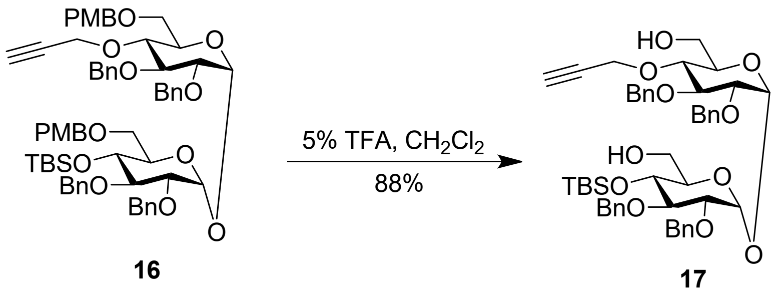

Compound **16** (1.0 g, 0.91 mmol) was dissolved in CH_2Cl_2 with 5% TFA (5 mL) at 0 °C, and stirred for 1 h at rt. The reaction was quenched by saturated $NaHCO_3$, and diluted with EA. Then, the organic layer was washed with brine, dried over anhydrous Na_2SO_4 and concentrated in vacuum. The residue was purified by silica gel column chromatography using PE/EA (2:1, v/v) as eluent to give the desired product (684 mg, 88% yield). ^{1}H NMR (400 MHz, $CDCl_3$) δ 7.32 – 7.12 (m, 20H), 5.19 (d, *J* = 2.8 Hz, 1H), 5.14 (d, *J* = 2.8 Hz, 2H), 5.16 (d, *J* = 11.6 Hz, 1H), 5.00 (d, *J* = 11.6 Hz, 1H), 4.87 (d, *J* = 10.8 Hz, 1H), 4.78 (d, *J* = 10.8 Hz, 1H), 4.73 – 4.66 (m, 2H), 4.63 – 4.57 (m, 2H), 4.43 – 4.39 (m, 2H), 4.07 (t, *J* = 9.2 Hz, 1H), 4.01 – 3.87 (m, 2H), 3.83 -

3.76 (m, 1H), 3.71 - 3.47 (m, 8H) , 2.47 (s, 1H), 0.87 (s, 9H), 0.01 (d, J = 24.0 Hz, 6H). ^{13}C NMR (100 MHz, $CDCl_3$) δ 139.09, 138.53, 137.97, 137.86, 128.52, 128.48, 128.46, 128.17, 128.15, 127.79, 127.77, 127.63, 127.43, 127.08, 93.55, 81.16, 81.08, 80.32, 79.97, 79.85, 76.72, 75.52, 74.91, 74.54, 73.13, 72.93, 72.85, 71.13, 70.38, 61.38, 59.97, 26.08, 18.17, -3.66, -4.88. HRMS (ESI-TOF) *m/z*: [M + COOH]$^-$ calcd for $C_{49}H_{63}O_{13}Si$, 899.4043; Found, 899.4034.

Compound 18

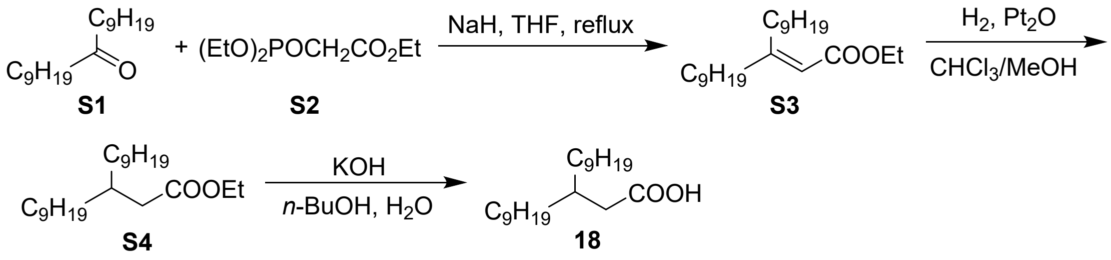

The synthesis of compound **18** was according to published procedure.[2] Some typical experimental details are as follows.

a) The synthesis of compound **S3**. To a solution of compound S2 and NaH in anhydrous THF was stirred at 0 °C for 30 min. Then compound S1 was added, and reacted at 70 °C for 18 h. Afterwards, the reaction was quenched by H_2O, and extracted with DCM. The organic layers was combined, dried over anhydrous Na_2SO_4 and concentrated in vacuum. The residue was purified by silica gel column chromatography using PE/DCM (3:1, v/v) as eluent to give the desired product. ^{1}H NMR (400 MHz, $CDCl_3$) δ 5.61 (s, 1H), 4.14 (q, J = 7.2 Hz, 2H), 2.58 (t, J = 8.1 Hz, 2H), 2.12 (t, J = 7.8 Hz, 2H), 1.44 (m, 4H), 1.27 (s, 27H), 0.88 (t, J = 6.9 Hz, 6H).

b) The synthesis of compound **S4**. To a solution of compound S3 and PtO_2 in $CHCl_3$/MeOH (5:1) was stirred at rt under H_2 atmosphere for 23 h. Then the reaction mixture was diluted with DCM, filtrated, and the organic layers were concentrated in vacuum. The residue was purified by silica gel column chromatography using PE/DCM (6:1, v/v) as eluent to give the desired product. Colourless oil, yield: 92%. ^{1}H NMR (400 MHz, $CDCl_3$) δ 4.12 (q, J = 7.2 Hz, 2H), 2.21 (d, J = 6.6 Hz, 2H), 1.86 – 1.82 (m, 1H), 1.27 – 1.26 (m, 35H), 0.88 (t, J = 6.6 Hz, 6H).

c) The synthesis of compound **18**. To a solution of compound S4 (795 mg, 2.25 mmol), KOH (1.26g, 22.5 mmol) in *n*-BuOH/H_2O (2:1) was stirred at 100 °C for 6 h. Then, the reaction was quenched by 1 N HCl, and extracted with EA. The organic layer was dried over anhydrous Na_2SO_4, and concentrated in vacuum. The residue was purified by silica gel column chromatography using PE/DCM (3:1, v/v) as eluent to give the desired product. ^1H NMR (400 MHz, $CDCl_3$) δ 2.27 (d, *J* = 6.9 Hz, 2H), 1.84 (s, 1H), 1.26 (s, 33H), 0.88 (t, *J* = 6.8 Hz, 6H).

2,3,2′,3′-Tetra-*O*-benzyl-4-*O*-tert-butyldimethylsilyl-4′-*O*-(2-propyny)-6,6′-Bis-*O*-(3-nonyldodecanoyl)-*α*,*α*′-trehalose (7)

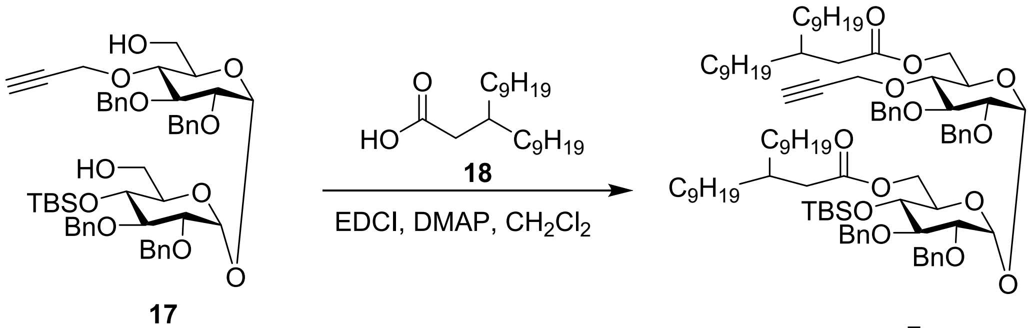

EDCI (731 mg, 2.46 mmol) was added to a mixture of **17** (427 mg, 0.5 mmol), **18** (447 mg, 1.37 mmol) and DMAP (6.1 mg, 0.05 mmol) in anhydrous DCM (5 mL) at 0 °C. Then, the mixture was stirred for 12 h at 50 °C. The resulting solution was diluted with DCM, washed with saturated $NaHCO_3$. The organic layer was dried over anhydrous Na_2SO_4 and concentrated in vacuum. The crud product was purified by silica gel column chromatography using PE/EA (10:1, v/v) as eluent to give the desired product **7** (647 mg, 88% yield). ^1H NMR (400 MHz, $CDCl_3$) δ 7.37 − 7.18 (m, 20H), 5.22 (s, 1H), 5.17 (s, 1H), 5.08 (d, *J* = 11.6 Hz, 1H), 4.98 (d, *J* = 11.6 Hz, 1H), 4.87 (d, *J* = 10.4 Hz 1H), 4.77 - 4.63 (m, 4H), 4.56 (d, *J* = 11.6 Hz, 1H), 4.44 (d, *J* = 11.6 Hz, 1H), 4.42 (d, *J* = 15.2 Hz, 1H),4.30 (d, *J* = 15.2 Hz, 1H), 4.18 - 4.04 (m, 6H), 3.89 (dd, *J* = 12.0 Hz, 3.6 Hz, 1H), 3.79 (t, *J* = 8.8 Hz, 1H), 3.63 (t, *J* = 9.6 Hz, 1H), 3.54 (t, *J* = 8.8 Hz, 2H), 3.46 (t, *J* = 9.2 Hz, 1H), 2.44 (s, 1H), 2.17 (d, *J* = 6.0 Hz,4H), 1.78 (s, 2H), 1.24 (s, 64H), 0.89 - 0.85 (m, 21H), 0.00 (s, 6H). ^{13}C NMR (100 MHz, $CDCl_3$) δ 173.25, 173.23, 139.03, 138.41, 137.76, 137.60, 128.55, 128.46, 128.19,

128.10, 127.85, 127.81, 127.78, 127.59, 127.34, 127.03, 127.01, 93.31, 81.06, 80.87, 79.98, 79.80, 79.72, 75.57, 74.76, 74.61, 73.10, 72.75, 70.64, 70.60, 68.81, 62.65, 60.42, 60.08, 39.08, 38.98, 34.87, 34.74, 33.79, 33.71, 33.67, 31.93, 29.95, 29.68, 29.65, 29.38, 26.56, 26.54, 26.51, 26.03, 22.71, 18.14, 14.14, -3.64, -4.99. HRMS (ESI-TOF) *m/z*: $[M + Na]^+$ calcd for $C_{91}H_{142}O_{13}NaSi$, 1494.0112; Found, 1494.0111.

2,3,2′,3′-Tetra-*O*-benzyl-4-*O*-tert-butyldimethylsilyl-4′-*O*-(2-propyny)-6,6′-Bis-*O*-(1-docosylmethyl)-*α*,*α*′-trehalose (8)

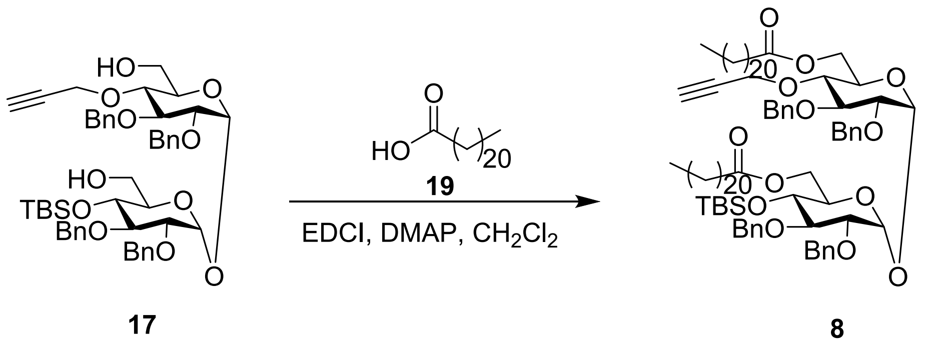

The synthesis of compound **8** was similar to **7** just using compound **19** instead of **18** (667 mg, 89%). ^{1}H NMR (400 MHz, $CDCl_3$) δ 7.36 − 7.21 (m, 20H), 5.20 (d, *J* = 12.0 Hz, 2H), 5.09 (d, *J* = 11.6 Hz, 1H), 4.99 (d, *J* = 11.6 Hz, 1H), 4.87 (d, *J* = 10.4 Hz 1H), 4.75 - 4.56 (m, 5H), 4.44 - 4.20 (m, 2H), 4.10 – 4.06 (m, 6H), 3.93 - 3.76 (m, 2H), 3.66 - 3.45 (m, 4H), 2.43 (d, *J* = 4.0 Hz, 1H), 2.24 – 2.22 (m, 4H), 1.62 (s, 4H), 1.25(m, 72H), 0.88 - 0.85 (m, 15H), -0.07 (s, 6H). ^{13}C NMR (100 MHz, $CDCl_3$) δ 173.52, 173.47, 138.99, 138.37, 137.75, 137.61, 128.57, 128.47, 128.20, 128.11, 127.87, 127.82, 127.81, 127.58, 127.32, 127.03, 93.38, 81.10, 80.89, 79.89, 79.73, 75.58, 74.80, 74.59, 73.09, 72.74, 70.60, 70.57, 68.82, 62.79, 60.04, 34.15, 34.11, 31.94, 29.72, 29.68, 29.65, 29.50, 29.38, 29.29, 29.18, 29.15, 26.01, 24.87, 24.79, 22.71, 18.13, 14.14, 1.03, -3.62, -5.02. HRMS (ESI-TOF) *m/z*: $[M + Na]^+$ calcd for $C_{93}H_{146}NaO_{13}Si$,1522.0425; Found, 1522.0420.

2,3,2′,3′-Tetra-*O*-benzyl-4′-*O*-(2-propyny)-6,6′-Bis-*O*-(3-nonyldodecanoyl)-*α*,*α*′-trehalose (20)

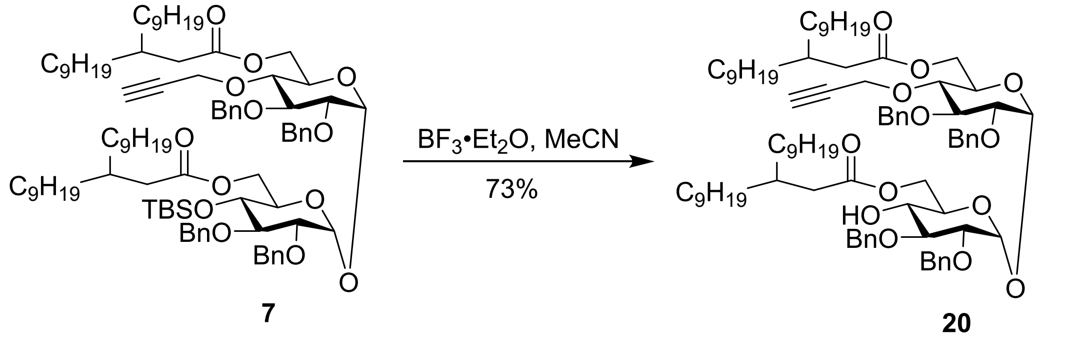

To a solution of compound **7** (1.0 g, 0.93 mmol) in anhydrous acetonitrile was added BF_3•Et_2O (1.1 eq) with a drop wise manner at 0 °C. After stirring for 2 h, the reaction was quenched by saturated $NaHCO_3$, and diluted with CH_2Cl_2. The organic layer was dried over anhydrous Na_2SO_4 and concentrated in vacuum. The crud product was purified by silica gel column chromatography using PE/EA (5:1, v/v) as eluent to give the desired product (921 mg, 73% yield). ^{1}H NMR (400 MHz, $CDCl_3$) δ 7.36 − 7.30 (m, 20H), 5.17 (d, *J* = 3.6 Hz, 2H), 5.01 - 4.97 (m, 2H), 4.87 - 4.83 (m, 2H), 4.75 - 4.56 (m, 4H), 4.45 - 4.41 (m, 1H), 4.32 - 4.28 (m, 2H), 4.23 - 4.11 (m, 4H), 4.02 (t, *J* = 16.0 Hz, 2H), 3.89 - 3.85 (m, 2H), 3.54 - 3.40 (m, 4H), 2.45 (s, 1H), 2.21 - 2.18 (m, 4H), 1.80 (m, 2H), 1.25 (s, 64H), 0.88 (t, *J* = 4.0 Hz, 12H). ^{13}C NMR (100 MHz, $CDCl_3$) δ 174.23, 173.27, 138.66, 138.39, 137.75, 137.73, 128.55, 128.50, 128.10, 128.05, 127.93, 127.83, 127.80, 127.61, 127.45, 94.37, 94.04, 81.40, 80.61, 79.68, 79.40, 78.91, 75.76, 75.53, 74.66, 73.03, 72.87, 70.16, 69.92, 68.93, 62.59, 60.14, 39.10, 39.04, 34.94, 34.88, 33.77, 33.71, 31.93, 29.94, 29.67, 29.65, 29.38, 26.57, 26.52, 22.71, 14.15. HRMS (ESI-TOF) *m/z*: $[M + H]^+$ calcd for $C_{85}H_{128}NaO_{13}$, 1379.9247; Found, 1379.9206.

2,3,2′,3′-Tetra-*O*-benzyl-4′-*O*-(2-propyny)-6,6′-Bis-*O*-(1-docosylmethyl)-*α*,*α*′-trehalose (21)

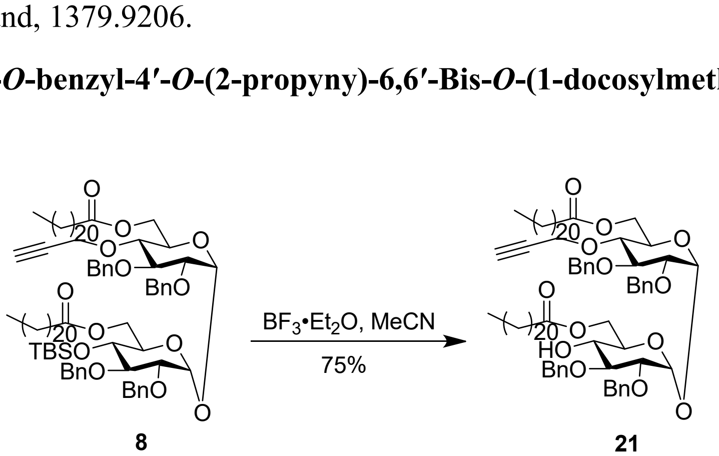

The synthesis of compound **21** was similar to **20** just using compound **8** instead of **7**

(966 mg, 75%). ^{1}H NMR (400 MHz, $CDCl_3$) δ 7.36 − 7.21 (m, 20H), 5.17 (d, *J* = 3.6 Hz, 2H), 5.01 - 4.97 (m, 2H), 4.87 – 4.83 (m, 2H), 4.73 - 4.66 (m, 4H), 4.43 (dd, *J* = 12.0, 2.4 Hz, 1H), 4.34 - 4.28 (m, 2H), 4.23 - 4.11 (m, 4H), 4.02 (t, *J* = 8.0 Hz, 1H), 3.90 - 3.85 (m, 2H), 3.56 - 3.40 (m, 4H), 2.43 (t, *J* = 2.4 Hz, 1H), 2.29 - 2.24 (m, 4H), 1.58 - 1.56 (m, 4H), 1.25 - 1.2 (m, 72H), 0.88 (t, *J* = 4.0 Hz, 6H). HRMS (ESI-TOF) *m/z*: $[M + Na]^+$ calcd for $C_{87}H_{132}O_{13}$, 1407.9578; Found, 1407.9567.

2,3,2′,3′-Tetra-*O*-benzyl-4′-*O*-propyl-6,6′-Bis-*O*-(1-docosylmethyl)-*α*,*α*′-trehalose (S14) used as the capture reagent (S5)

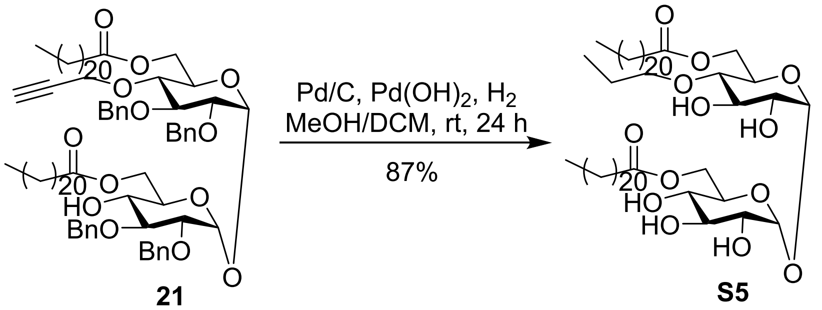

To a solution of compound **21** (20 mg) in MeOH and DCM was added Pd/C (10.5 mg) and $Pd(OH)_2$ (11 mg). The mixture was stirred at rt for 24 h under hydrogen atmosphere. Afterwards, the mixture was filtrated through a celite pad, and the filtrate was concentrated in vacuum to give the desired product. ^{1}H NMR (400 MHz, MeOH-*d*$_4$) δ 5.09 (dd, *J* = 3.7, 1.4 Hz, 2H), 4.38 – 4.24 (m, 4H), 3.97 – 3.75 (m, 2H), 3.91 – 3.81 (m, 2H), 3.78 – 3.74 (m, 1H), 3.59 – 3.45 (m, 3H), 3.36 (q, *J* = 1.6 Hz, 2H), 3.29 – 3.14 (m, 1H), 2.35 (td, *J* = 7.6, 4.8 Hz,4H), 1.60 (dq, *J* = 17.4, 7.0 Hz, 6H), 1.27 (s, 76H), 0.90 (dt, *J* = 9.2, 7.1 Hz, 9H).

Compound 23

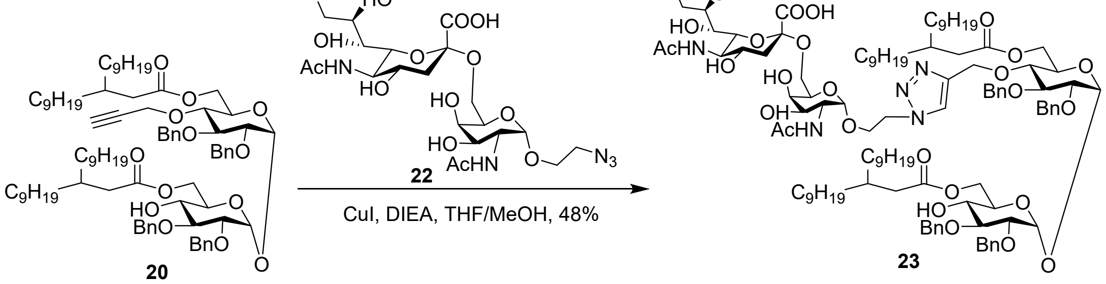

To a mixture of **20** (40 mg, 29 μmol), **22** (17.9 mg, 30.8 μmol), and CuI (53.3 mg, 0.28 mmol) in THF (2 mL) and MeOH (2 mL) was added DIEA (50 uL, 0.28 mmol).

The reaction was stirred at rt for 24 h, filtrated and concentrated. The residue was purified by silica gel column chromatography using MeOH/DCM (1 : 20, v/v) as eluent to give the desired product as a white solid (27.4 mg, 48%). ^{1}H NMR (600 MHz, $CD_3OD/CDCl_3$) δ 7.55 (s, 1H), 7.40 - 7.24 (m, 20H), 5.21 - 3.44 (m, 37H, 8H of Ar-CH_2, 29H of sugar and linker), 2.76 - 2.72 (m, 1H), 2.1 (s, 4H), 2.05 (m, 6H, -NHAc), 1.84 - 1.77 (m, 4H), 1.62 – 1.58 (m, 1H), 1.46 – 1.43 (m, 4H), 1.26 (s, 64H, CH_2 of lipid), 0.89 (t, J = 9.6 Hz, 12H, CH_3 of lipid). ^{13}C NMR (150 MHz, $CD_3OD/CDCl_3$) δ 180.70, 178.54, 178.11, 177.62, 177.53, 177.17, 148.97, 142.50, 142.43, 141.72, 141.35, 132.40, 132.30, 132.28, 131.89, 131.83, 131.59, 131.48, 131.44, 103.97, 101.67, 97.75, 97.67, 97.38, 97.31, 85.06, 84.96, 83.40, 82.73, 81.91, 79.36, 77.05, 76.83, 74.19, 74.07, 72.98, 72.68, 72.39, 71.63, 70.74, 70.23, 69.95, 69.87, 68.52, 66.92, 66.29, 64.33, 58.25, 57.96, 56.59, 46.25, 46.18, 43.01, 42.94, 38.87, 38.79, 37.56, 35.75, 33.76, 33.48, 33.19, 30.32, 27.08, 26.51, 26.01, 22.19, 20.86, 17.86, 15.95, 14.78, 14.75. HRMS (ESI-TOF) *m/z*: [M + H]$^+$ calcd for $C_{106}H_{164}N_5O_{27}$, 1939.1608; Found, 1939.1629.

Compound 24

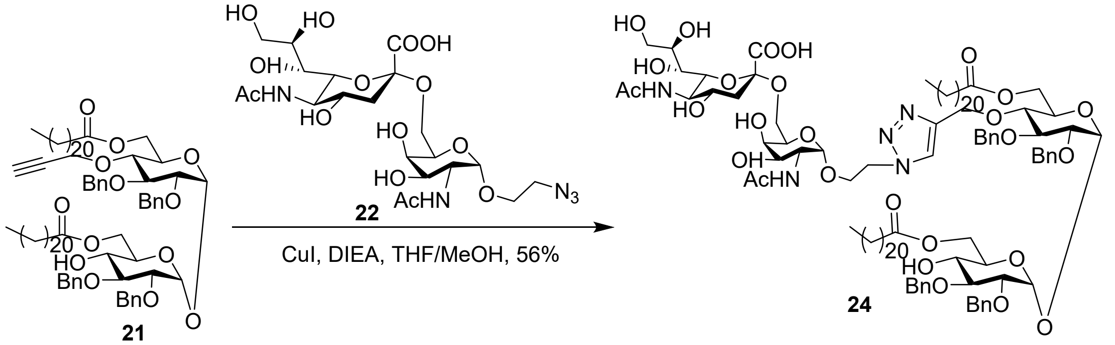

The synthesis of compound **24** was similar to **23** just using compound **21** instead of **20**. White solid, yield: 56%. ^{1}H NMR (600 MHz, $CD_3OD/CDCl_3$) δ 7.65 (s, 1H), 7.38 - 7.28 (m, 20H), 5.22 - 3.43 (m, 42H, 8H of Ar-CH_2, 34H of sugar and linker), 2.76 - 2.72 (m, 1H), 2.1 (s, 4H), 2.08 - 1.98 (m, 6H, -NHAc), 1.80 - 1.62 (m, 2H), 1.60 - 1.50 (m, 4H), 1.26 (s, 72H, CH_2 of lipid), 0.89 (t, J = 9.6 Hz, 6H, CH_3 of lipid). ^{13}C NMR (150 MHz, $CD_3OD/CDCl_3$) δ 176.83, 174.67, 174.47, 173.96, 173.92, 173.91, 173.90, 173.87, 138.58, 137.84, 137.50, 128.54, 128.44, 128.03, 127.76, 100.11,

97.84, 93.95, 93.60, 81.22, 81.10, 79.40, 78.78, 78.71, 77.98, 75.54, 75.49, 75.33, 74.89, 73.16, 72.91, 70.32, 70.12, 69.12, 66.01, 63.08, 62.54, 54.03, 42.28, 39.82, 34.13, 31.90, 29.67, 29.51, 29.44, 29.33, 29.24, 29.12, 24.88, 24.81, 23.22, 22.65, 14.00, 10.89. HRMS (ESI-TOF) *m/z*: $[M + H]^+$ calcd for $C_{108}H_{168}N_5O_{27}$, 1967.1921; Found, 1967.1908.

Compound 1

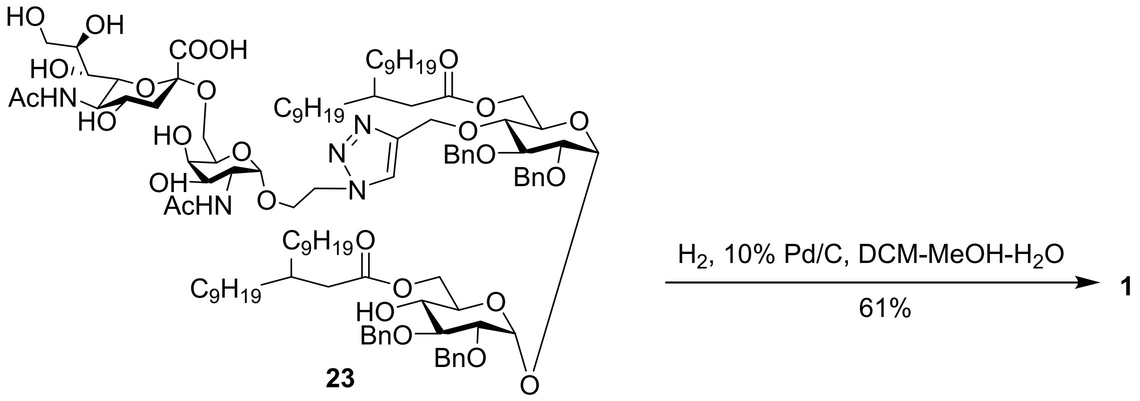

A mixture of **23** (30 mg, 15.5 μmol) and 10% Pd/C (30 mg) in DCM/MeOH/ H_2O (3:3:0.1, 20 mL) was stirred under hydrogen atmosphere at rt for 24 h. Then, the reaction mixture was diluted and filtrated through a celite pad. The filtrate was washed with water, and the organic layer was concentrated in vacuum to give **1** as a white solid (14.9 mg, 61% yield). ^1H NMR (600 MHz, $CD_3OD/CDCl_3$) δ 8.12 (s, 1H), 5.10 - 2.67 (m, 27H, 27H of sugar and linker), 2.32 - 2.28 (m, 4H), 2.32 – 2.28 (m, 4H), 2.05 (s, 6H, -NHAc), 1.85 (s, 2H), 1.63 – 1.60 (m, 1H), 1.46 – 1.40 (m, 4H), 1.26 (s, 64H, CH_2 of lipid), 0.89 (t, J = 9.6 Hz, 12H, CH_3 of lipid). HRMS (ESI-TOF) *m/z*: $[M + H]^+$ calcd for $C_{78}H_{140}N_5O_{27}$, 1578.9730; Found, 1578.9726.

Compound 2

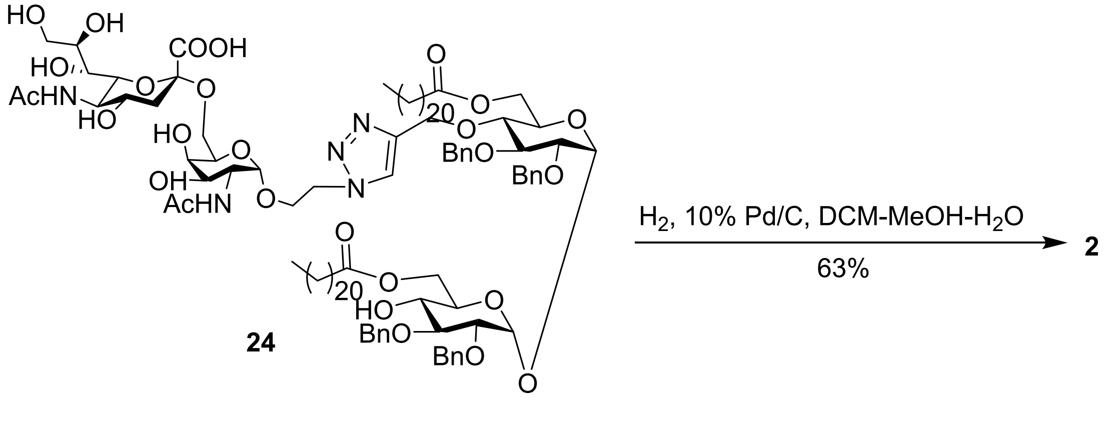

The synthesis of compound **2** was similar to **1** just using compound **24** instead of **23**. White solid, yield: 63%. ^{1}H NMR (600 MHz, $CD_3OD/CDCl_3$) δ 8.12 (s, 1H), 5.11 - 3.43 (m, 35H, 35H of sugar and linker), 2.72 - 2.70 (m, 1H), 2.41 - 2.38 (m, 4H), 2.07 (s, 6H, -NHAc), 1.80 - 1.78 (m, 1H), 1.60 - 1.62 (m, 4H), 1.26 (s, 72H, CH_2 of lipid), 0.89 (t, J = 9.6 Hz, 6H, CH_3 of lipid). HRMS (ESI-TOF) *m/z*: $[M + H]^+$ calcd for $C_{80}H_{144}N_5O_{27}$, 1607.0043; Found, 1607.0044.

III. Preparation and Characterization of STn

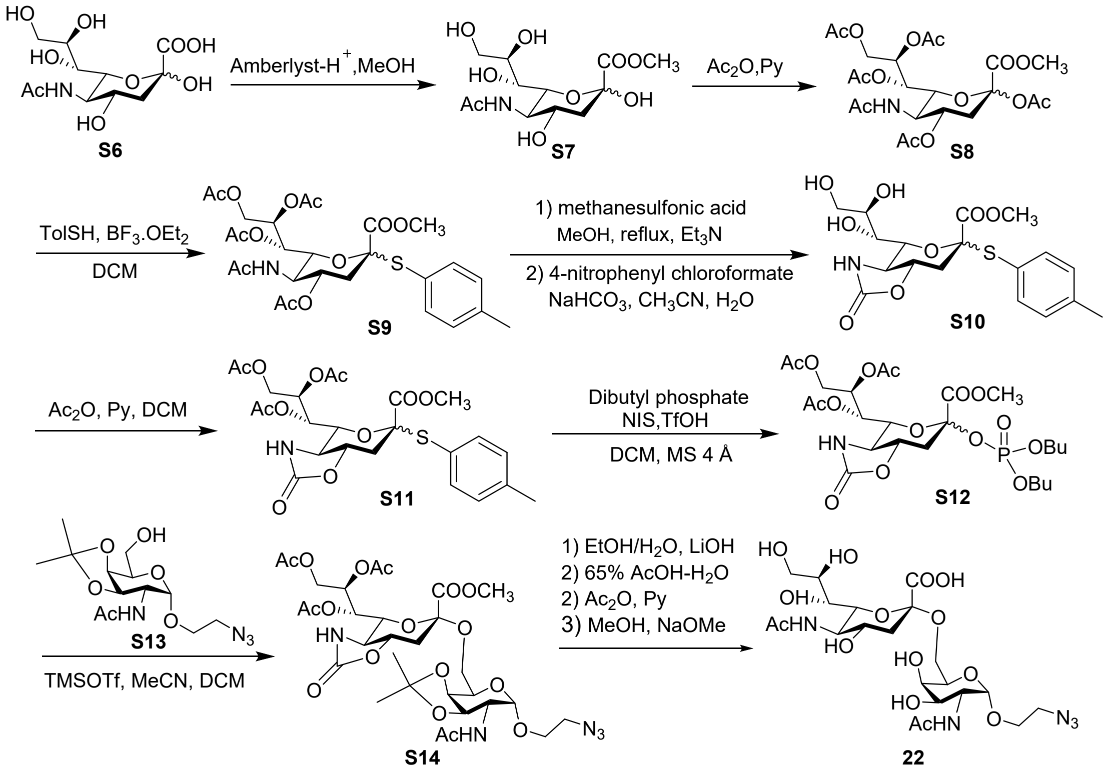

The synthesis of STn was similar to the published processes.[3,4] Some typical experimental details are as follows.

a) The synthesis of compound **S10**. To a solution of compound **S9** (5.51 g) in MeOH was added PTSA and stirred at 0 °C for 15 min. Afterwards, the mixture was reacted at 90 °C for 4 h. The reaction was quenched by Et_3N, and the mixture was concentrated in vacuum. Then, to a solution of above product, $NaHCO_3$ in $MeCN/H_2O$ was added *p*-nitrophenyl chloroformate, and stirred for 3 h. The reaction mixture was diluted with EA, and washed with H_2O. The organic layer was dried over

anhydrous Na_2SO_4 and concentrated in vacuum. The crud product was purified by silica gel column chromatography using DCM/MeOH (40:1, v/v) as eluent to give the desired product. Yellow solid, yield: 55.2%. ^{1}H NMR (400 MHz, $CDCl_3$) δ 7.47 (d, *J* = 9.2 Hz, 2H), 7.18 (d, *J* = 9.2 Hz, 2H), 4.66 – 4.63 (m, 2H), 3.86 – 3.82 (m, 1H), 3.75 – 3.71 (m, 2H), 3.61 – 3.57 (m, 2H), 2.39 (t, *J* = 12 Hz, 1H), 2.37 – 2.33 (m, 3H).

b) The synthesis of compound **S11**. To a mixture of compound S10 (2.1 g) in anhydrous pyridine was added Ac_2O at 0 °C. After stirring at rt for 5 h, the mixture was concentrated. The crud product was purified by silica gel column chromatography using PE/EA/AcOH (2:1:0.01, v/v) as eluent to give the desired product. White solid, yield: 86%. ^{1}H NMR (400 MHz, $CDCl_3$) δ 7.29 (d, *J* = 8.0 Hz, 2H), 7.16 (d, *J* = 8.0 Hz, 2H), 5.50 (s, 1H), 5.23 – 5.17 (m, 2H), 4.71 (td, *J* = 12.4, 3.6 Hz, 1H), 4.59 (dd, *J* = 9.6, 2.4 Hz, 1H), 4.42 (dd, *J* = 12.8, 2.4 Hz, 1H), 4027 – 4.22 (m, 1H), 3.63 (s, 3H), 3.10 (t, *J* = 11.2 Hz, 1H), 2.81 (dd, *J* = 12.8, 4.0 Hz, 1H), 2.36 (s, 3H), 2.25 (t, *J* = 12.8 Hz, 1H), 2.16 (s, 3H), 2.10 (s, 3H).

c) The synthesis of compound **S12**. A mixture of S11, dibutyl phosphate and 4 Å MS in anhydrous DCM was stirred at rt under N_2 atmosphere for 2 h. Then, NIS and TfOH were added under 0 °C, and stirred for 6h. The reaction mixture was diluted with DCM, washed with saturated $NaHCO_3$. The organic layer was dried over anhydrous Na_2SO_4 and concentrated in vacuum. The crud product was purified by silica gel column chromatography using PE/EA (5:1, v/v) as eluent to give the desired product. White solid, yield: 66%. ^{1}H NMR (400 MHz, $CDCl_3$) δ 5.40 – 5.34 (m, 2H), 5.12 (d, *J* = 10.0 Hz, 1H), 4.46 (d, *J* = 9.2 Hz, 1H), 4.36 (d, *J* = 12.8 Hz, 1H), 4.29 (d, *J* = 12.8 Hz, 1H), 4.14 – 4.05 (m, 6H), 3.83 (d, *J* = 8.8 Hz, 3H), 3.23 (t, *J* = 10.4 Hz, 1H), 2.92 (d, *J* = 11.6 Hz, 1H), 2.65 (t, *J* = 12.8 Hz, 1H), 2.19 (s, 3H), 2.15 (s, 3H), 2.10 (s, 1H), 2.06 (s, 3H), 1.72 – 1.62 (m, 8H), 1.44 – 1.39 (m, 5H), 0.95 (t, *J* = 7.2 Hz, 6H).

d) Compound **S13** was synthesized according to the reported procedures.[4]

e) The synthesis of intermediate **S14**. A mixture of compound **S12** (1.0 g, 1.6 mmol), **S13** (0.44g, 1.3 mmol) and 4 Å MS in DCM/MeCN (20 mL, *v/v* = 2:1) was stirred 2 h

under N_2 atmosphere. Then, trimethylsilyl trifluoromethanesulfonate (0.31 g, 0.14 mmol) was added at -40 °C, and reacted for 2 h. After the reaction was accomplished, Et_3N was added to quench the reaction. The organic layer was concentrated in vacuum, and the crud product was purified by silica gel column chromatography using PE/EA as eluent to give the desired product. White solid, yield: 66%. ^{1}H NMR (400 MHz, $CDCl_3$) δ 5.74 (d, J = 9.4 Hz, 1H), 5.51 – 5.42 (m, 1H), 5.14 (dd, J = 9.6, 1.7 Hz, 1H), 4.82 (d, J = 3.3 Hz, 1H), 4.35 – 4.22 (m, 2H), 4.18 (dd, J = 4.8, 2.4 Hz, 1H), 4.08 (dt, J = 11.7, 4.5 Hz, 1H), 4.02 – 3.94 (m, 1H), 3.90 (dd, J = 9.8, 6.4 Hz, 1H), 3.81 (s, 2H), 3.64 (ddd, J = 10.3, 7.5, 5.0 Hz, 1H), 3.40 (dd, J = 7.2, 4.2 Hz, 1H), 3.09 (t, J = 10.5 Hz, 1H), 2.91 (dt, J = 7.4, 3.7 Hz, 1H), 2.20 (s, 2H), 2.18 (s, 2H), 2.12 (s, 1H), 2.06 (d, J = 4.7 Hz, 2H), 2.02 (s, 2H), 1.56 (s, 2H), 1.35 (s, 2H).

f) The synthesis of compound **22**. To a solution of compound S14 in EtOH/H_2O (1:1) was added LiOH under N_2 atmosphere. Then, it reacted at 80 °C for 48 h, and 10% HCl was added. The reaction mixture was concentrated in vacuum, and the residue was dissolve in 65% AcOH/H_2O. The mixture was stirred at 65 °C for 3 h, and concentrated in vacuum again. The residue was dissolved in pyridine, and acetic anhydride was added at 0 °C. After the mixture was stirred for 6 h, pyridine was removed and excess amounts of EA and saturated sodium bicarbonate solution was added. The organic layer was concentrated in vacuum. The residue was dissolved in MeOH, and MeONa was added until the pH is 10~11. The solution was stirred at rt for 6 h, and concentrated in vacuum. The crud product was purified by gel column chromatography. Yield: 67%. ^{1}H NMR (400 MHz, D_2O) δ 4.86 (d, J = 3.7 Hz, 1H), 4.07 (dd, J = 11.0, 3.7 Hz, 1H), 4.01 – 3.90 (m, 1H), 3.88 – 3.70 (m, 3H), 3.65 – 3.40 (m, 4H), 2.64 (dd, J = 12.4, 4.6 Hz, 1H), 1.95 (d, J = 3.2 Hz, 3H), 1.59 (t, J = 12.1 Hz, 1H). ^{13}C NMR (100 MHz, D_2O) δ 174.99, 174.64, 173.32, 100.38, 97.08, 72.54, 71.73, 69.56, 68.43, 68.22, 68.18, 67.36, 66.70, 63.75, 62.60, 51.81, 50.20, 49.76, 40.20, 22.01, 21.99.

IV. Preparation and Characterization of Conjugates 3 and 4

2,3,2′,3′-Tetra-*O*-benzyl-4,4′-di-*O*-(2-propyny)-6,6′-di-*O*-(*p*-methoxybenzylidene)-*α*,*α*′-*D*-trehalose (25)

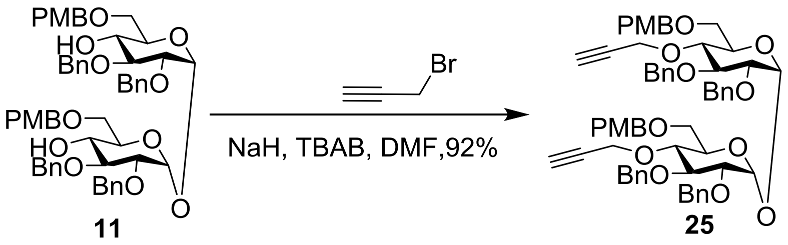

A mixture of **11** (1.0 g, 1.06 mmol) and 4 Å molecular sieves in anhydrous DMF was stirred at rt for 2 h. Then, NaH (60% in oil, 0.2 g, 5.3 mmol) was added at 0 °C under nitrogen atmosphere. After reaction for 15 minutes, propargyl bromide (0.5 mL, 6.36 mmol) and TBAB (0.7g, 2.12 mmol) were added and stirred at rt for 6 h. The reaction was quenched by MeOH, and diluted with CH_2Cl_2. Then, the mixture was washed with saturated $NaHCO_3$, brine, and filtered. The filter cake was purified by silica gel column chromatography using PE/EA (4:1, v/v) as eluent to give the desired product (1.08 g, 92% yield). ^{1}H NMR (400 MHz, $CDCl_3$) δ 7.40 – 7.27 (m, 20H) , 7.19 (d, *J* = 8.4 Hz, 4H), 6.83 (d, *J* = 8.4 Hz, 4H), 5.15 (d, *J* = 3.6 Hz, 2H), 4.98-4.94 (m, 2H), 4.85 (d, *J* = 8.0 Hz, 2H), 4.67 – 4.61 (m, 4H), 4.48–4.45 (d, *J* = 11.6 Hz, 4H), 4.39–4.33 (m, 4H), 4.16–4.11 (m, 2H), 3.98–3.96 (t, *J* = 9.2 Hz, 2H), 3.79 (s, 6H)， 3.53-3.48 (m, 6H), 3.40 – 3.37 (m, 2H), 2.41 (t, *J* = 2.4 Hz, 2H). ^{13}C NMR (100 MHz, $CDCl_3$) δ 159.22, 138.73, 138.11, 130.00, 129.53, 128.42, 128.36, 128.10, 127.63, 127.58, 127.52, 113.74, 94.62, 81.53, 80.13, 79.11, 77.49, 75.68, 74.19, 73.09, 72.68, 70.34, 67.86, 60.06, 55.28. HRMS (ESI-TOF) *m/z*: $[M + Na]^+$ calcd for $C_{62}H_{66}NaO_{13}$, 1041.4396; Found, 1041.4395.

2,3,2′,3′-Tetra-*O*-benzyl-4, 4′-di-*O*-(2-propyny)-*α*,*α*′-*D*-trehalose (26)

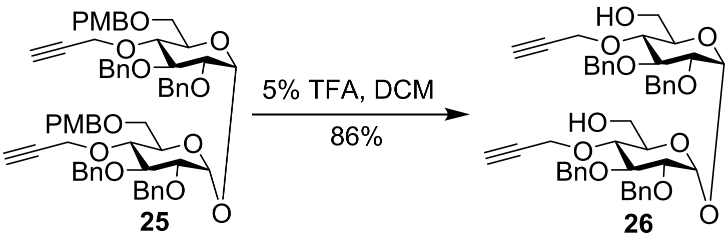

Compound **25** (1.5 g, 1.5 mmol) was dissolved in CH_2Cl_2 with 5% TFA (8 mL) at 0 °C, and stirred for 1 h at rt. The reaction was quenched by saturated $NaHCO_3$, and diluted with EA. Then, the organic layer was washed with brine, dried over anhydrous Na_2SO_4 and concentrated in vacuum. The residue was purified by silica gel column chromatography using PE/EA (2:1, v/v) as eluent to give the desired product (986 mg, 86% yield). ^1H NMR (400 MHz, $CDCl_3$) δ 7.39 – 7.25 (m, 20H), 5.11 (d, J = 3.6 Hz, 2H), 4.99 (d, J = 10.8 Hz, 2H), 4.85 (d, J = 10.8 Hz, 2H), 4.72 - 4.65 (m, 4H), 4.46 – 4.37 (m, 4H), 4.04 – 4.00 (m, 4H), 3.72 – 3.60 (m, 4H), 3.54 - 3.49 (m, 4H), 2.48 (t, J = 2.0 Hz, 2H). ^{13}C NMR (100 MHz, $CDCl_3$) δ 138.56, 137.92, 128.50, 128.48, 128.04, 127.83, 127.74, 127.60, 94.20, 81.47, 80.30, 79.38, 76.69, 75.66, 74.54, 73.03, 71.17, 61.33, 60.00. HRMS (ESI-TOF) *m/z*: $[M + COOH]^-$ calcd for $C_{47}H_{51}O_{13}$, 823.3335; Found, 823.3311.

2,3,2′,3′-Tetra-*O*-benzyl-4,4′-di-*O*-(2-propyny)-6,6′-Bis-*O*-(3-nonyldodecanoyl)-*α*,*α*′-trehalose (9)

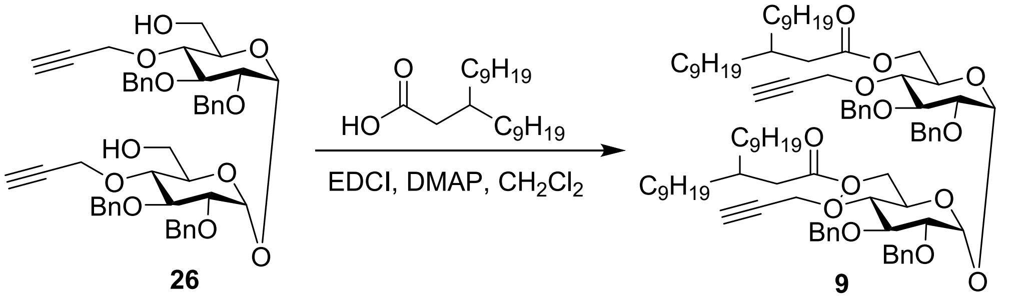

EDCI (37 mg, 1.93 mmol) was added to a mixture of **26** (500 mg, 0.64 mmol), **18** (628 mg, 1.93 mmol) and DMAP (235 mg, 1.93 mmol) in anhydrous DCM (5 mL) at 0 °C. Then, the mixture was stirred for 12 h at 50 °C. The resulting solution was diluted with DCM, washed with saturated $NaHCO_3$. The organic layer was dried over anhydrous Na_2SO_4 and concentrated in vacuum. The crud product was purified by silica gel column chromatography using PE/EA (10:1, v/v) as eluent to give the desired product **9** (759 mg, 85% yield). ^1H NMR (400 MHz, $CDCl_3$) δ 7.40 − 7.27 (m, 20H), 5.15 (d, J = 3.2 Hz, 2H), 4.99 (d, J = 10.8 Hz, 2H), 4.85 (d, J = 10.8 Hz, 2H), 4.72 - 4.65 (m, 4H), 4.44 (dd, J = 15.2, 2.4 Hz, 2H,), 4.30 (dd, J = 15.2, 2.4 Hz, 2H), 4.22 – 4.17 (m, 2H), 4.14 – 4.13 (m, 4H), 4.01 (t, J = 9.2 Hz, 2H), 3.52 (dd, J = 9.6,

3.2 Hz, 2H), 3.45 (t, J = 9.2 Hz, 2H), 2.44 (s, 2H), 1.67 (s, 2H), 2.19 (d, J = 6.8 Hz, 4H), 1.80 (s, 2H), 1.24 (s, 64H), 0.87 (t, J = 6.4 Hz, 12H). ^{13}C NMR (100 MHz, $CDCl_3$) δ 173.24, 138.45, 137.71, 128.55, 128.48, 128.11, 127.92, 127.77, 127.58, 94.02, 81.33, 79.70, 79.26, 75.75, 74.62, 72.95, 68.89, 62.59, 60.14, 39.08, 34.86, 33.78, 33.71, 31.93, 29.95, 29.67, 29.64, 29.37, 26.56, 26.51, 22.71, 14.15. HRMS (ESI-TOF) *m/z*: $[M + Na]^+$ calcd for $C_{88}H_{130}NaO_{13}$, 1417.9404; Found, 1417.9404.

2,3,2′,3′-Tetra-*O*-benzyl-4,4′-di-*O*-(2-propyny)-6,6′-Bis-*O*-(1-docosylmethyl)-*α*,*α*′-trehalose (10)

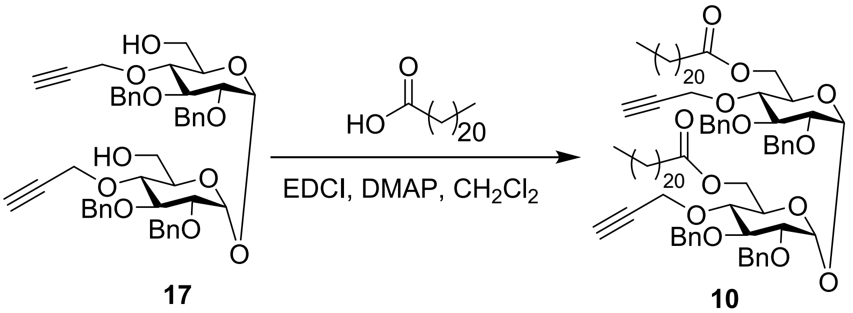

17 **10**

The synthesis of compound **10** is similar to compound **9**. Yield: 86%. ^{1}H NMR (400 MHz, $CDCl_3$) δ 7.37 − 7.25 (m, 20H), 5.15 (s, 2H), 4.99 (d, J = 10.8 Hz, 2H), 4.85 (d, J = 10.8 Hz, 2H), 4.69 (t, J = 12.0 Hz, 4H), 4.43 (d, J = 15.6 Hz, 2H), 4.31 (d, J = 15.6 Hz, 2H), 4.21 - 4.10 (m, 6H), 4.01 (t, J = 9.2 Hz, 2H), 3.54-3.44 (m, 4H), 2.44 (s, 2H), 2.25 (t, J = 7.6 Hz, 2H), 1.58 – 1.55 (m, 5H), 1.25 (s, 72H), 0.88 (t, J = 6.8 Hz, 6H). ^{13}C NMR (100 MHz, $CDCl_3$) δ 173.51, 138.41, 137.71, 128.57, 128.51, 128.13, 127.95, 127.81, 127.59, 94.04, 81.36, 79.72, 79.18, 75.76, 74.63, 72.95, 68.90, 62.73, 60.11, 34.16, 31.96, 29.74, 29.70, 29.65, 29.52, 29.40, 29.30, 29.19, 24.88, 22.73, 14.17. HRMS (ESI-TOF) *m/z*: $[M + Na]^+$ calcd for $C_{90}H_{134}NaO_{13}$, 1445.9710; Found, 1445.9730.

Compound 27

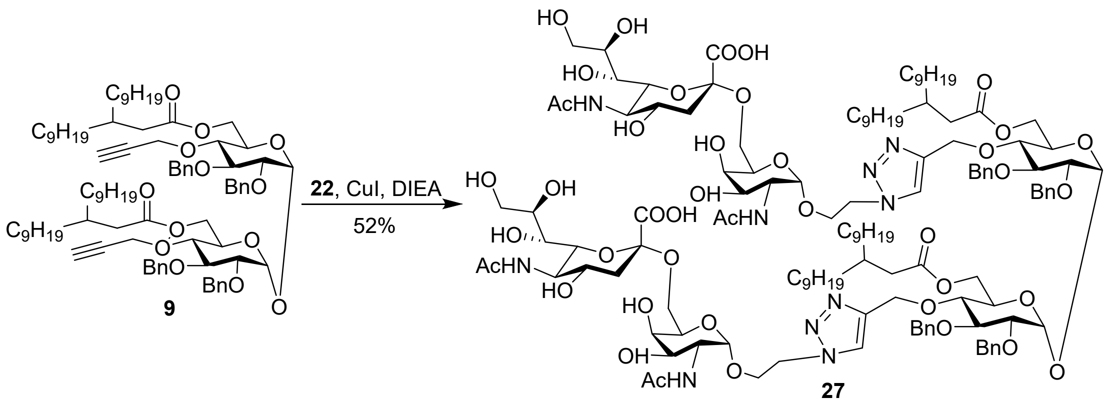

The synthesis of compound **27** was similar to **23**. White solid, yield: 52%. ^{1}H NMR (400 MHz, $CD_3OD/CDCl_3$) δ 7.63 (s, 2H), 7.38 - 7.24 (m, 20H), 5.19 - 3.49 (m, 64H, 8H of Ar-CH_2, 56H of sugar and linker), 2.1 (d, 4H), 1.96 - 2.07 (m, 12H, -NHAc), 1.76 - 1.90 (m, 4H), 1.26 (s, 64H, CH_2 of lipid), 0.89 (t, J = 9.6 Hz, 12H, CH_3 of lipid); HRMS (ESI-TOF) m/z: $[M + 2H]^{2+}$ calcd for $C_{130}H_{202}N_{10}O_{41}$, 1279.7009; Found, 1279.7037.

Compound 28

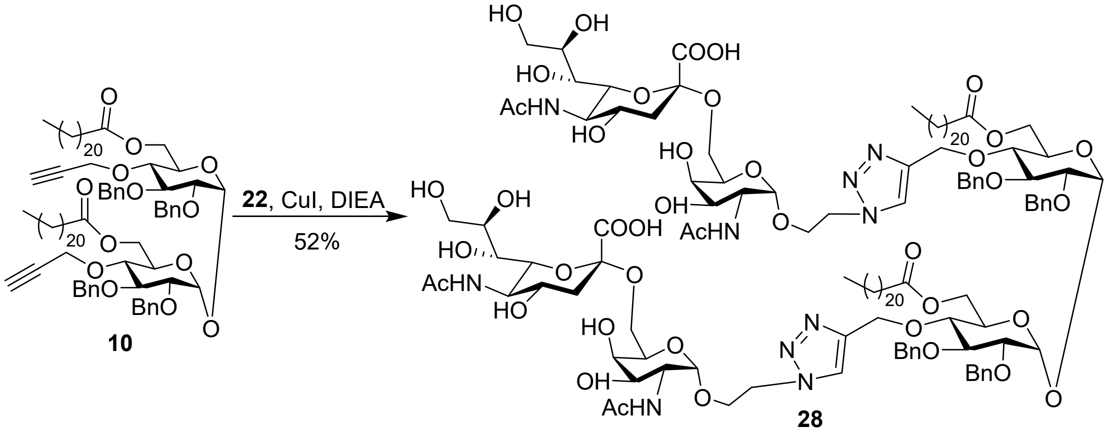

The synthesis of compound **28** was similar to **23**. White solid, yield: 52%. ^{1}H NMR (600 MHz, $CD_3OD/CDCl_3$) δ 7.74 (s, 1H), 7.67 (s, 1H), 7.38 - 7.24 (m, 20H), 5.19 - 3.49 (m, 62H, 8H of Ar-CH_2, 54H of sugar and linker), 2.56 - 2.52 (m, 2H), 2.1 (s, 4H), 2.08 - 1.98 (m, 12H, -NHAc), 1.80 - 1.62 (m, 2H), 1.60 - 1.50 (m, 4H), 1.26 (s, 72H, CH_2 of lipid), 0.89 (t, J = 9.6 Hz, 6H, CH_3 of lipid). ^{13}C NMR (150 MHz, $CD_3OD/CDCl_3$) δ 180.84, 180.65, 178.61, 178.05, 177.95, 149.37, 142.37, 142.17, 141.75, 141.56, 132.60, 132.26, 132.14, 131.64, 131.43, 131.23, 130.08, 104.04, 103.98, 102.13, 101.67, 100.05, 98.99, 85.51, 83.82, 83.02, 82.79, 79.61, 78.31, 77.17,

76.73, 76.41, 76.28, 73.97, 73.84, 73.69, 73.19, 73.05, 72.45, 72.34, 72.11, 71.39, 70.72, 69.66, 68.77, 68.45, 68.22, 66.52, 66.25, 64.29, 58.08, 53.67, 53.22, 52.97, 44.20, 43.69, 38.04, 37.98, 35.75, 33.49, 33.32, 33.29, 33.17, 33.08, 33.04, 32.93, 28.75, 27.17, 26.88, 26.48, 25.91, 17.69, 4.68, 3.44. HRMS (ESI-TOF) *m/z*: $[M + 2H]^{2+}$ calcd for $C_{132}H_{204}N_{10}O_{41}$, 1293.7165; Found, 1293.7167.

Compound 3

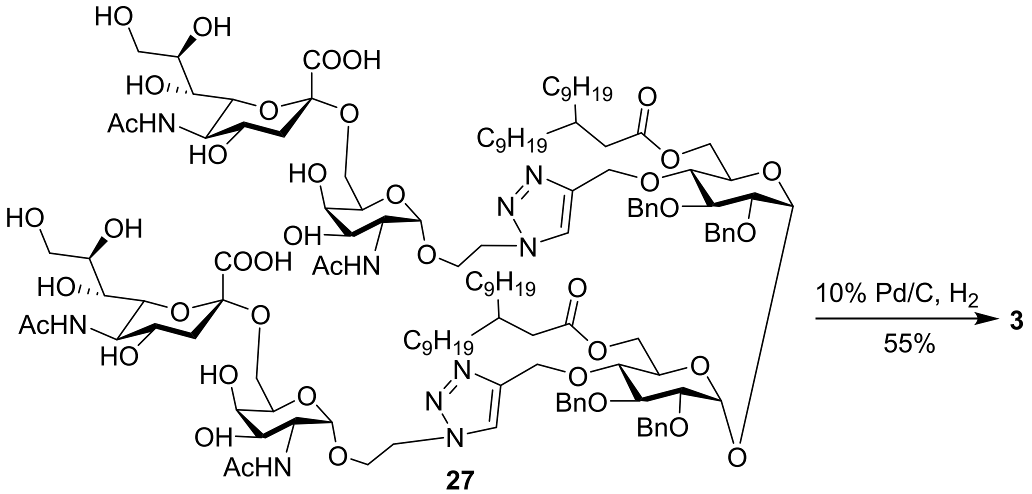

The synthesis of compound **3** was similar to **1**. White solid, yield: 55%. ^{1}H NMR (600 MHz, $CD_3OD/CDCl_3$) δ 7.63 (s, 2H), 5.19 - 3.49 (m, 54H of sugar and linker), 2.66 - 2.57 (m, 2H), 2.1 (s, 4H), 1.93 - 1.75 (m, 12H, -NHAc), 1.69 - 1.62 (m, 2H), 1.55 - 1.48 (m, 2H), 1.21 (s, 64H, CH_2 of lipid), 0.89 (t, *J* = 9.6 Hz, 12H, CH_3 of lipid). HRMS (ESI-TOF) *m/z*: $[M + Na]^+$ calcd for $C_{102}H_{176}N_{10}NaO_{41}$, 2220.1887; Found, 2220.1807.

Compound 4

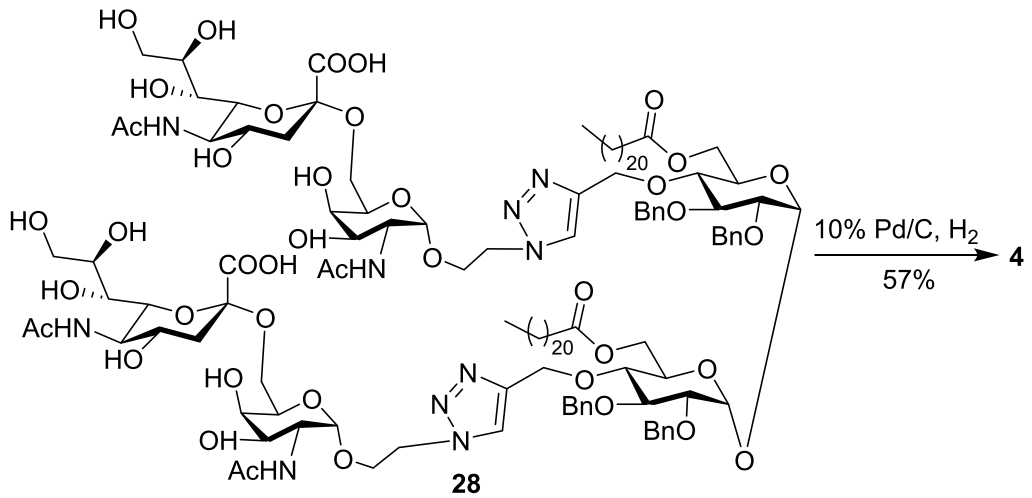

The synthesis of compound **4** was similar to **1**. White solid, yield: 57%. ^1H NMR (600 MHz, $CD_3OD/CDCl_3$) δ 8.09 (s, 2H), 5.10 - 3.00 (m, 54H of sugar and linker), 2.76 - 2.72 (m, 2H), 2.41 - 2.37 (t, 4H), 2.05 (s, 12H, -NHAc), 1.85-1.75 (m, 2H),1.66-1.62 (m, 4H), 1.26 (s, 72H, CH_2 of lipid), 0.89 (t, J = 9.6 Hz, 6H, CH_3 of lipid). HRMS (ESI-TOF) *m/z*: $[M+2H]^{2+}$ $C_{104}H_{182}N_{10}O_{41}$, 1113.6226; Found, 1113.6251.

V. Analysis of Sialic Acid Loading of STn-CRM197 and STn-HSA

The conjugate of STn-CRM197 was identified by SDS-PAGE (Fig. S1). The epitope ratio of STn-CRM197 was analyzed by Svennerholm method.[5,6] Accurately weighed samples of STn-CRM197 (0.51 mg) were dissolved in distilled water (0.51 mL), mixed well with resorcinol reagent (2.0 mL), and heated in boiling water for 30 min. The solutions were then cooled to rt and combined with an extraction solution (1-butanol acetate and 1-butanol, 85:15 v/v, 3.0 mL). The mixture was shaken vigorously and allowed to stand still for 10 min to separate the organic layer from inorganic layer. The upper organic layer was transferred to a 96-well plate, and absorbance at 580 nm was determined by a microplate analyzer, using the organic solvents as the blank control. The sialic acid content of the glycoconjugate was determined with a calibration curve created with standard Neu5Acyl solutions and analyzed under the same conditions. Sialic acid loading of the conjugates was calculated according to the following equation:

$$sialic\ acid\ loading\ (\%) = \frac{weight\ of\ sialic\ acid\ (mg) of\ the\ sample}{weight\ of\ the\ glycoconjugate\ sample\ (mg)} \times 100\%$$

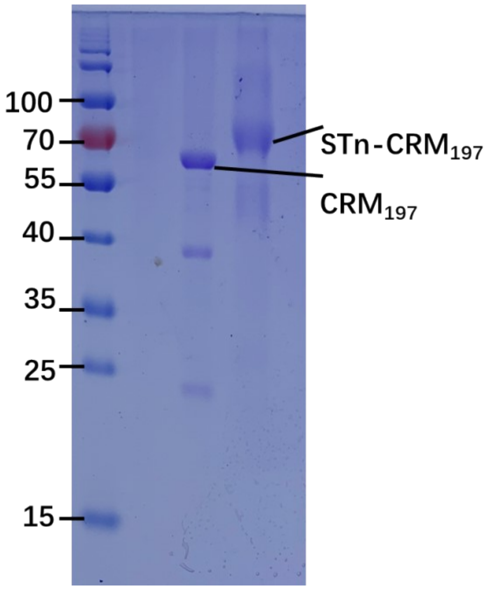

Fig. S1 STn-CRM197 characterization

Table S1 The absorbance of standard Neu5Acyl solutions in different concentrations

Sialic acid (μg)	1	2	3	Mean
10	0.2163	0.2143	0.2317	0.220767
20	0.3423	0.3222	0.3476	0.337367
30	0.4280	0.4357	0.4357	0.433133
40	0.4576	0.4634	0.4692	0.4634
50	0.6041	0.6142	0.5731	0.597133
60	0.6428	0.6616	0.6668	0.657067
70	0.7018	0.7782	0.7941	0.758033
80	0.8328	0.8750	0.8579	0.855233

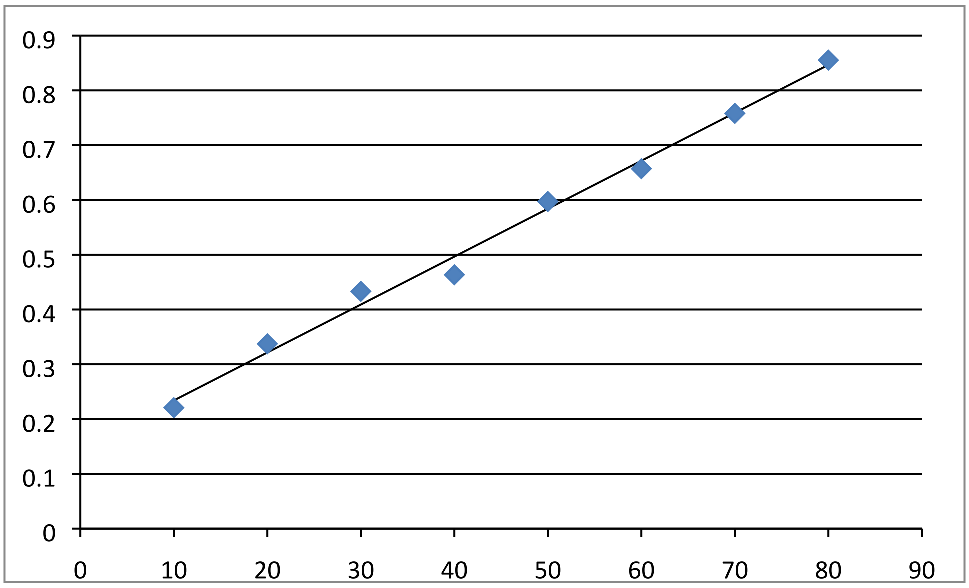

Fig. S2 The calibration curve created with standard Neu5Acyl solutions

Table S2 The absorbance of STn-CRM197 solution

NO.	absorbance
1	0.4795
2	0.4833
3	0.4992
4	0.4772
5	0.4884
6	0.4793
mean	0.484483

$$sialic\ acid\ loading\ (\%) = \frac{38.407}{400} \times 100\% = 9.60\%$$

The carbohydrate loading of **6**, which was analyzed with MALDI-TOF MS (Fig. S3).

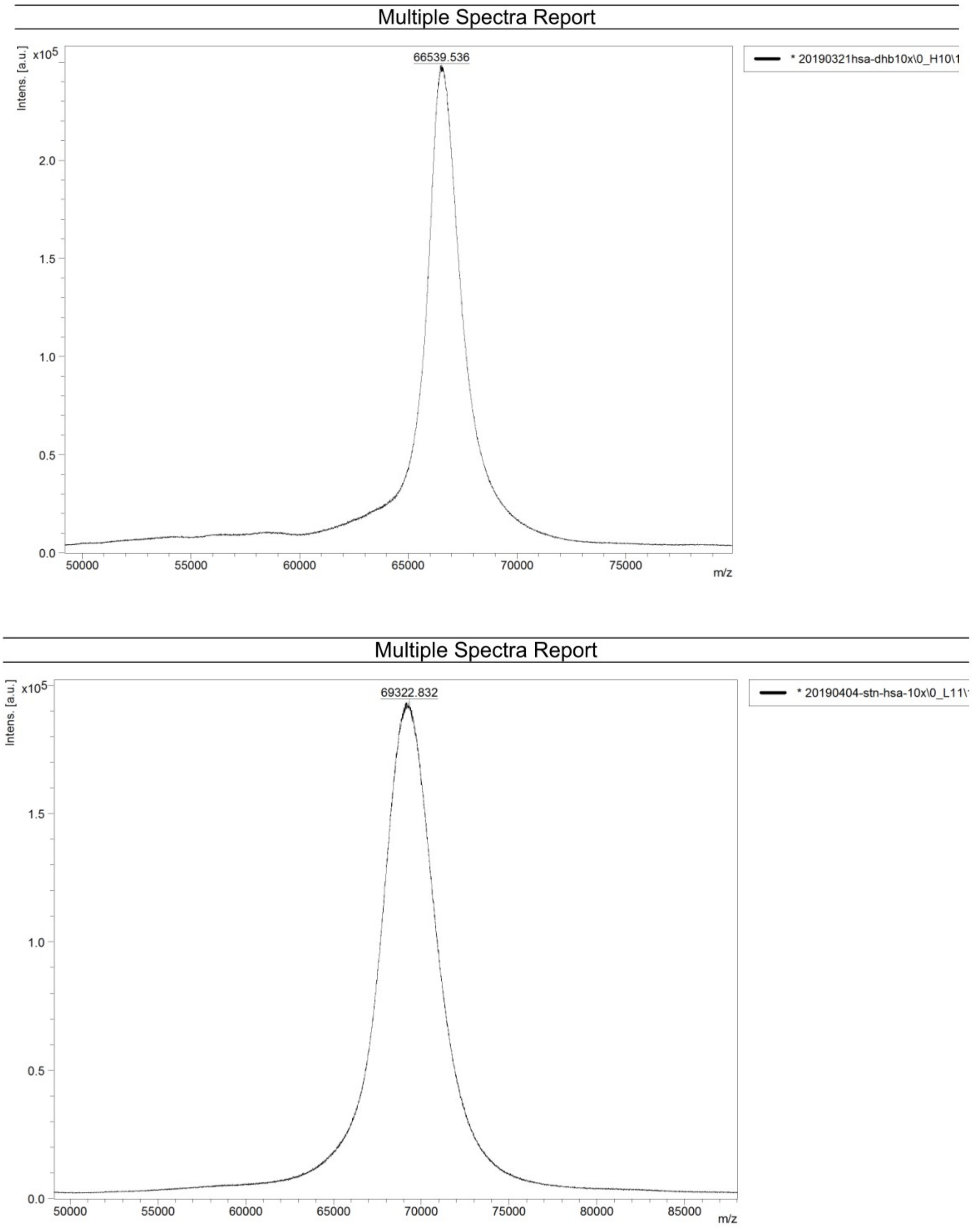

Figure S3. Characterization of STn-HSA

VI. Synthesis of Triazole-HSA Conjugate

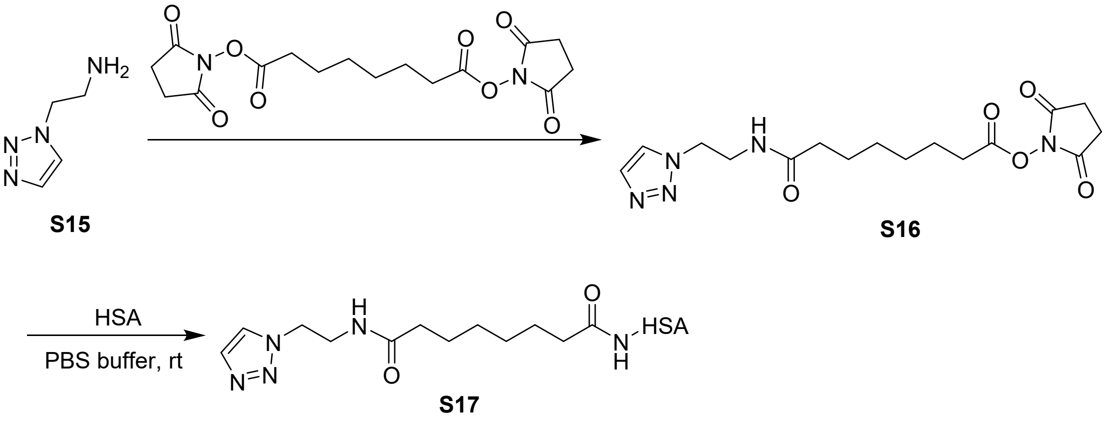

A solution of 2-(1*H*-1,2,3-triazol-1-yl)ethan-1-amine **(S15)** (5 mg, 0.045 mmol), bis(2,5-dioxopyrrolidin-1-yl) octanedioate (20 mg, 0.045 mmol) and triethylamine (5 μL) in anhydrous DMSO was stirred at rt for 5 h. Then, the excess triethylamine was removed in vacuum at rt to give the DMSO solution of **S16**, which was used directly for the next step without purification.

A solution of HSA (2 mg) in 0.5 mL of 0.1 M PBS buffer was gently stirred at rt, then the above solution of **S16** was added with dropwise. After stirring for 2.5 days, the mixture was purified on a Biogel A 0.5 column with 0.1 M PBS buffer as the eluent. The combined fractions containing the glycoconjugate indicated by the bicinchoninic acid (BCA) assay for proteins were dialyzed in distilled water for 2 days, and then lyophilized to obtain the desired conjugates **S17** as white solid.

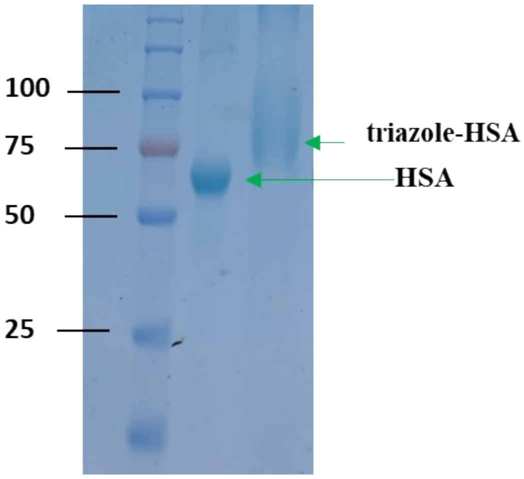

Fig. S4 Triazole-HSA characterization.

VII. Synthesis of Mincle Ligands Vizantin and TDB

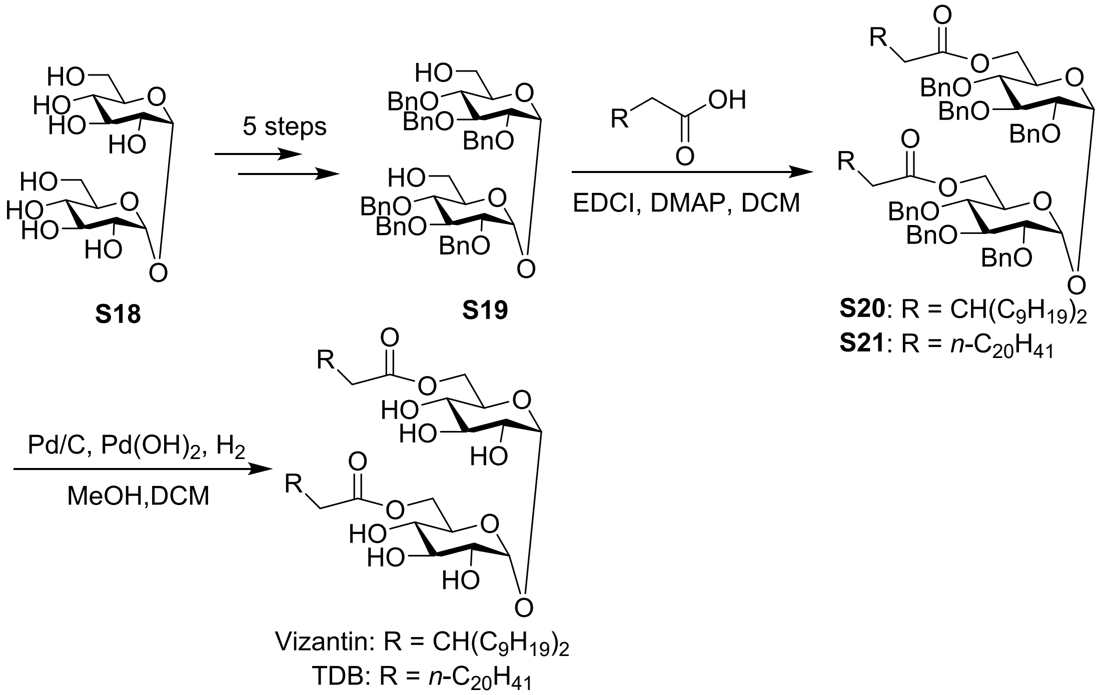

The synthesis of vizantin and TDB started from trehalose according to the literature reported protocol[2]. Some typical experimental details are as follows.

(1) **The synthesis of 2,3,4,2′,3′,4′-Tetra-*O*-benzyl-6,6′-Bis-*O*-(3-nonyldodecanoyl) -*α*,*α*′-trehalose** (**S20**). EDCI (86 mg, 0.45 mmol) was added to a mixture of **S19** (100 mg, 0.11 mmol), **18** (77.5 mg, 0.24 mmol) and DMAP (69 mg, 0.56 mmol) in anhydrous DCM (5 mL) at rt. Then, the mixture was stirred for 6 h under 50 °C. The resulting mixture was diluted with DCM and washed with brine. The organic layer was dried over anhydrous Na_2SO_4 and concentrated in vacuum. The crude product was purified by silica gel column chromatography using PE/EA (10: 1, v/v) as eluent to give the desired product as a colorless oil (20.0 mg, 85%). ^{1}H NMR (400 MHz, $CDCl_3$) δ 7.40 − 7.21 (m, 30H), 5.18 (d, *J* = 3.6 Hz, 2H), 5.00 (d, *J* = 10.8 Hz, 2H), 4.86 (dd, *J* = 10.7 Hz, 4H), 4.76 − 4.64 (m, 4H), 4.32 (dt, *J* = 10.1, 2.8Hz, 2H), 4.15 (dd, *J* = 12.2, 3.4 Hz, 2H), 4.11 – 4.01 (m, 4H), 3.59 – 3.49 (m, 4H), 2.19 (d, *J* = 6.9 Hz, 4H), 1.87 – 1.74 (m, 2H), 1.37 – 1.13 (m, 64H), 0.87 (td, *J* = 6.9, 1.7 Hz, 12H). ^{13}C NMR (100 MHz, $CDCl_3$) δ 173.26, 138.64, 137.82, 128.50, 128.48, 128.43,

128.09, 127.94, 127.89, 127.80, 127.65, 127.47, 94.02,.81.60, 79.45, 77.67, 75.72, 75.31, 72.99, 69.17, 62.40, 39.13, 34.93, 33.82, 31.71, 33.93, 29.96, 29.68, 29.63, 29.36, 26.56, 26.53, 22.71,14.15. HRMS (ESI-TOF) *m/z*: $[M + Na]^+$ calcd for $C_{96}H_{138}O_{13}Na$, 1522.0016; Found, 1522.0012.

(2) **The synthesis of 2,3,4,2′,3′,4′-Tetra-*O*-benzyl -6,6′-Bis-*O*-(1-docosylmethyl)-*α,α′*-trehalose** (**S21**). The synthesis of compound **S21** was similar to **20** just using compound **19** instead of **18**. Colorless oil, yield: 70%. ^{1}H NMR (400 MHz, $CDCl_3$) δ 7.37 − 7.25 (m, 30H), 5.17 (d, *J* = 3.6 Hz, 2H), 5.00 (d, *J* = 10.8 Hz, 2H), 4.86 (dd, *J* = 10.7, 2.6 Hz, 4H), 4.75 − 4.65 (m, 4H), 4.52 (d, *J* = 10.6 Hz, 2H), 4.26 – 4.19 (m, 2H), 4.18 – 4.12 (m, 2H), 4.10 – 4.02 (m, 4H), 3.61 – 3.52 (m, 4H), 2.23 (dd, *J* = 8.3, 6.8 Hz, 4H), 1.24 (m, 72H), 0.92 – 0.85 (m, 6H). ^{13}C NMR (100 MHz, $CDCl_3$) δ 173.48, 138.62, 137.82, 128.57, 128.52, 128.50, 128.46, 128.32, 128.14, 127.98, 127.96, 127.83, 127.69, 127.49, 94.03, 81.64, 79.38, 77.56, 77.26, 75.74,75.24, 72.99, 69.19, 62.55, 34.15, 31.97, 29.75, 29.70, 29.65, 29.52, 29.40, 29.31, 29.19, 24.91, 22.73, 14.17. HRMS (ESI-TOF) *m/z*: $[M + Na]^+$ calcd for $C_{98}H_{142}O_{13}Na$, 1551.0365; Found, 1551.0368.

(3) **The synthesis of vizantin.** A mixture of **S20** (200.0 mg), Pd $(OH)_2$ (68.0 mg) and Pd/C (65.0 mg) was stirred in the solution of DCM and MeOH (3: 1, v/v, 40.0 mL) under a H_2 atmosphere at rt for 6h. The reaction mixture was filtered through a pad of Celite and the filtrate was concentrated in vacuum to give vizantin (99.0 mg, 77.3%). ^{1}H NMR (400 MHz, MeOD/$CDCl_3$) δ 5.11 (d, *J* = 3.5 Hz, 2H, H-1), 4.31 (d, *J* = 2.3 Hz, 4H), 4.04 – 3.90 (m, 2H), 3.80 (t, *J* = 9.2 Hz, 2H), 3.53 (m, 2H), 3.38 (s, 2H), 2.29 (d, *J* = 6.9 Hz, 4H), 1.84 (s, 2H), 1.27 (s, 64H), 0.98 – 0.70 (m, 12H). ^{13}C NMR (100 MHz, CD_3OD/$CDCl_3$) δ 174.93, 94.44, 73.82, 72.35, 71.12, 70.87, 63.71, 39.61, 35.45, 34.24, 32.43, 30.42, 30.15, 30.13, 30.03, 29.87, 27.02, 27.00, 23.18, 14.46. HRMS (ESI-TOF) *m/z*: $[M + Na]^+$ calcd for $C_{54}H_{102}O_{13}Na$, 981.7213; Found, 981.7210.

(4) **The synthesis of TDB.** The synthesis of compound **TDB** was similar to **vizantin** just using compound **S21** instead of **S20**. Yield: 96.2%. ^{1}H NMR (400 MHz,

MeOD/$CDCl_3$) δ 5.02 (d, *J* = 2.8 Hz, 2H, H-1), 4.34 – 4.14 (m, 4H), 3.98 – 3.87 (m, 2H), 3.72 (s, 2H), 3.45 (d, *J* = 8.4 Hz, 2H), 3.30 (d, *J* = 7.4 Hz, 2H), 2.26 (t, *J* = 7.5 Hz, 4H), 1.54 (t, *J* = 7.2 Hz, 4H), 1.18 (s, 72H), 0.80 (t, *J* = 6.7 Hz, 6H). ^{13}C NMR (100 MHz, $CD_3OD/CDCl_3$) δ 175.18, 94.26, 73.89, 72.28, 71.01, 70.68, 63.83, 34.70, 32.47, 30.23, 30.18, 30.04, 29.89, 29.83, 29.69, 25.43, 23.20, 14.43. HRMS (ESI-TOF) *m/z*: $[M + Na]^+$ calcd for $C_{56}H_{106}O_{13}Na$, 1009.7521; Found, 1009.7507.

VIII. Binding affinity of conjugates 1-4 to hMincle

Table S3 The binding affinity of conjugates **1-4** to hMincle-Fc and hMincle-His proteins

	OD (hMincle-Fc)			OD (hMincle-His)		
	Mean	SD	N	Mean	SD	N
Control	0.09	0.01	3	0.09	0.01	3
DSPC/Chol	0.20	0.13	3	0.39	0.08	3
Vizantin	3.14	0.15	3	2.93	0.06	3
TDB	3.06	0.11	3	2.62	0.07	3
Conj. **1**	3.47	0.07	3	3.18	0.24	3
Conj. **2**	3.11	0.11	3	2.63	0.04	3
Conj. **3**	3.28	0.17	3	3.08	0.15	3
Conj. **4**	3.20	0.13	3	2.84	0.10	3

IX. Abilities of conjugates 1-4 to induce the production of TNF-α and IL-6

Table S4 The capabilities of conjugates **1-4** to induce BMDMs to produce inflammatory cytokines IL-6 and TNF-α

	IL-6						TNF-α					
Control	DMSO Solution			DSPC/Chol			DMSO Solution			DSPC/Chol		
	Mean	SD	N	Mean	SD	N	Mean	SD	N	Mean	SD	N
Vizantin	3.39	1.92	3	8.37	2.79	3	6.12	0.27	3	17.08	4.74	3
LPS	60.39	5.53	3	106.99	4.00	3	101.34	10.82	3	158.39	4.35	3
Vizantin	37.05	7.82	3	86.95	3.65	3	74.42	7.30	3	132.83	16.41	3
TDB	37.64	8.21	3	76.31	7.25	3	74.37	3.10	3	112.36	20.98	3
Conj. **1**	58.92	7.17	3	106.17	3.82	3	66.24	4.49	3	155.08	3.05	3
Conj. **2**	50.21	10.21	3	78.03	21.46	3	54.83	11.21	3	142.55	5.39	3

Conj. **3**	32.98	1.88	3	69.24	20.63	3	39.54	3.52	3	130.48	6.63	3
Conj. **4**	41.33	4.00	3	79.81	21.53	3	55.61	4.81	3	133.19	5.50	3

X. Liposomes Size Analysis of the Conjugates 1-4

The liposomal size of conjugates **1-4** prepared for the immunization of mice was detected by laser particle size meter. The sample was tested three times, and the results are listed in Table S5. It was concluded that the average diameter of conjugate **1**, **2**, **3**, and **4** were 700.3 ± 192.0 (SD), 717.0 ± 62.9, 890.5 ± 94.4, and 789.5 ± 51.6 nm, respectively. The polydispersity index (PDI) of them were around 0.2200, 0.2150, 0.3060, and 0.111, respectively (Table S5-S8).

Table S5 Liposomes size analysis results of conjugate 1

Conjugate 1

Test	PDI	Size(d,nm)
**#1**	0.1650	515.7000
**#2**	0.1310	686.3000
**#3**	0.3640	898.9000
**Ave**	0.2200	700.3000
**SD**	0.1260	192.0000

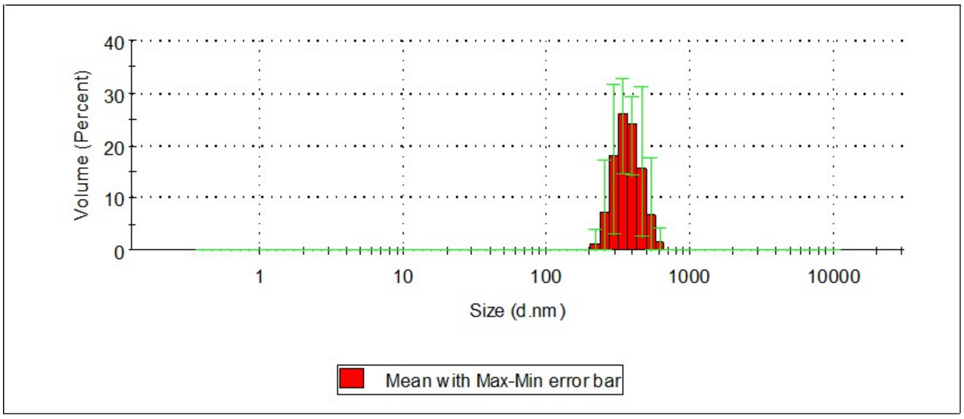

Fig. S5. Size distribution of the liposomes of conjugate **1**

Table S6 Liposomes size analysis results of conjugate **2**

Conjugate 2

Test	PDI	Size(d,nm)

**#1**	0.2580	698.1000
**#2**	0.3440	787.2000
**#3**	0.0430	665.6000
**Ave**	0.215	717.0000
**SD**	0.155	62.9600

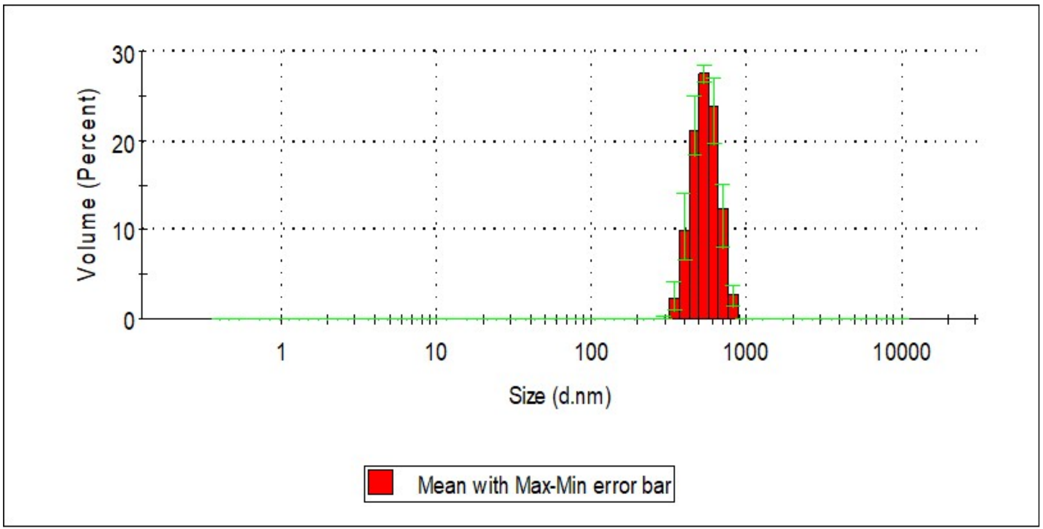

Fig. S6. Size distribution of the liposomes of conjugate **2**

Table S7 Liposomes size analysis results of conjugate **3**

**Test**	**PDI**	**Size(d,nm)**
**#1**	0.2810	800.6000
**#2**	0.2880	988.9000
**#3**	0.3480	881.9000
**Ave**	0.3060	890.5000
**SD**	0.0370	94.4400

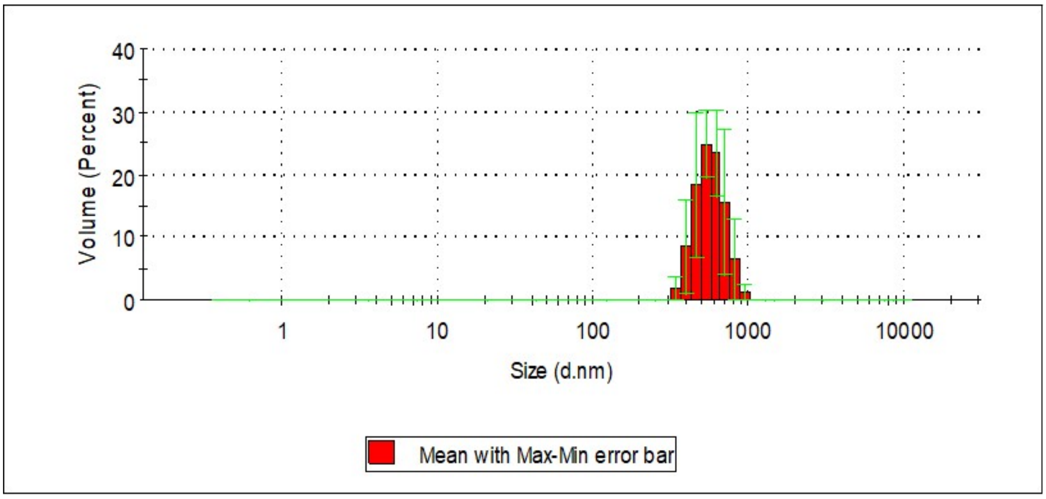

Fig. S7. Size distribution of the liposomes of conjugate **3**

Table S8 Liposomes size analysis results of conjugate **4**

Conjugate 4		
**Test**	**PDI**	**Size(d,nm)**
**1**	0.0970	729.9000
**2**	0.0200	819.2000
**3**	0.2170	819.4000
**Ave**	0.111	789.5000
**SD**	0.099	51.6200

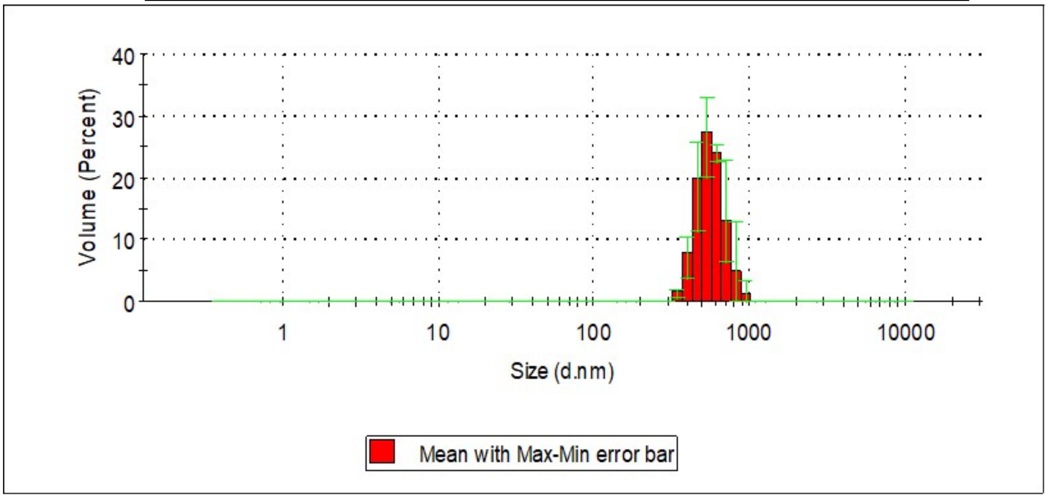

Fig. S8. Size distribution of the liposomes of conjugate **4**

XI. Calculated Antibody Titers of ELISA Experiments

Table S9 The Ig M antibody titers of pooled day 21, 27, and 38 sera derived from mice immunized with conjugate **1-5**

	d21			d27			d38		
	Mean	SD	N	Mean	SD	N	Mean	SD	N
Conj.1	15105	2022	3	23824	3885	3	16963	4484	3
Conj.2	45964	3755	3	51308	1795	3	29145	4025	3
Conj.3	33306	4884	3	46726	3726	3	30318	5805	3
Conj.4	5411	2402	3	37558	3866	3	32386	2554	3
Conj.5	55113	1143	3	69818	2123	3	38752	7814	3

Table S10 The Ig G antibody titers of pooled day 21, 27, and 38 sera derived from mice immunized with conjugate **1-5**

	d21			d27			d38		
	Mean	SD	N	Mean	SD	N	Mean	SD	N
Conj.1	48583	2723	3	75388	2585	3	157970	5385	3
Conj.2	14426	302	3	42619	426	3	94855	1635	3
Conj.3	31264	832	3	65737	1007	3	156389	2695	3
Conj.4	14668	304	3	40971	4367	3	112054	1708	3
Conj.5	41782	1098	3	78775	4348	3	97150	4390	3

Table S11 The antibody titers of Kappa, IgG subclasses, and IgM in mice antisera on day 38 induced by conjugate **1**

Mouse	1	2	3	4	5	6	Mean
Kappa	221904	112420	102744	140084	154817	76115	134681
IgG1	77652	200787	34201	16318	18215	156373	83924
IgG2a	92041	117008	96761	46630	80017	94845	87884
IgG2b	52575	56954	80822	26108	26108	94845	56235

IgG3	11048	29144	13494	16815	8691	15835	15838
IgM	39340	37421	37049	35954	33523	35596	36481

Table S12 The antibody titers of Kappa, IgG subclasses, and IgM in mice antisera on day 38 induced by conjugate **2**

Mouse	1	2	3	4	5	6	Mean
Kappa	40946	92967	79221	49021	47572	45707	59239
IgG1	15063	6974	46166	15678	17501	5115	17750
IgG2a	52575	57526	97733	52575	67508	58689	64434
IgG2b	29437	16984	26370	36316	19536	18398	24507
IgG3	14765	10301	14765	12088	11384	9897	12200
IgM	38561	27174	33523	34201	28567	28567	31765

Table S13 The antibody titers of Kappa, IgG subclasses, and IgM in mice antisera on day 38 induced by conjugate **3**

Mouse	1	2	3	4	5	6	Mean
Kappa	101722	92042	276509	30946	31257	144351	112805
IgG1	103777	78433	105873	92967	137311	111302	104944
IgG2a	14045	28001	28283	34201	43915	27889	29389
IgG2b	8866	25848	18034	21163	40135	23156	22867
IgG3	493	5014	2540	1901	1703	953	2101
IgM	1863	16814	5826	11271	3678	2122	6929

Table S14 The antibody titers of Kappa, IgG subclasses, and IgM in mice antisera on day 38 induced by conjugate **4**

Mouse	1	2	3	4	5	6	Mean
Kappa	128028	110194	77653	194853	179872	74608	127534
IgG1	32860	43045	12333	58689	29437	27723	34014
IgG2a	108012	110194	101722	113550	121784	103777	109840
IgG2b	39735	66836	48050	99708	30638	57526	57082
IgG3	41773	30946	16482	19930	30946	43478	30592
IgM	40135	39340	21163	51021	50514	43478	40942

Table S15 The antibody titers of Kappa, IgG subclasses, and IgM in mice antisera on day 38 induced by conjugate **5**

Mouse	1	2	3	4	5	6	Mean
Kappa	185350	196811	101722	36315	35596	34201	98333
IgG1	54176	72403	54721	106937	38561	97734	70755
IgG2a	880	934	198	589	450	77	522
IgG2b	19732	9228	12333	16155	6836	8350	12106
IgG3	23861	14618	2864	1436	3752	13767	10050
IgM	37421	42617	22248	18770	40946	36680	33114

Table S16 Titers of IgG antibody reactive to triazole-HSA in the pooled day 38 sera obtained with conjugate **1**-**4**.

	Mean	SD	N
1	43612	1603	3
2	27552	7047	3
3	16546	3570	3
4	18609	7938	3

Table S17 Titers of IgG antibody reactive to trehalose derivative in the pooled day 38 sera obtained with conjugate **1-5**

	Mean	SD	N
1	24680	4221	3
2	20961	5381	3
3	17811	3711	3
4	24919	5498	3
5	245	32	3

Table S18 Titers of IgG antibody reactive to CRM197 in the pooled day 38 sera obtained with conjugate **1-5**

	Mean	SD	N
1	413	285	3
2	438	206	3
3	184	234	3
4	638	157	3
5	203014	11217	3

XII. ELISpot Assay

Table S19 IFN-γ and IL-4 spot-forming cells responding to conjugates **1-5**

	IFN-γ			IL-4		
	Mean	SD	N	Mean	SD	N
Control	5	2	3	13.7	3.1	3
1	124.3	9.5	3	467.3	14.8	3
2	226.0	81.0	3	130.7	29.3	3
3	194.7	24.5	3	398.7	58.4	3
4	37.0	13.0	3	93.3	36.5	3
5	42.3	7.5	3	198	40.3	3

XIII. FACS Analyses

Table S20 Mean FITC-A and collected MCF-7 cells of each group

Name	NS	**1**	**2**	**3**	**4**	**5**
Mean FITC-A	1006	33071	21436	26351	19808	11911
Cells	10000	10000	10000	10000	10000	10000

Table S21 Mean FITC-A and collected CT-26 cells of each group

Name	NS	**1**	**2**	**3**	**4**	**5**
Mean FITC-A	8680	42705	15464	18643	14873	18336
Cells	10000	10000	10000	10000	10000	10000

Table S22 Mean FITC-A and collected B16-F10 cells of each group

Name	NS	**1**	**2**	**3**	**4**	**5**
Mean FITC-A	13170	13457	10413	10639	10729	11872
Cells	10000	10000	10000	10000	10000	10000

XIV. Antibody-Mediated Complement-Dependent Cytotoxicity (CDC)

Table S23 lysis of MCF-7 cancer cell through antibody-mediated CDC

Name	NS	**1**	**2**	**3**	**4**	**5**
Mean	11.6	61.7	53.1	60.3	52.8	46.3
SD	5.9	4.3	5.6	6.3	6.1	5.4
N	6	6	6	6	6	6

Table S24 lysis of CT-26 cancer cell through antibody-mediated CDC

Name	NS	**1**	**2**	**3**	**4**	**5**
Mean	15.1	37.3	35.4	32.0	33.4	33.7
SD	2.1	4.1	3.6	3.5	6.5	3.2
N	6	6	6	6	6	6

Table S25 lysis of B16-F10 cancer cell through antibody-mediated CDC

Name	NS	**1**	**2**	**3**	**4**	**5**
Mean	3.9	5.3	4.6	5.2	5.9	5.2
SD	2.4	2.9	2.2	2.5	2.5	2.6
N	6	6	6	6	6	6

XV. Tumor Challenge Studies

Table S26 Tumor sizes (mm^3) with time

Group	Days	10	12	14	16	18	20	23	25	27
	Mean	229.97	631.98	1651.87	1882.77	2131.23	--	--	--	--
PBS	SD	122.81	316.19	1594.28	510.06	478.63	--	--	--	--
	N	8	8	7	6	3	--	--	--	--
	Mean	226.64	466.95	875.26	1776.06	2740.53	2539.44	--	--	--
PBS/CP	SD	69.82	281.34	304.17	299.55	927.25	447.56	--	--	--
	N	8	8	8	8	5	3	--	--	--
	Mean	17.81	37.09	79.08	107.06	201.57	271.19	479.23	518.97	760.79
1/CP	SD	11.13	28.01	38.96	66.78	86.12	225.11	299.12	329.01	356.84
	N	8	8	8	8	8	8	8	8	8
	Mean	14.57	16.24	62.24	97.86	280.07	354.46	607.71	724.63	947.07
1	SD	4.62	9.51	38.84	81.32	201.13	242.27	366.28	538.45	595.92
	N	8	8	8	8	8	8	8	7	7
	Mean	20.7	48.52	110.64	201.16	310.19	543.60	915.12	1087.31	1403.21
2/CP	SD	4.18	16.39	31.16	107.85	86.42	364.11	623.39	670.98	741.07
	N	6	6	6	6	6	6	6	6	5
	Mean	14.41	30.96	71.58	99.73	186.98	379.67	666.53	907.11	1532.50
3/CP	SD	10.36	20.48	22.98	57.85	78.55	132.06	256.64	452.54	606.29
	N	6	6	6	6	6	6	6	6	6
	Mean	23.62	47.58	90.33	131.49	238.15	326.01	646.61	909.99	1321.60
4/CP	SD	12.00	35.27	54.52	83.05	145.61	177.16	421.08	576.67	832.17
	N	8	8	8	8	8	8	8	8	8
	Mean	20.43	28.10	75.91	101.87	266.60	354.05	617.47	948.25	1250.21
4	SD	14.25	21.14	64.96	86.79	235.92	317.63	535.63	888.01	1202.45
	N	8	8	8	8	8	8	8	8	8
	Mean	63.28	110.40	159.75	228.66	324.62	553.63	990.64	1623.35	1879.82
5/Al/CP	SD	41.80	64.77	57.21	130.23	145.80	195.68	397.24	603.50	679.30
	N	8	8	8	8	8	8	8	7	6

Table S27 Survival time (day) of each mouse

	PBS	PBS/CP	1/CP	1	2/CP	3/CP	4/CP	4	5/Al/CP

1	12	16	30	29	23	18	27	27	23
2	14	16	32	29	25	27	27	27	25
3	16	16	34	31	30	29	30	29	27
4	16	18	34	34	30	30	32	31	27
5	16	18	34	36	34	30	36	34	29
6	18	19	41	36	41	32	39	34	29
7	18	20	43	36	--	--	41	34	31
8	18	20	48	43	--	--	42	36	31

References

1 V. A. Sarpe and S. S. Kulkarni, *Org. Biomol. Chem.*, 2013, **11**, 6460–6465.

2 H. Yamamoto, M. Oda, M. Nakano, N. Watanabe, K. Yabiku, M. Shibutani, M. Inoue, H. Imagawa, M. Nagahama, S. Himeno, K. Setsu, J. Sakurai and M. Nishizawa, *J. Med. Chem.*, 2013, **56**, 381–385.

3 J. Wu and Z. Guo, *Bioconjug. Chem.*, 2006, **17**, 1537–1544.

4 Q. Wang, S. A. Ekanayaka, J. Wu, J. Zhang and Z. Guo, *Bioconjug. Chem.*, 2008, **19**, 2060–2067.

5 D. V. Zinov’ev and P. Sole, *Probl. Peredachi Informatsii*, 2004, **40**, 50–62.

6 L. Svennerholm, *Biochim. Biophys. Acta*, 1957, **24**, 604–611.

NMR and MS Spectra of Synthesized Compounds

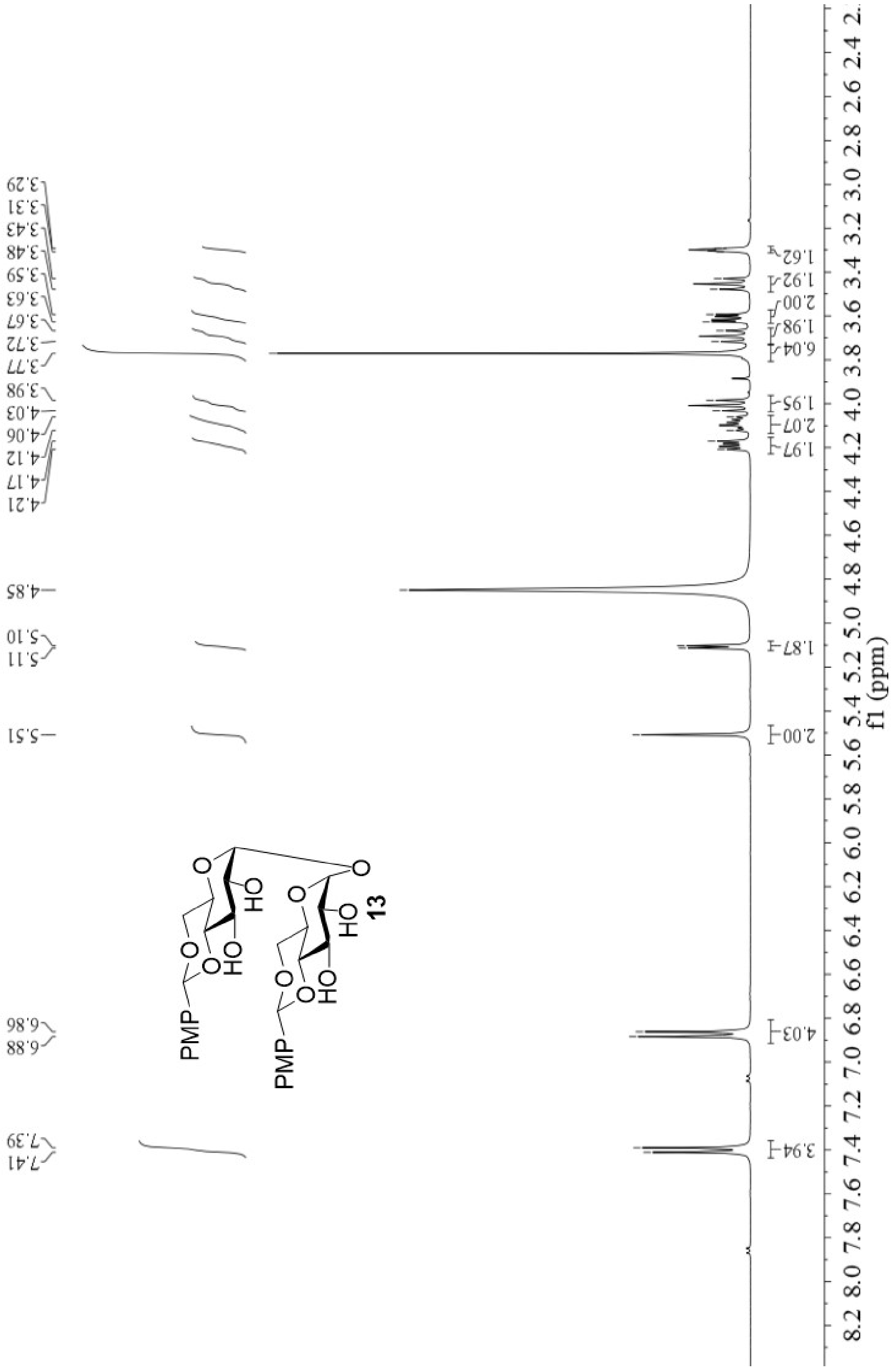

^{1}H NMR Spectrum of compound **13** ($CDCl_3$, 400 MHz)

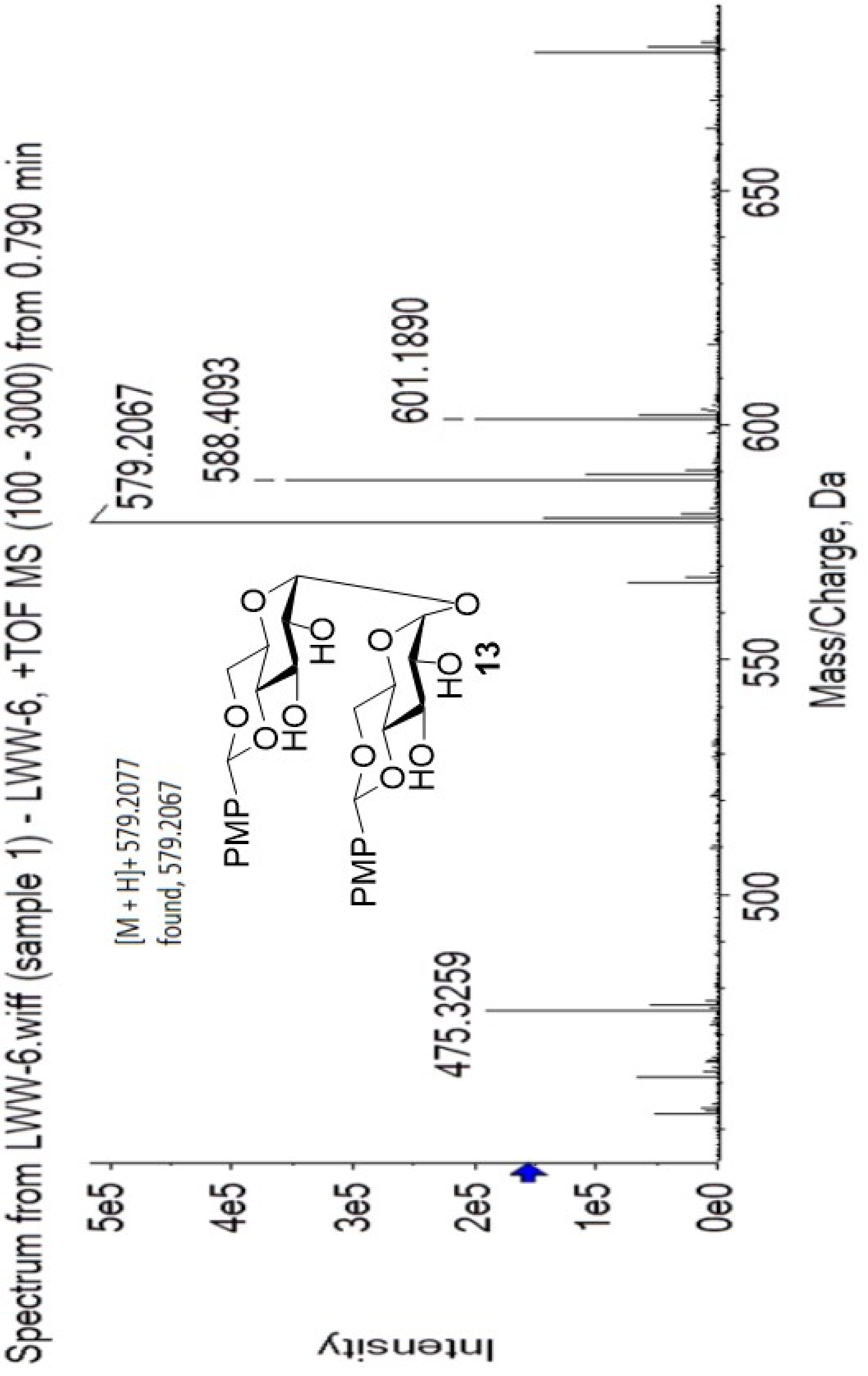

HR-ESI-MS spectrum of conjugate **13**

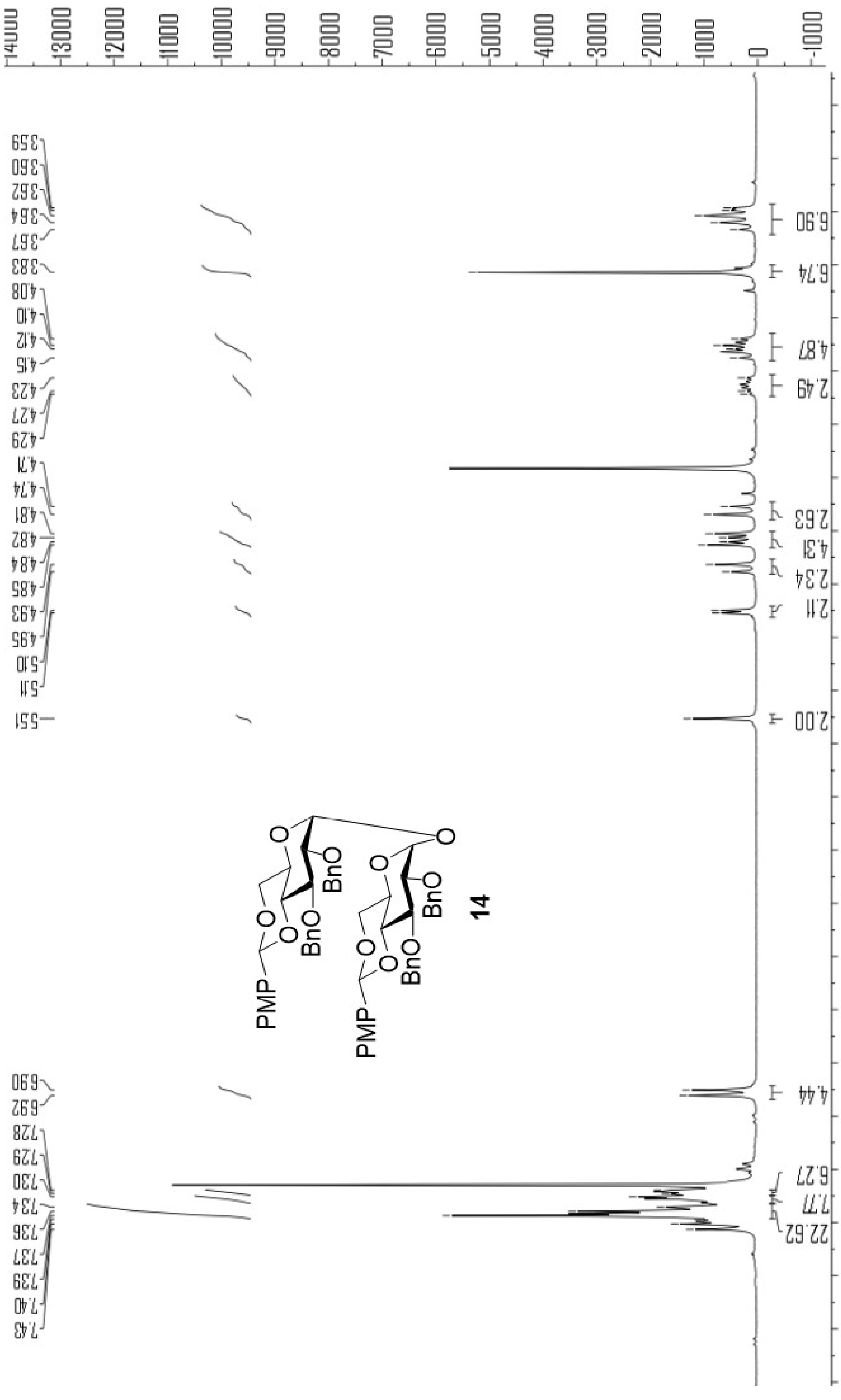

^{1}H NMR Spectrum of compound **14** ($CDCl_3$, 400 MHz)

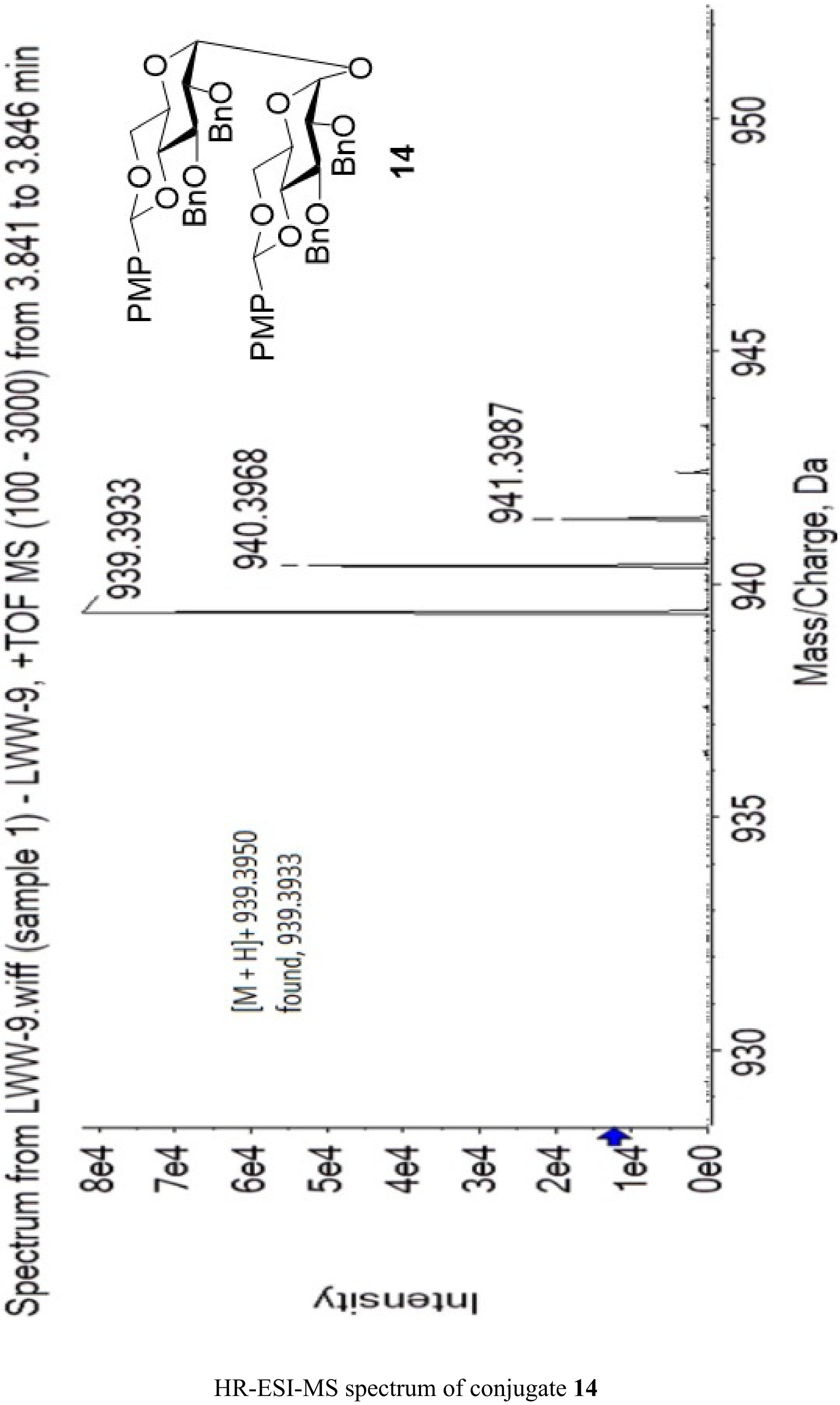

HR-ESI-MS spectrum of conjugate **14**

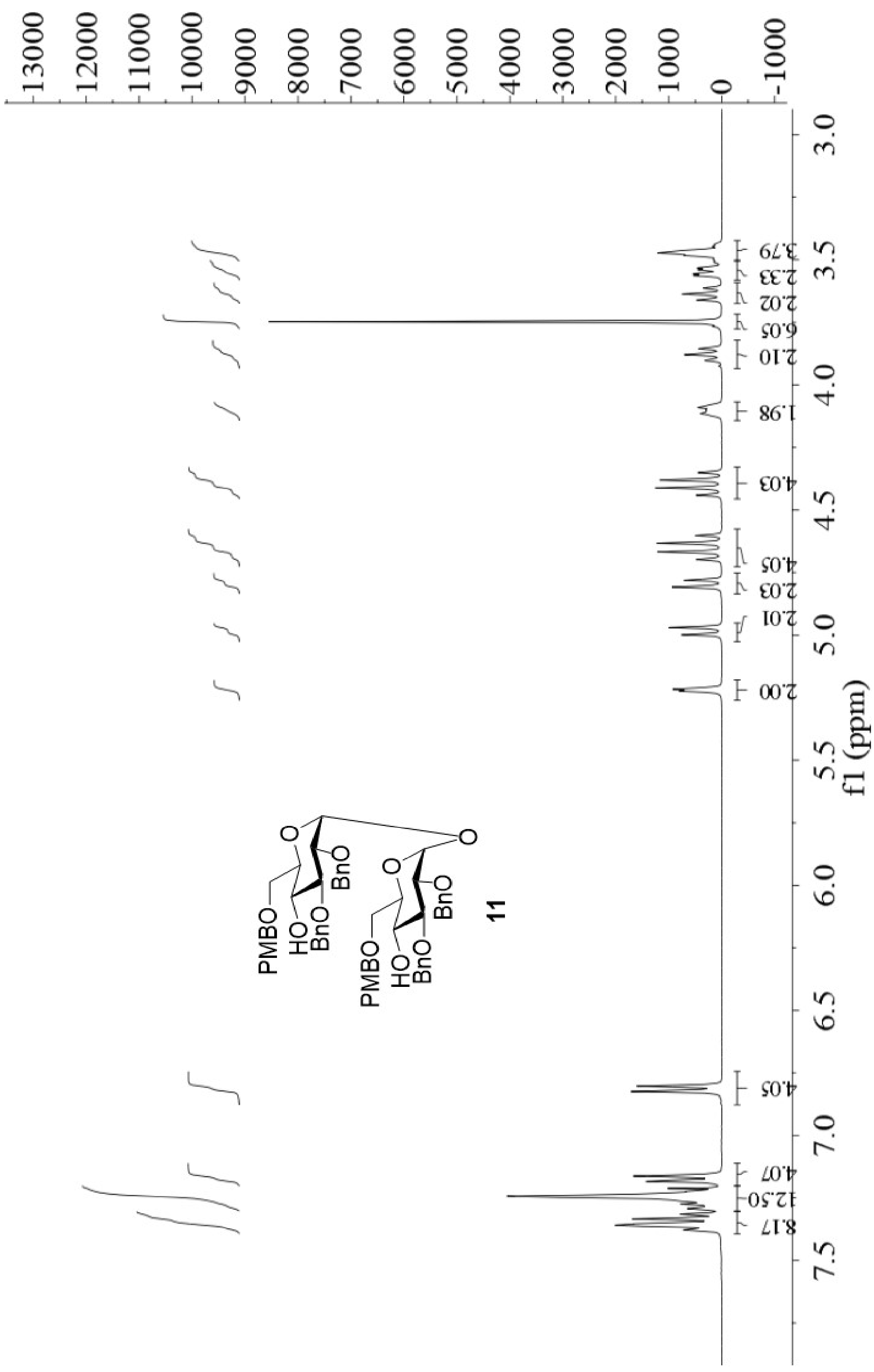

^{1}H NMR Spectrum of compound **11** ($CDCl_3$, 400 MHz)

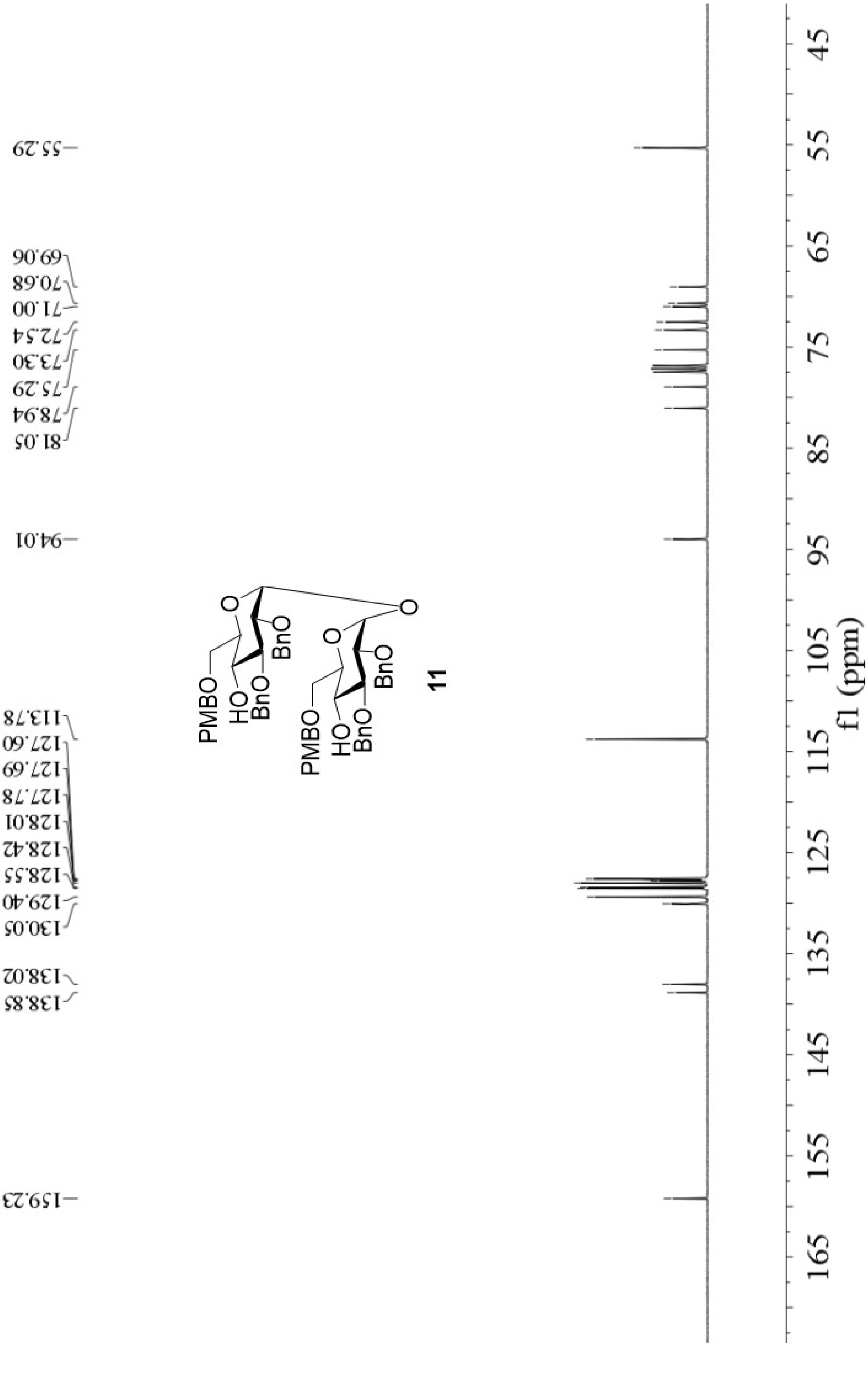

^{13}C NMR Spectrum of compound **11** ($CDCl_3$, 400 MHz)

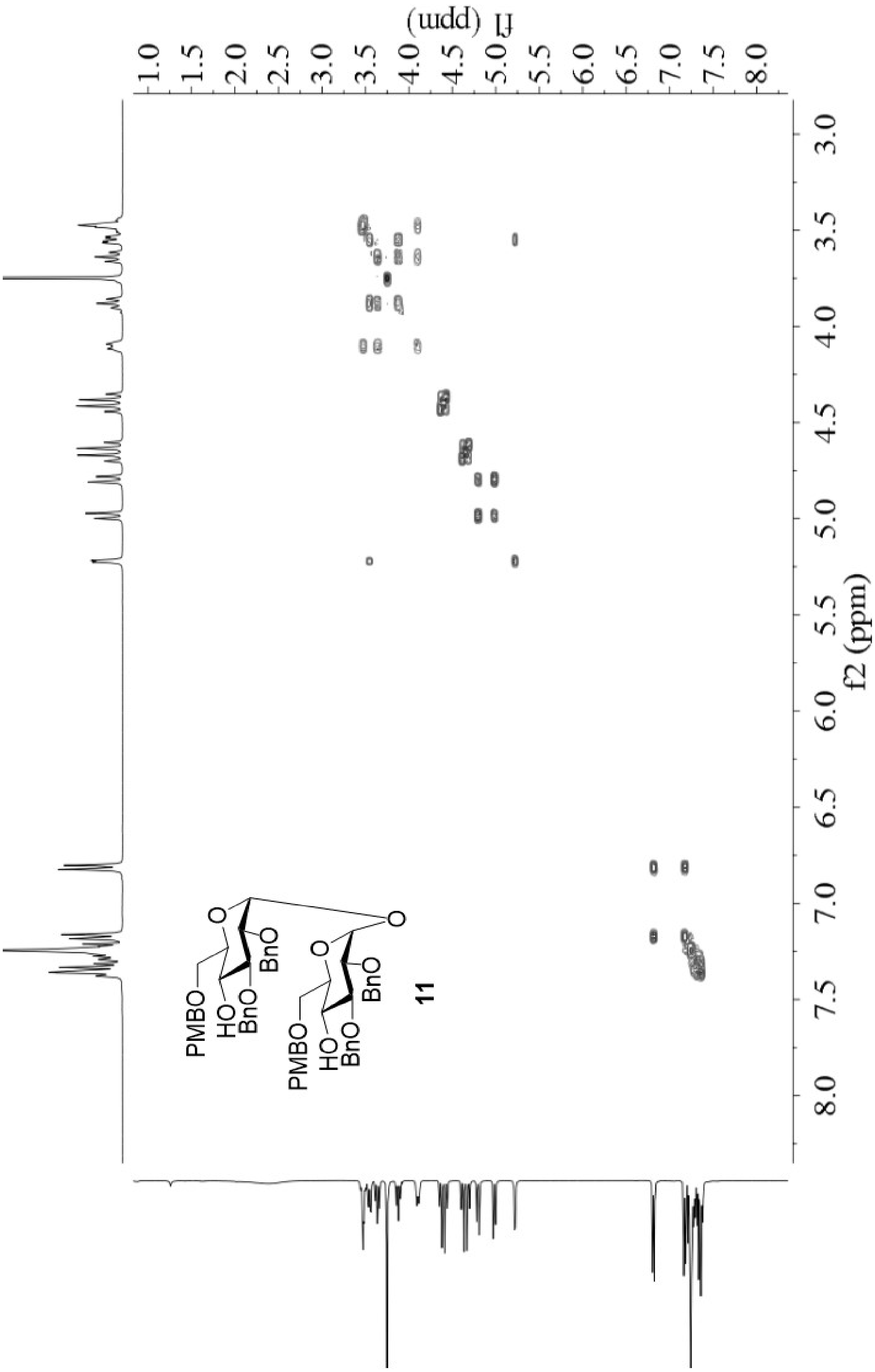

^{1}H-^{1}H COSY NMR Spectrum of compound **11** ($CDCl_3$, 400 MHz)

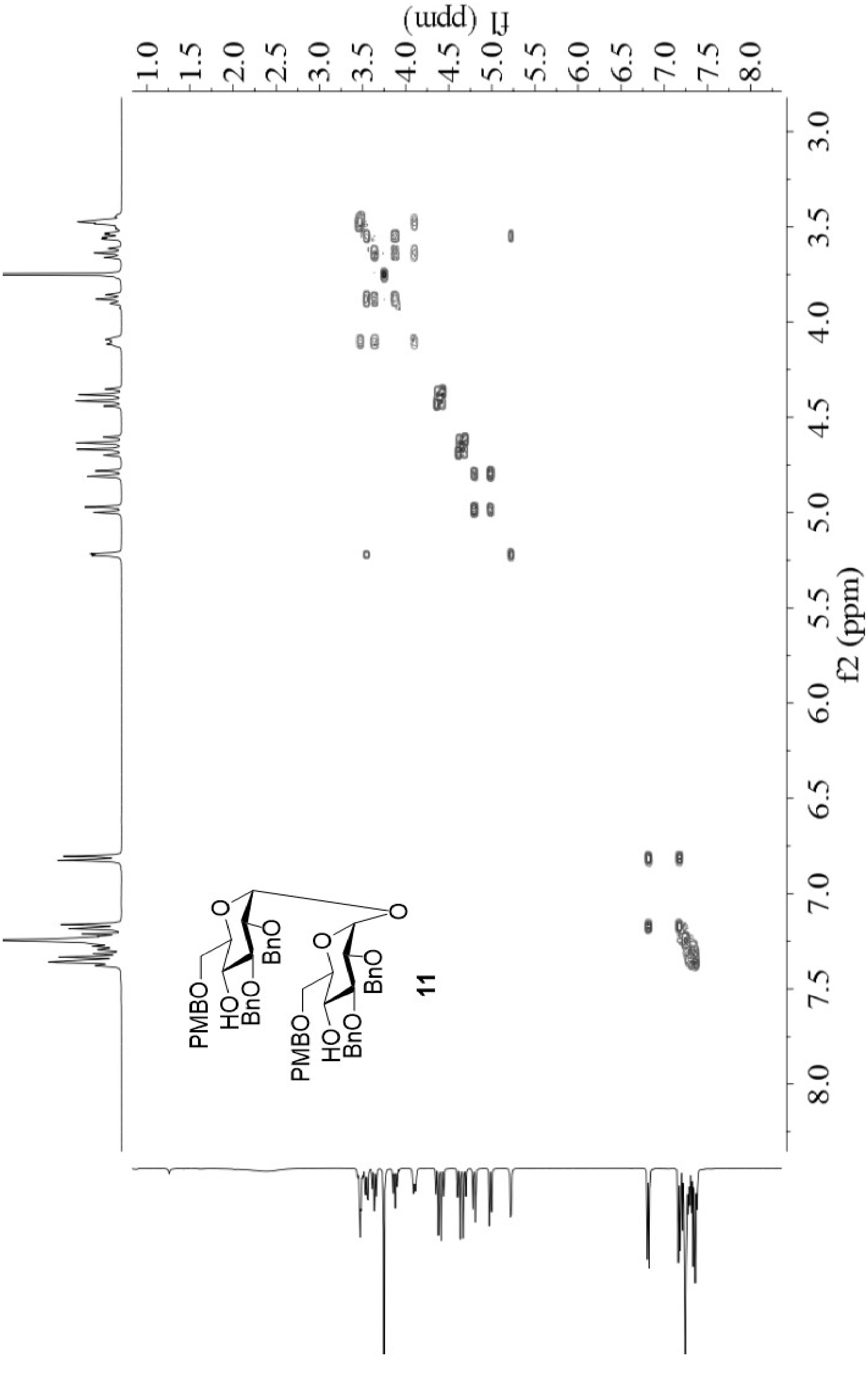

HSQC NMR Spectrum of compound **11** ($CDCl_3$, 400 MHz)

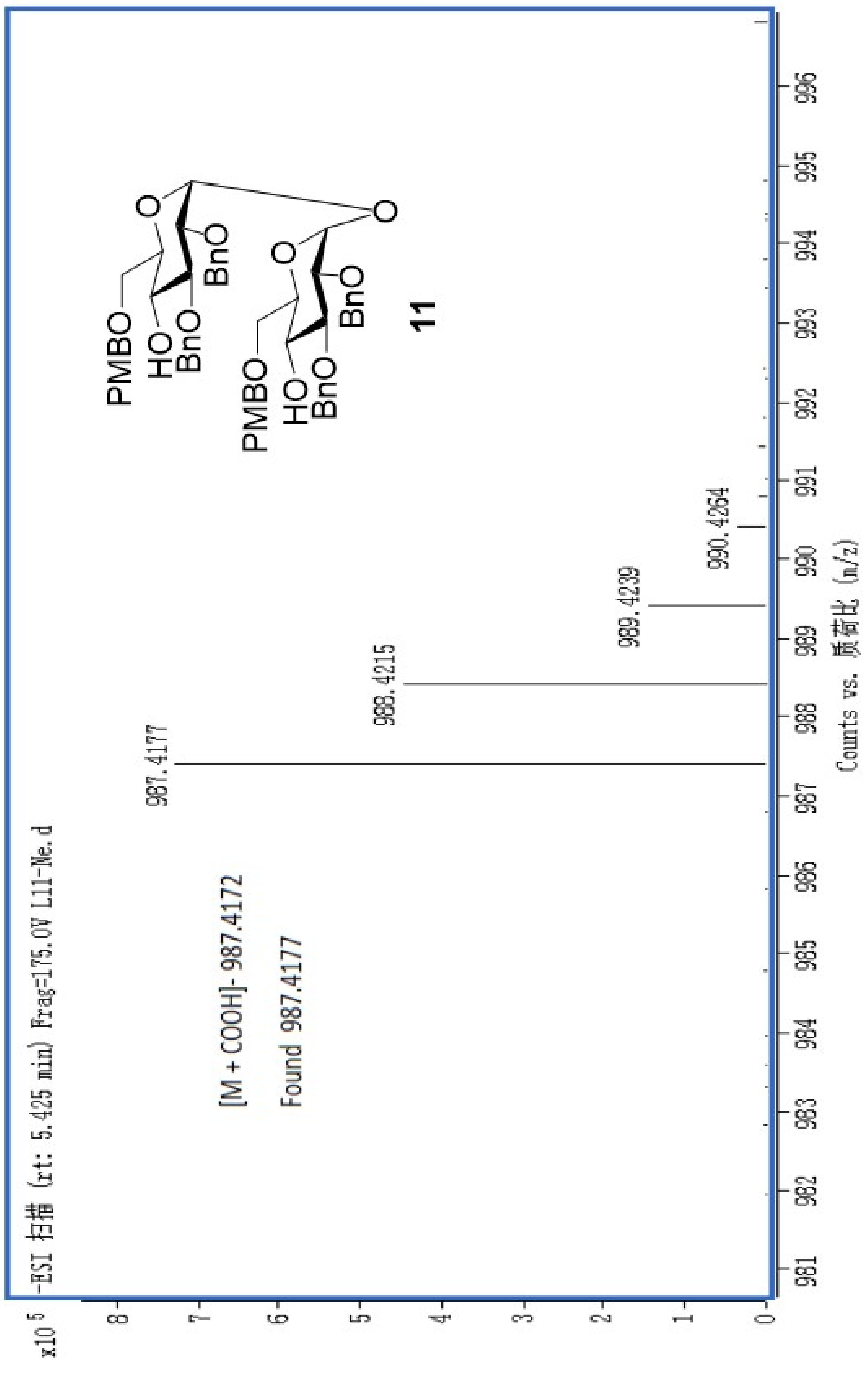

HR-ESI-MS spectrum of conjugate **11**

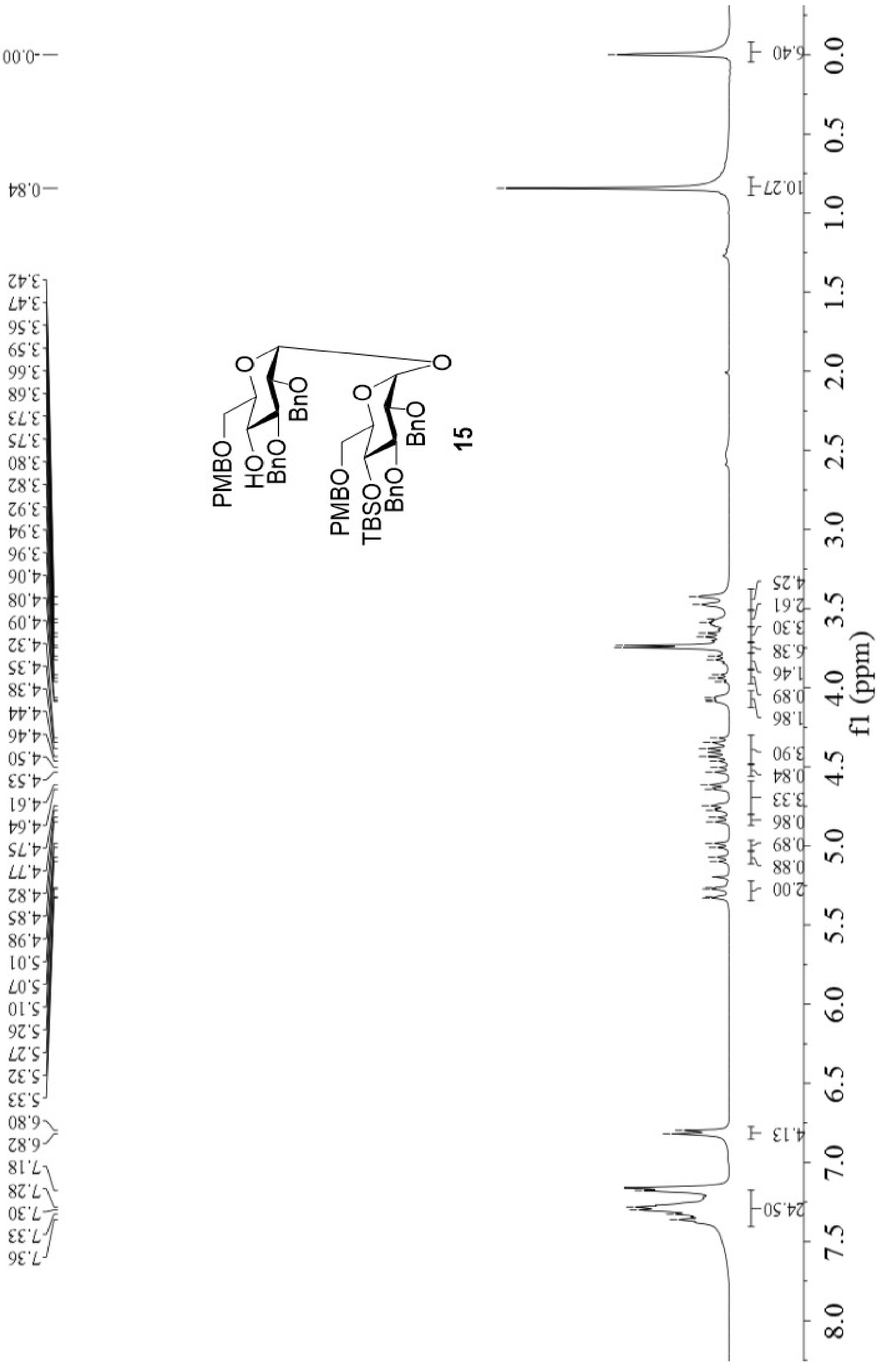

^{1}H NMR Spectrum of compound **15** ($CDCl_3$, 400 MHz)

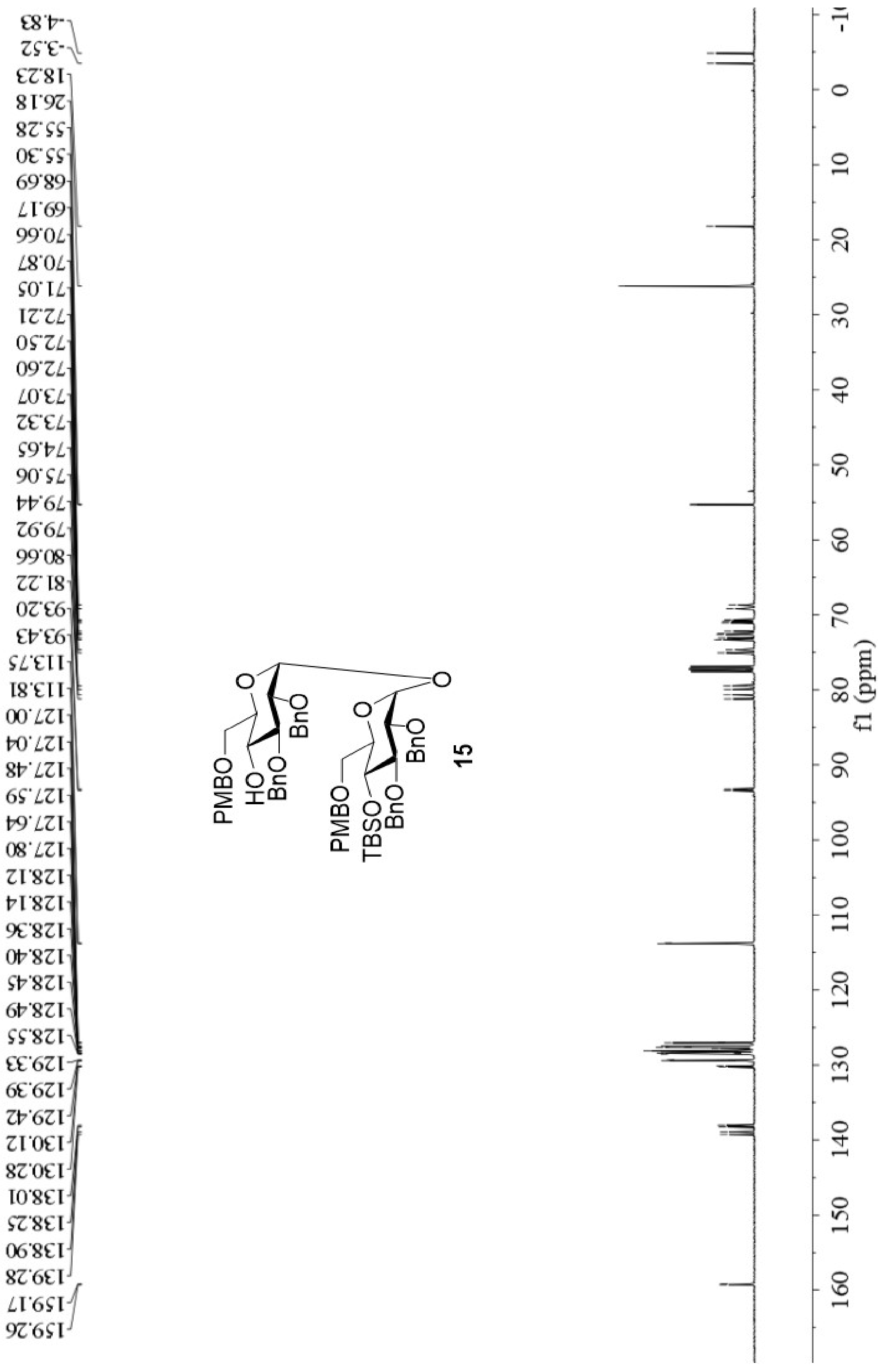

^{13}C NMR Spectrum of compound **15** ($CDCl_3$, 400 MHz)

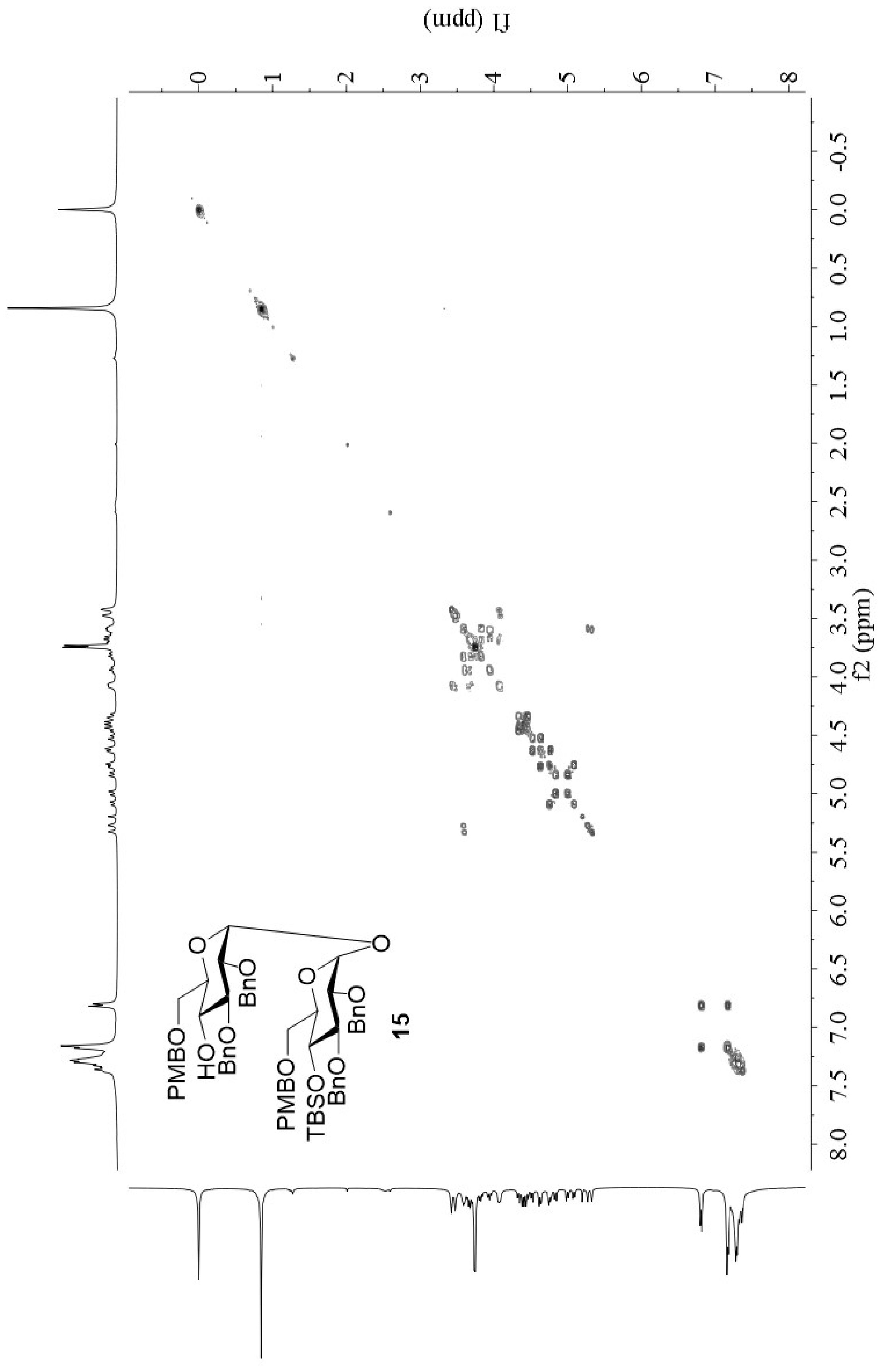

^{1}H-^{1}H COSY NMR Spectrum of compound **15** ($CDCl_3$, 400 MHz)

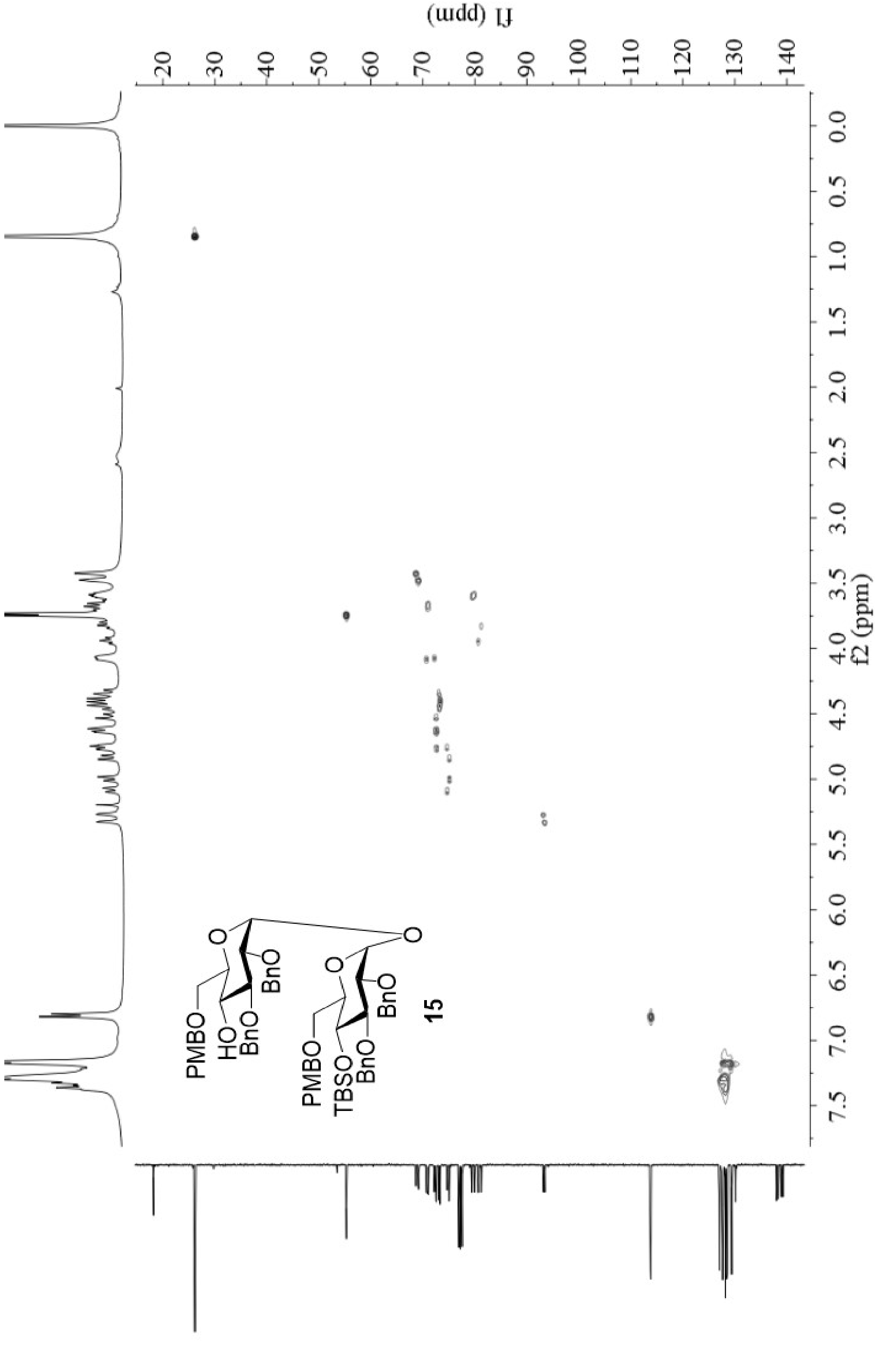

HSQC NMR Spectrum of compound **15** ($CDCl_3$, 400 MHz)

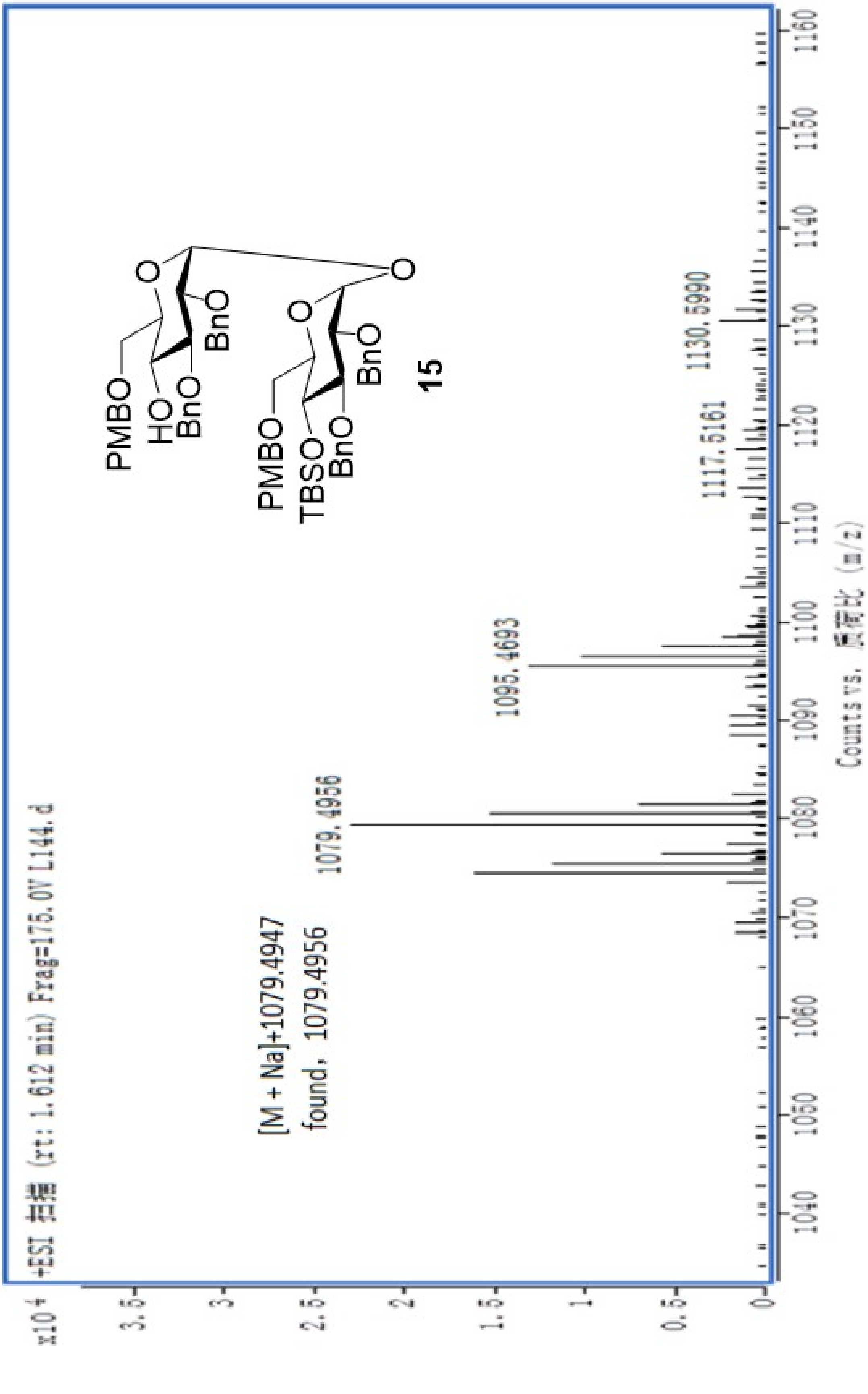

HR-ESI-MS spectrum of conjugate **15**

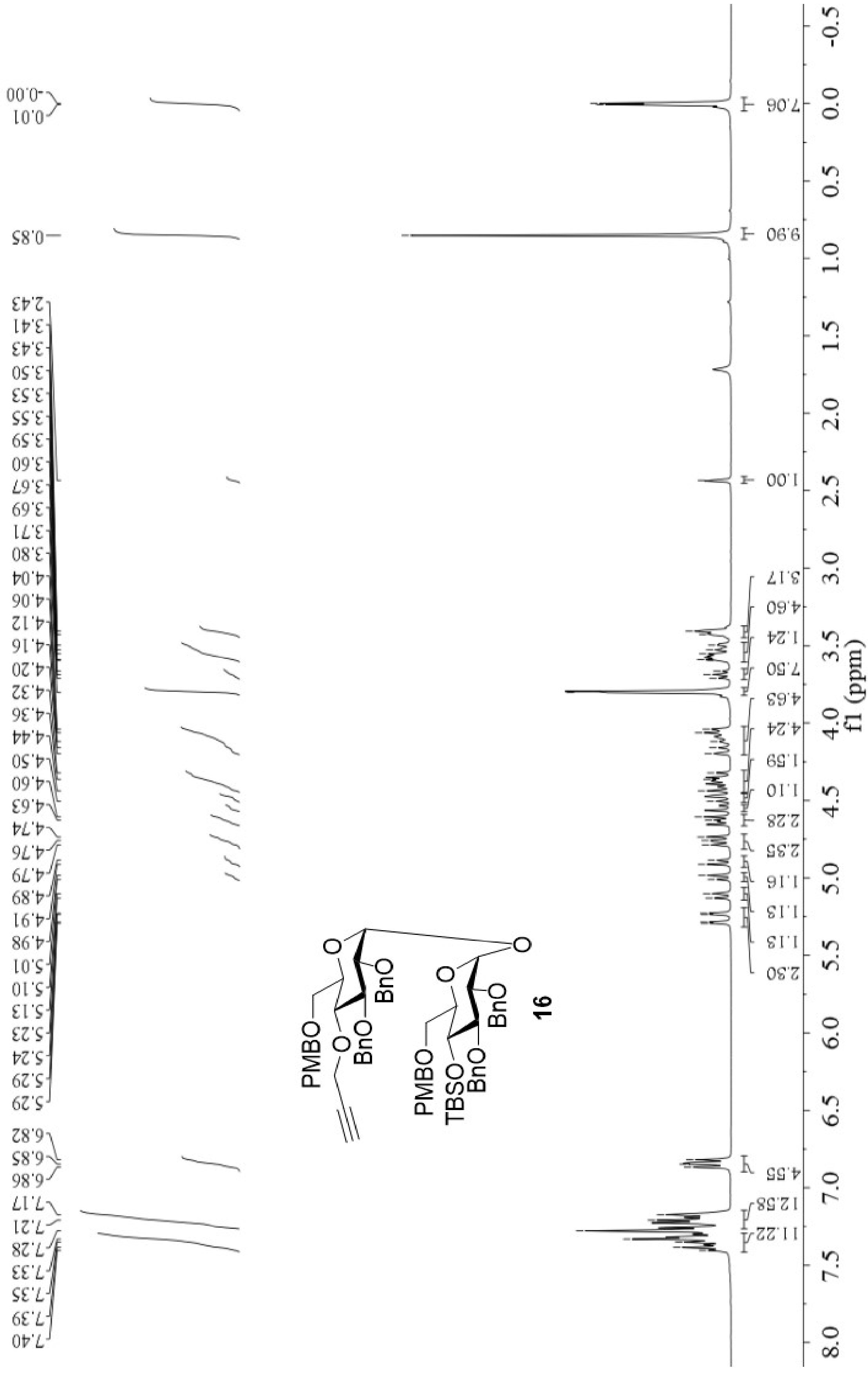

^{1}H NMR Spectrum of compound **16** ($CDCl_3$, 400 MHz)

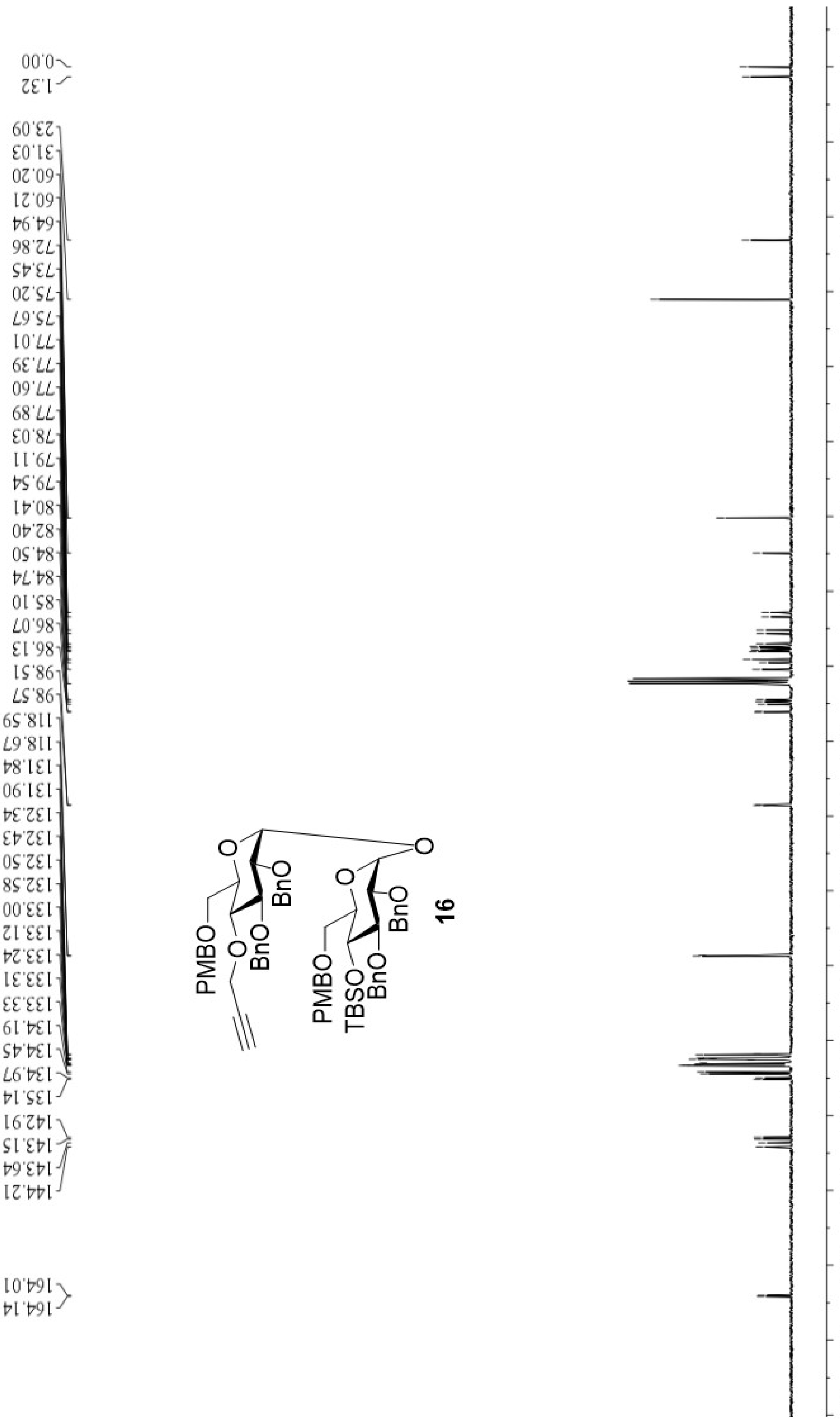

^{13}C NMR Spectrum of compound **16** ($CDCl_3$, 100 MHz)

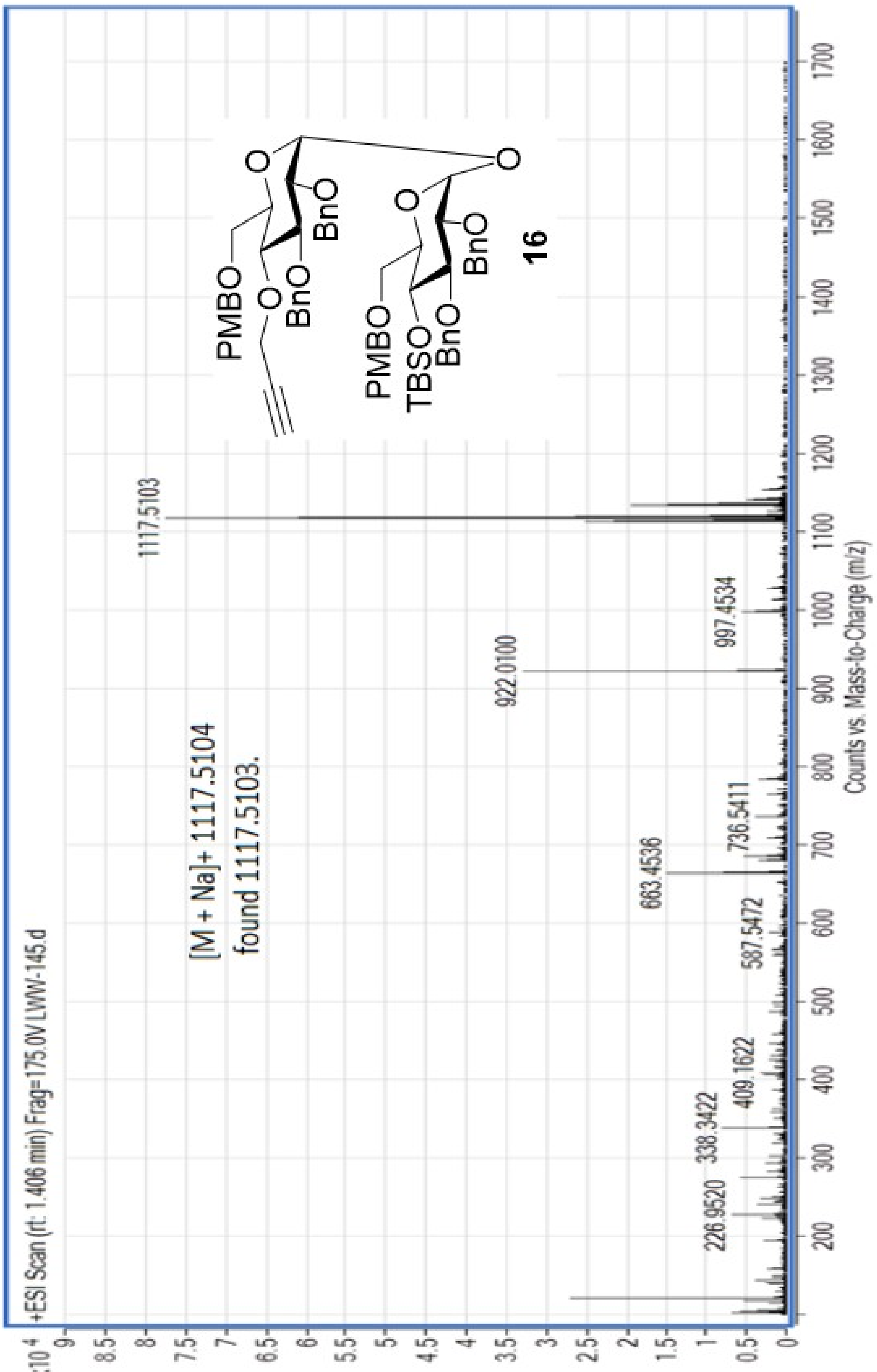

HR-ESI-MS spectrum of conjugate **16**

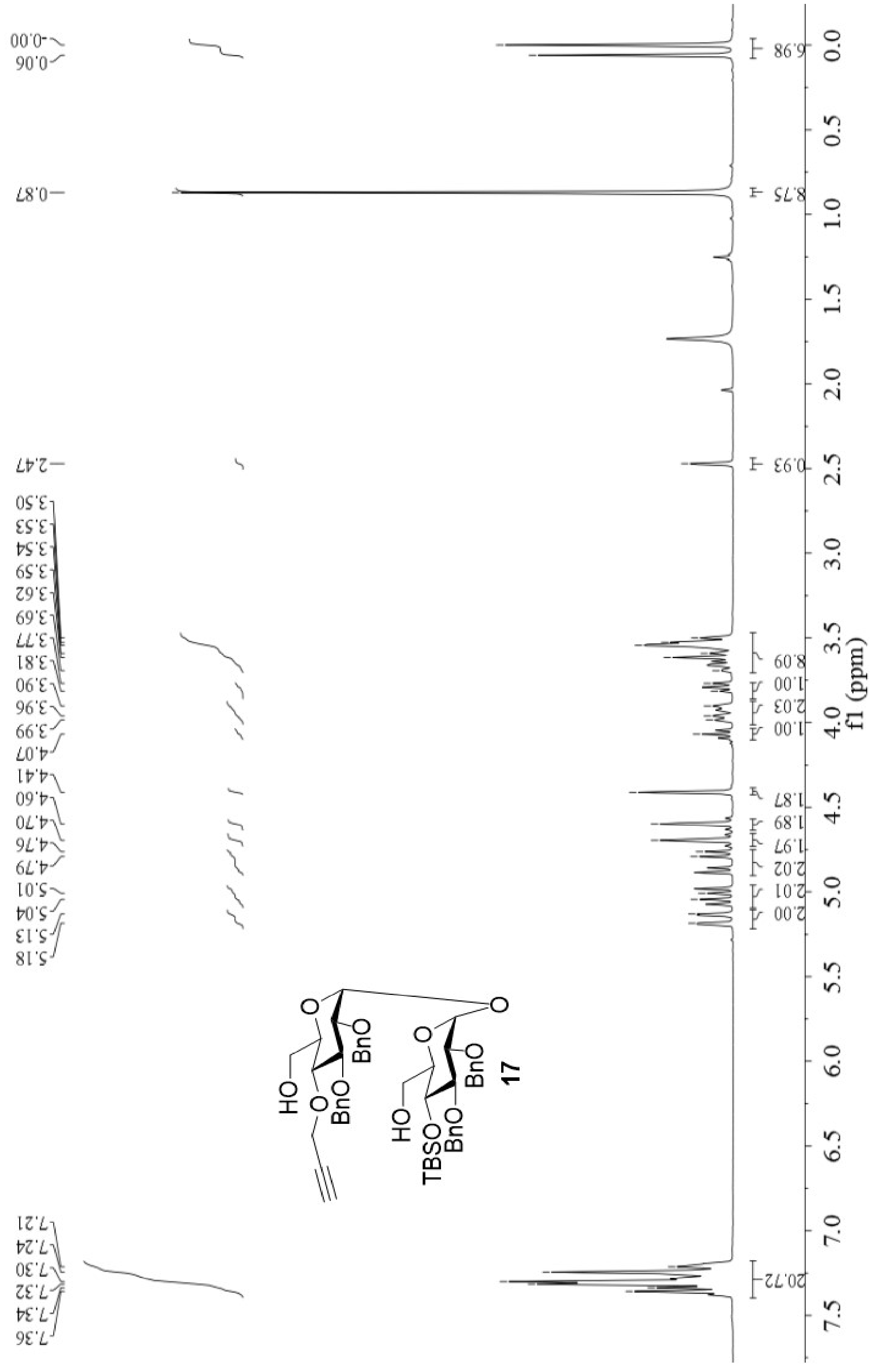

^{1}H NMR Spectrum of compound **17** ($CDCl_3$, 400 MHz)

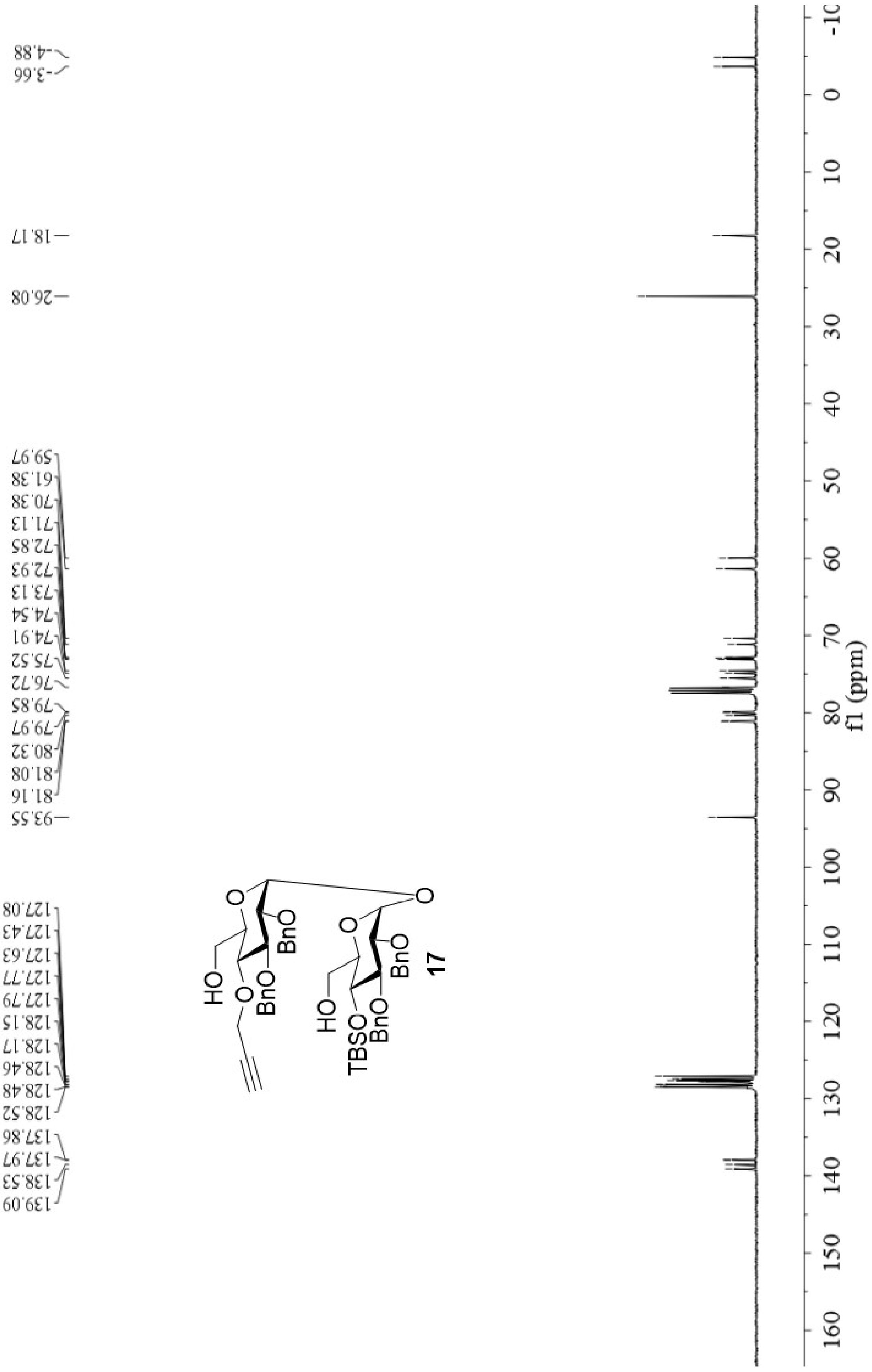

^{13}C NMR Spectrum of compound **17** ($CDCl_3$, 400 MHz)

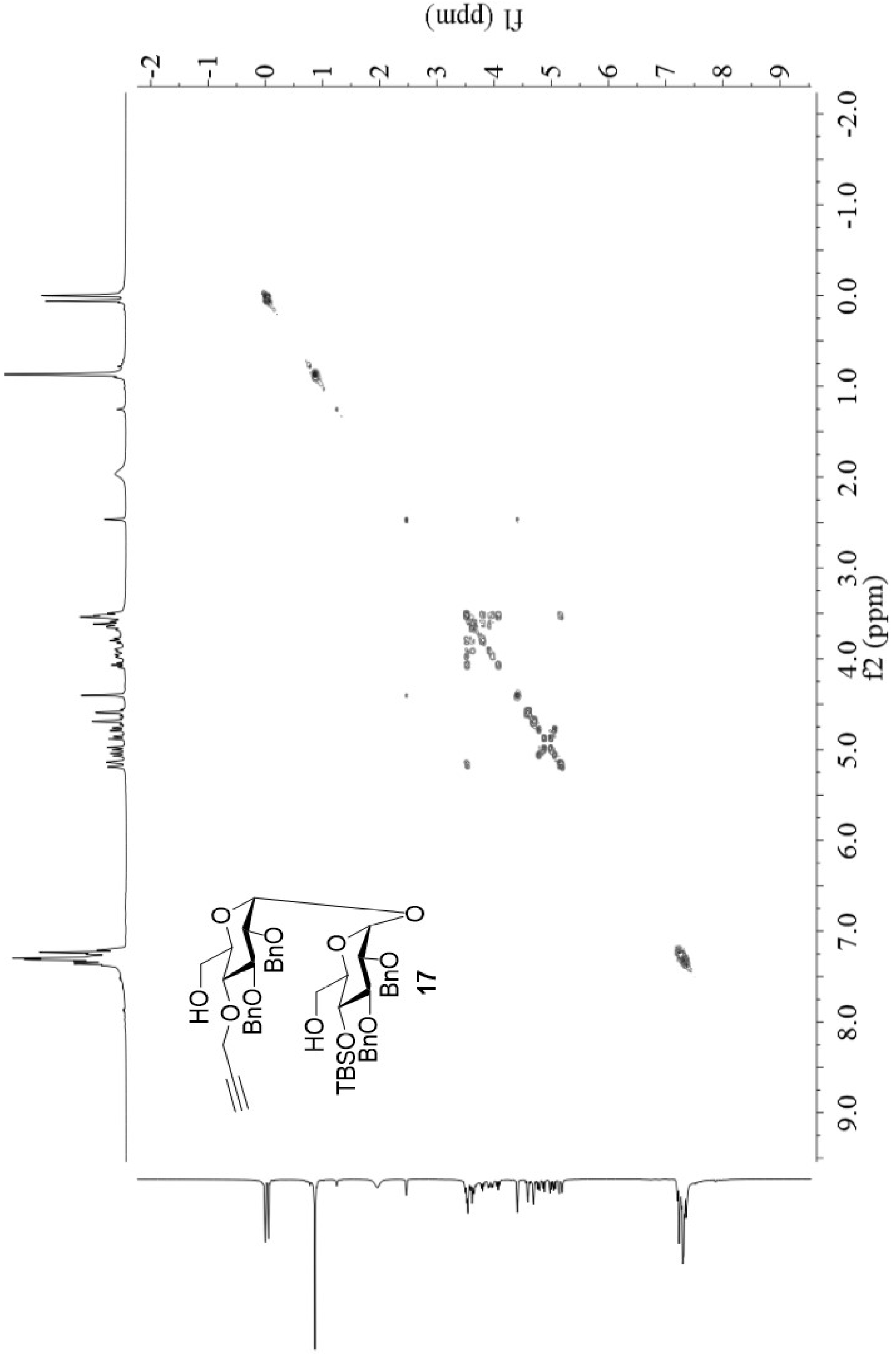

^{1}H-^{1}H COSY NMR Spectrum of compound **17** ($CDCl_3$, 400 MHz)

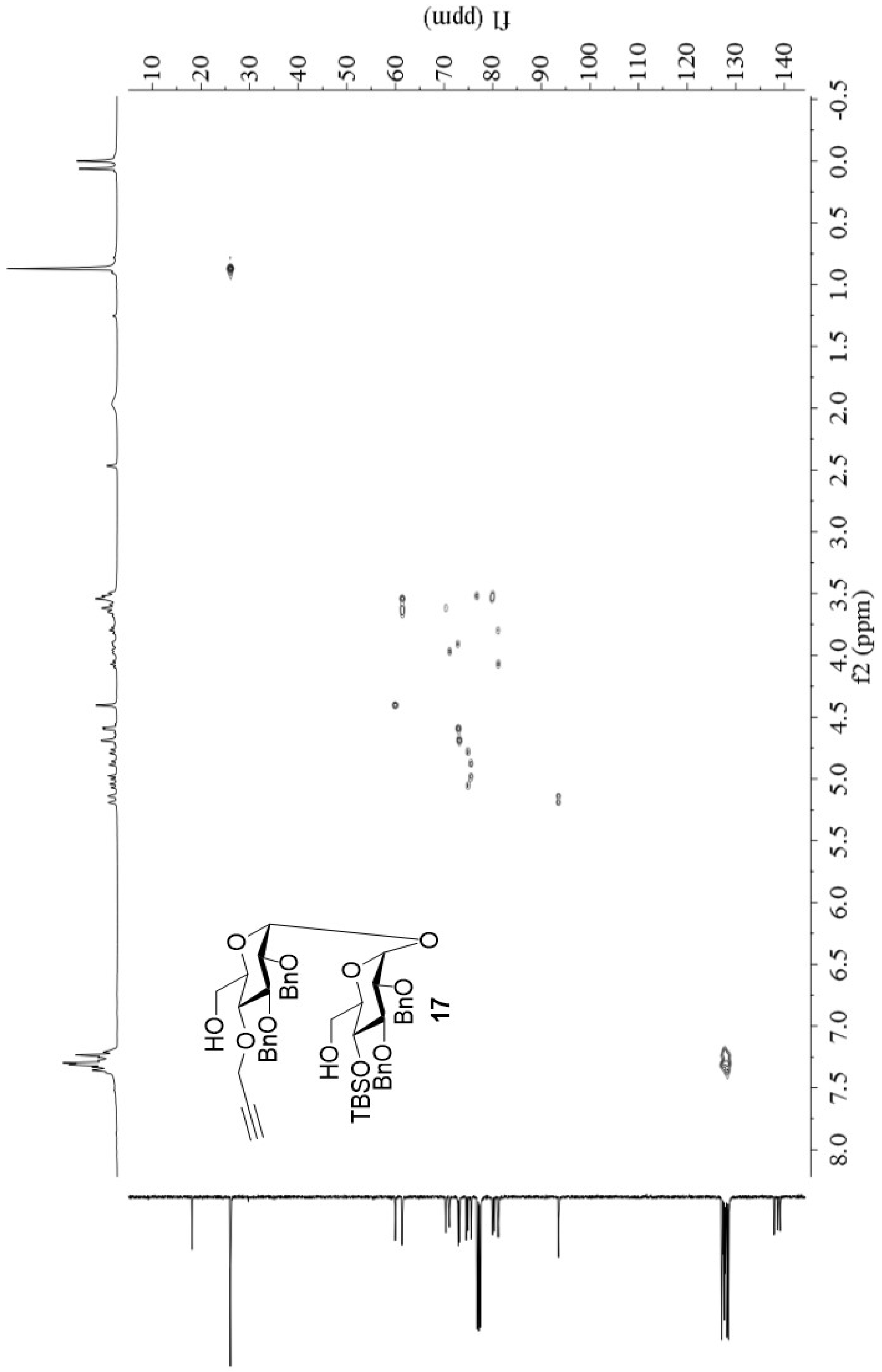

HSQC NMR Spectrum of compound **17** ($CDCl_3$, 400 MHz)

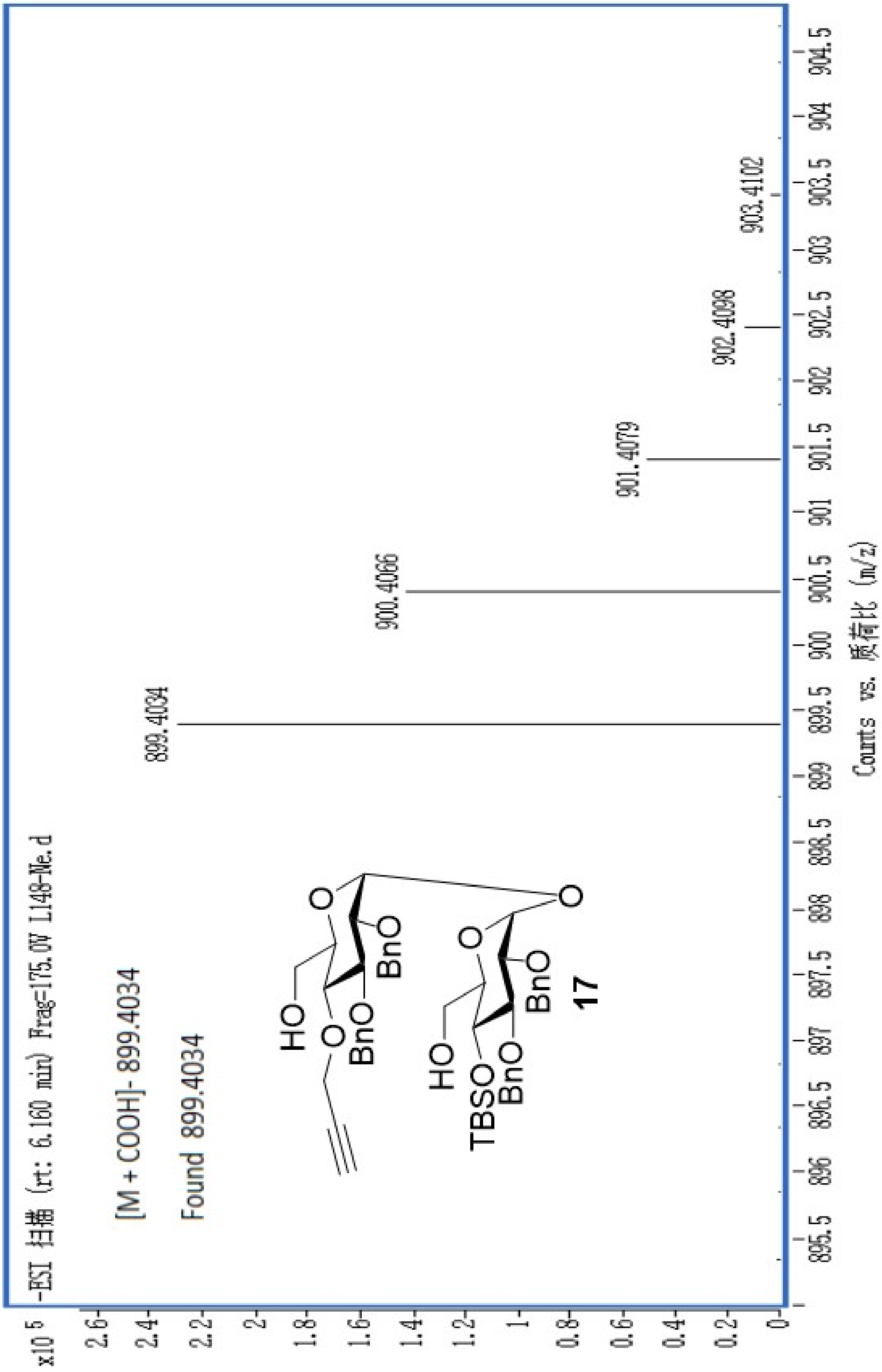

HR-ESI-MS spectrum of conjugate **17**

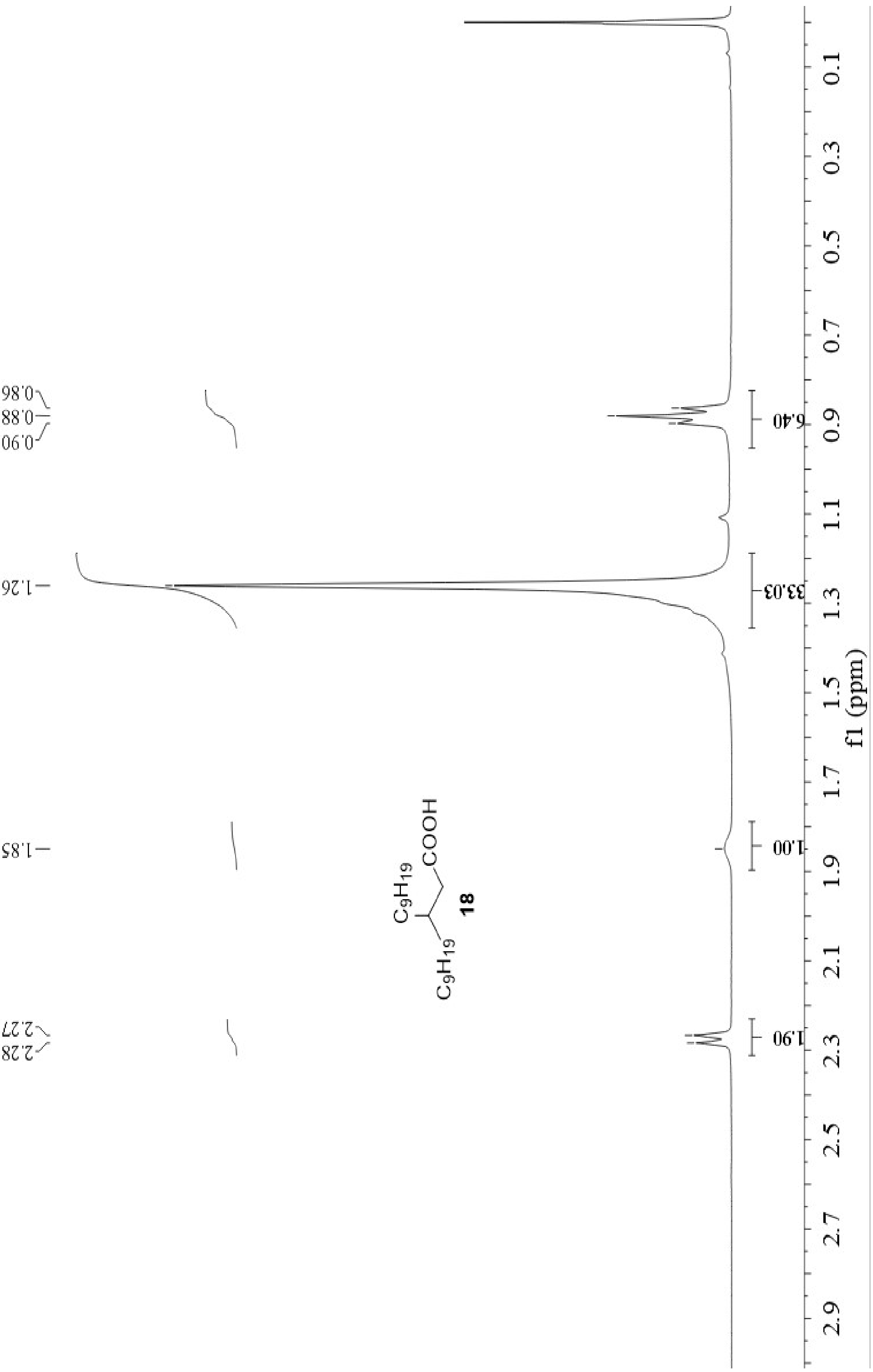

^{1}H NMR Spectrum of compound **18** ($CDCl_3$, 400 MHz)

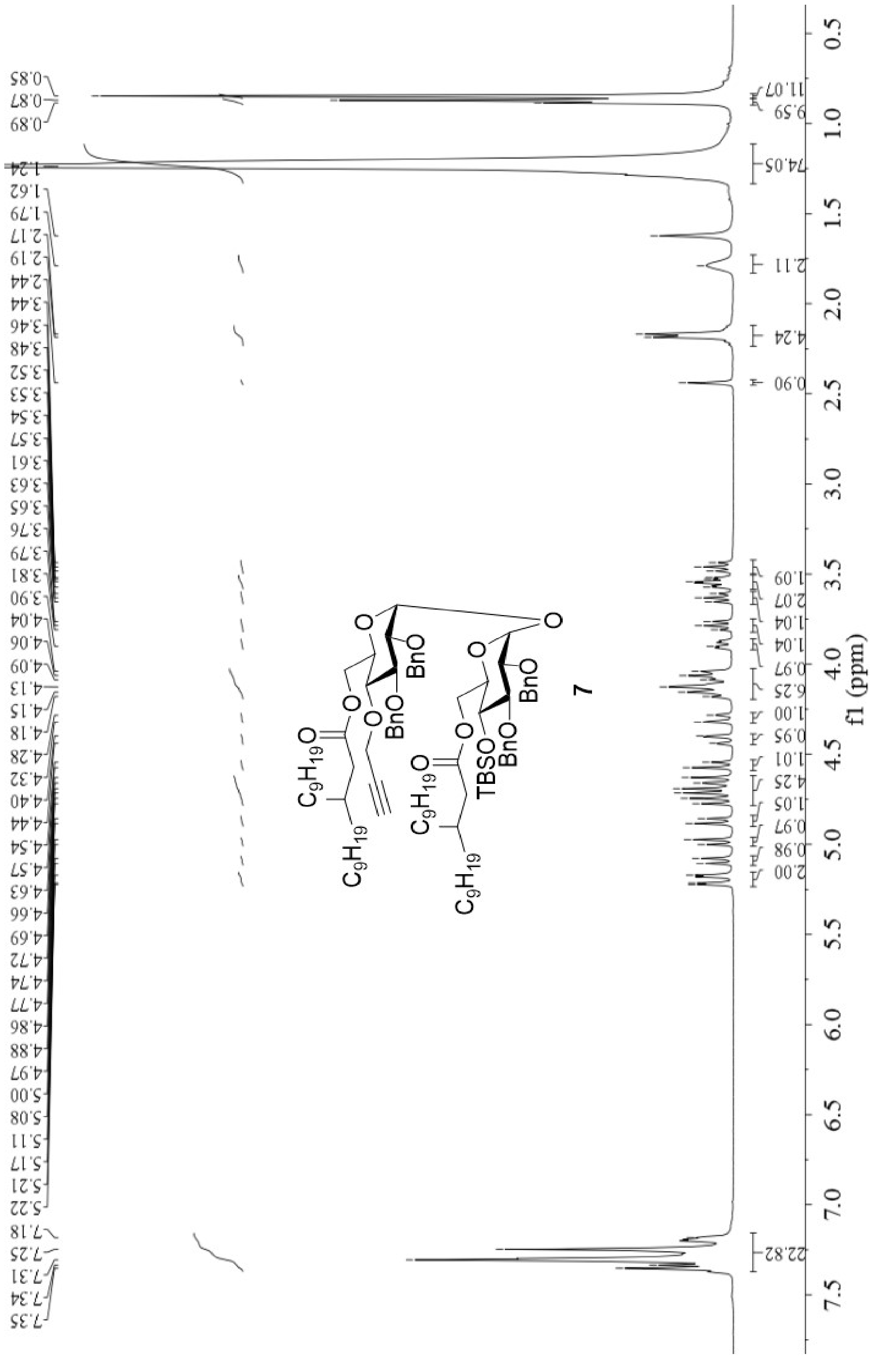

^{1}H NMR Spectrum of compound **7** ($CDCl_3$, 400 MHz)

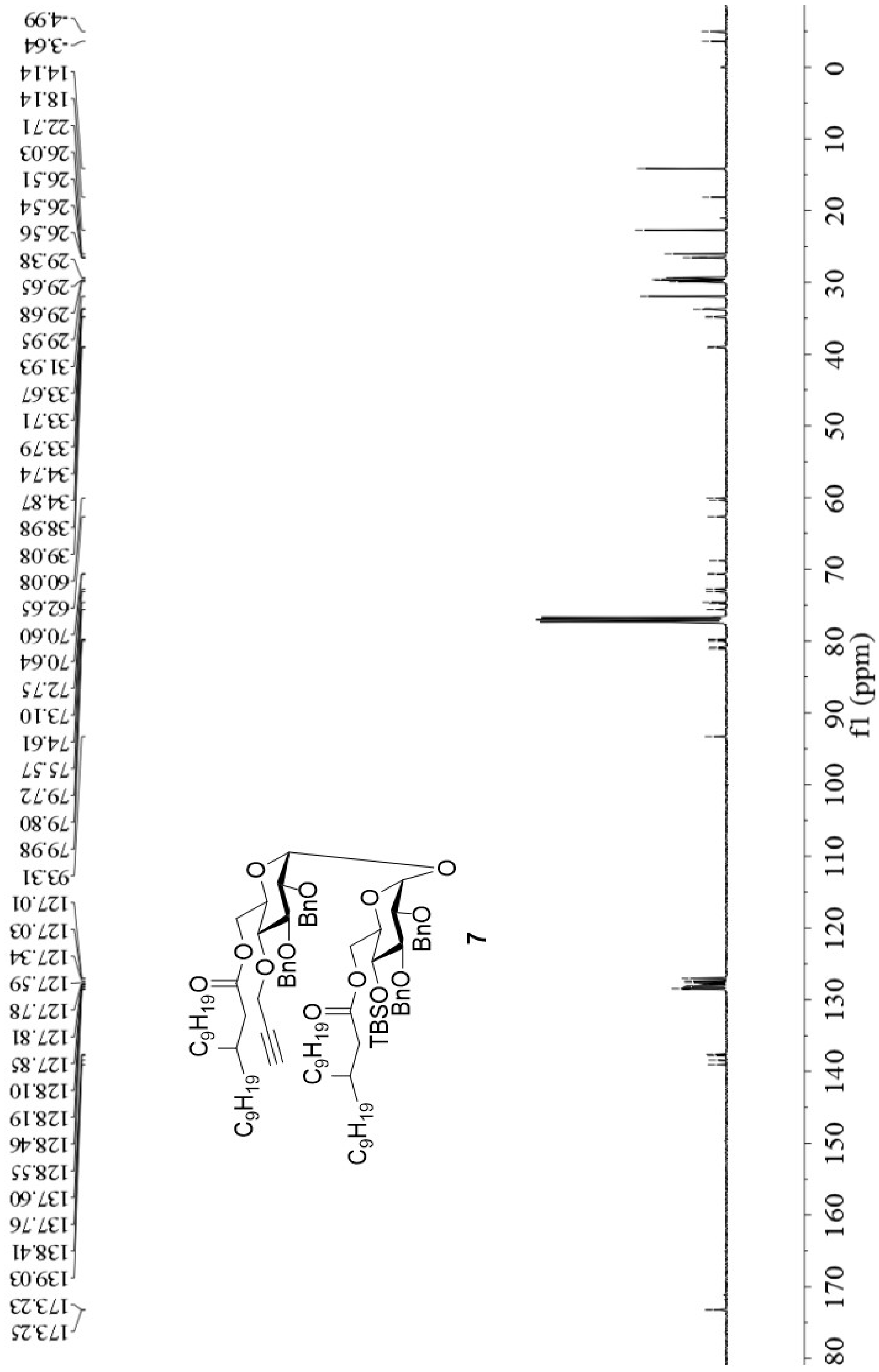

^{13}C NMR Spectrum of compound **7** ($CDCl_3$, 400 MHz)

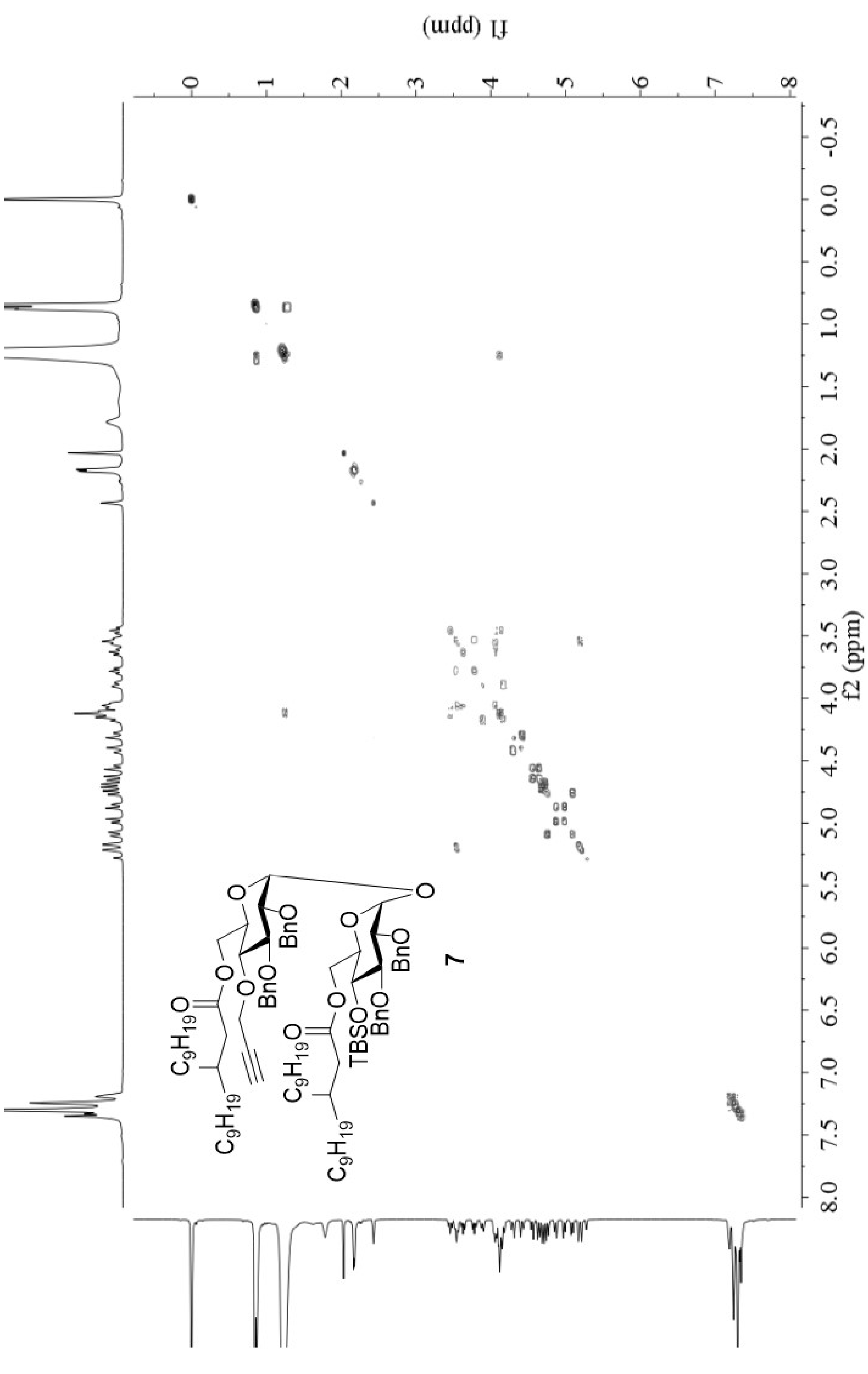

^{1}H- ^{1}H COSY Spectrum of compound **7** ($CDCl_3$,400 MHz)

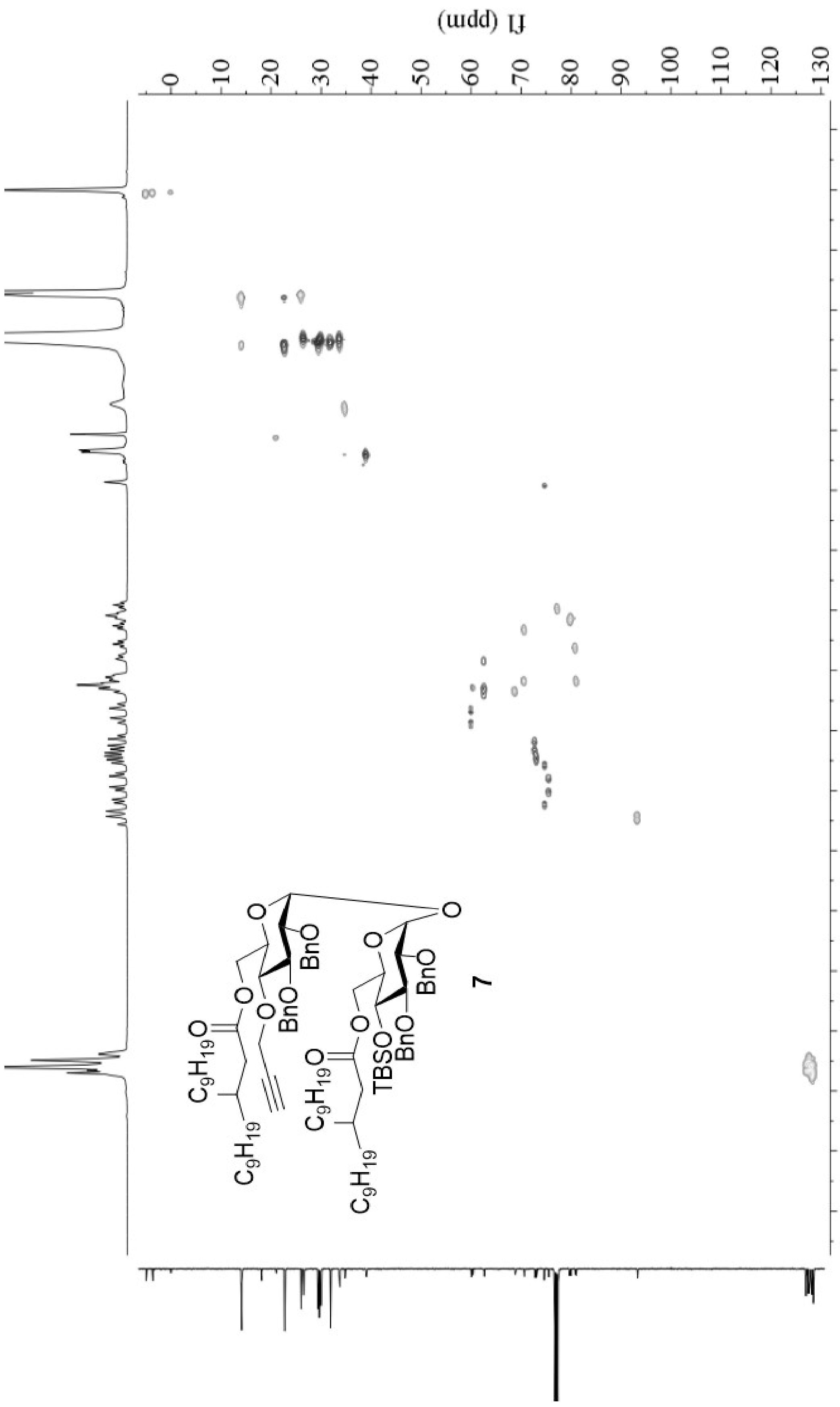

HSQC Spectrum of compound **7** ($CDCl_3$, 400 MHz)

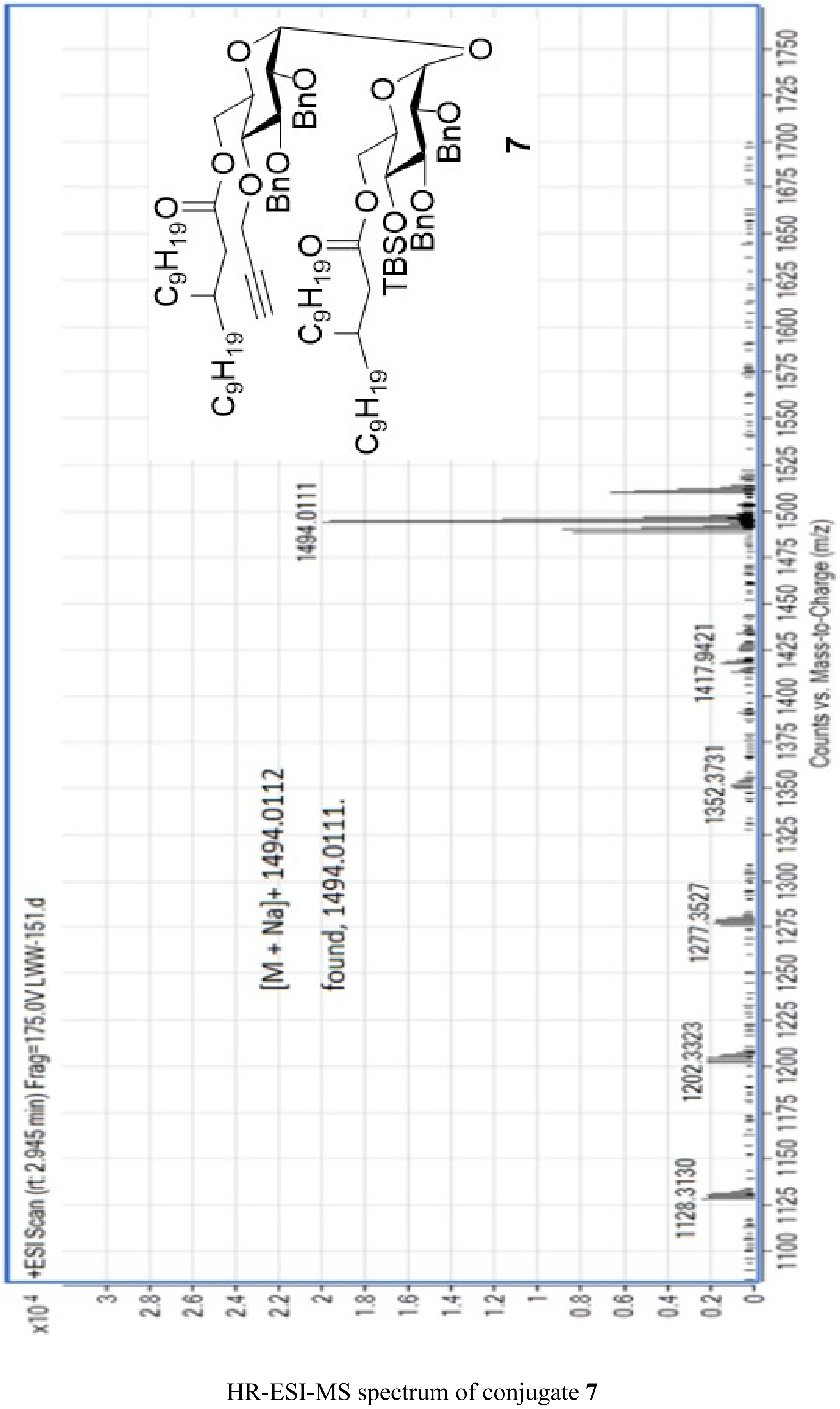

HR-ESI-MS spectrum of conjugate **7**

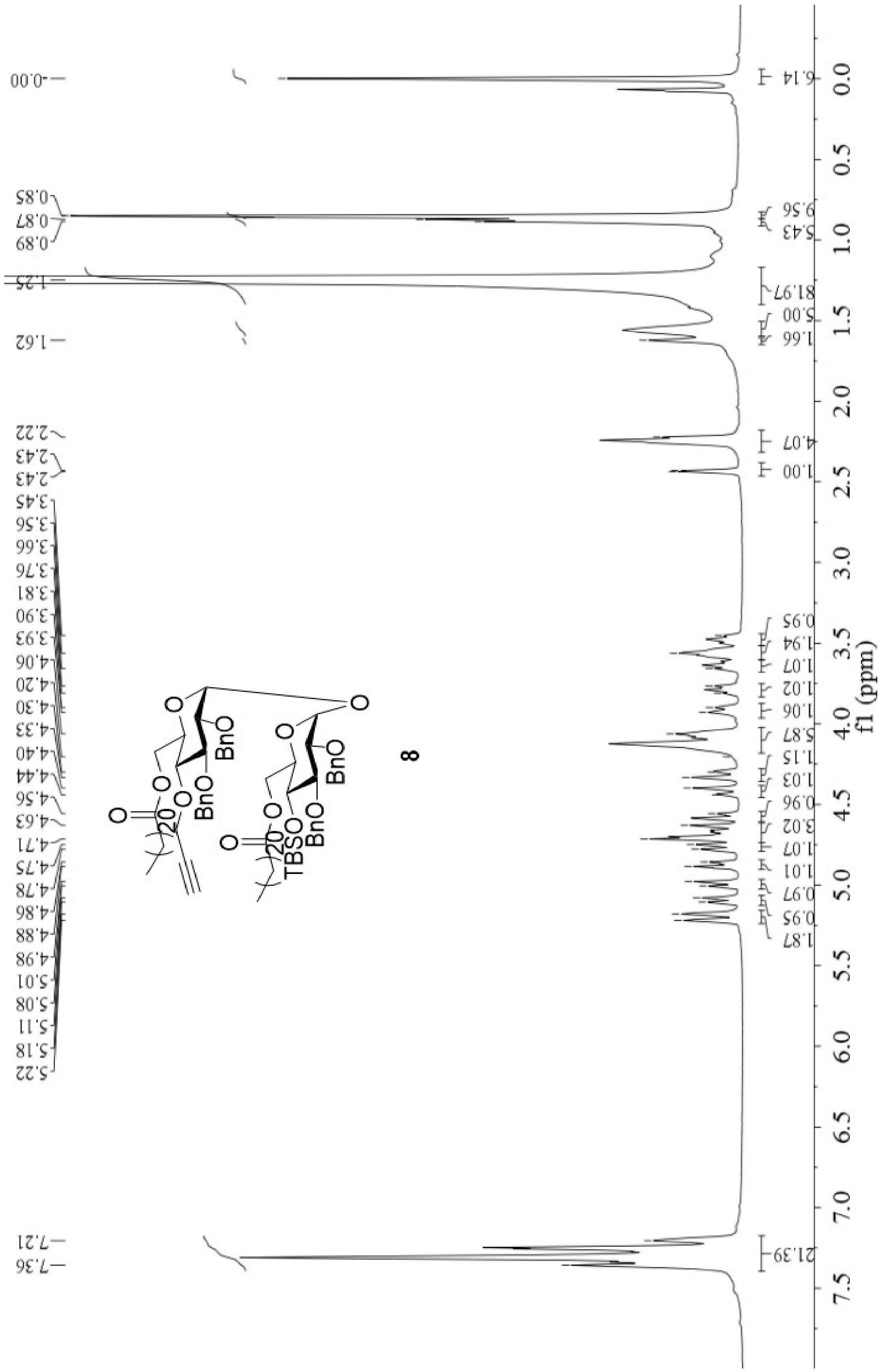

^{1}H NMR Spectrum of compound **8** ($CDCl_3$, 400 MHz)

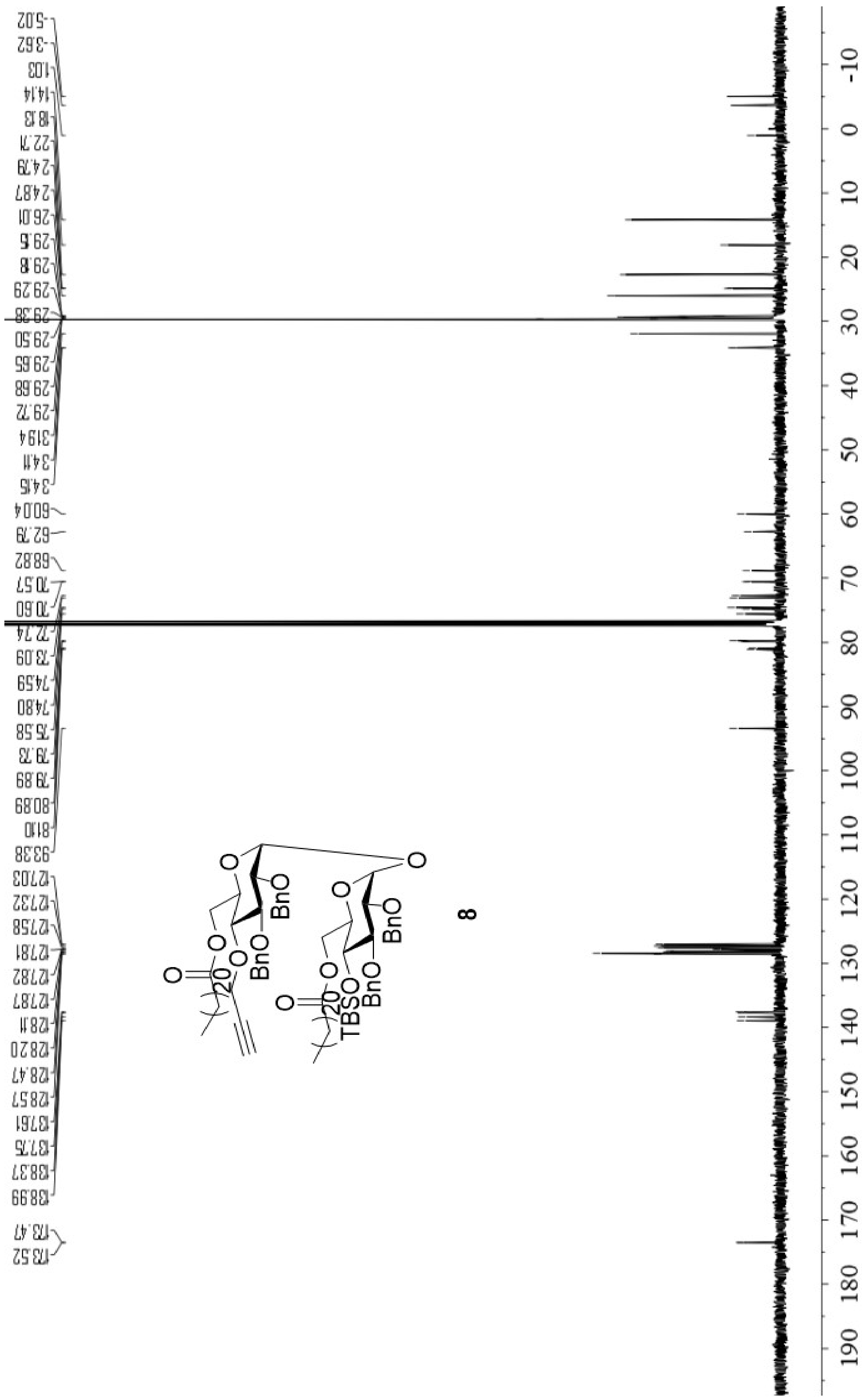

^{13}C NMR Spectrum of compound **8** ($CDCl_3$, 400 MHz)

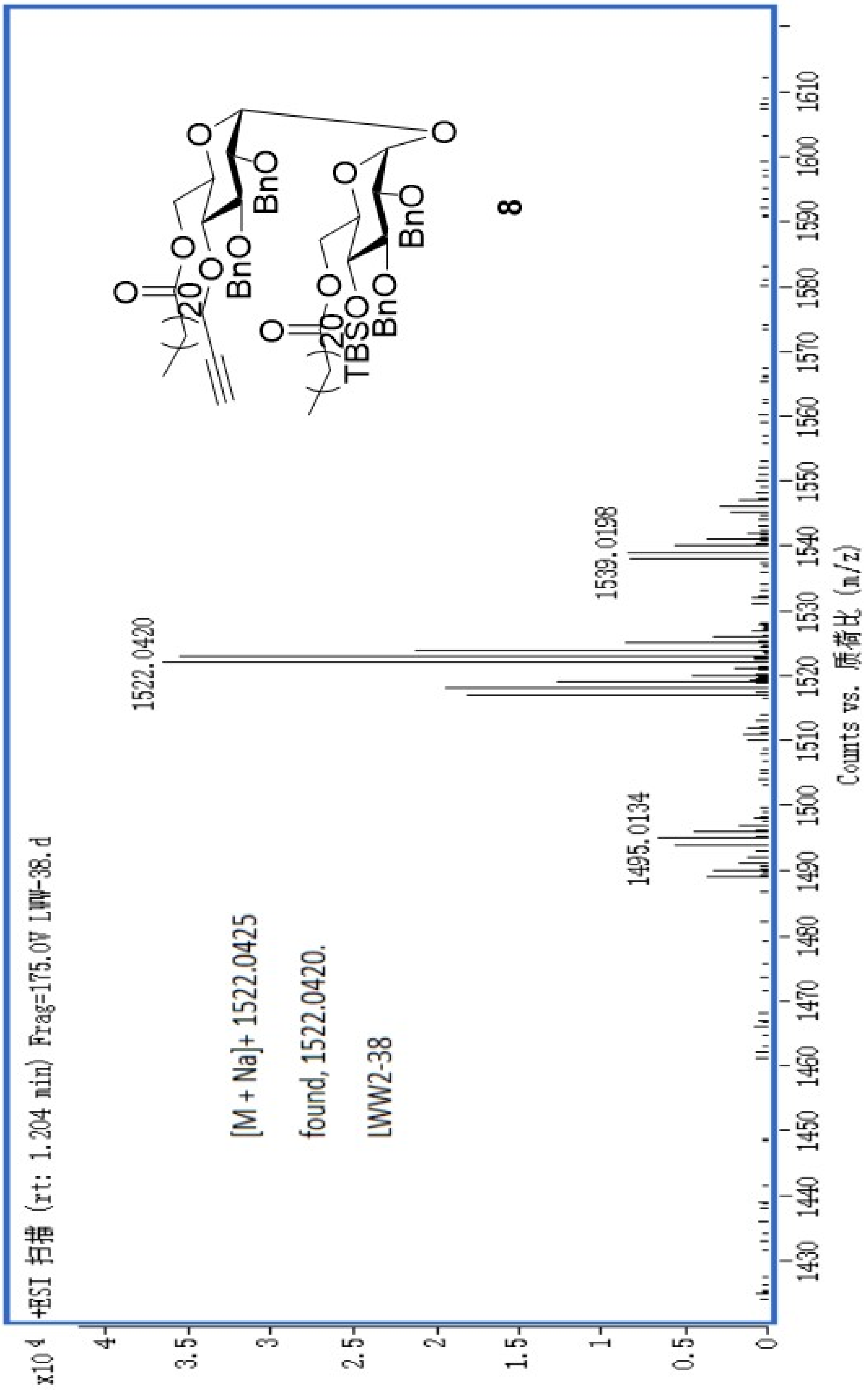

HR-ESI-MS spectrum of conjugate **8**

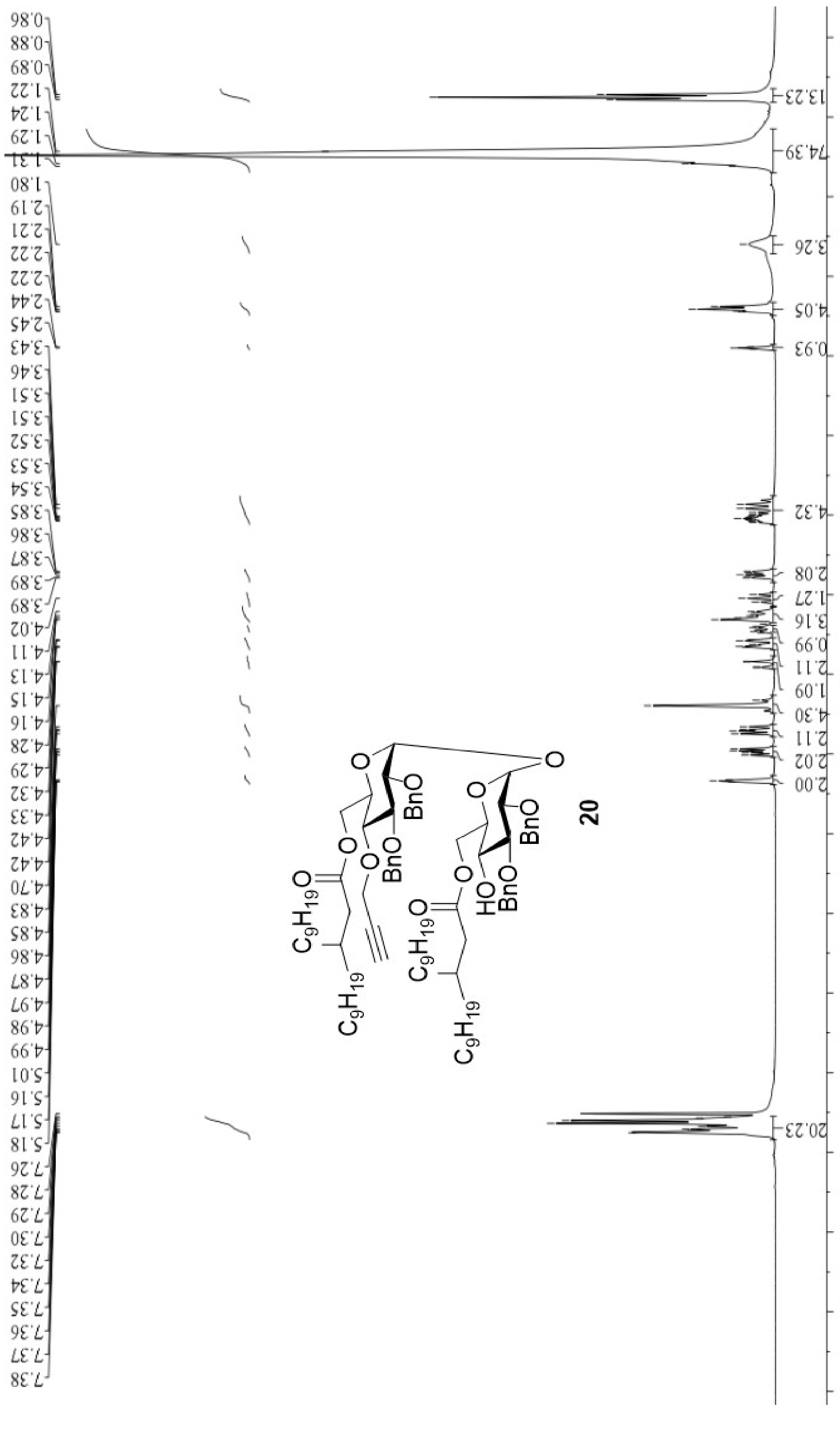

^{1}H NMR Spectrum of compound **20** ($CDCl_3$, 400 MHz)

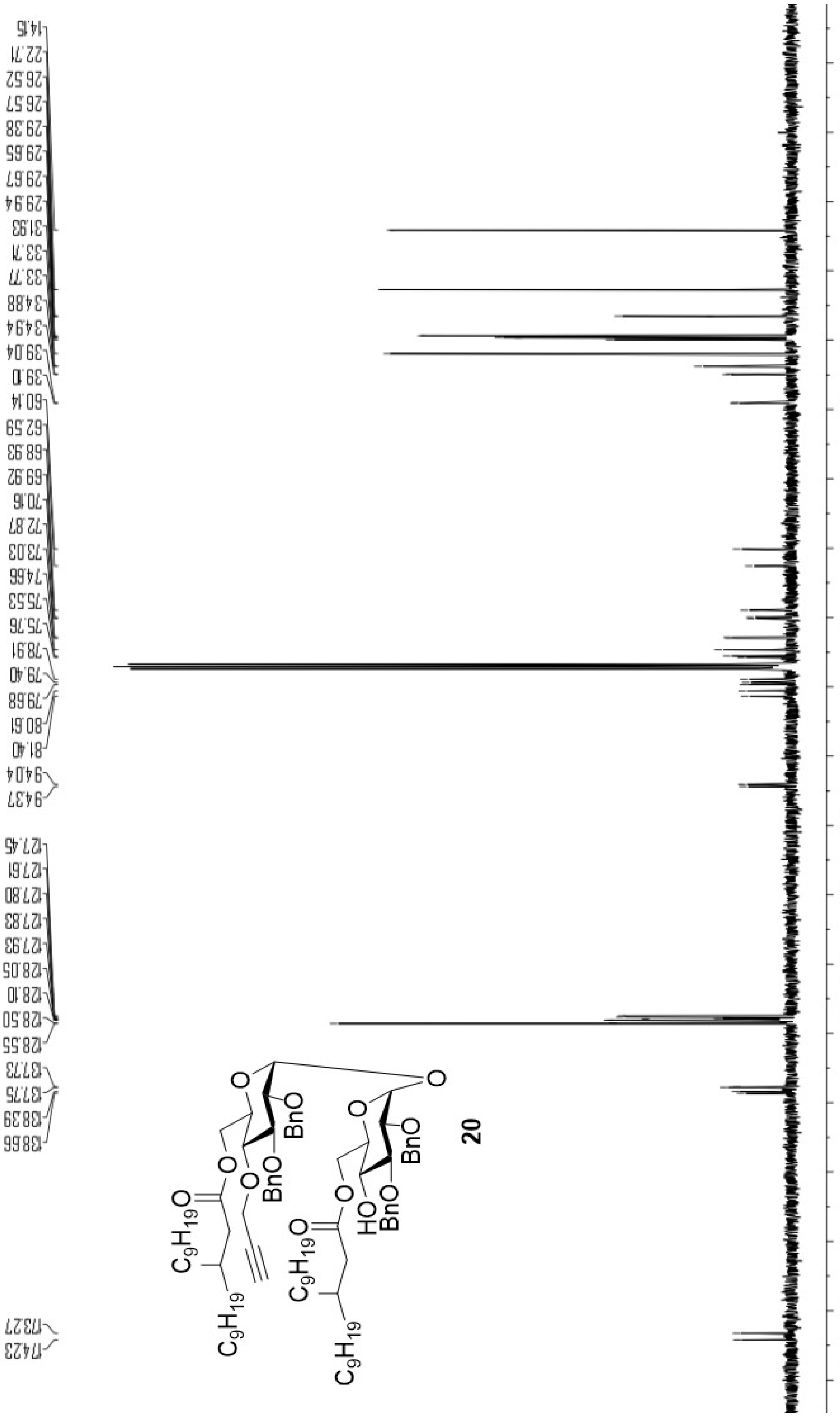

^{13}C NMR Spectrum of compound **20** (CDCl$_3$, 400 MHz)

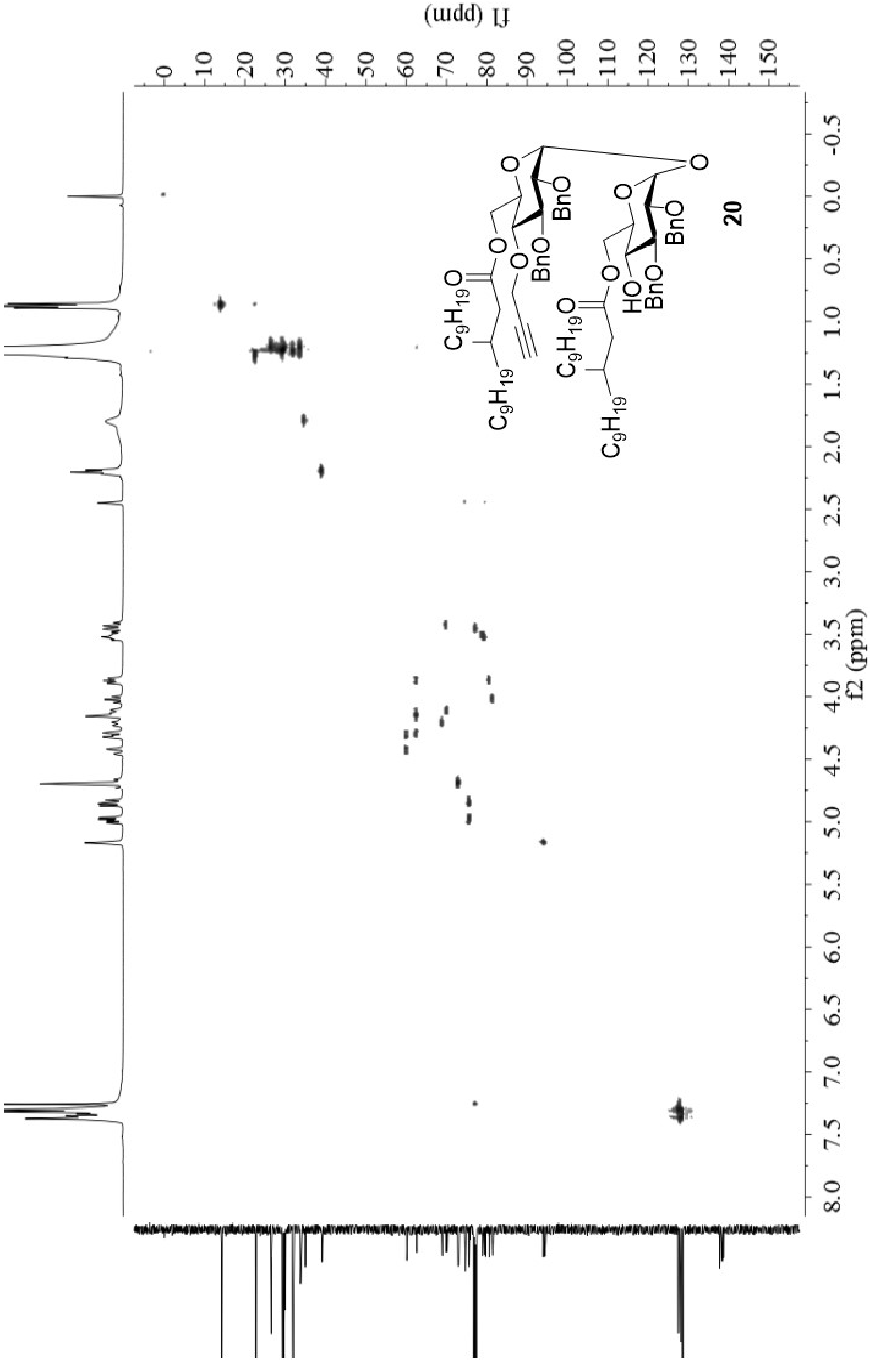

HSQC NMR Spectrum of compound **20** ($CDCl_3$, 400 MHz)

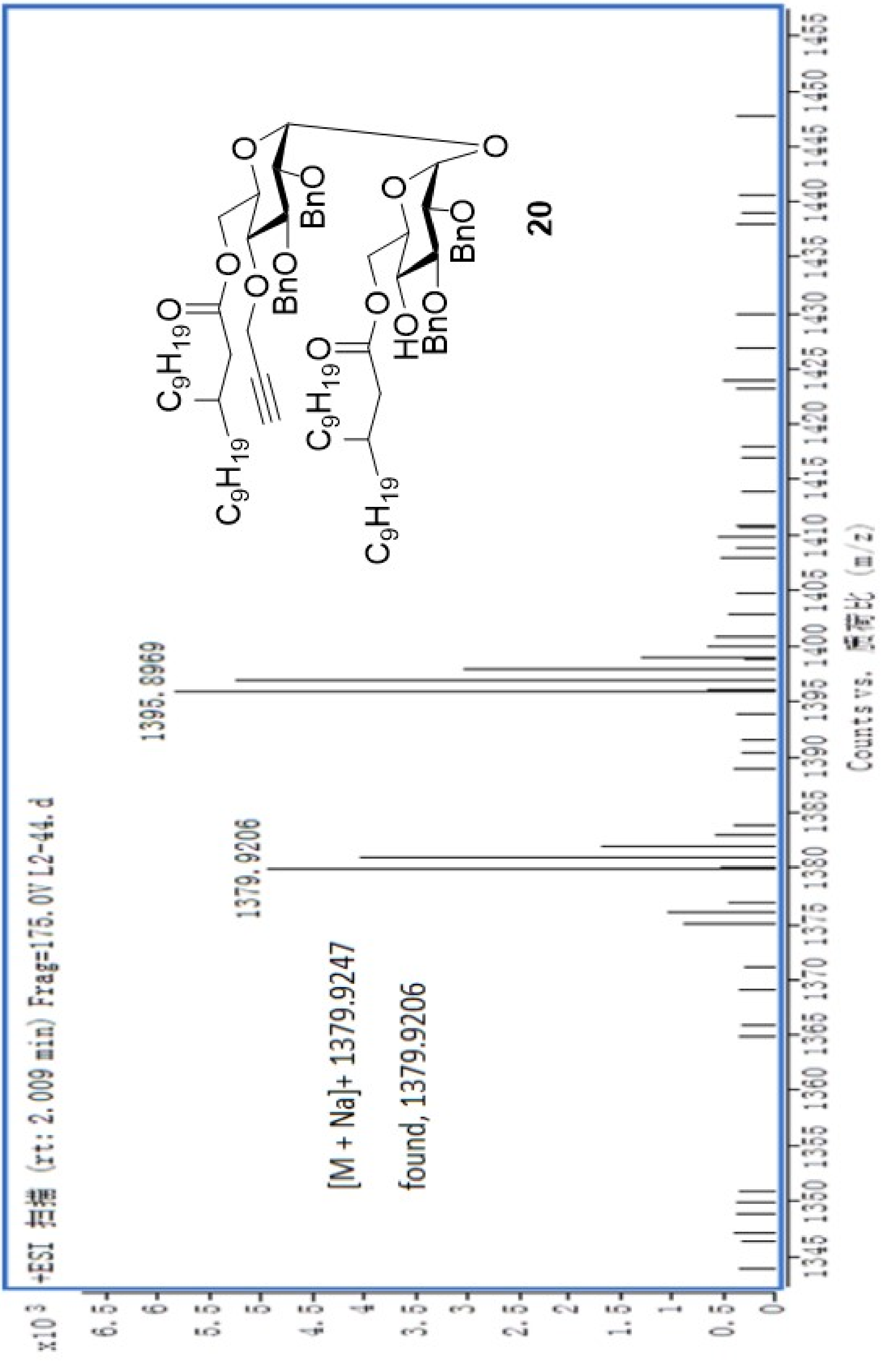

HR-ESI-MS spectrum of conjugate **20**

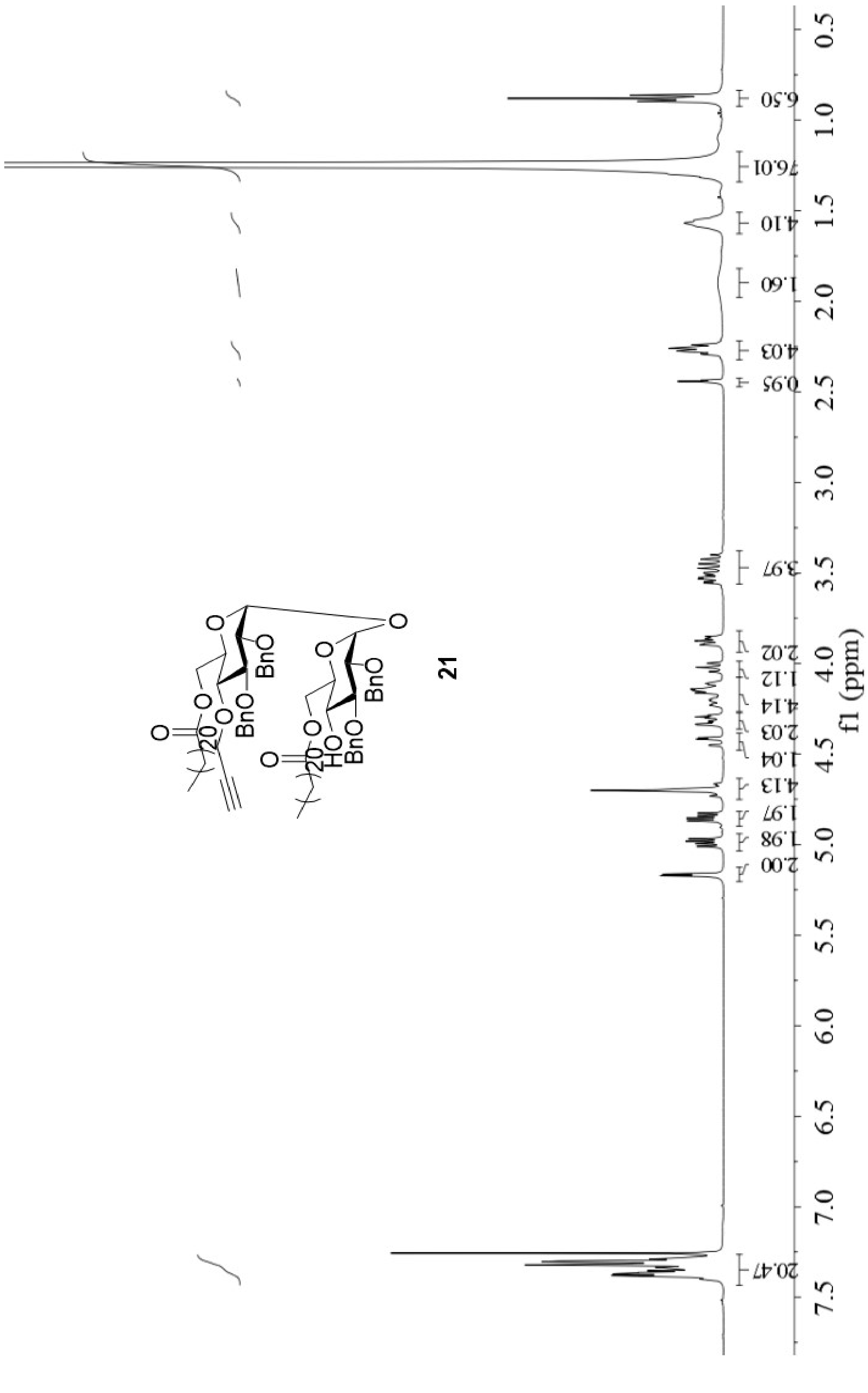

^{1}H NMR Spectrum of compound **21** ($CDCl_3$, 400 MHz)

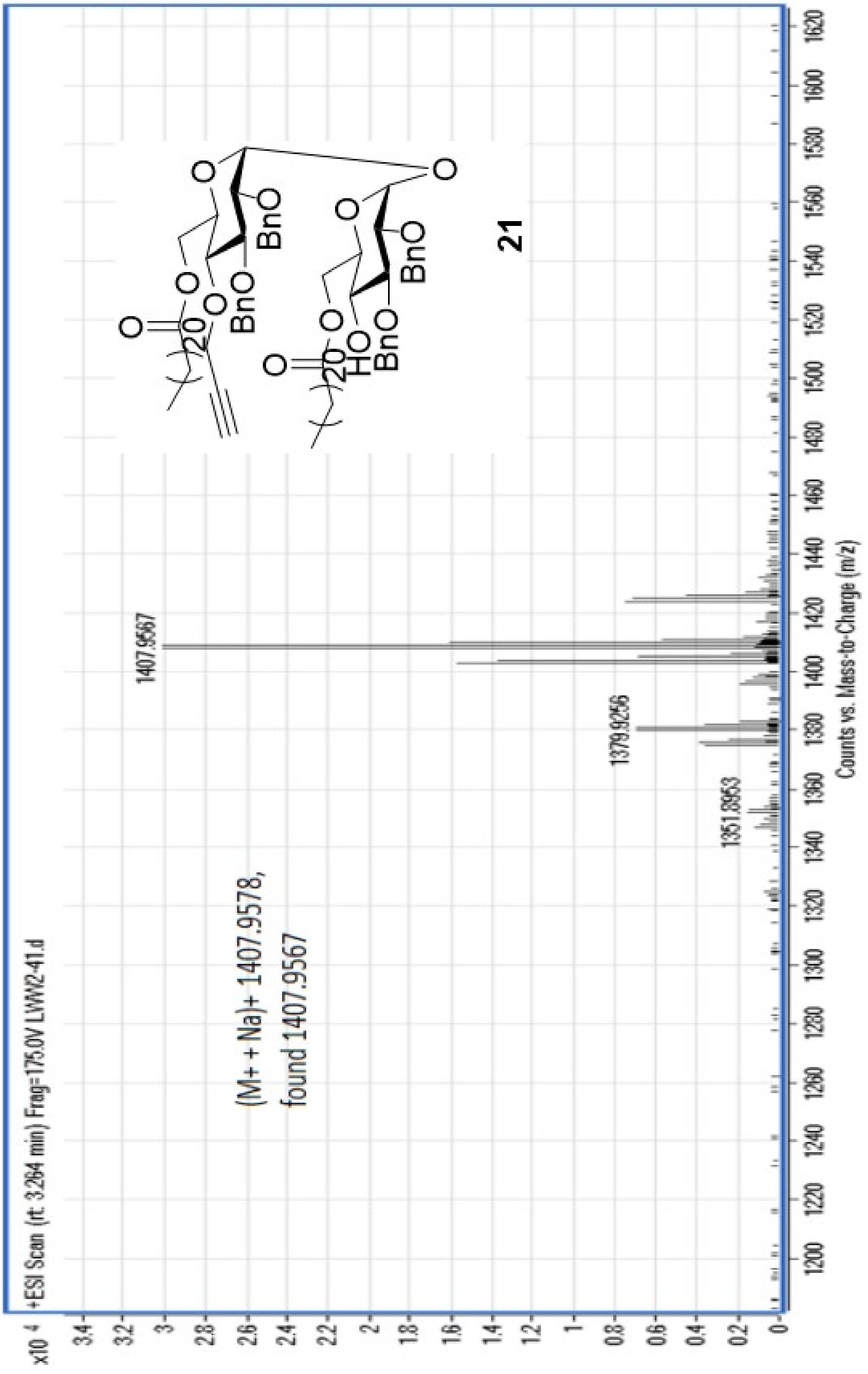

HR-ESI-MS spectrum of conjugate **21**

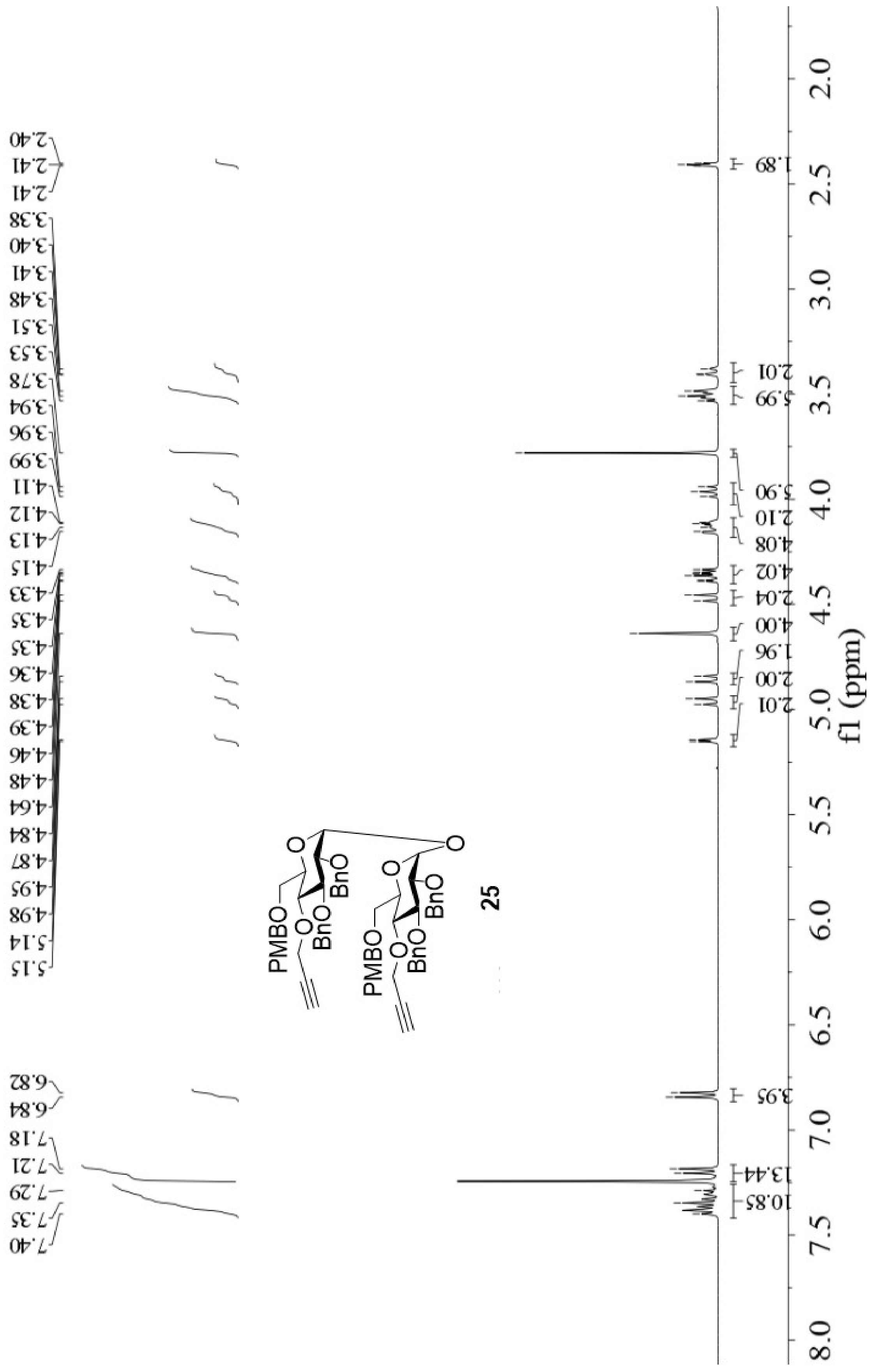

^{1}H NMR Spectrum of compound **25** ($CDCl_3$, 400 MHz)

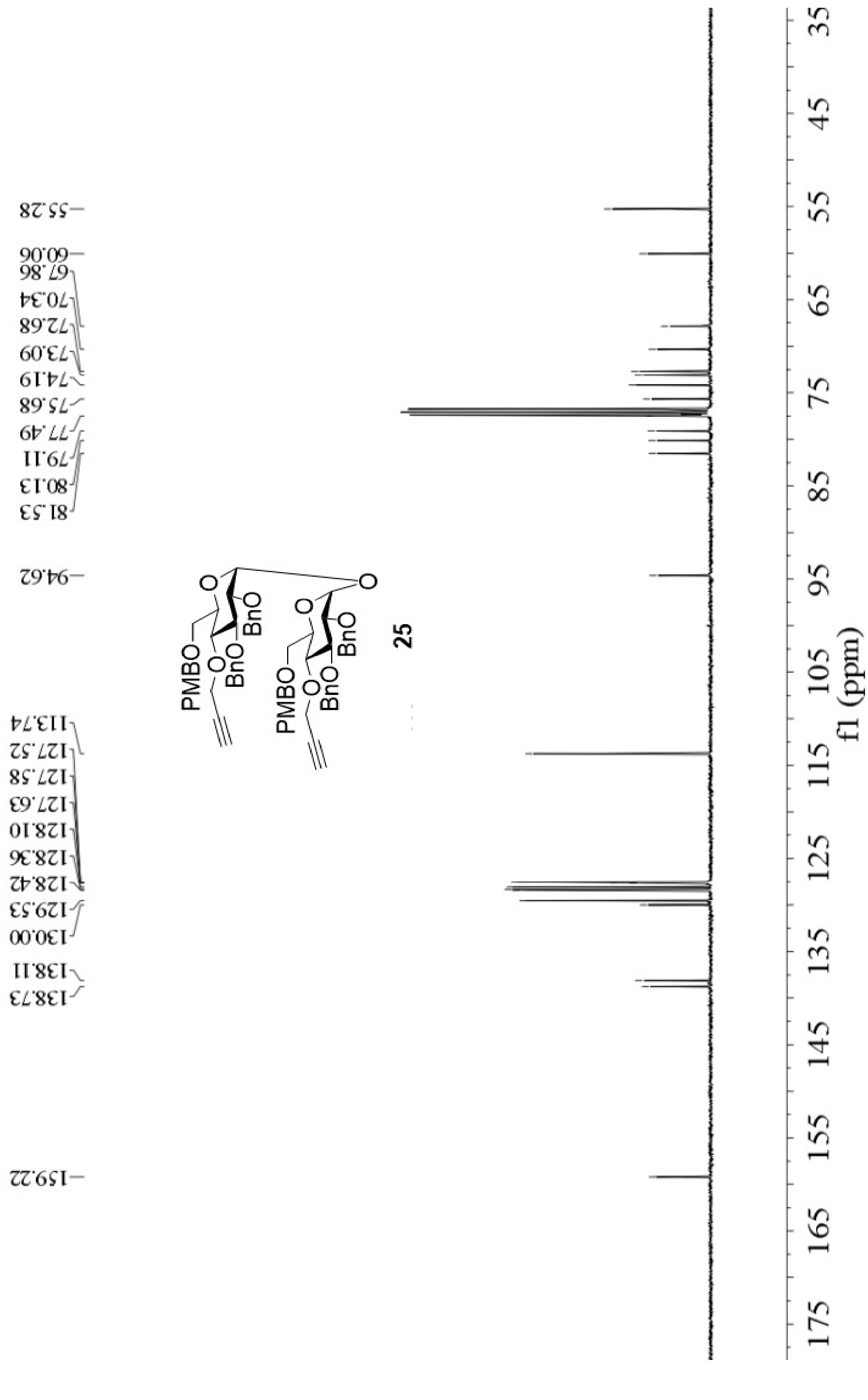

^{13}C NMR Spectrum of compound **25** ($CDCl_3$, 400 MHz)

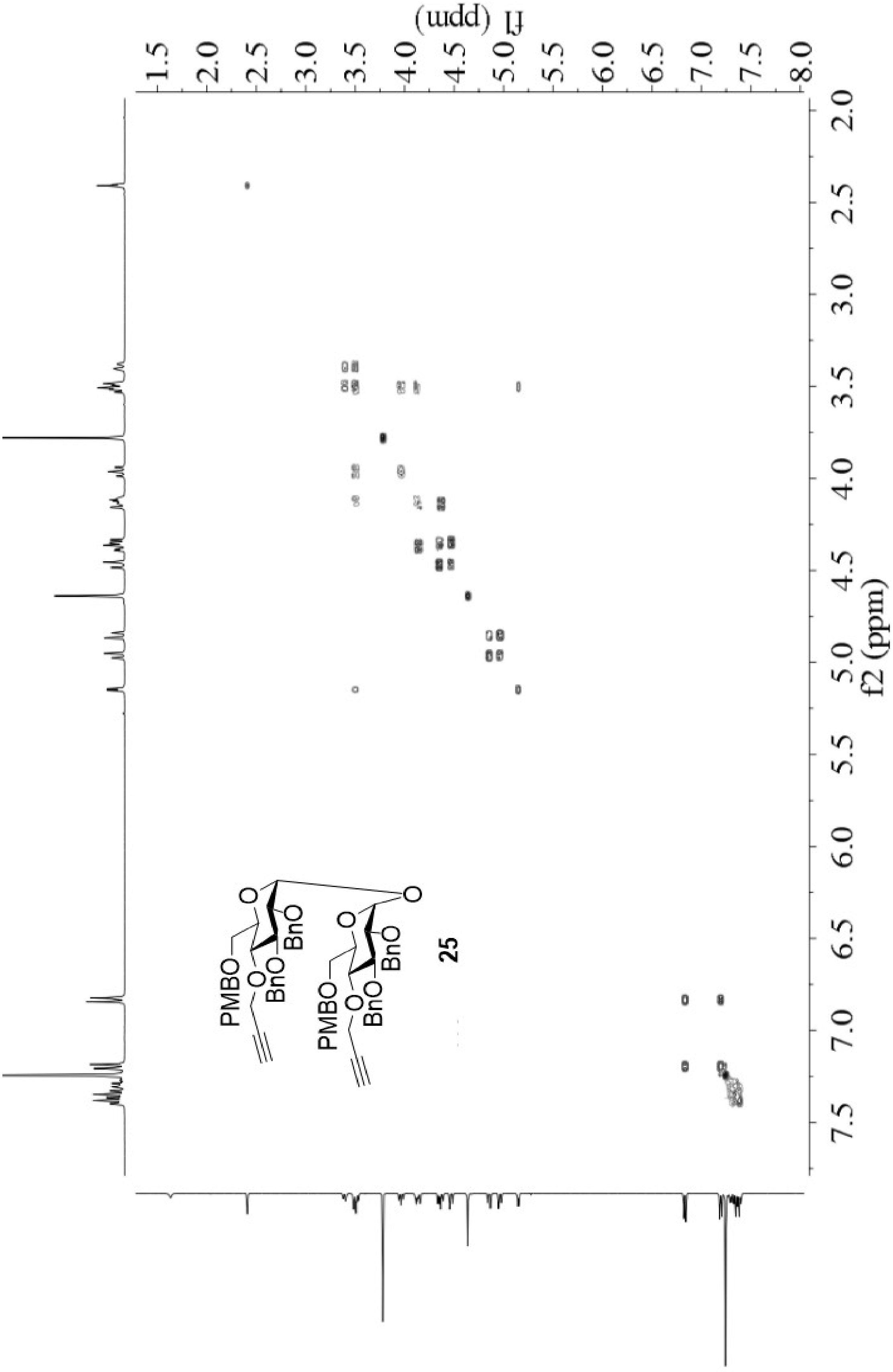

^{1}H-^{1}H COSY NMR Spectrum of compound **25** ($CDCl_3$, 400 MHz)

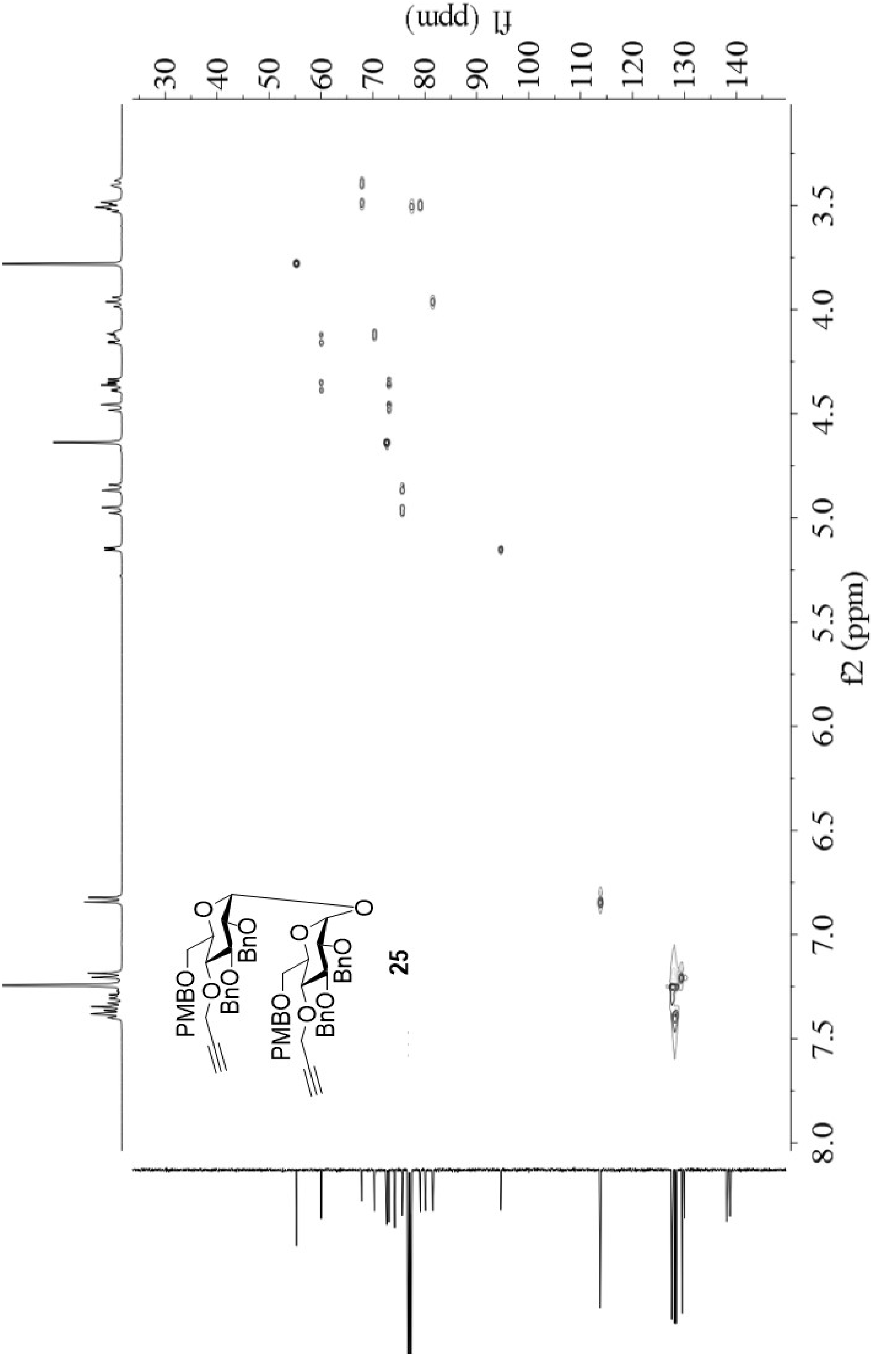

HSQC NMR Spectrum of compound **25** ($CDCl_3$, 400 MHz)

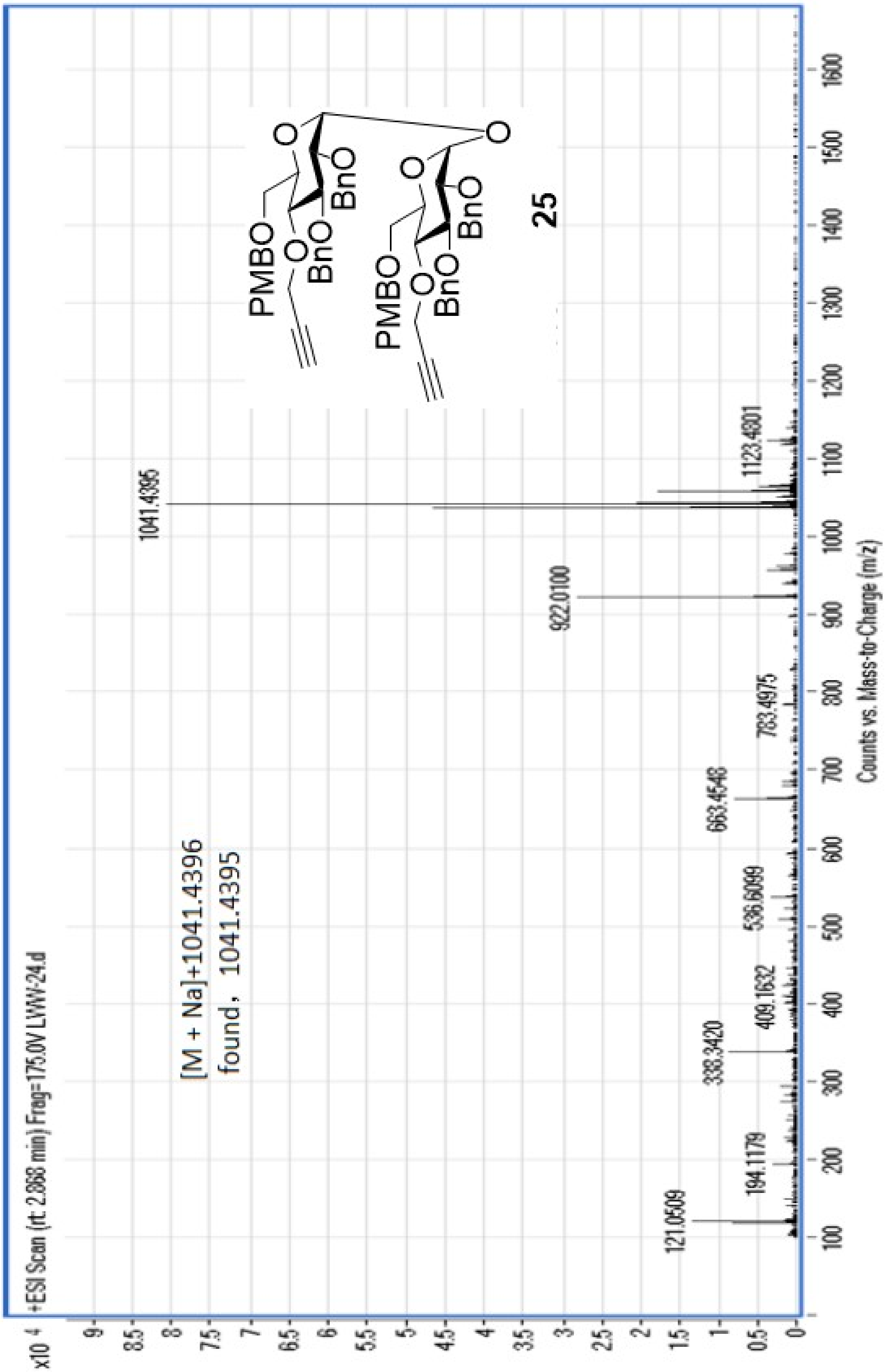

HR-ESI-MS spectrum of conjugate **25**

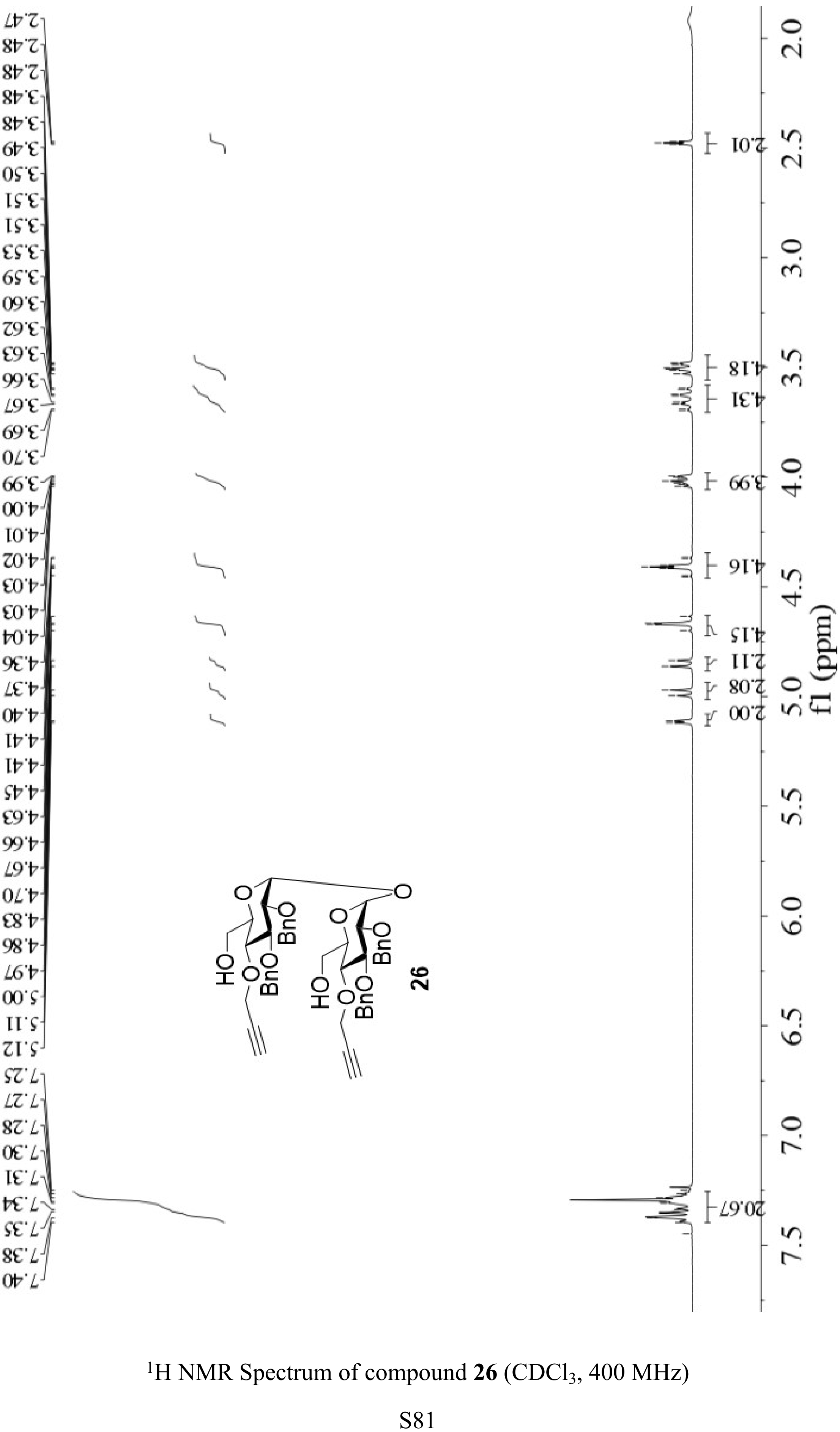

^{1}H NMR Spectrum of compound **26** ($CDCl_3$, 400 MHz)

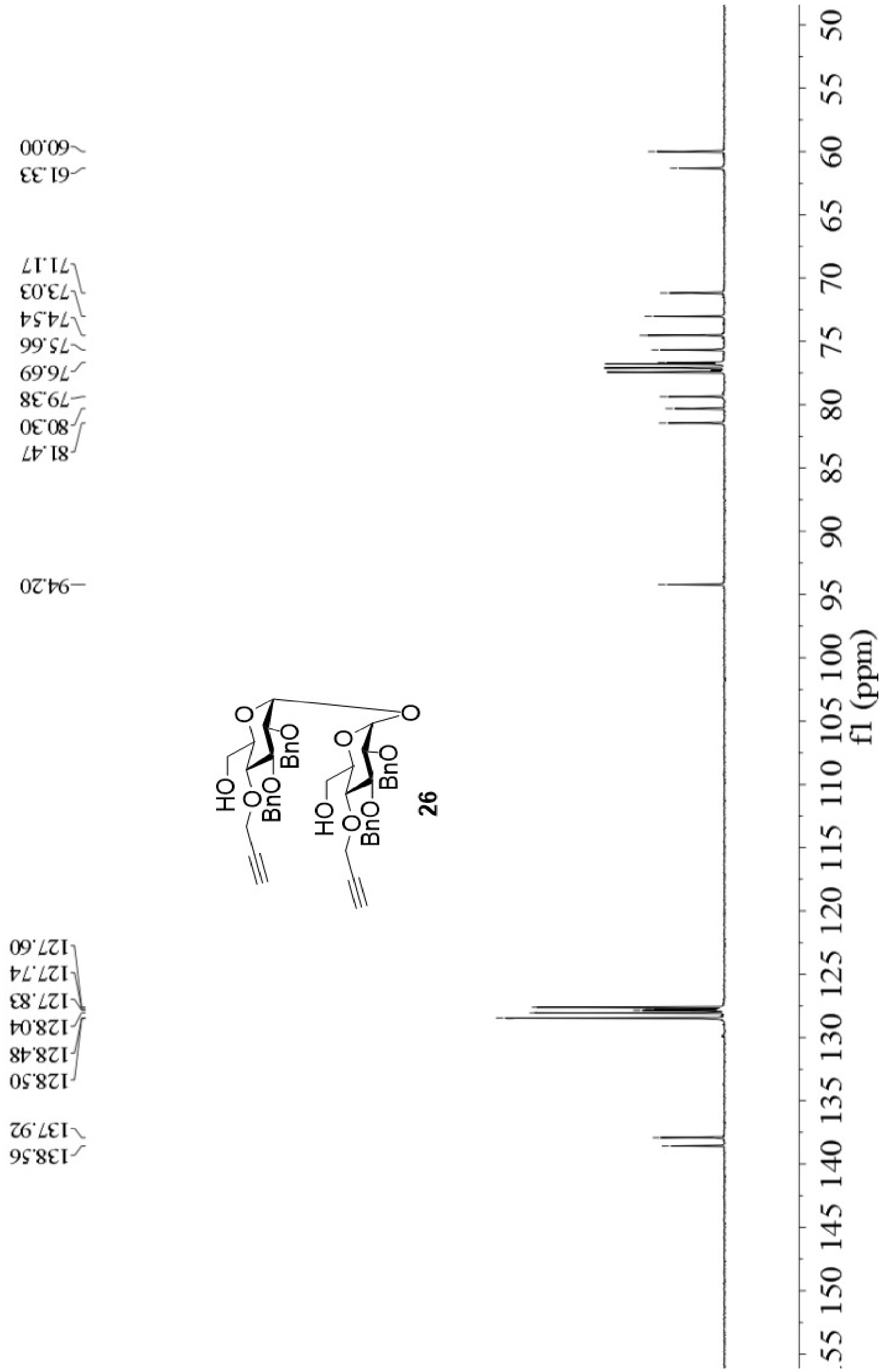

^{13}C NMR Spectrum of compound **26** ($CDCl_3$, 400 MHz)

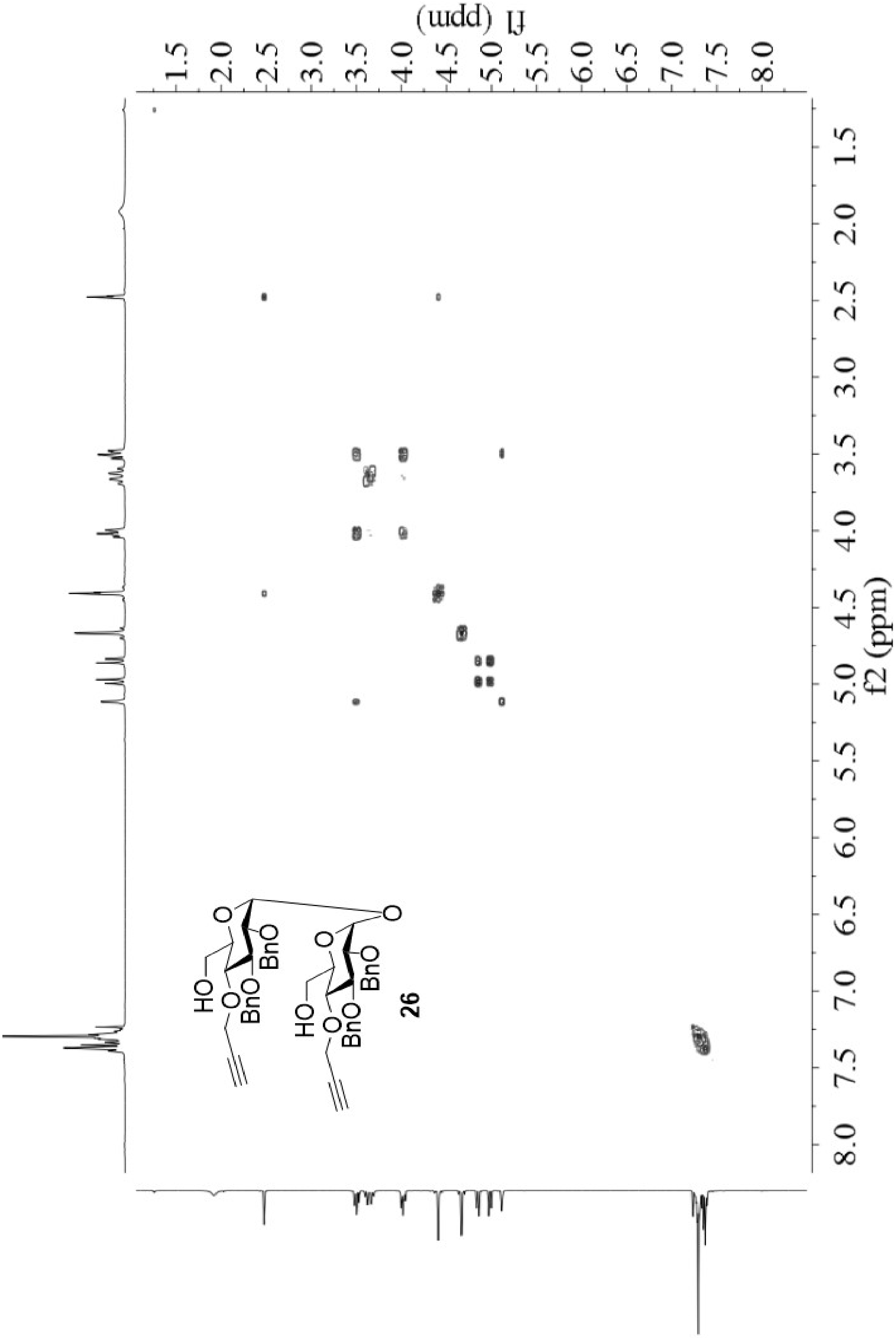

^{1}H-^{1}H COSY NMR Spectrum of compound **26** ($CDCl_3$, 400 MHz)

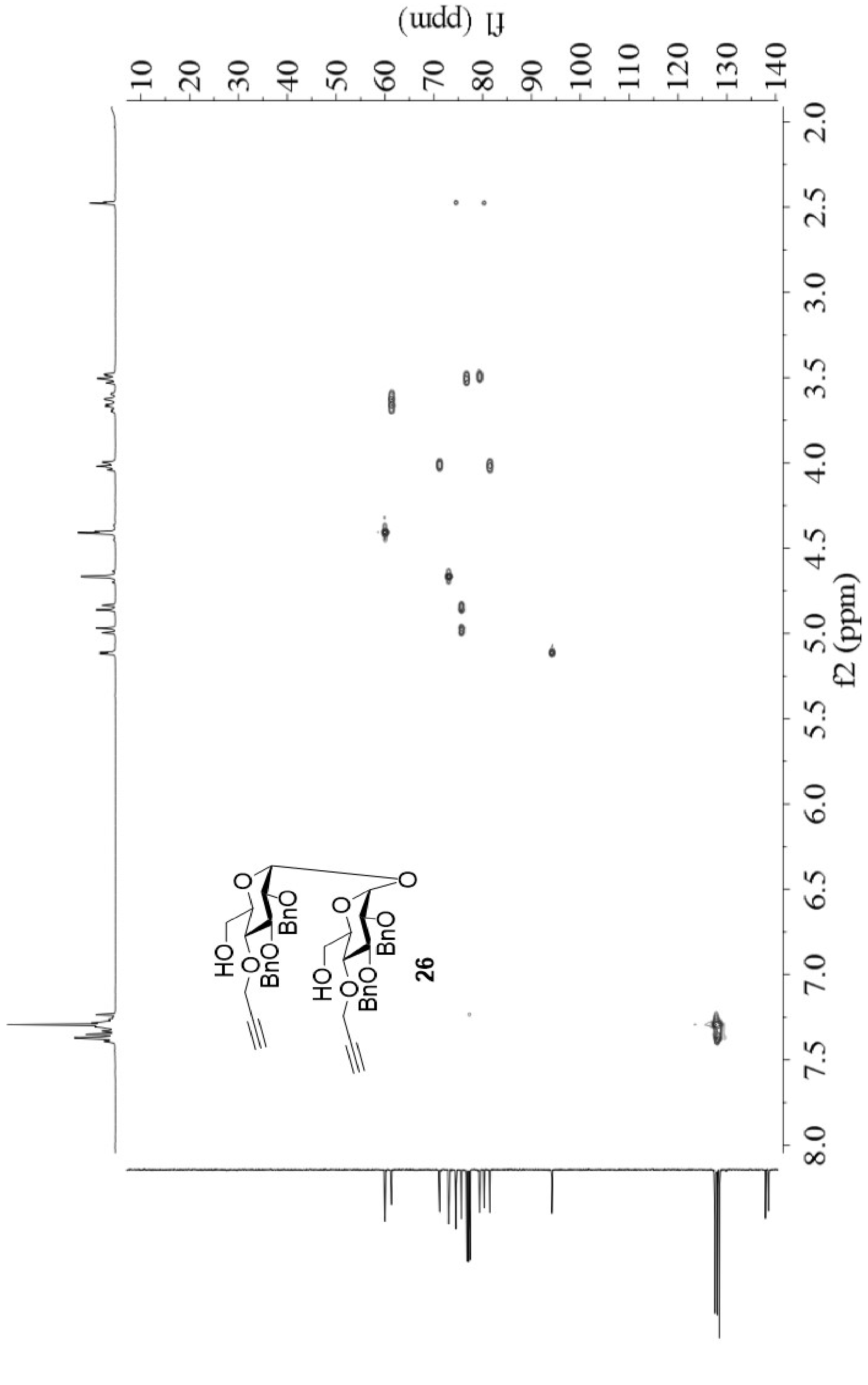

HSQC NMR Spectrum of compound **26** ($CDCl_3$, 400 MHz)

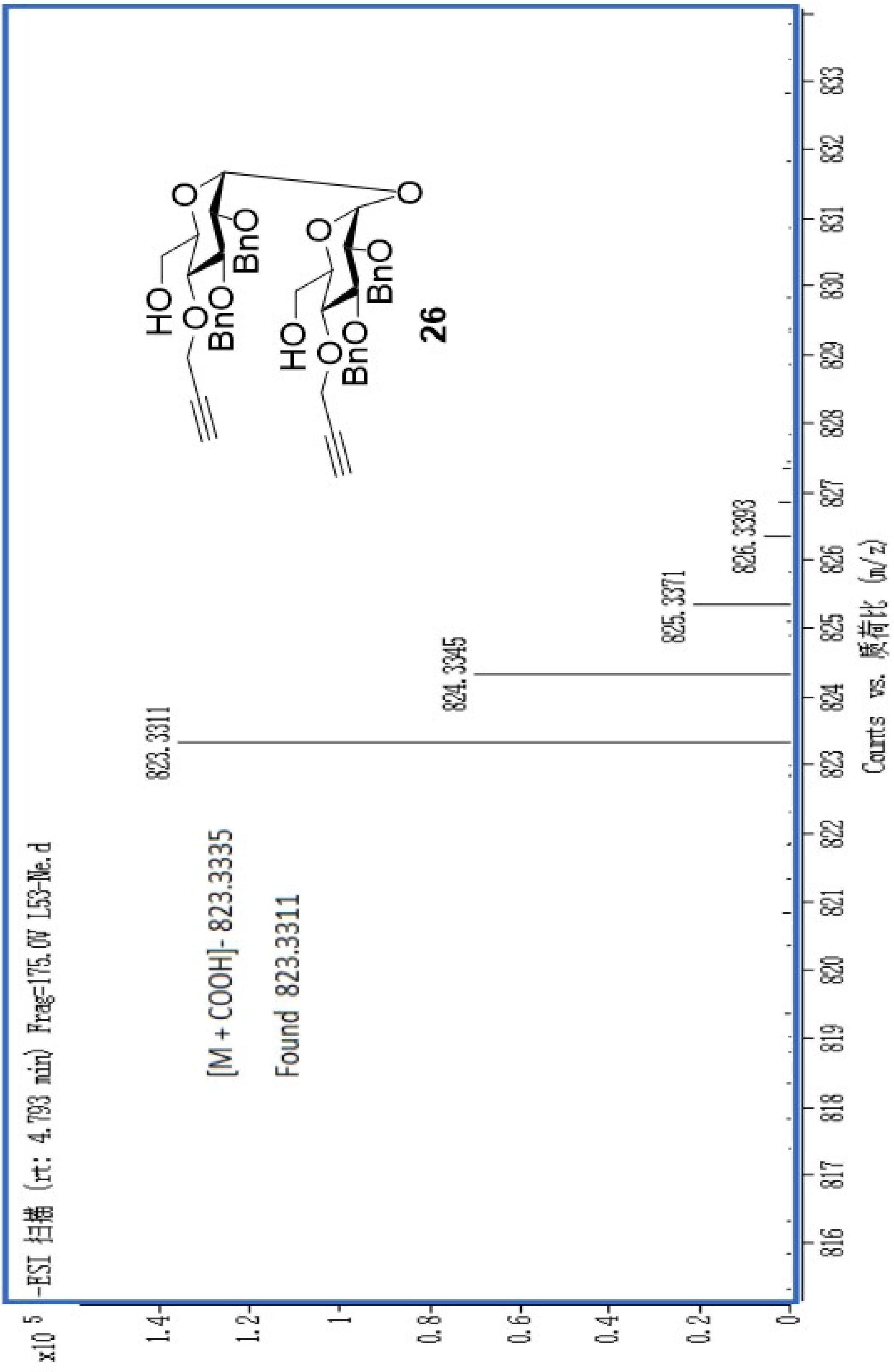

HR-ESI-MS spectrum of conjugate **26**

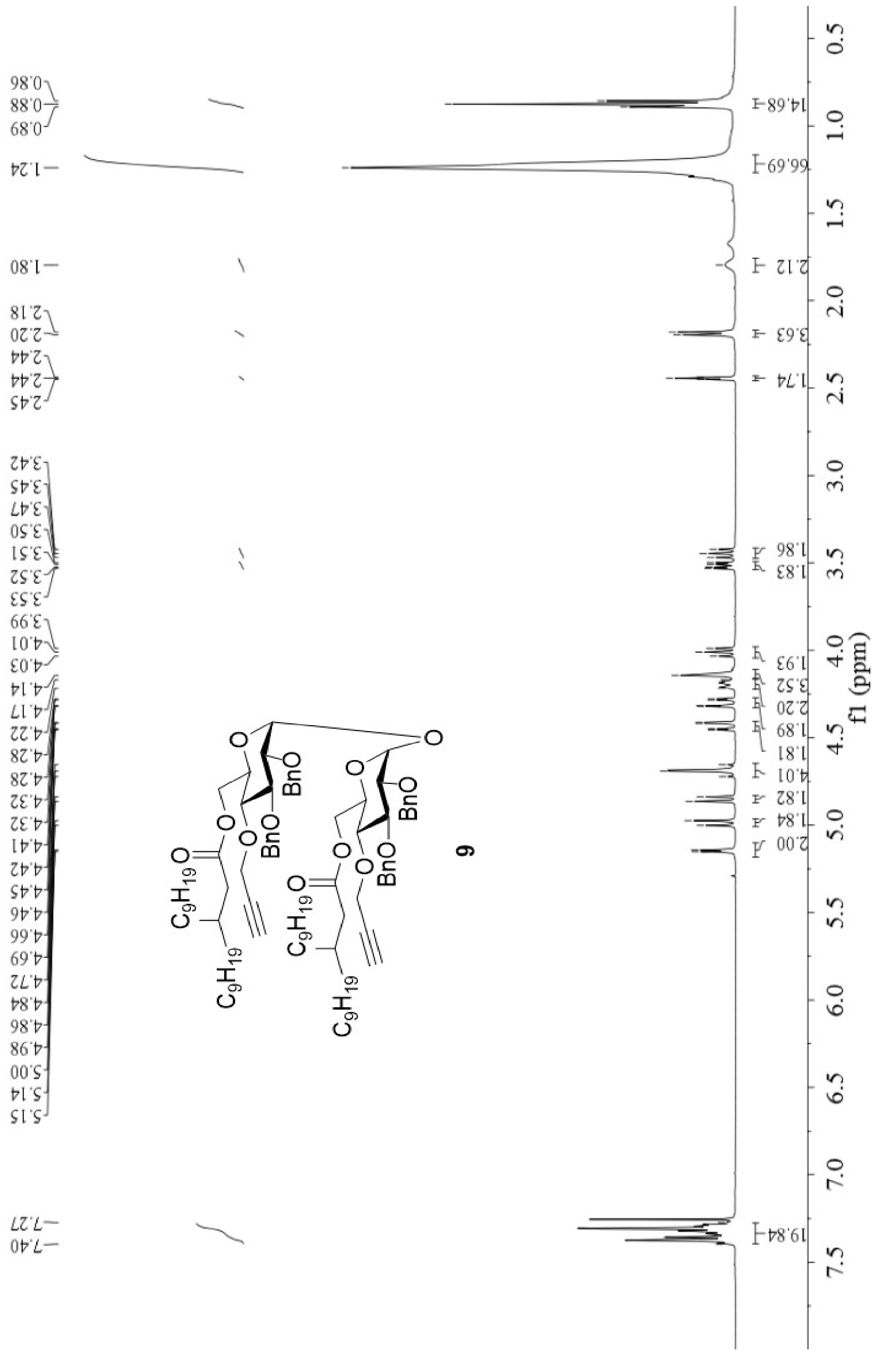

^{1}H NMR Spectrum of compound **9** ($CDCl_3$, 400 MHz)

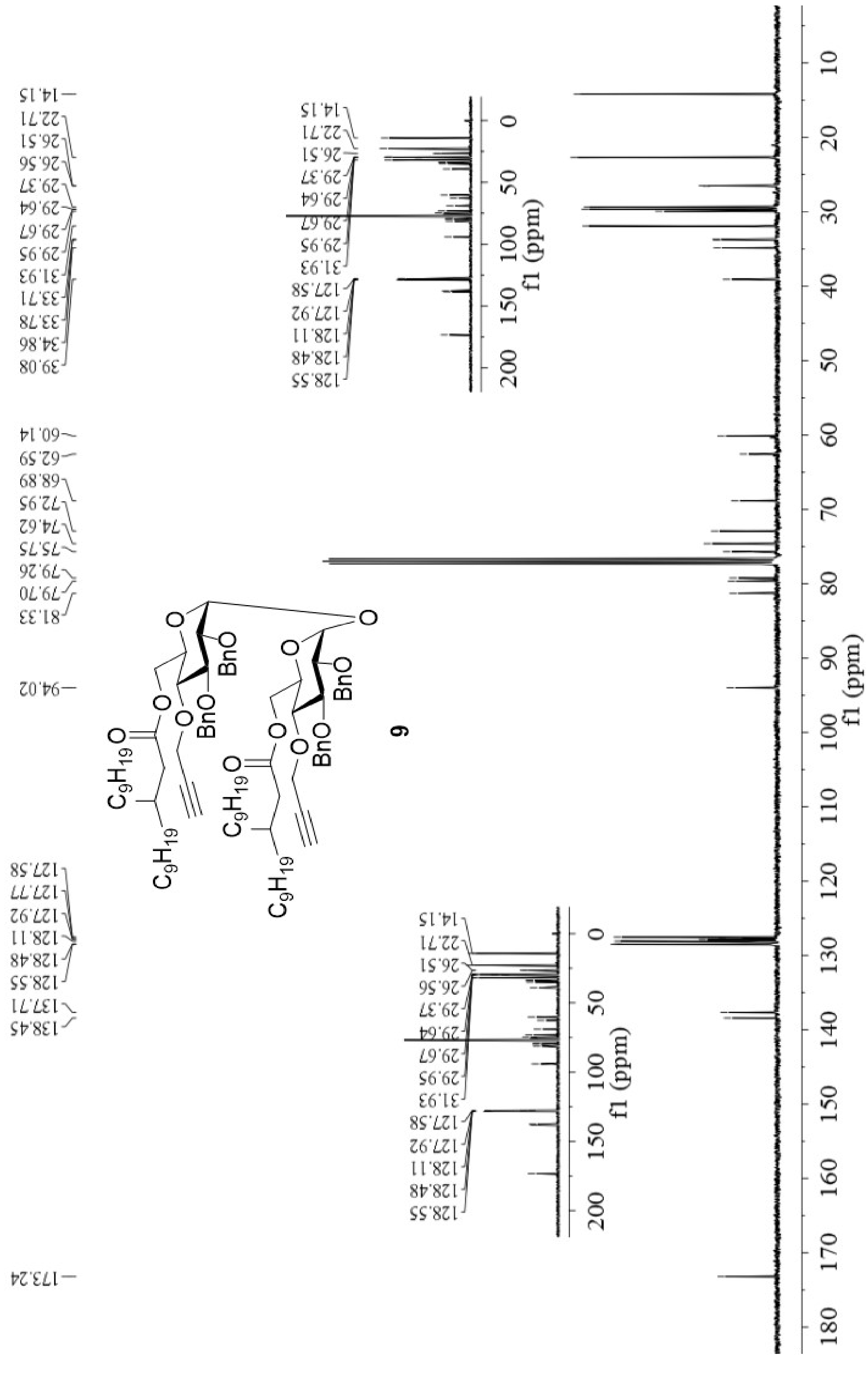

^{13}C NMR Spectrum of compound **9** ($CDCl_3$, 400 MHz)

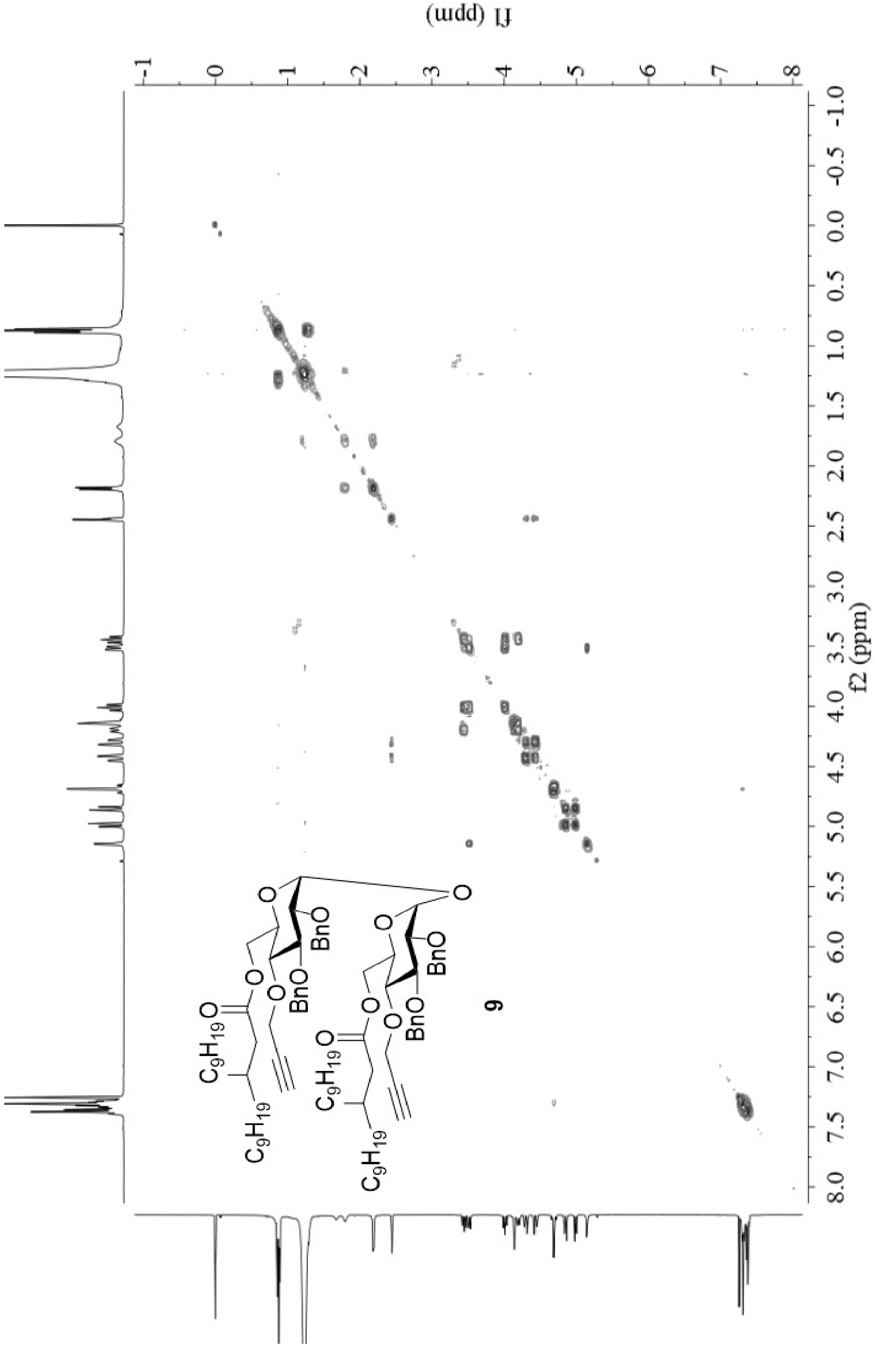

^1H-^1H COSY NMR Spectrum of compound **9** ($CDCl_3$, 400 MHz)

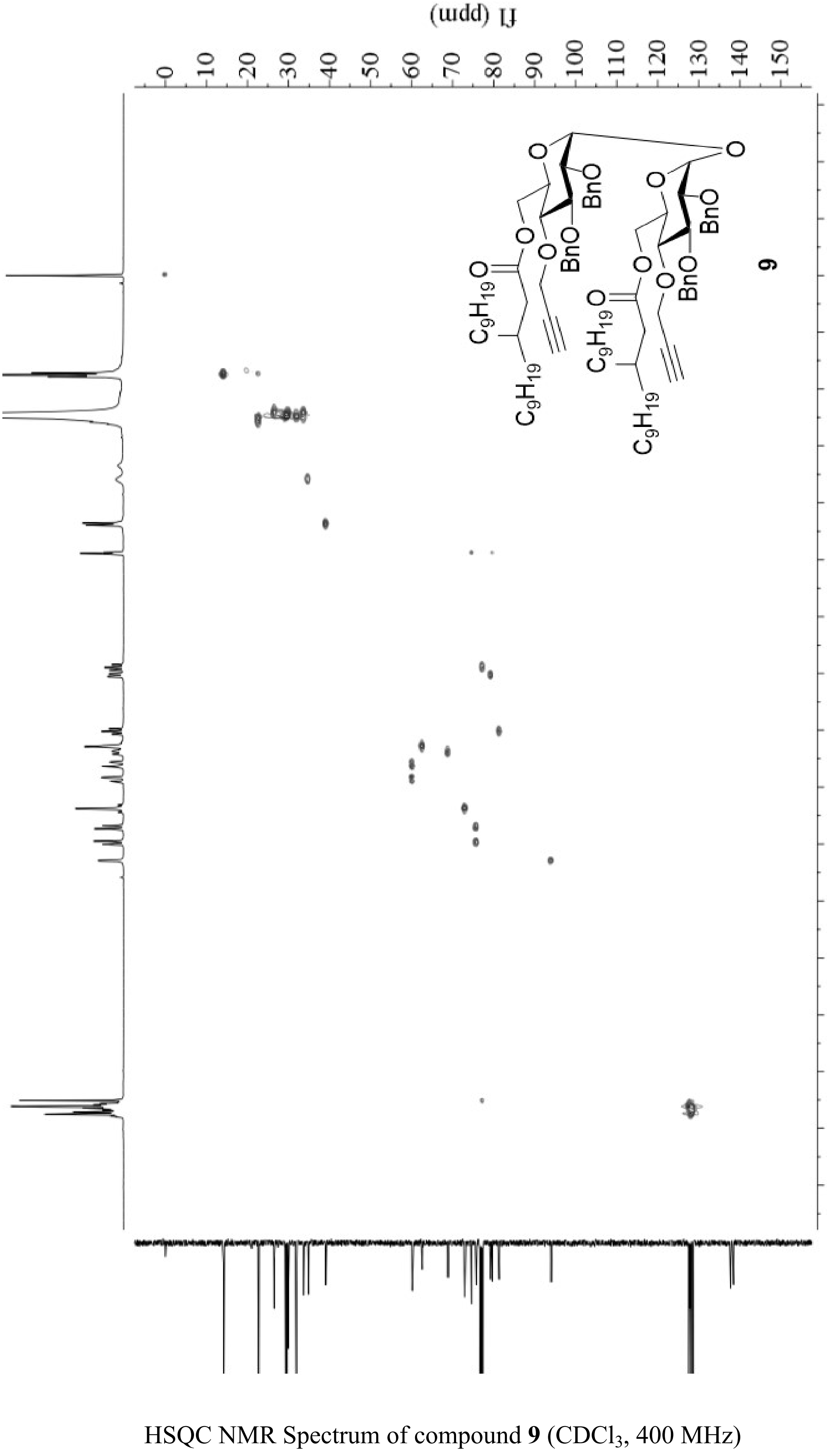

HSQC NMR Spectrum of compound **9** ($CDCl_3$, 400 MHz)

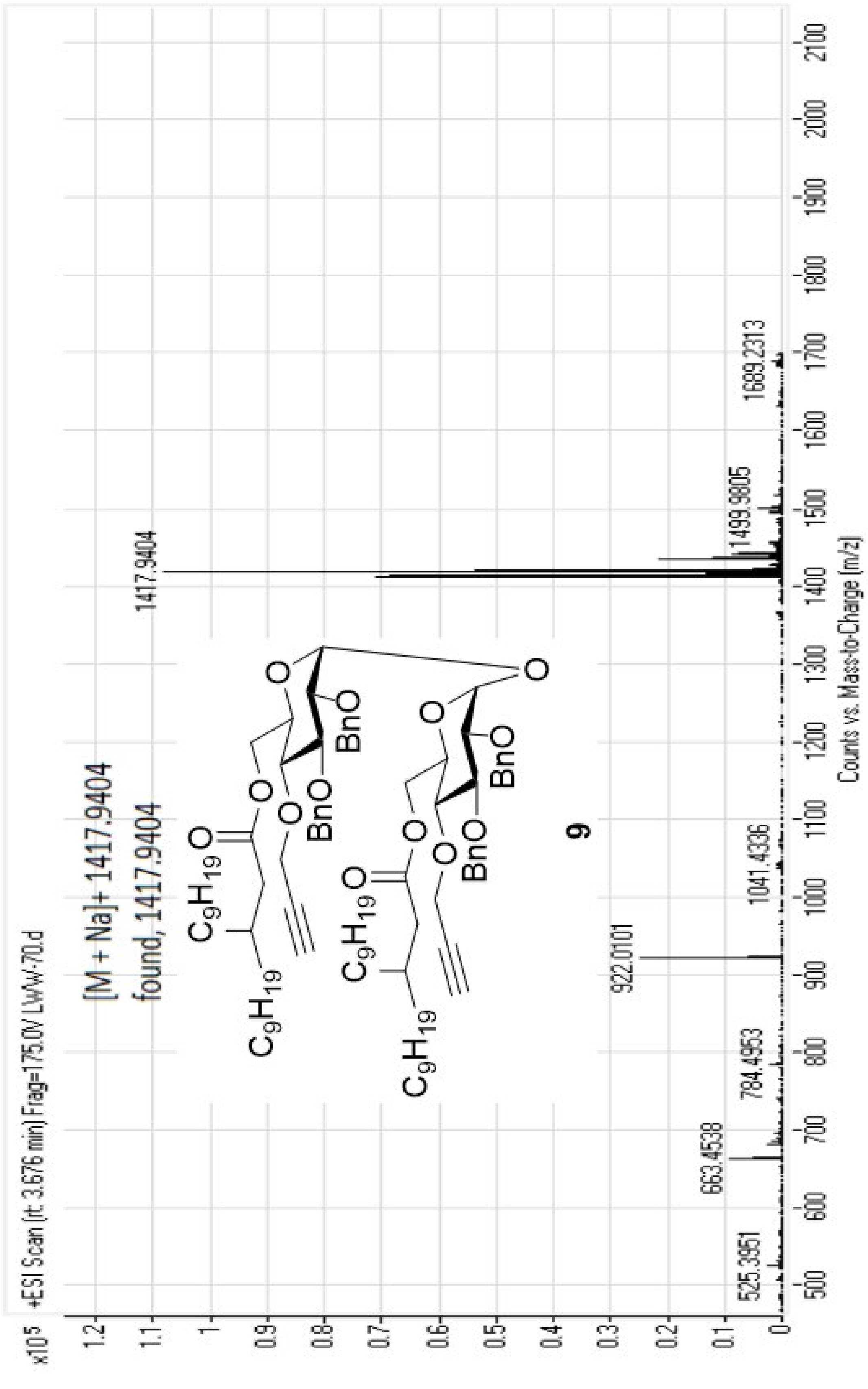

HR-ESI-MS spectrum of conjugate **9**

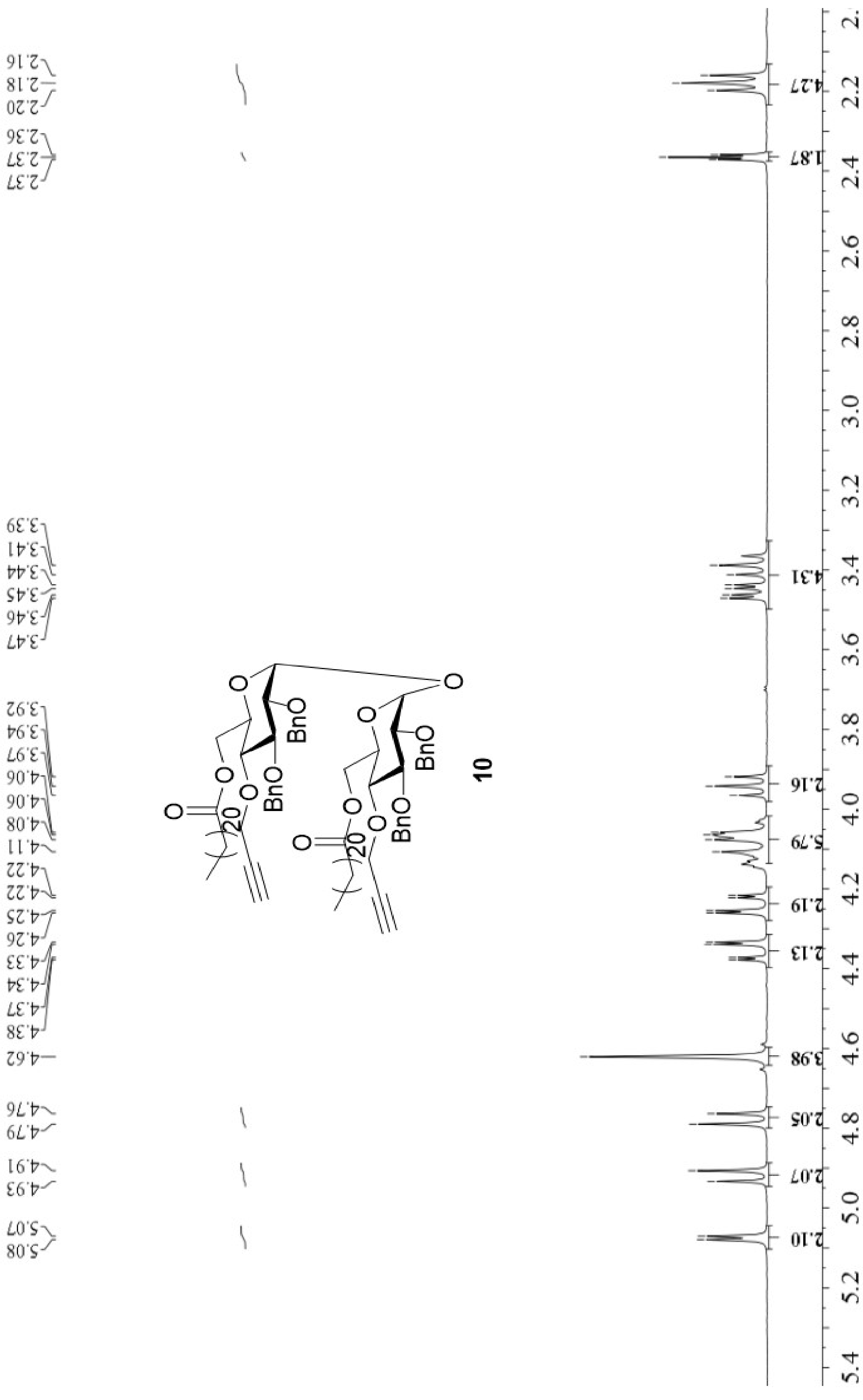

^{1}H NMR Spectrum of compound **10** ($CDCl_3$, 400 MHz)

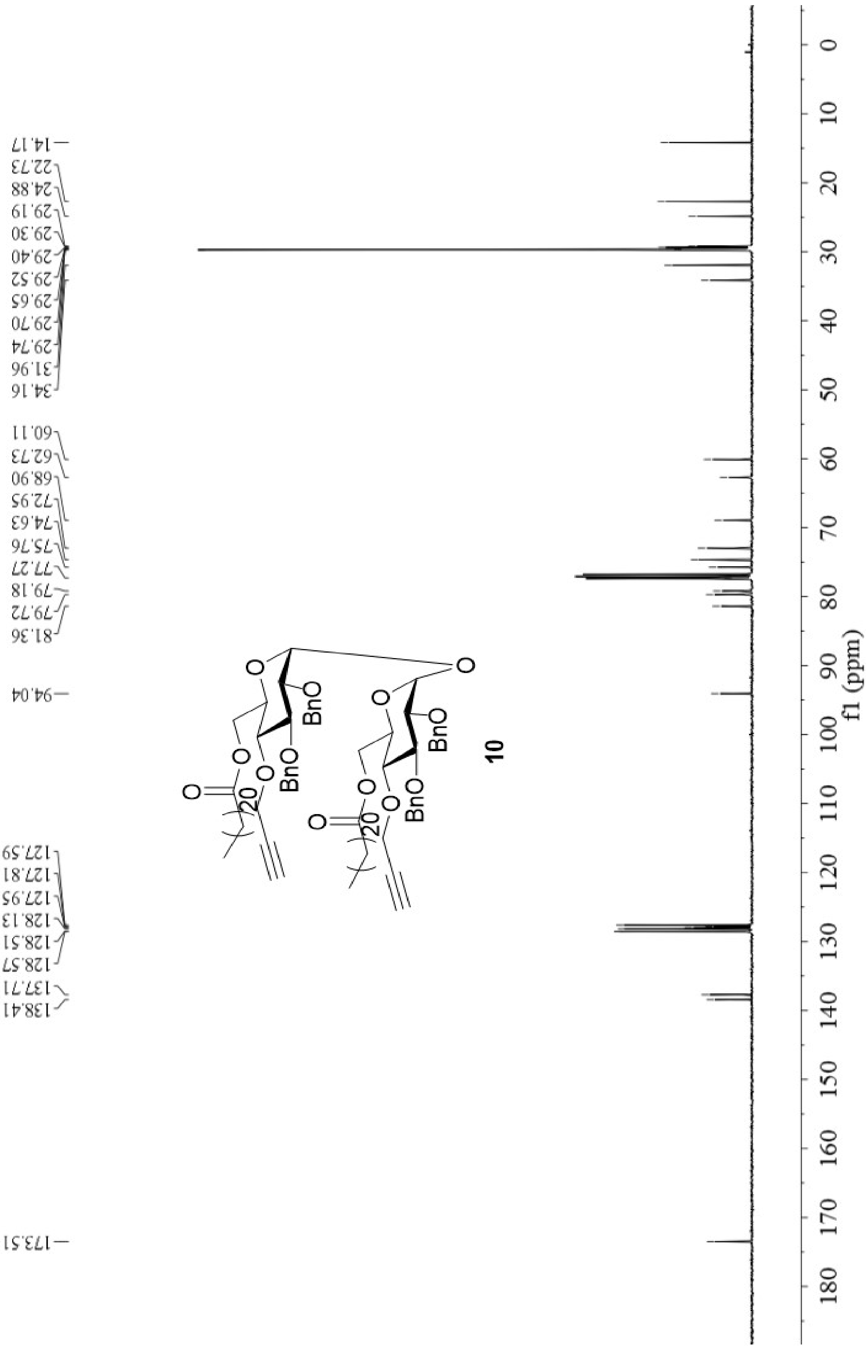

^{13}C NMR Spectrum of compound **10** ($CDCl_3$, 400 MHz)

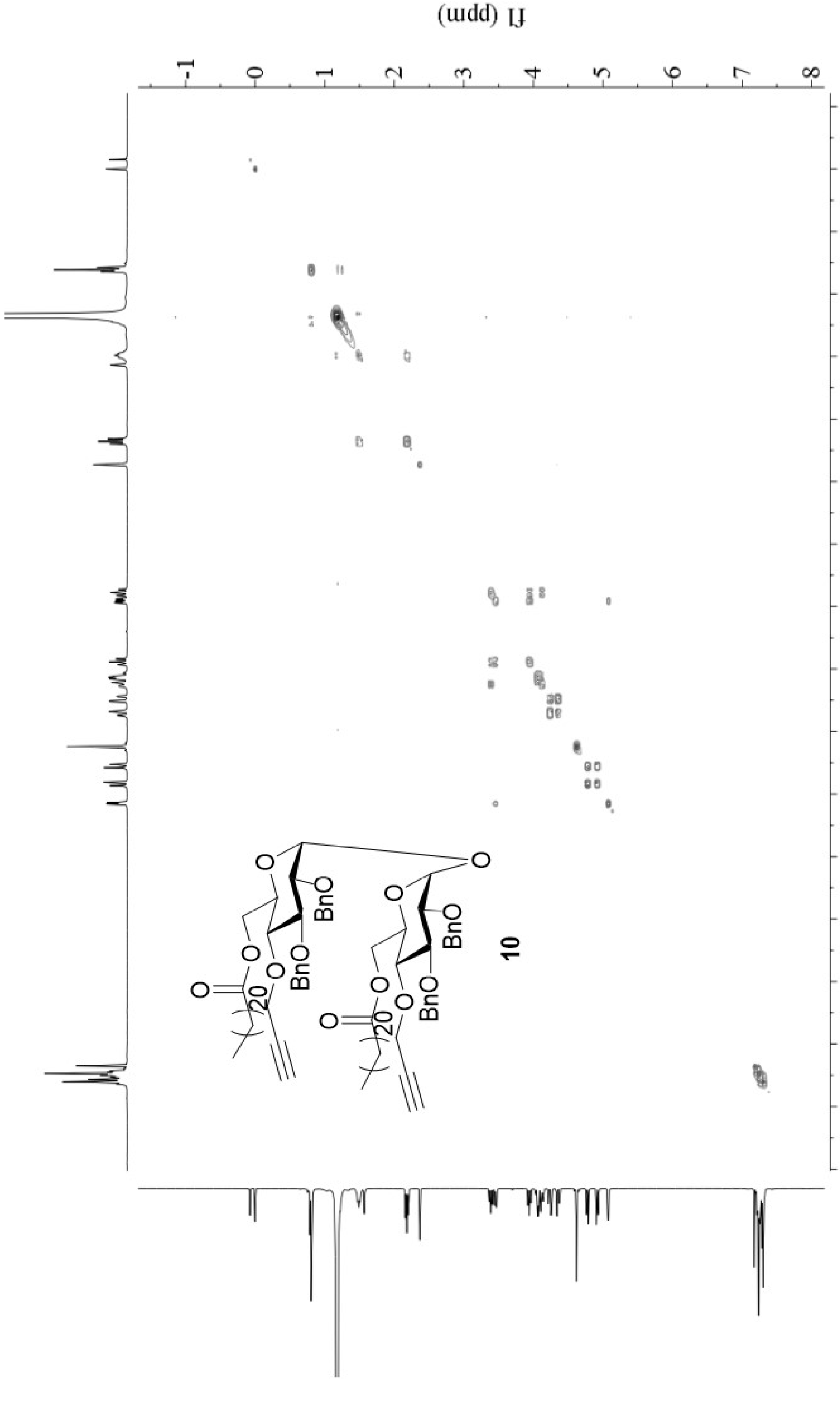

^{1}H-^{1}H COSY NMR Spectrum of compound **10** ($CDCl_3$, 400 MHz)

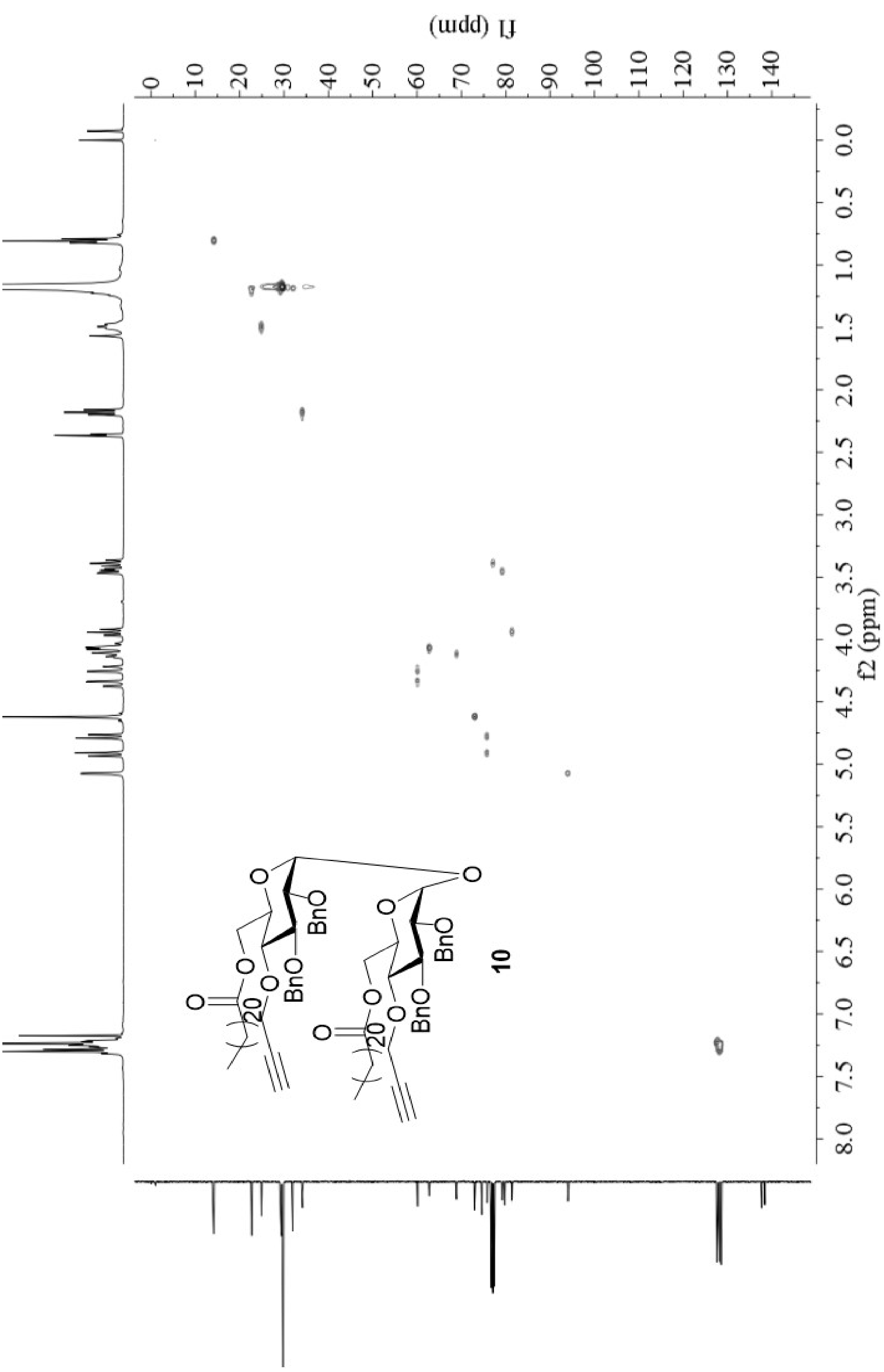

HSQC NMR Spectrum of compound **10** ($CDCl_3$, 400 MHz)

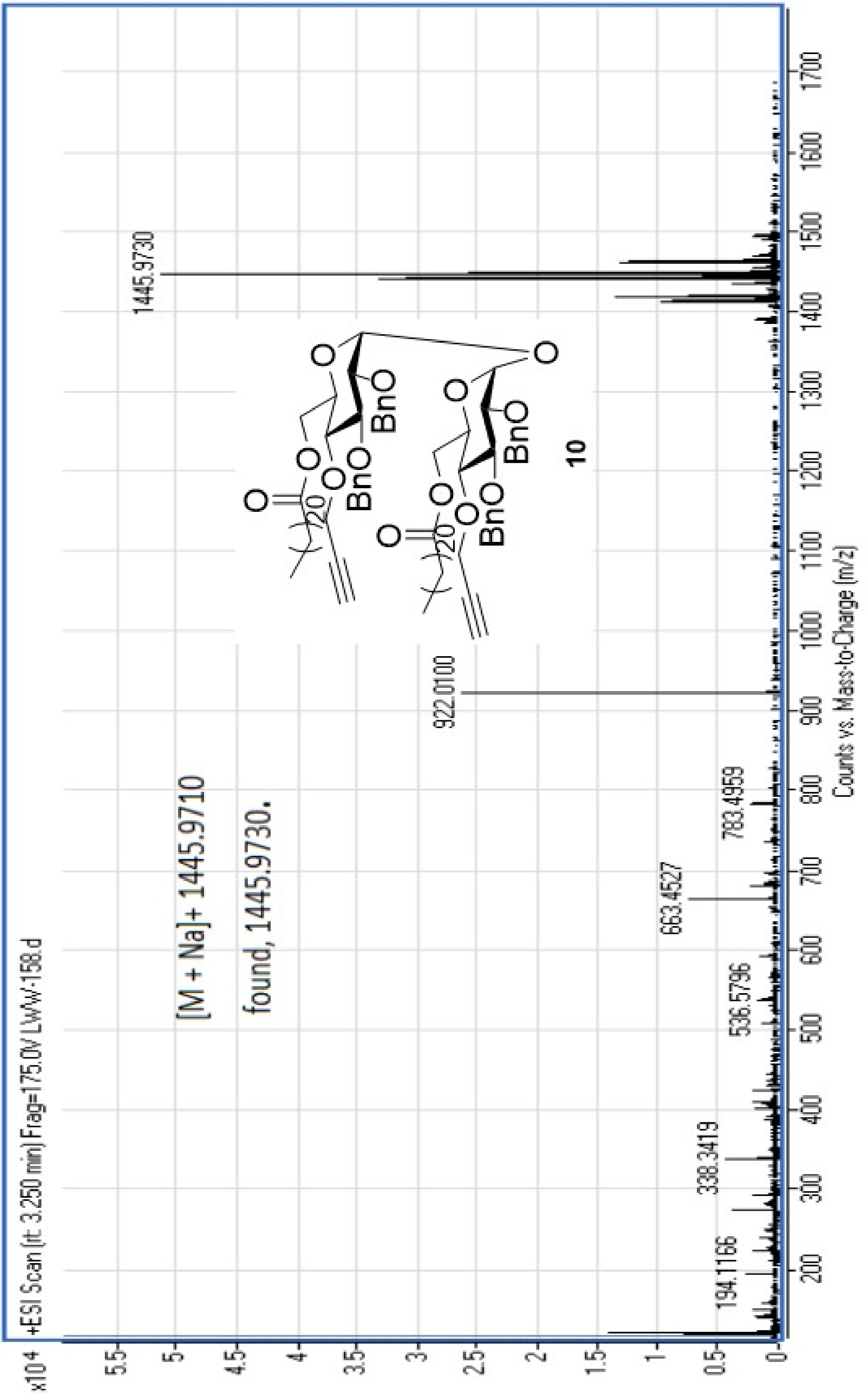

HR-ESI-MS spectrum of conjugate **10**

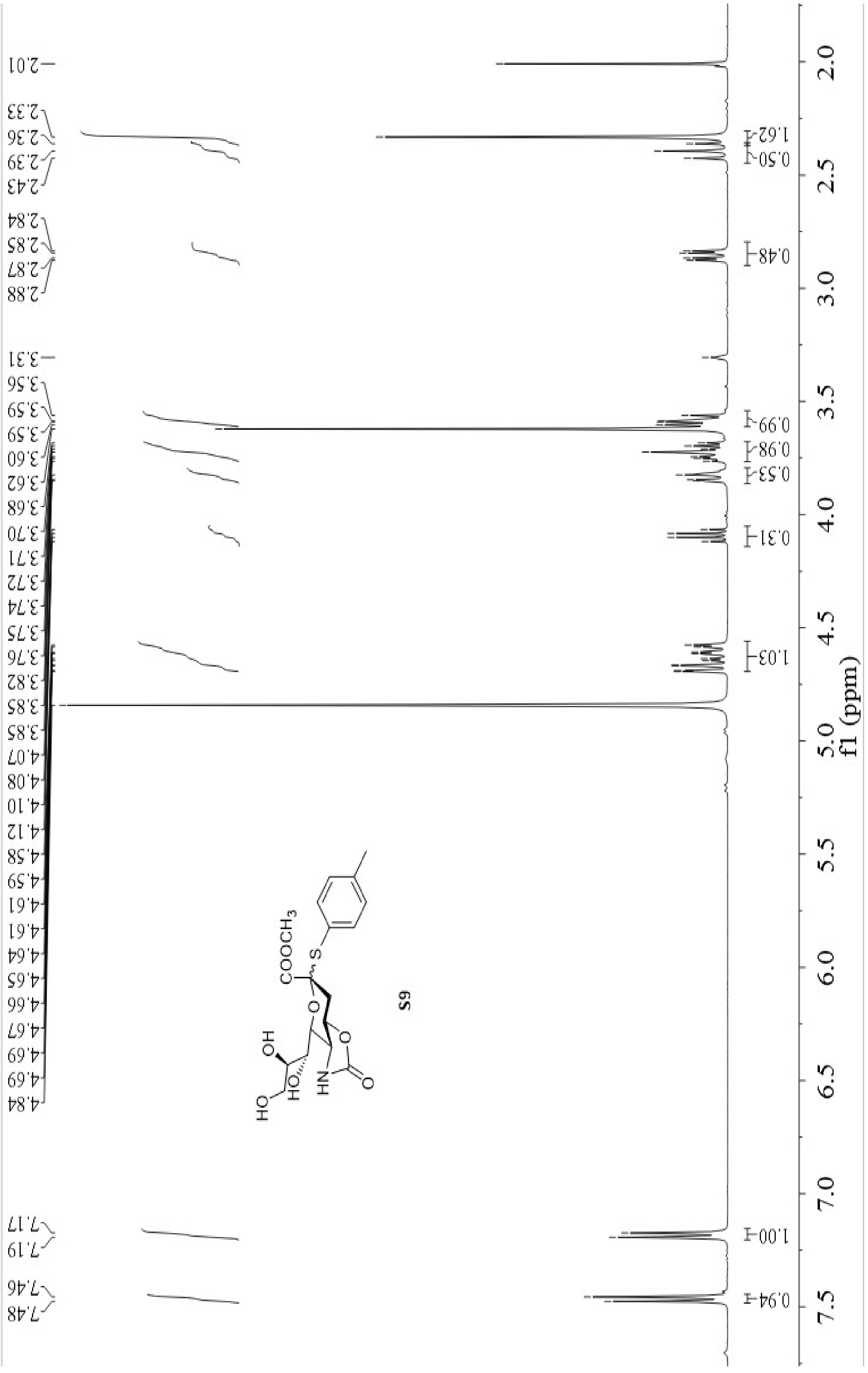

^{1}H NMR Spectrum of compound **S9** (MeOD, 400 MHz)

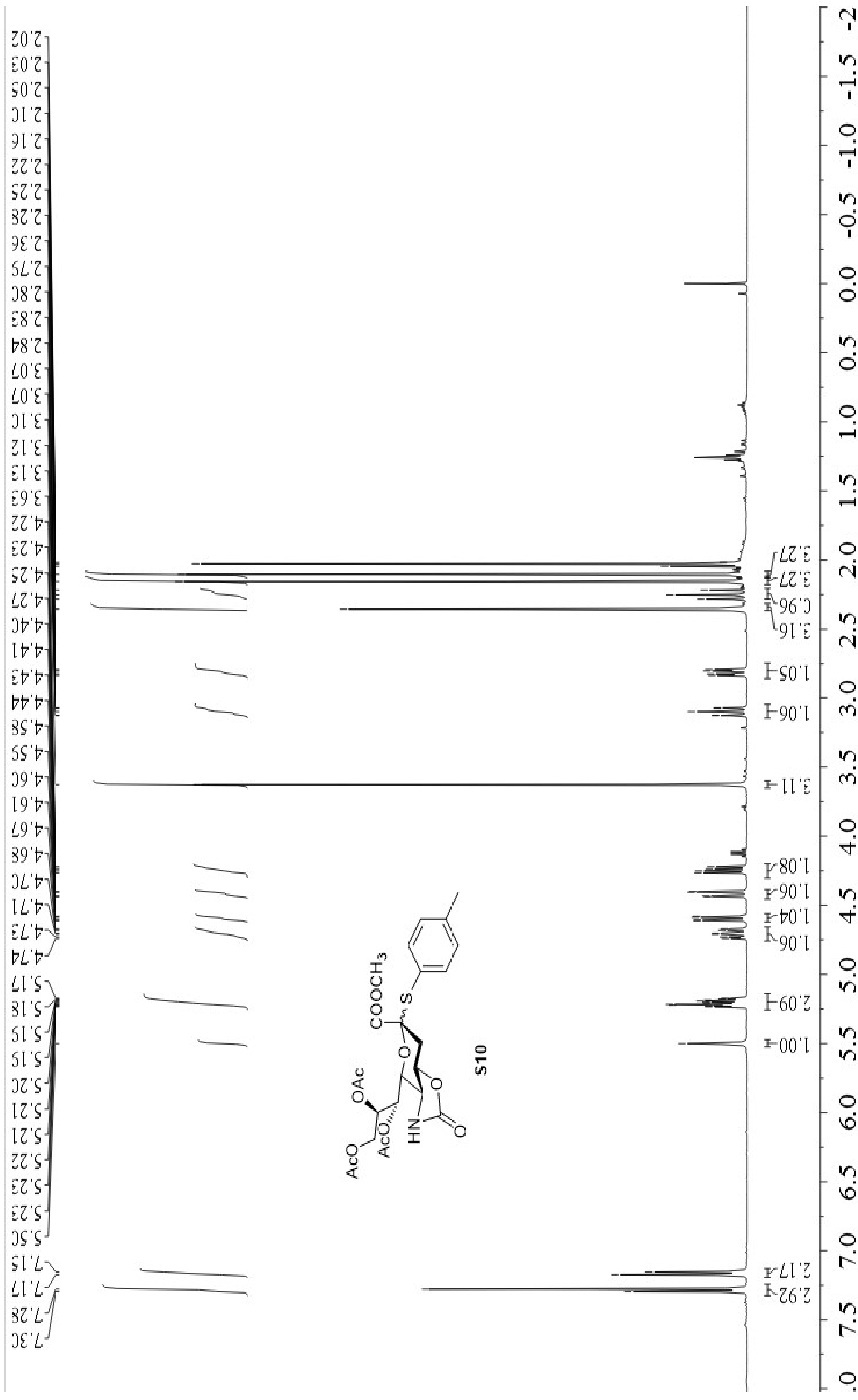

^{1}H NMR Spectrum of compound **S10** ($CDCl_3$, 400 MHz)

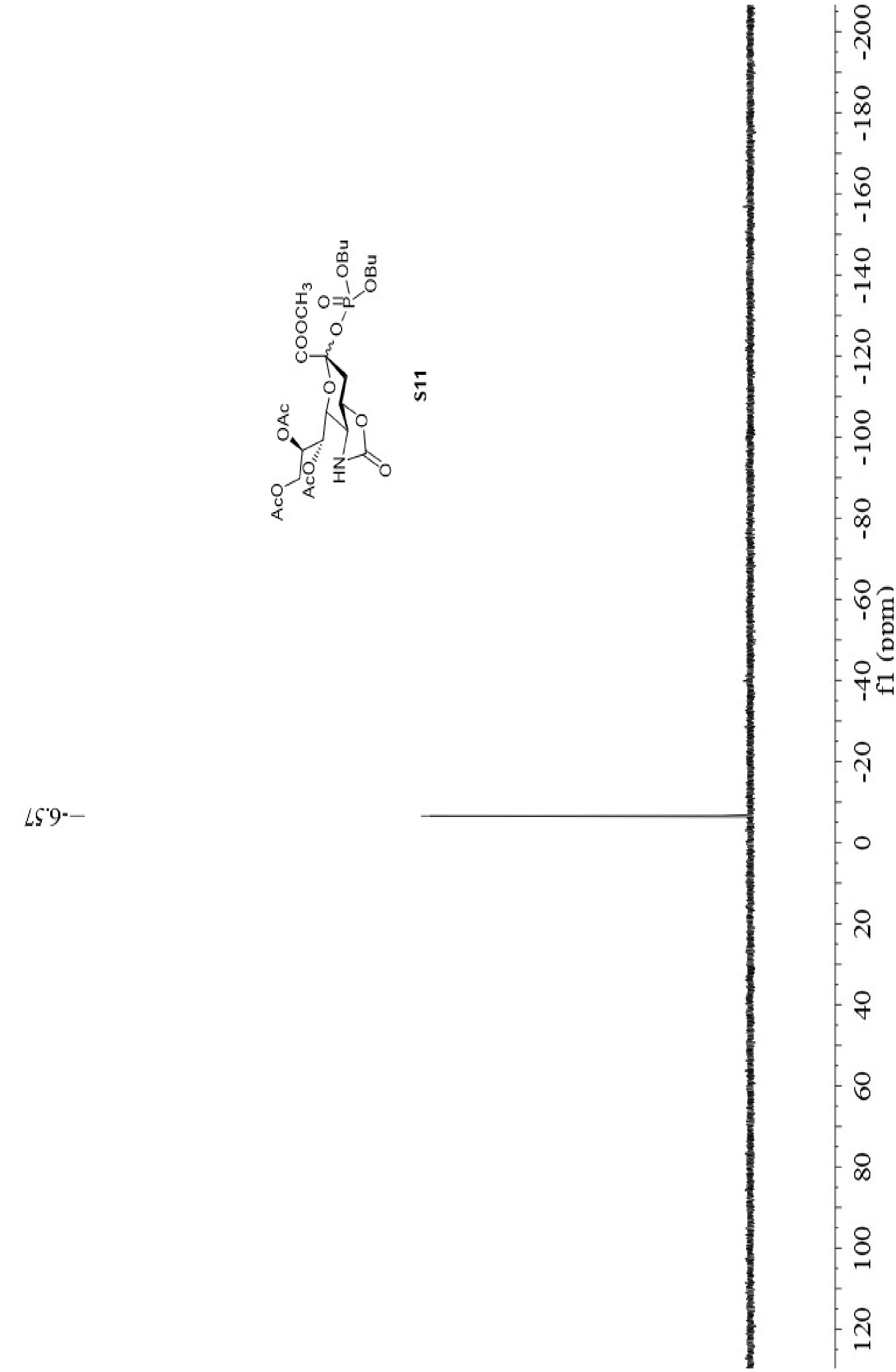

^{31}P NMR Spectrum of compound **S11** ($CDCl_3$, 400 MHz)

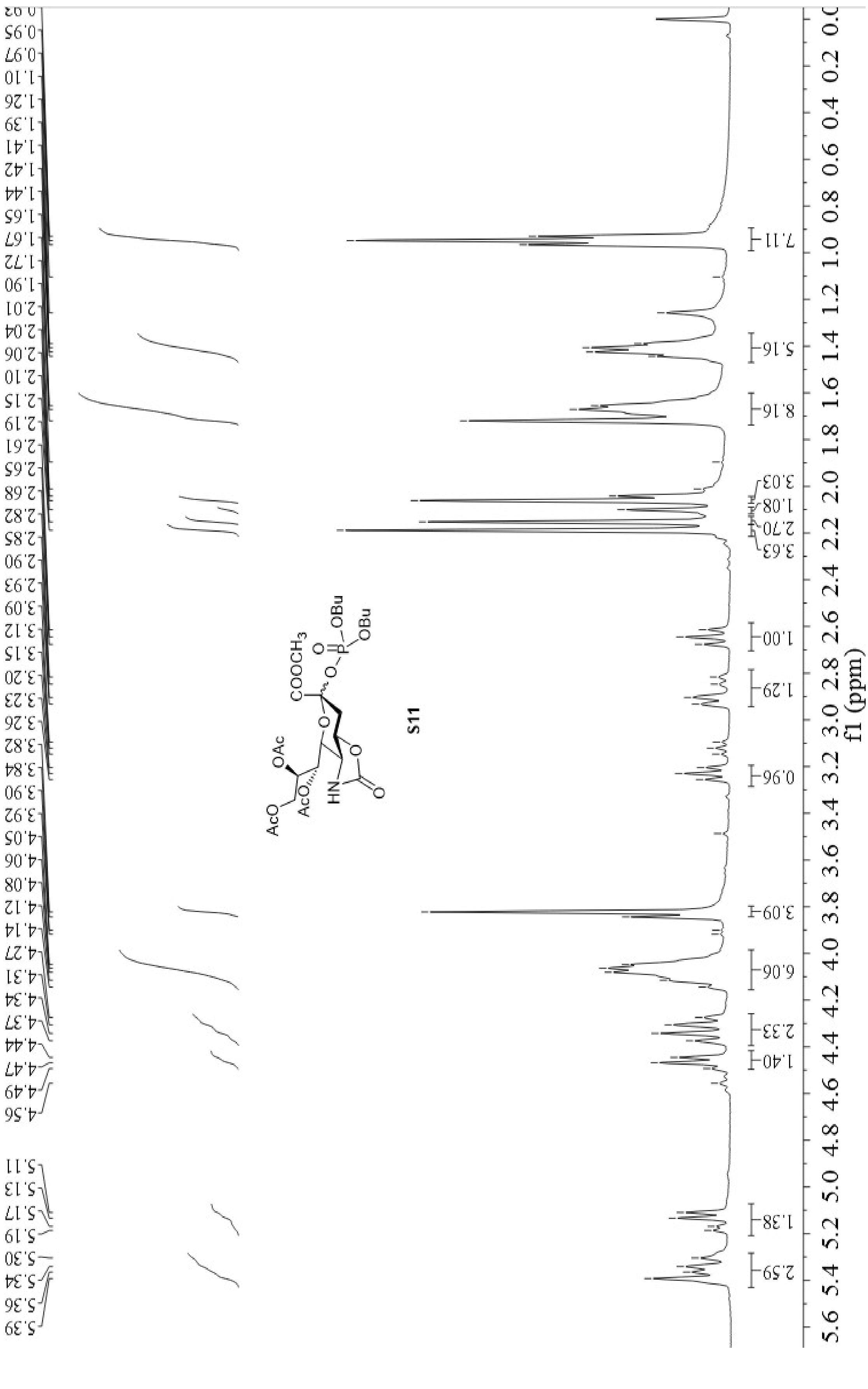

^{1}H NMR Spectrum of compound **S11** ($CDCl_3$, 400 MHz)

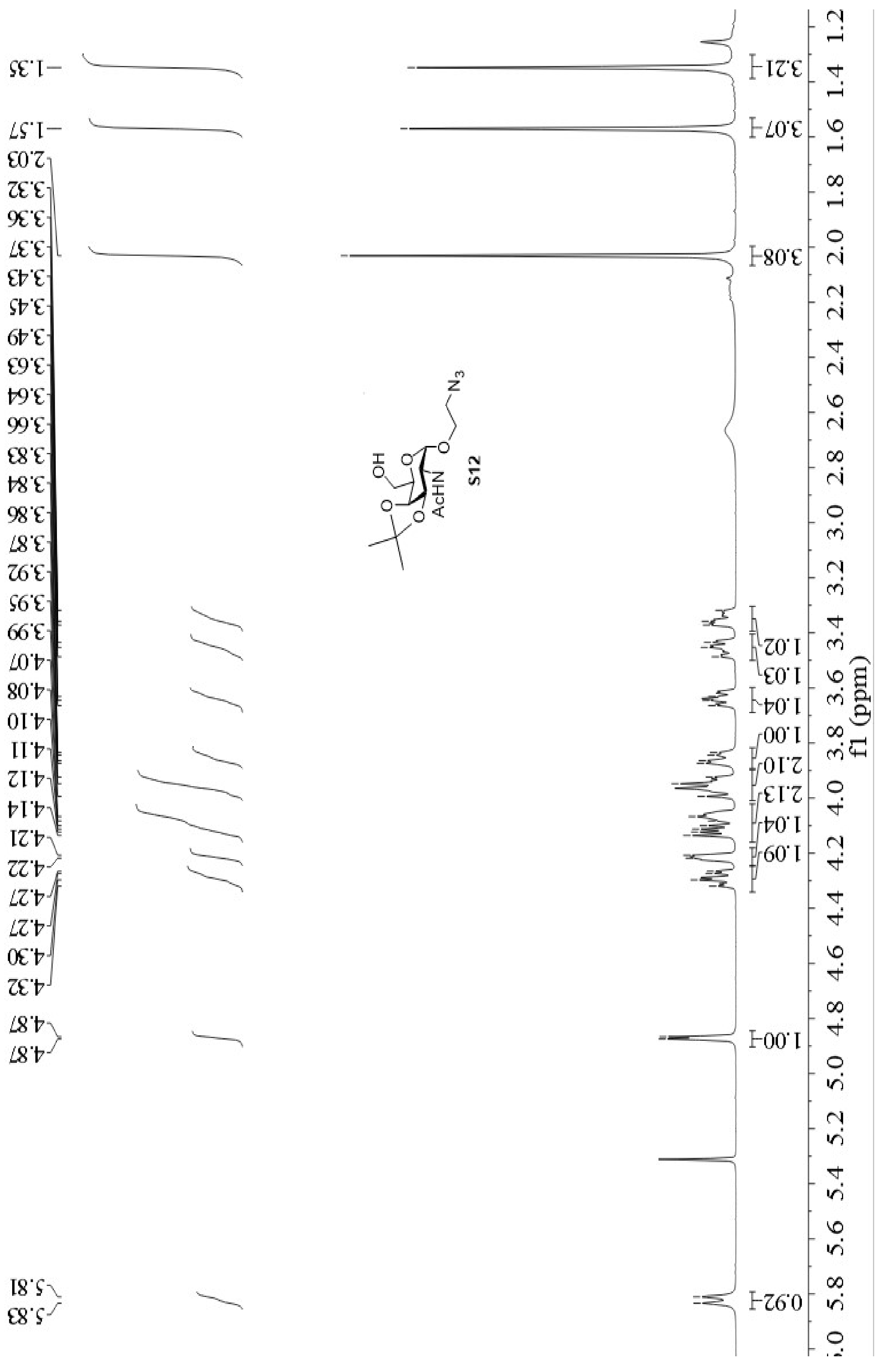

^{1}H NMR Spectrum of compound **S12** ($CDCl_3$, 400 MHz)

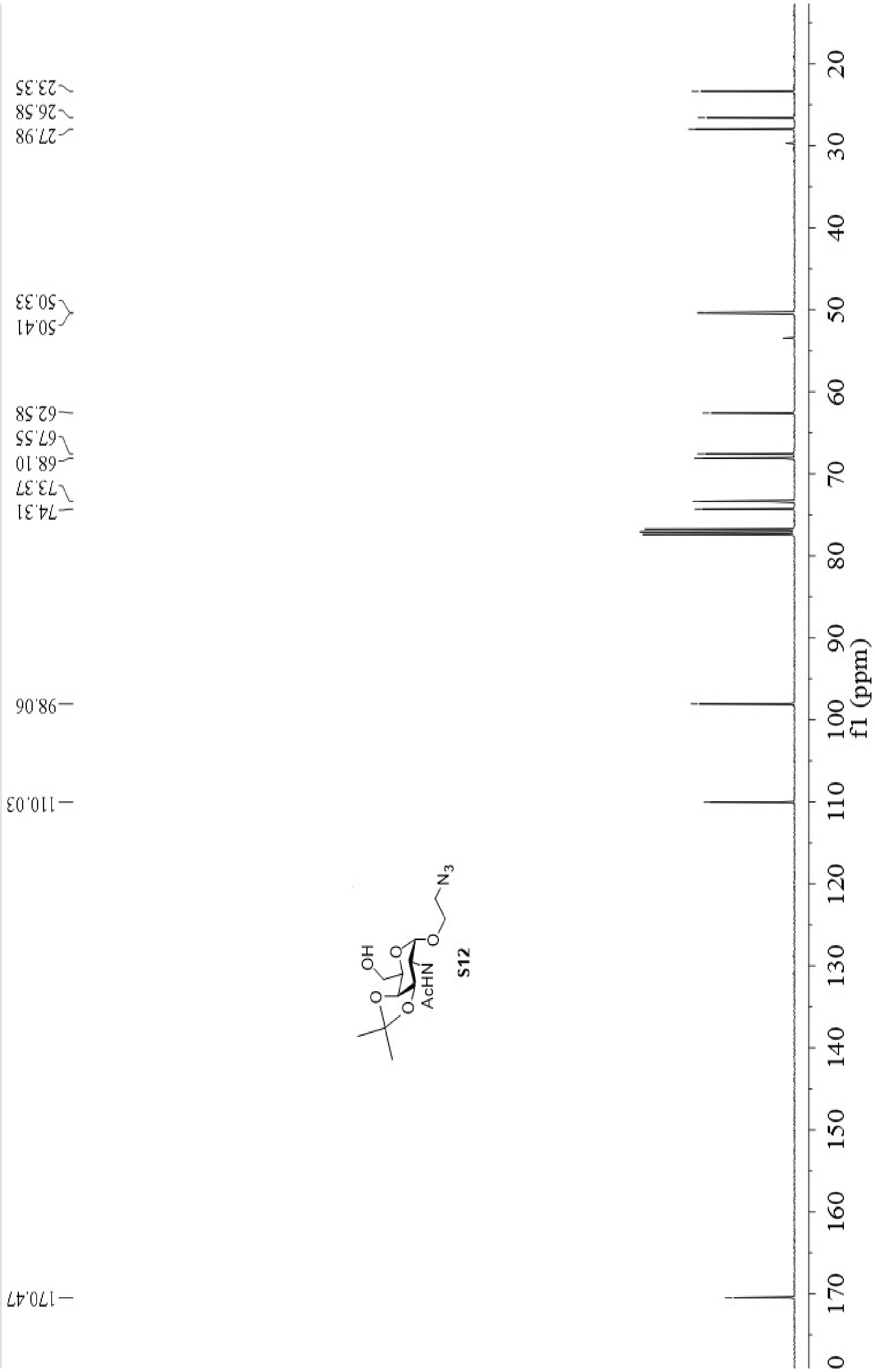

^{13}C NMR Spectrum of compound **S12** ($CDCl_3$, 400 MHz)

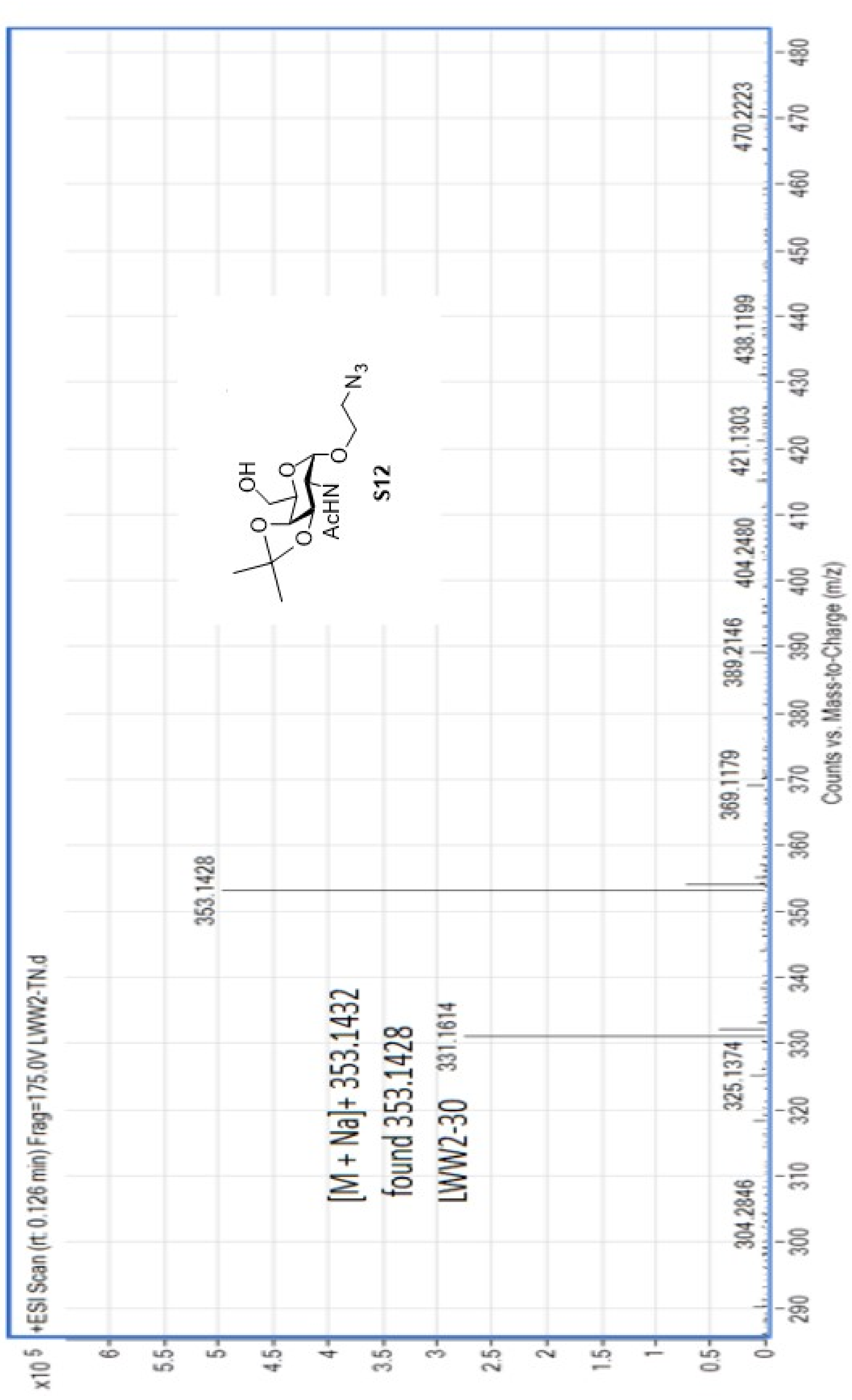

HR-ESI-MS spectrum of conjugate **S12**

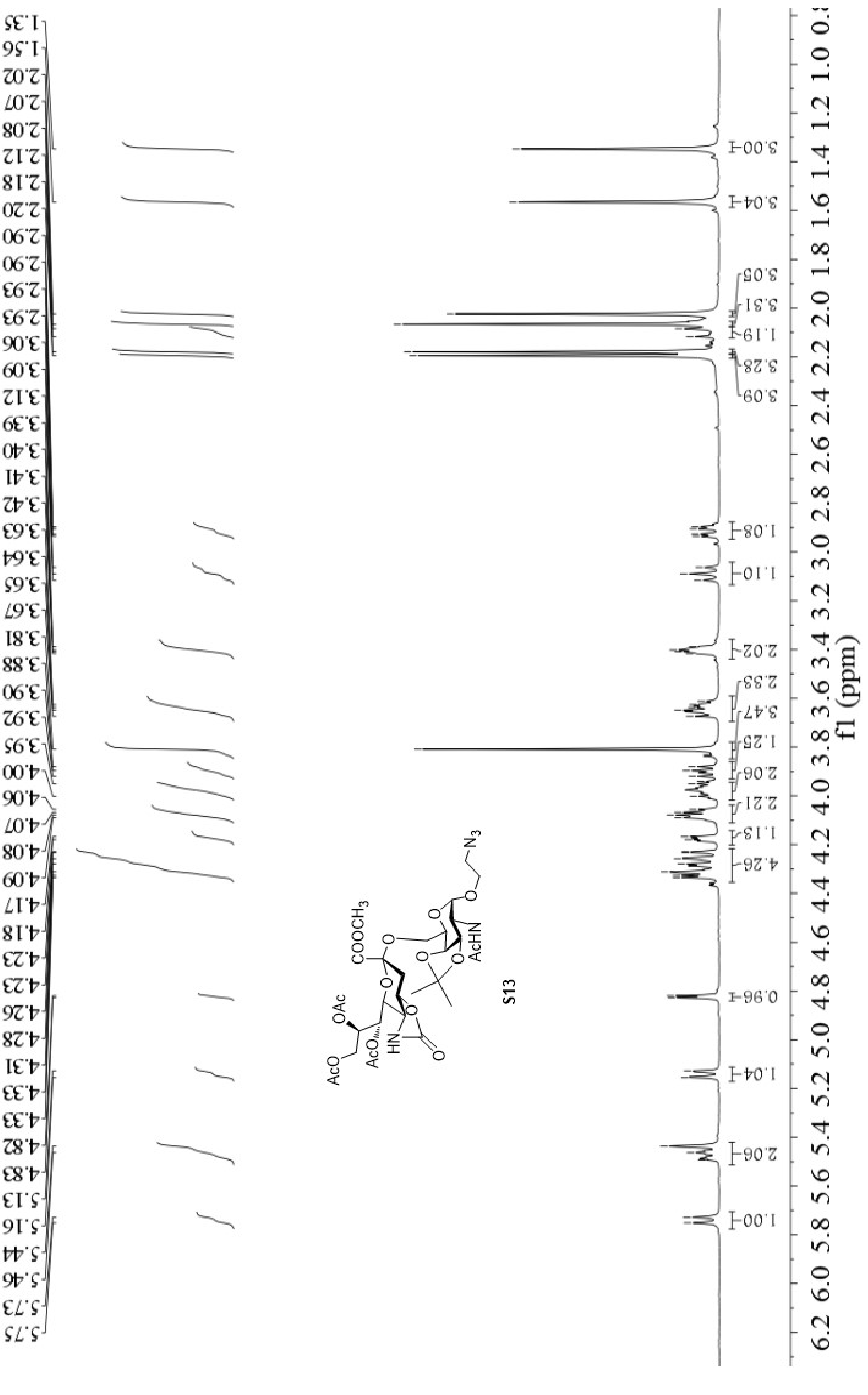

^{1}H NMR Spectrum of compound **S13** ($CDCl_3$, 400 MHz)

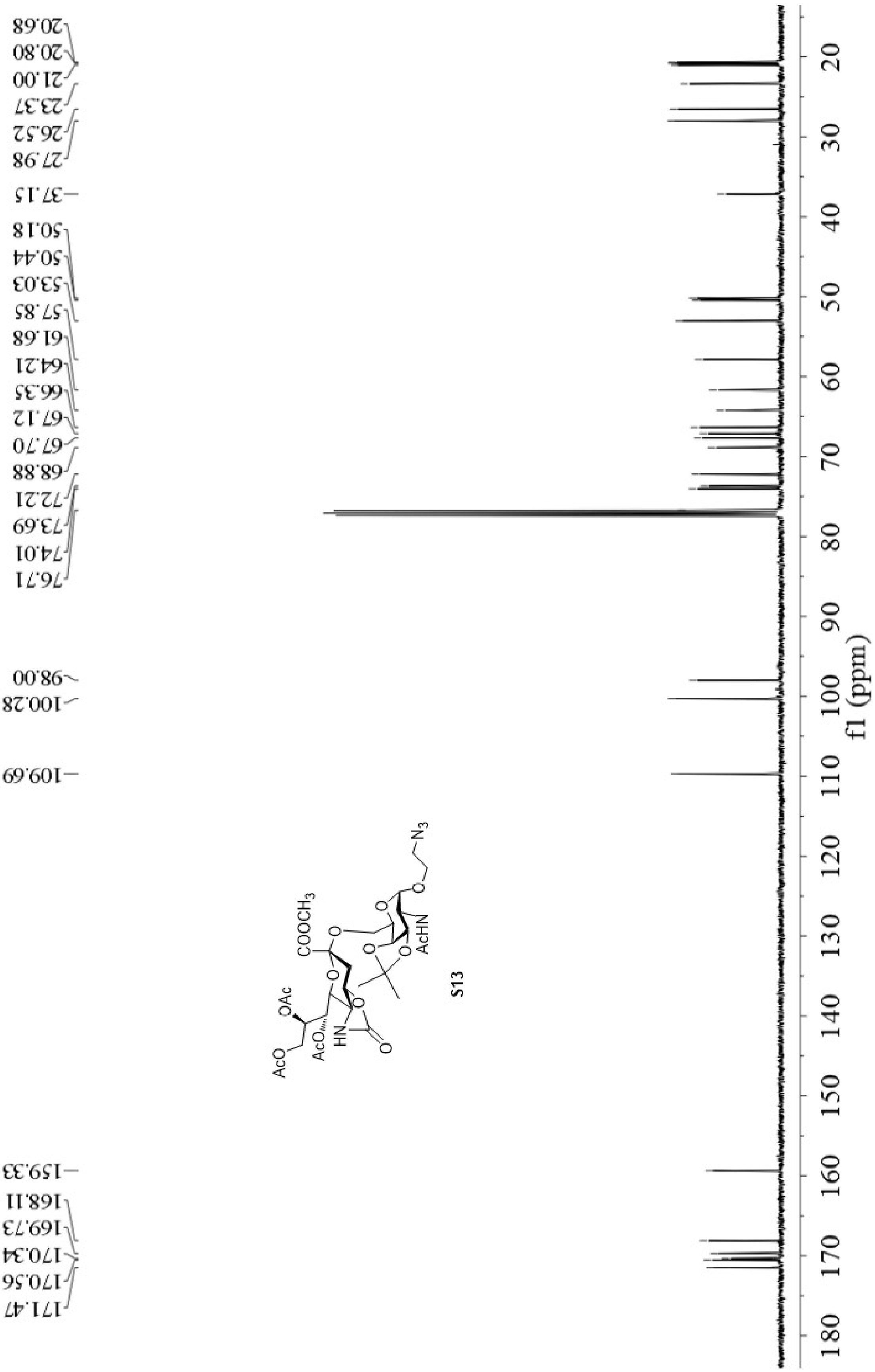

^{13}C NMR Spectrum of compound **S13** ($CDCl_3$, 400 MHz)

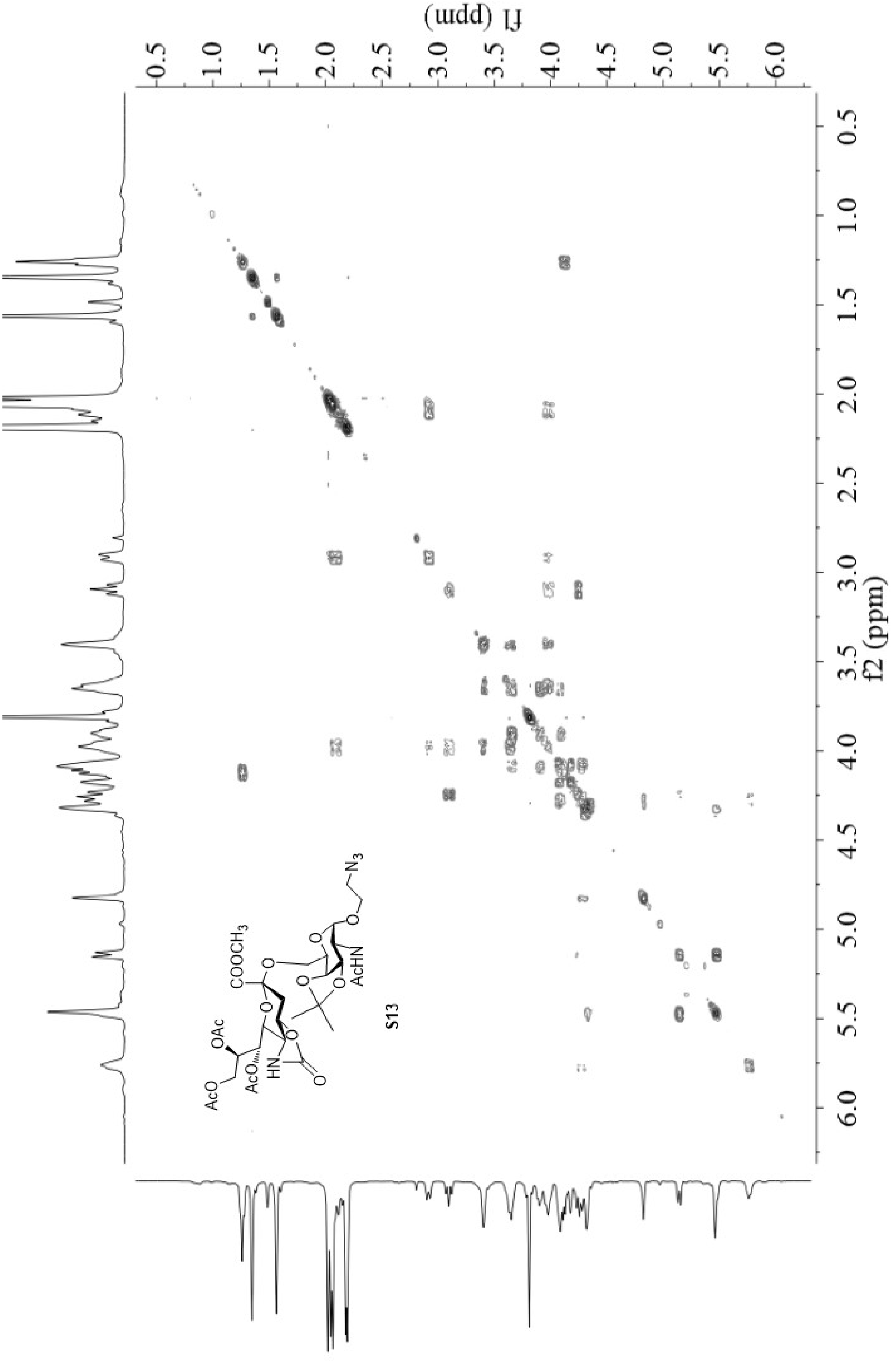

^1H-^1H COSY NMR Spectrum of compound **S13** ($CDCl_3$, 400 MHz)

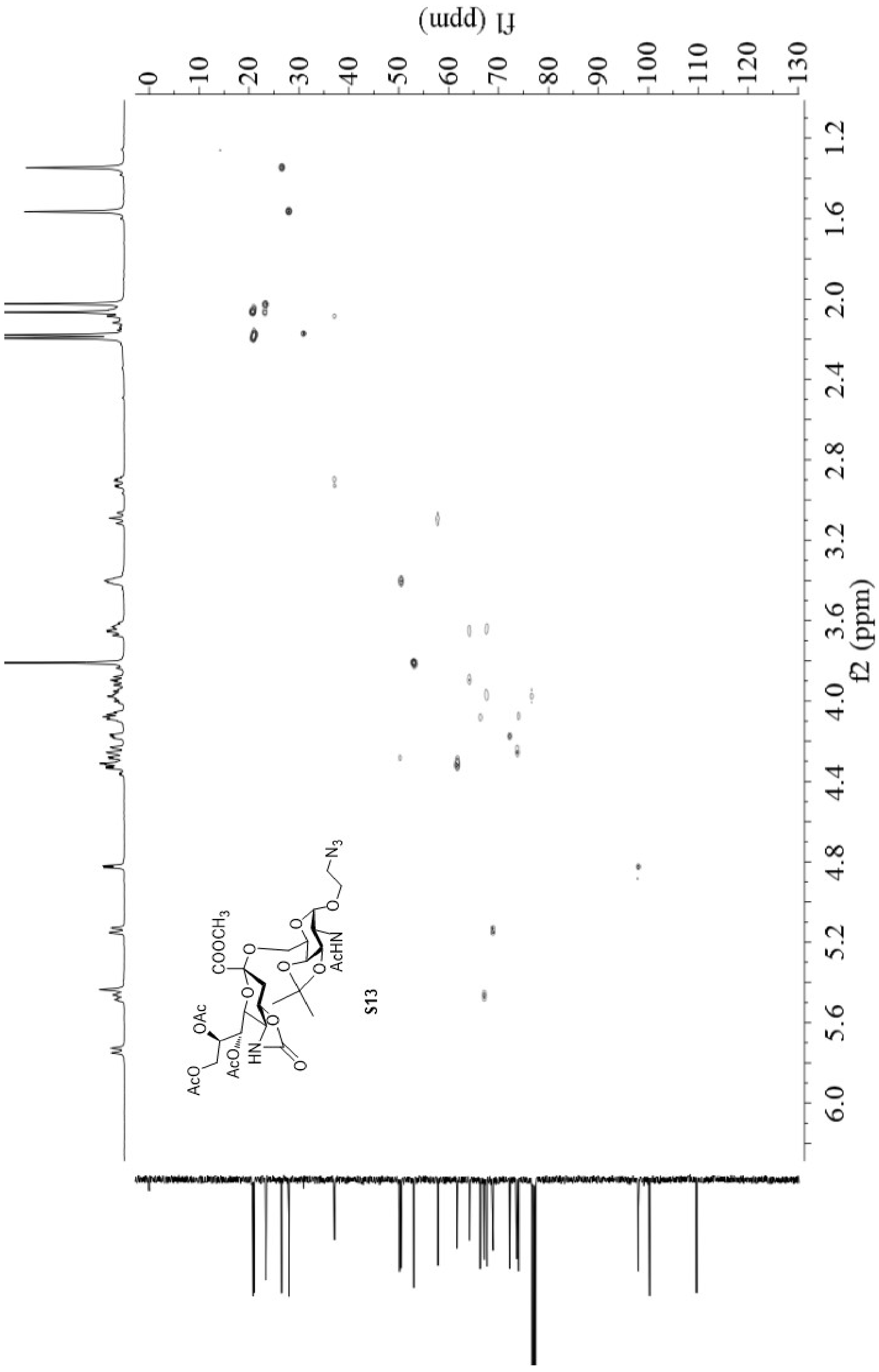

HSQC NMR Spectrum of compound **S13** ($CDCl_3$, 400 MHz)

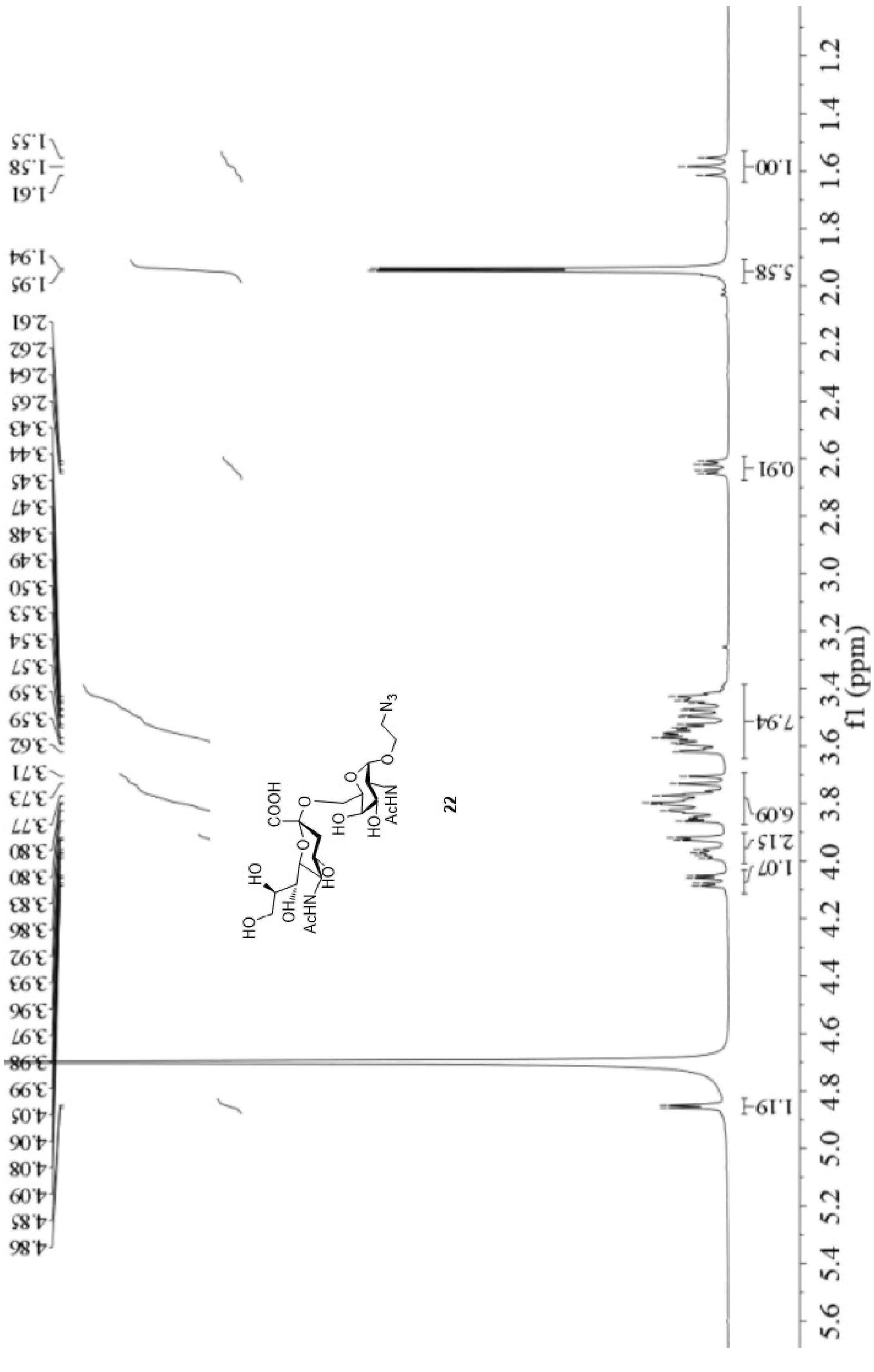

^{1}H NMR Spectrum of compound **22** (D_2O, 400 MHz)

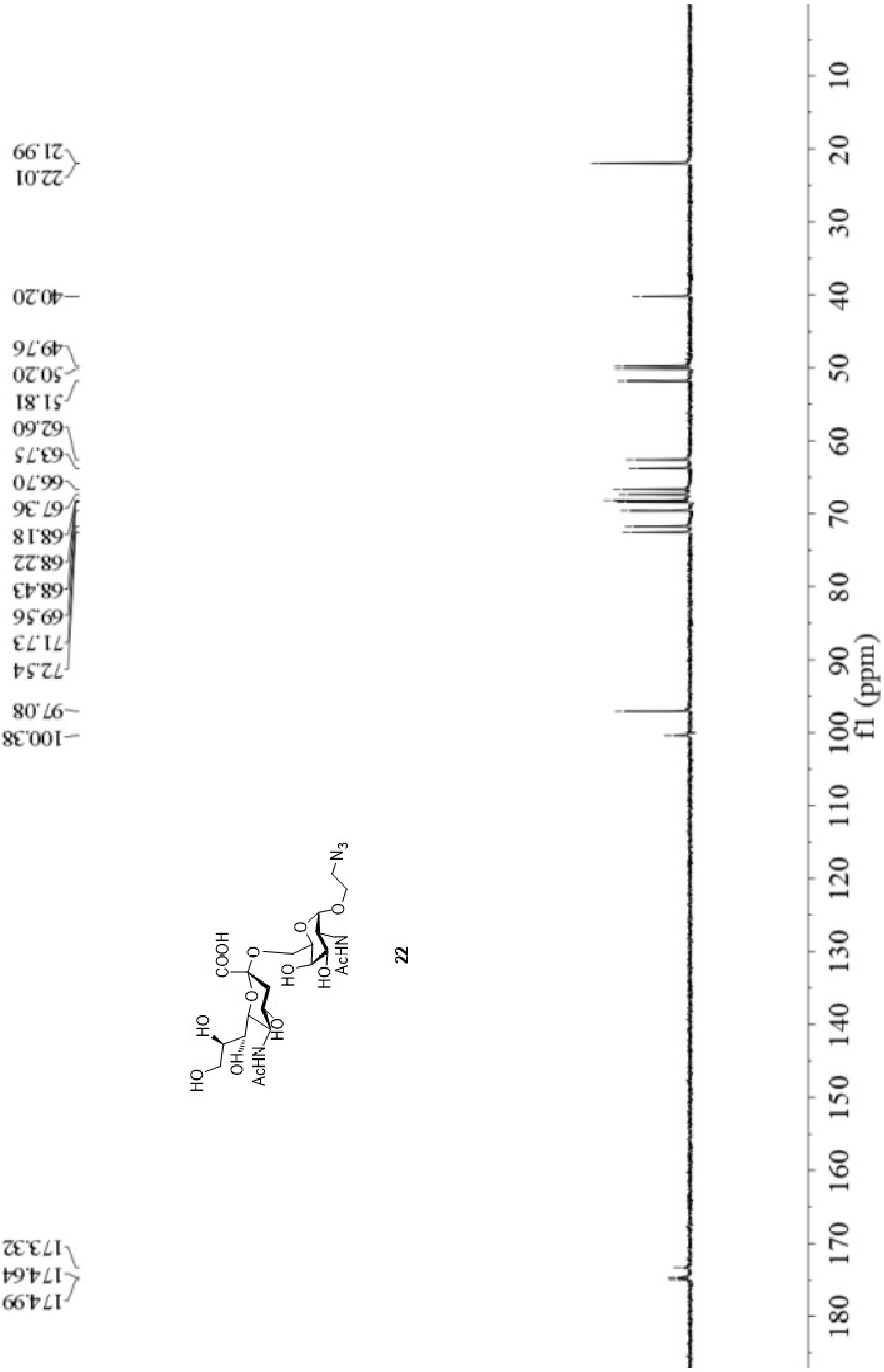

^{13}C NMR Spectrum of compound **22** (D_2O, 400 MHz)

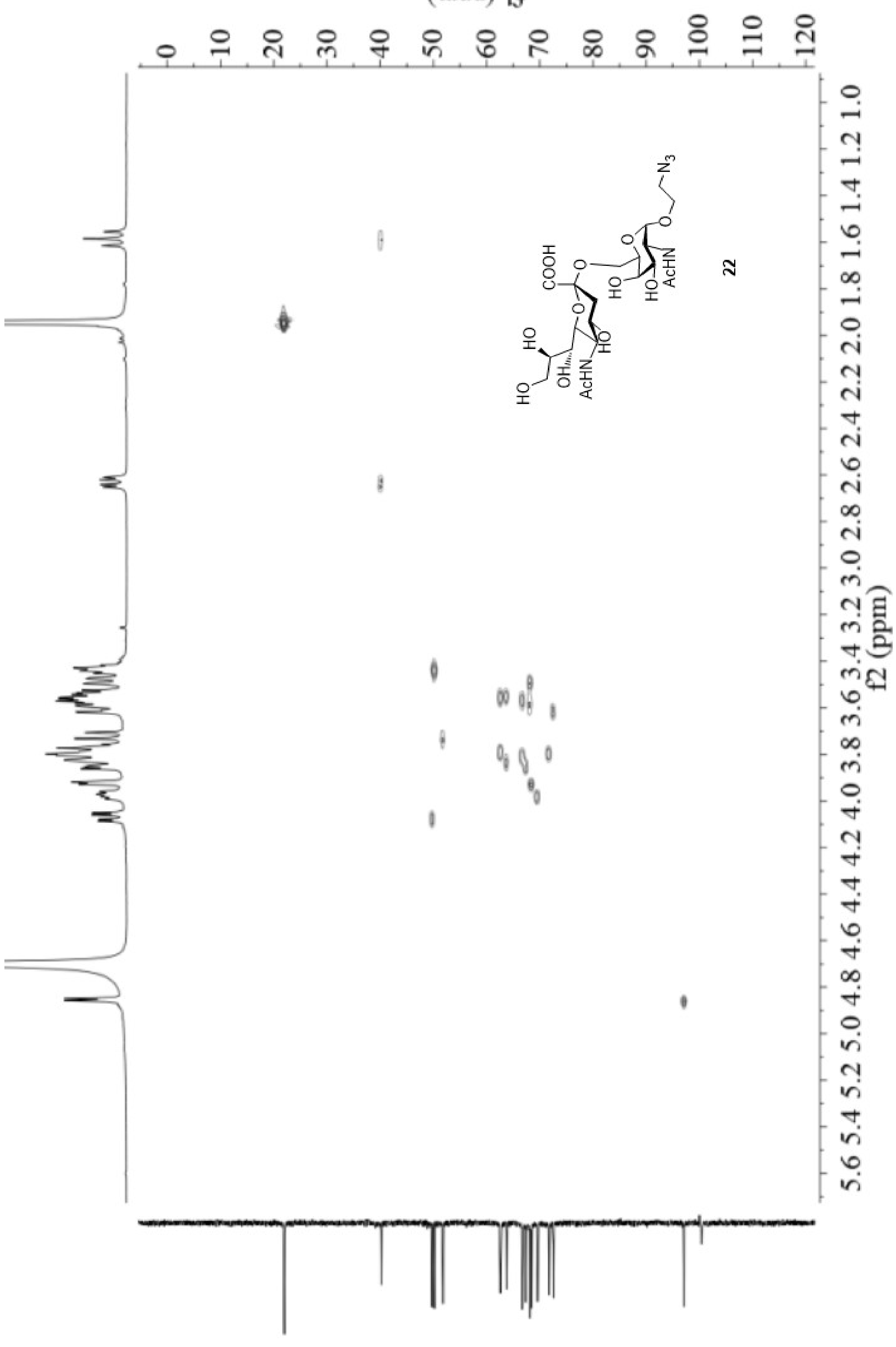

HSQC Spectrum of compound **22** (D_2O, 400 MHz)

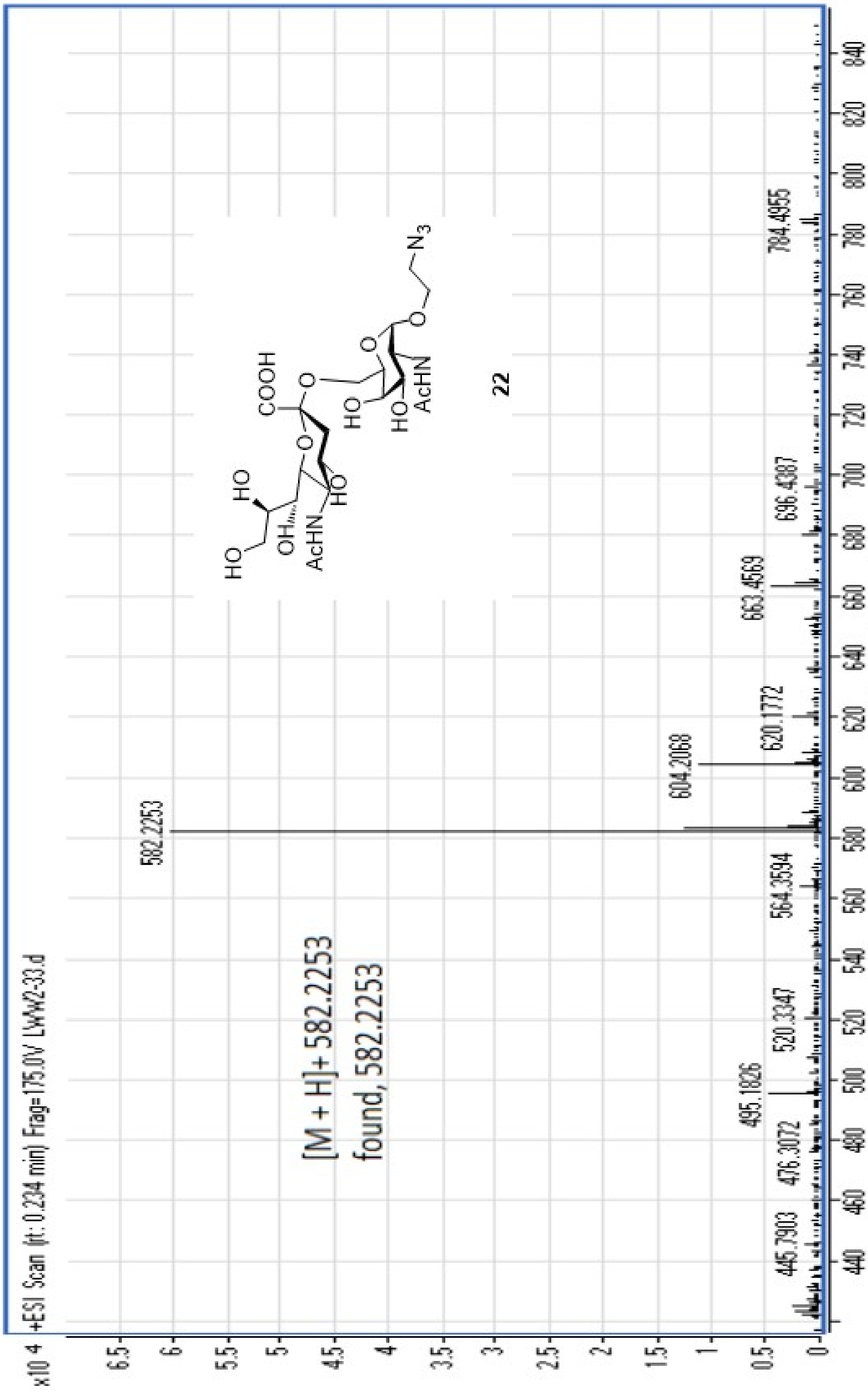

HR-ESI-MS spectrum of conjugate **22**

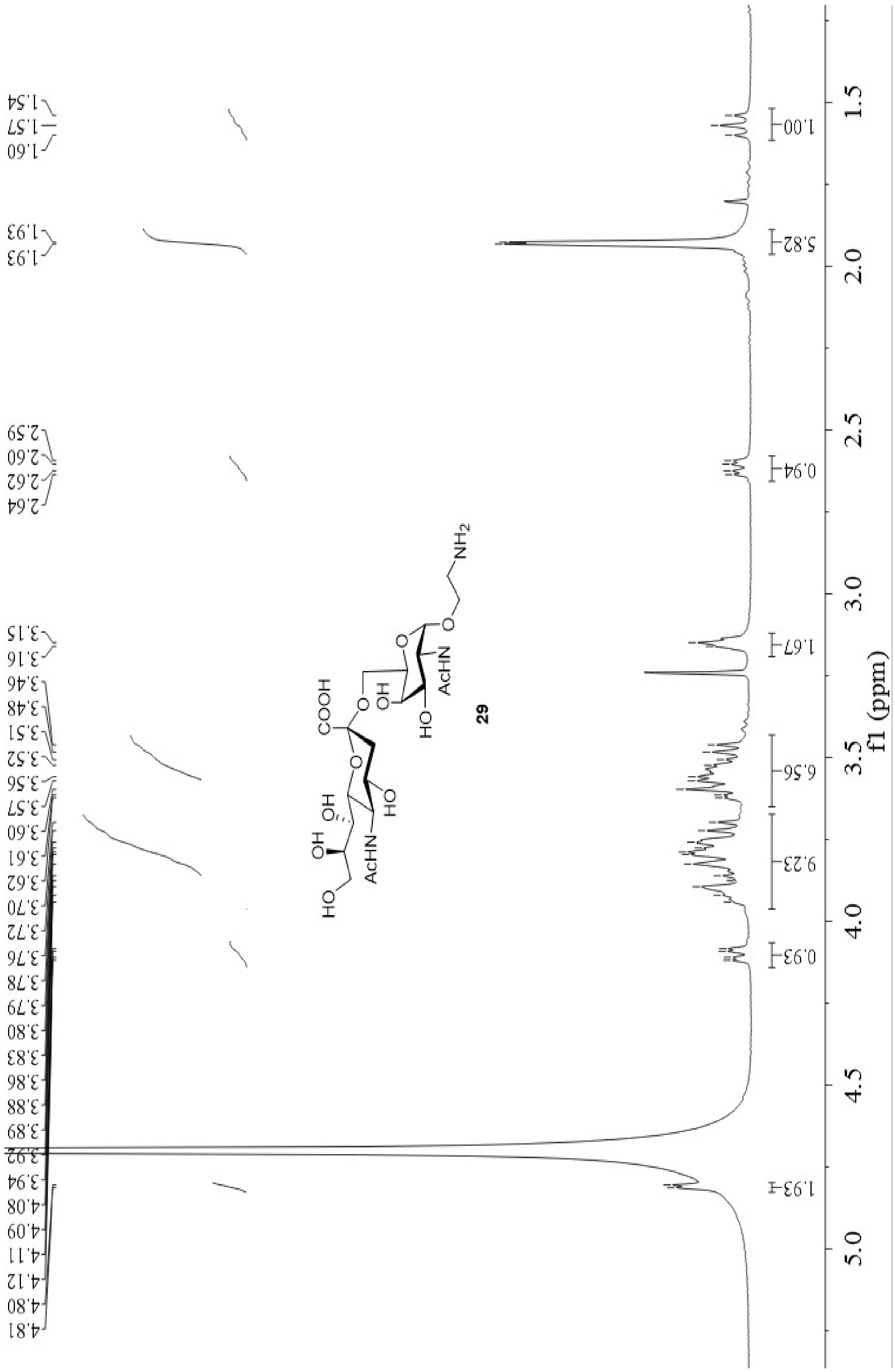

^{1}H NMR Spectrum of compound **29** (D_2O, 400 MHz)

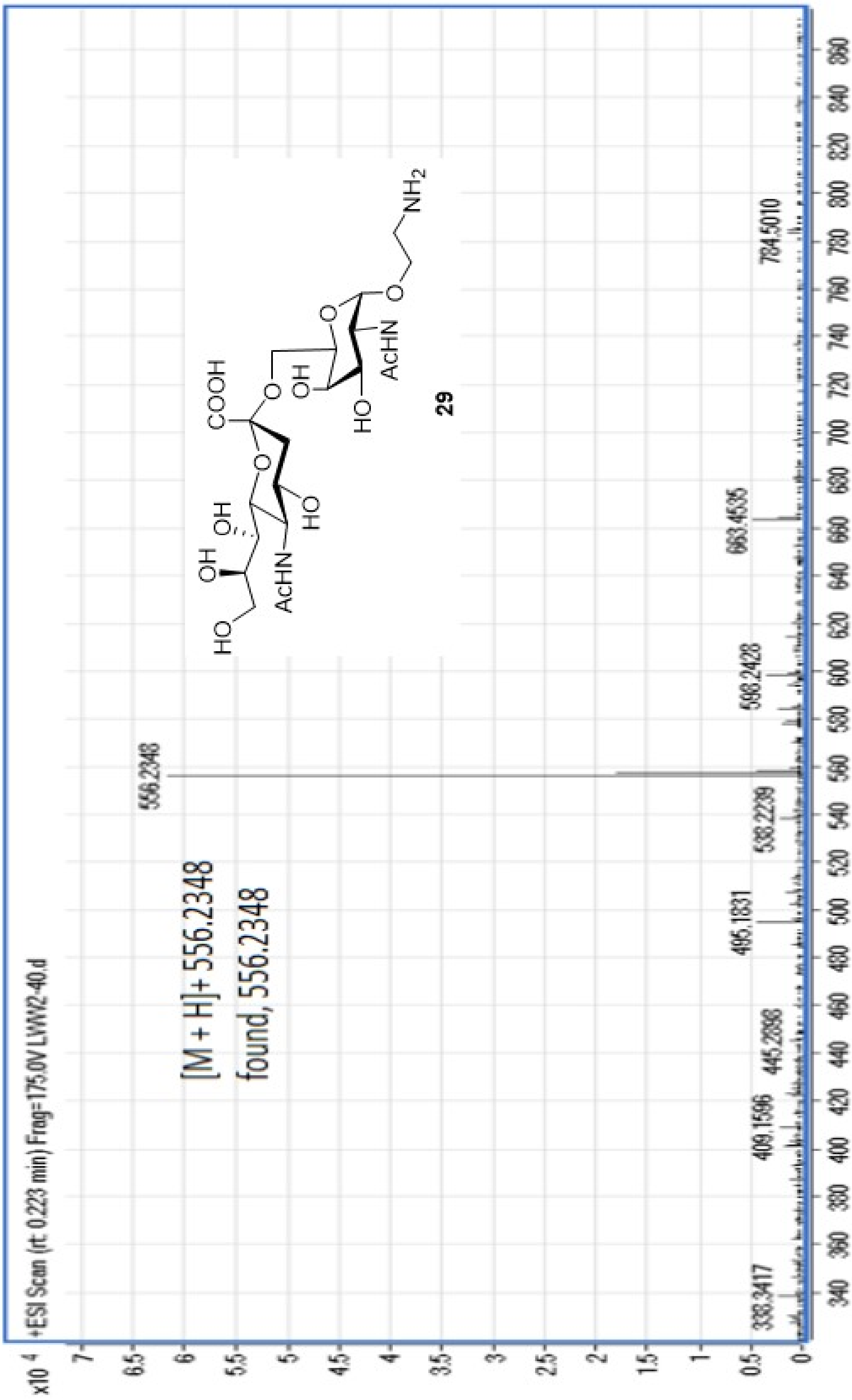

HR-ESI-MS spectrum of conjugate **29**

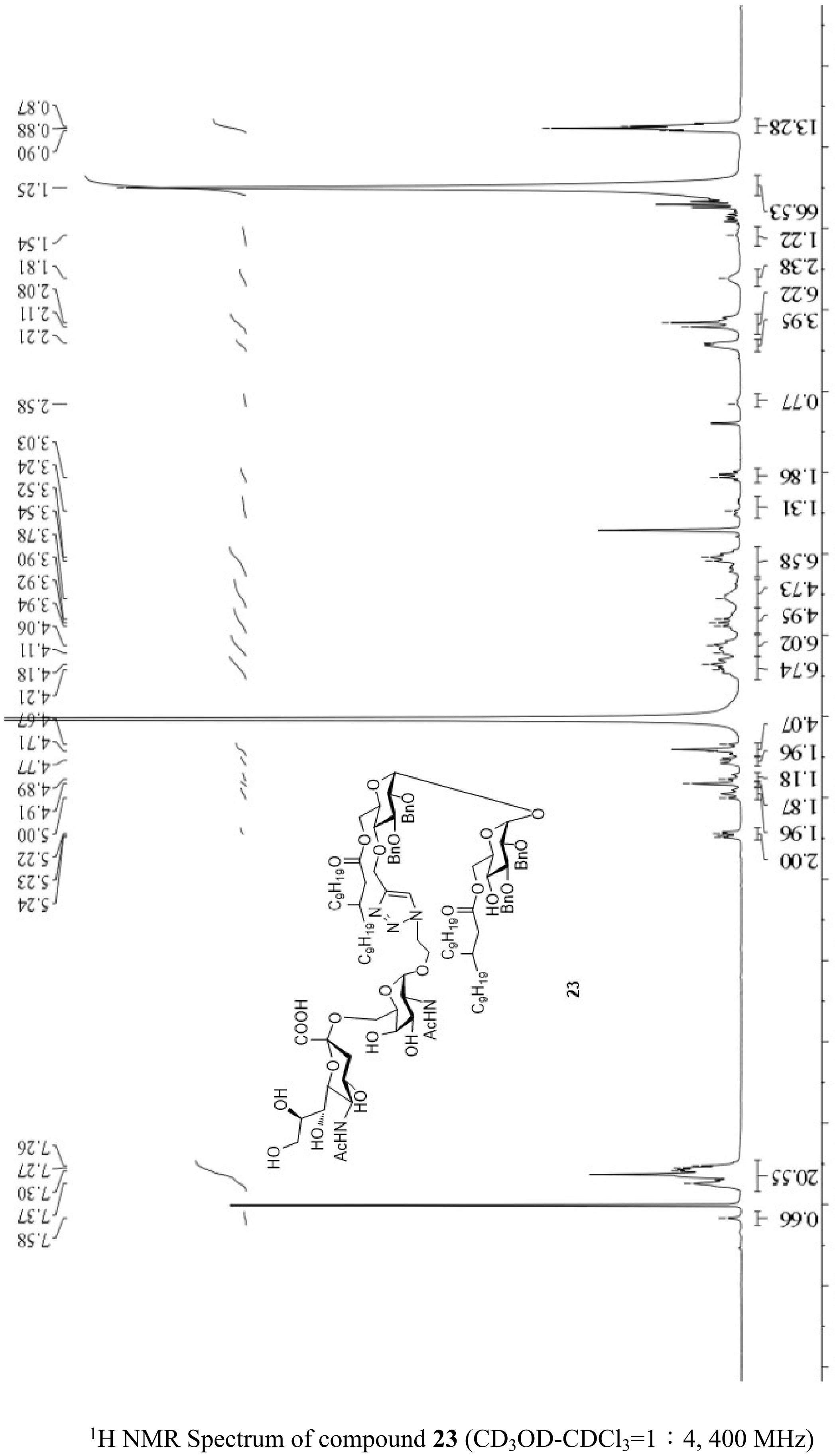

^{1}H NMR Spectrum of compound **23** (CD_3OD-$CDCl_3$=1：4, 400 MHz)

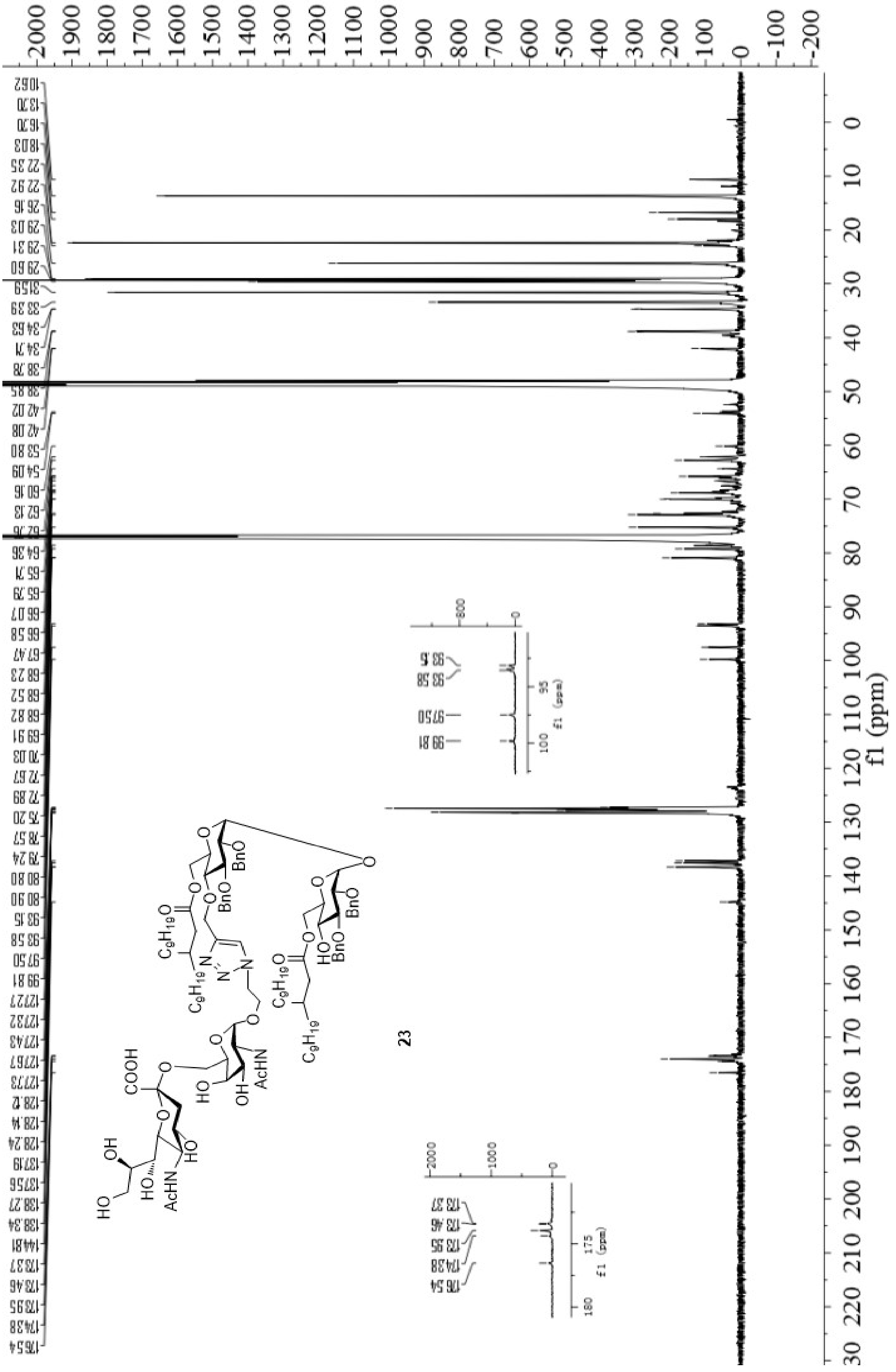

^{13}C NMR Spectrum of compound **23** (CD_3OD-$CDCl_3$=1：4, 400 MHz)

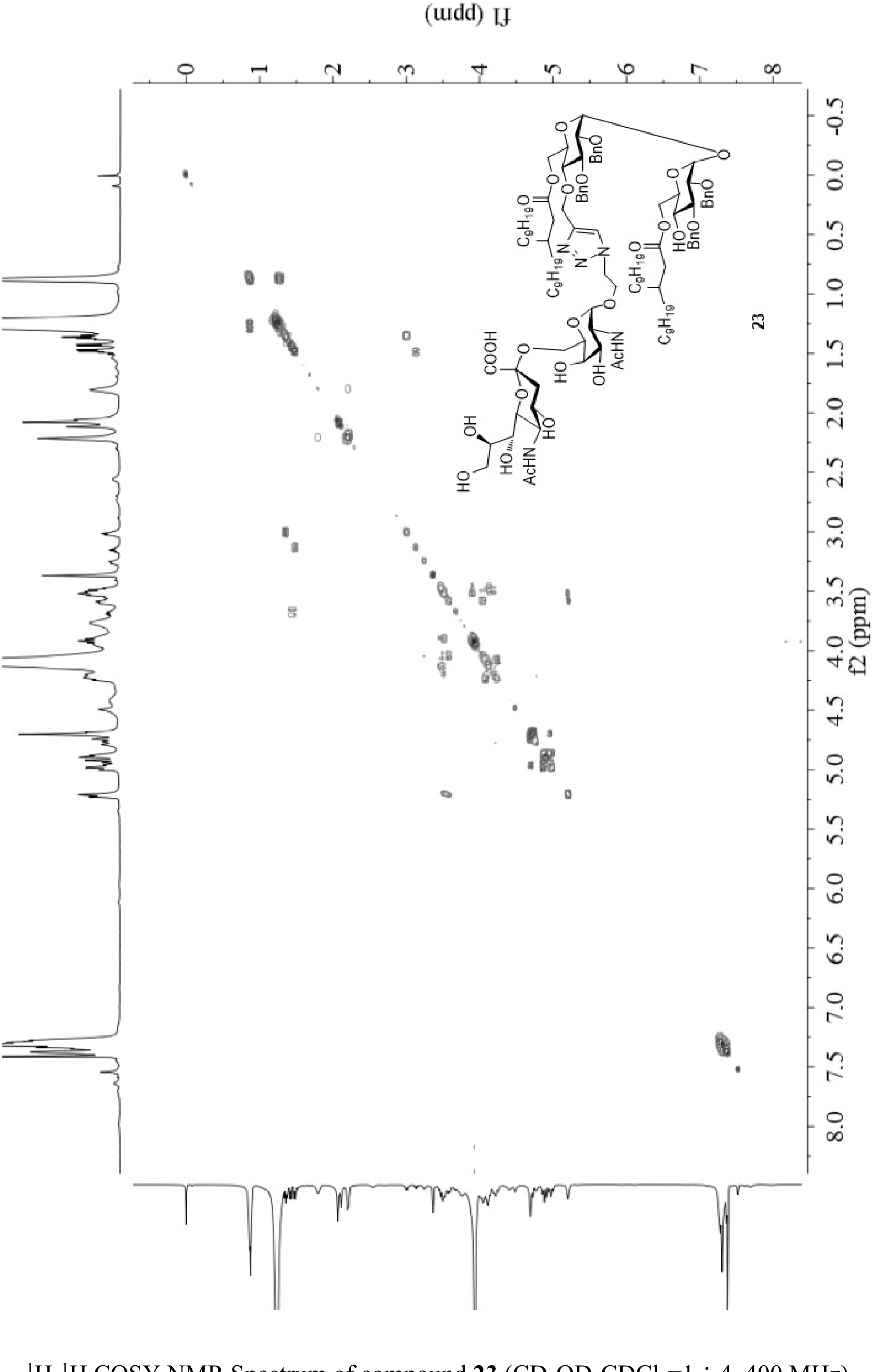

^1H-^1H COSY NMR Spectrum of compound **23** (CD_3OD-$CDCl_3$=1：4, 400 MHz)

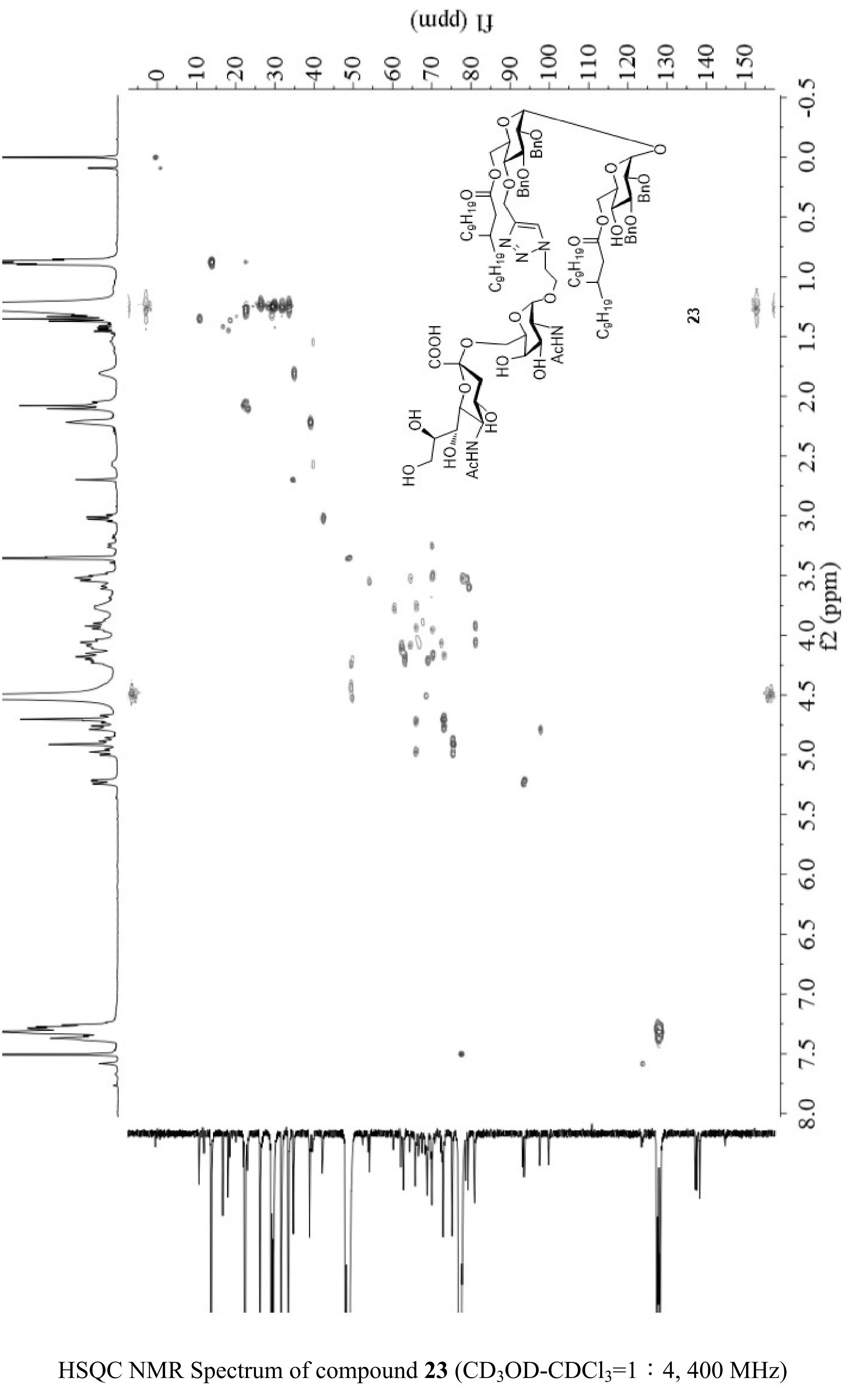

HSQC NMR Spectrum of compound **23** (CD_3OD-$CDCl_3$=1：4, 400 MHz)

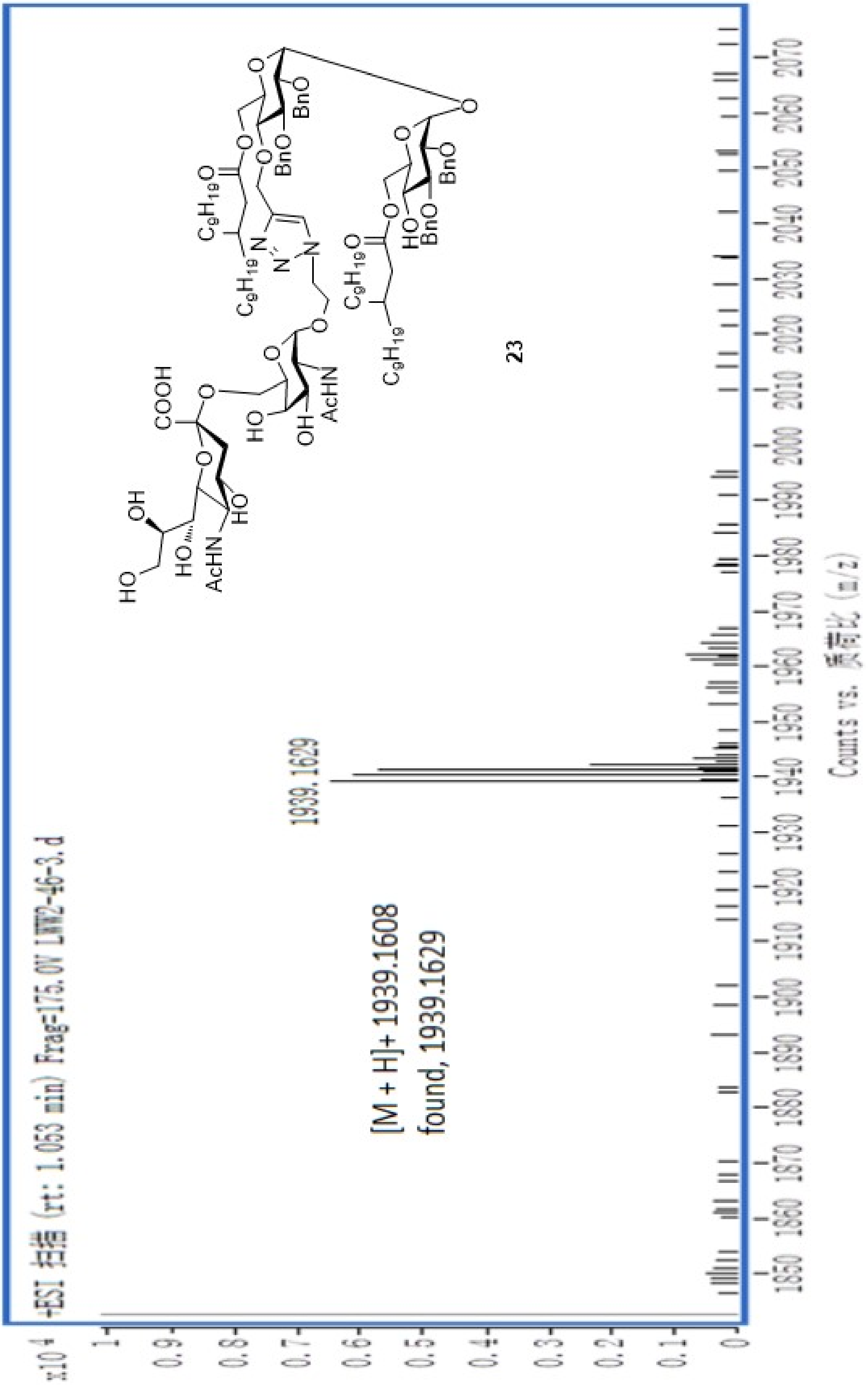

HR-ESI-MS spectrum of conjugate **23**

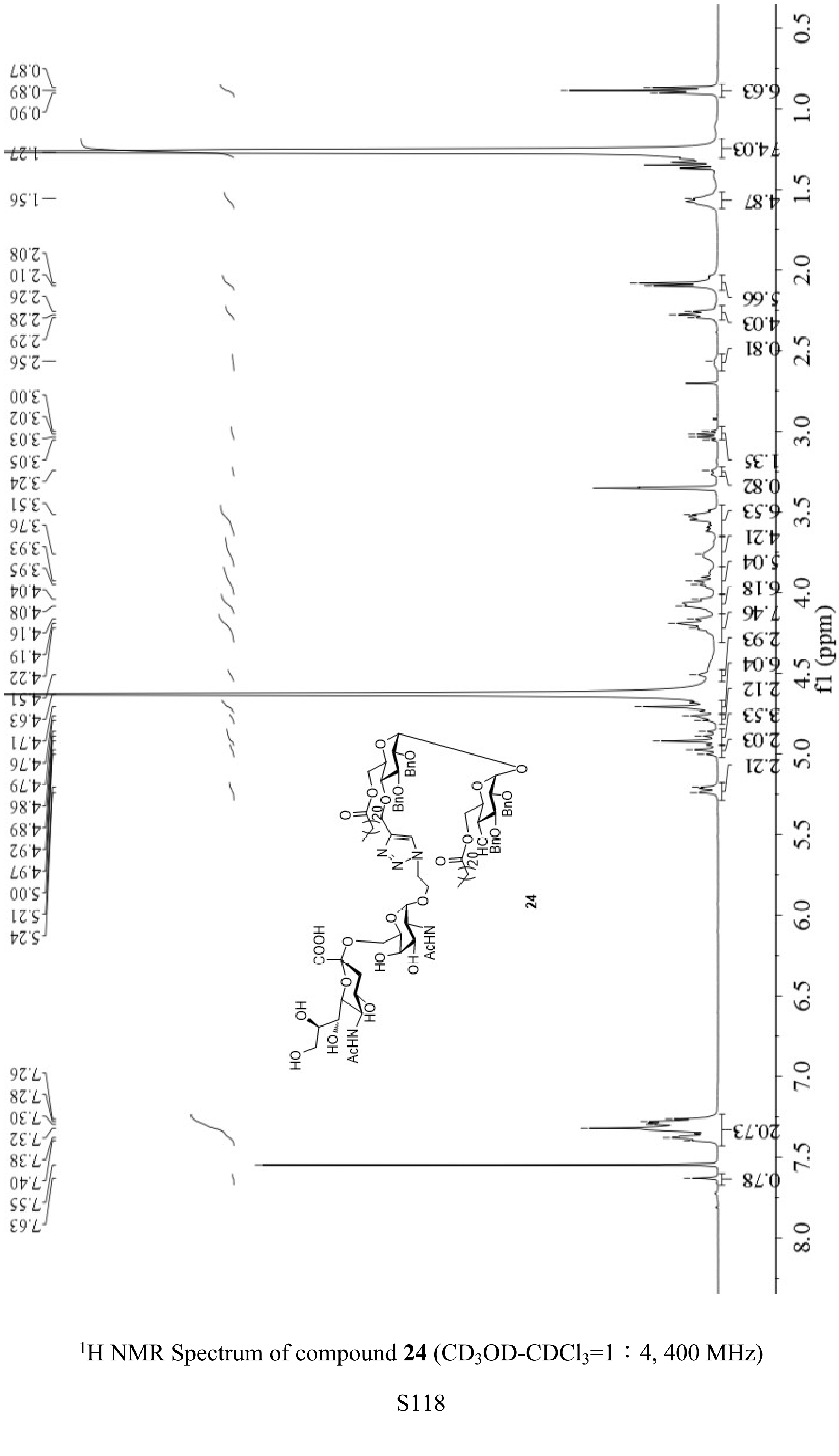

^{1}H NMR Spectrum of compound **24** (CD_3OD-$CDCl_3$=1：4, 400 MHz)

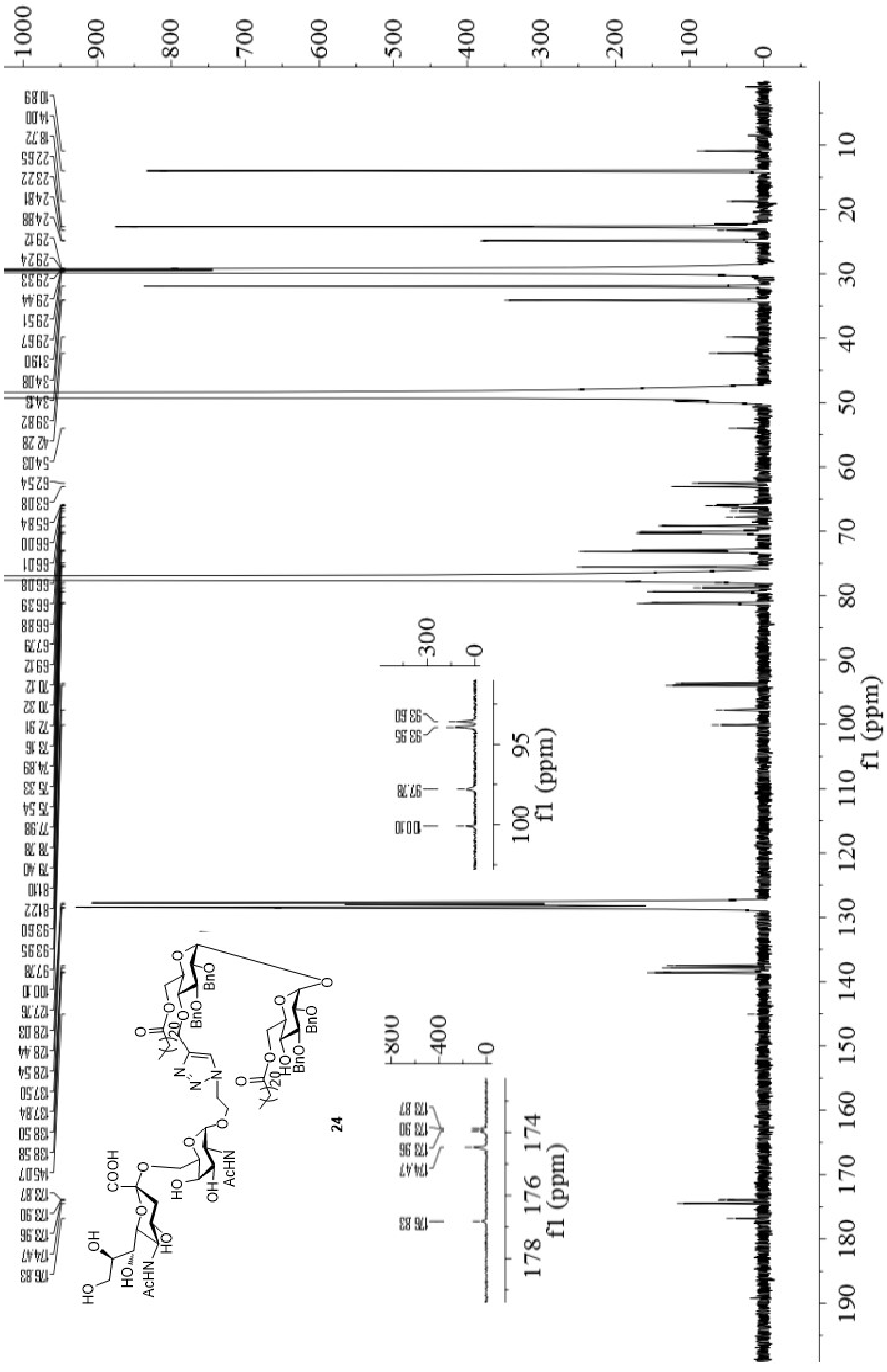

^{13}C NMR Spectrum of compound **24** (CD_3OD-$CDCl_3$=1：4, 400 MHz)

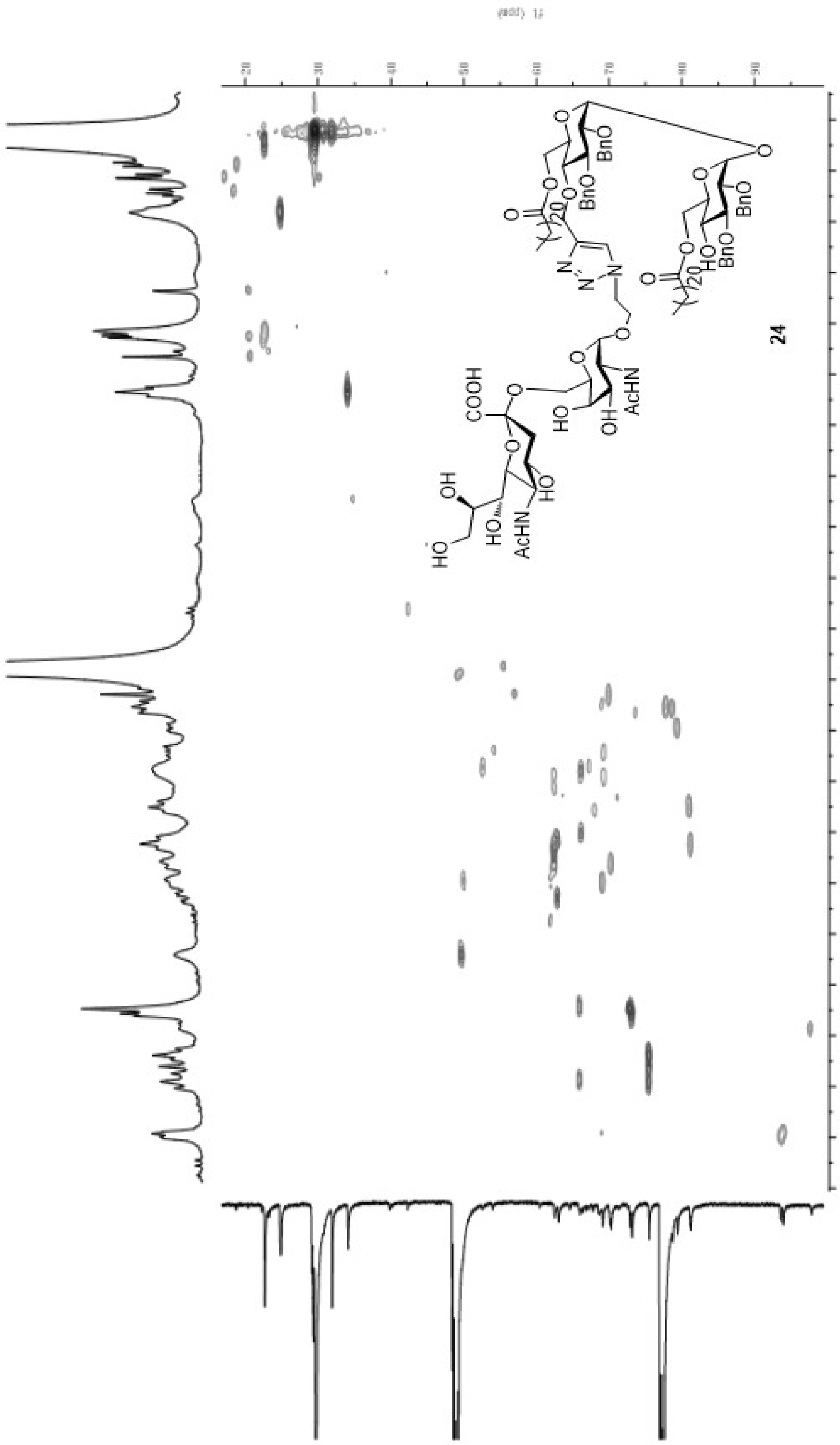

^{1}H-^{1}H COSY NMR Spectrum of compound **24** (CD_3OD-$CDCl_3$=1：4, 400 MHz)

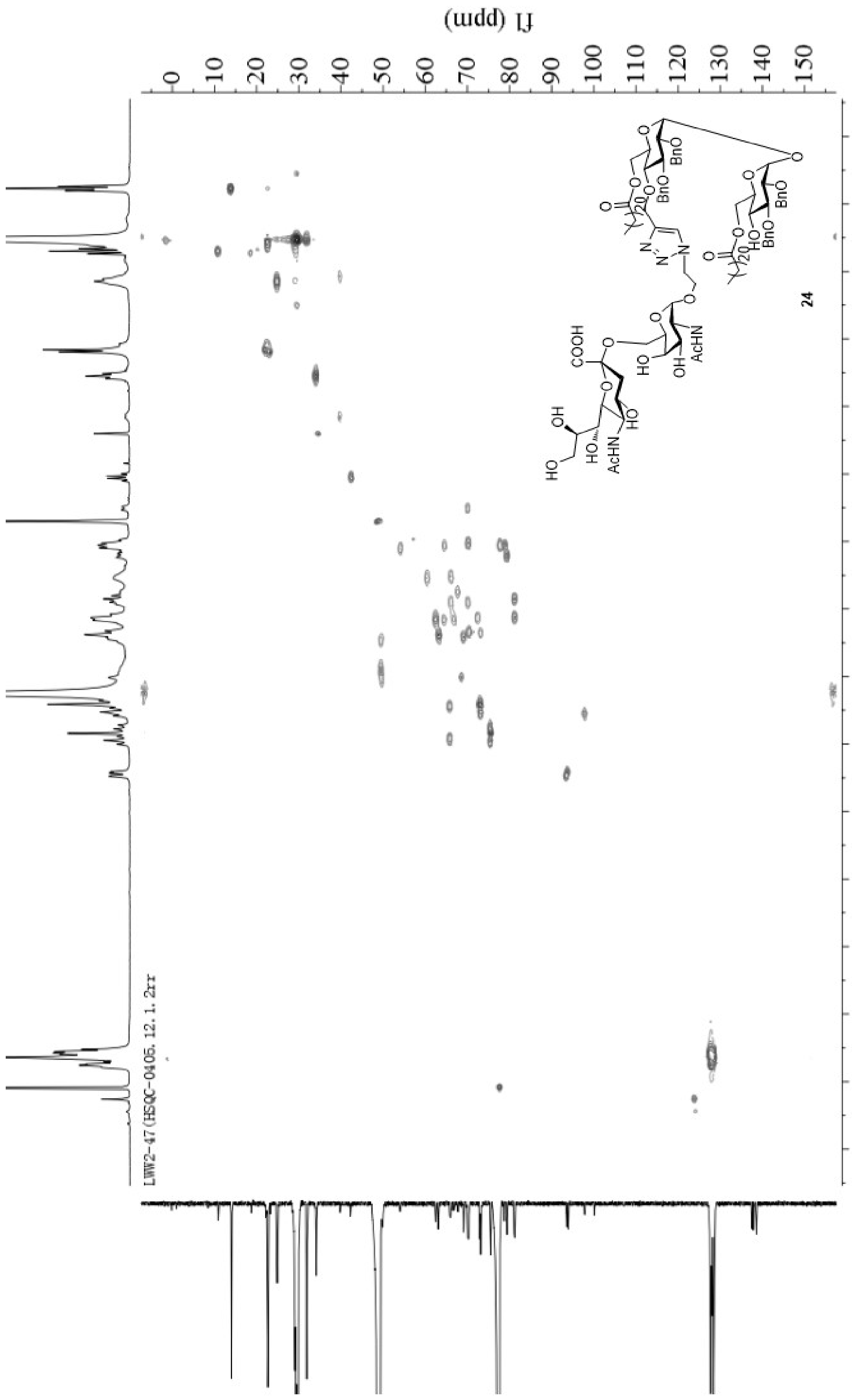

HSQC Spectrum of compound **24** (CD_3OD-$CDCl_3$=1：4, 400 MHz)

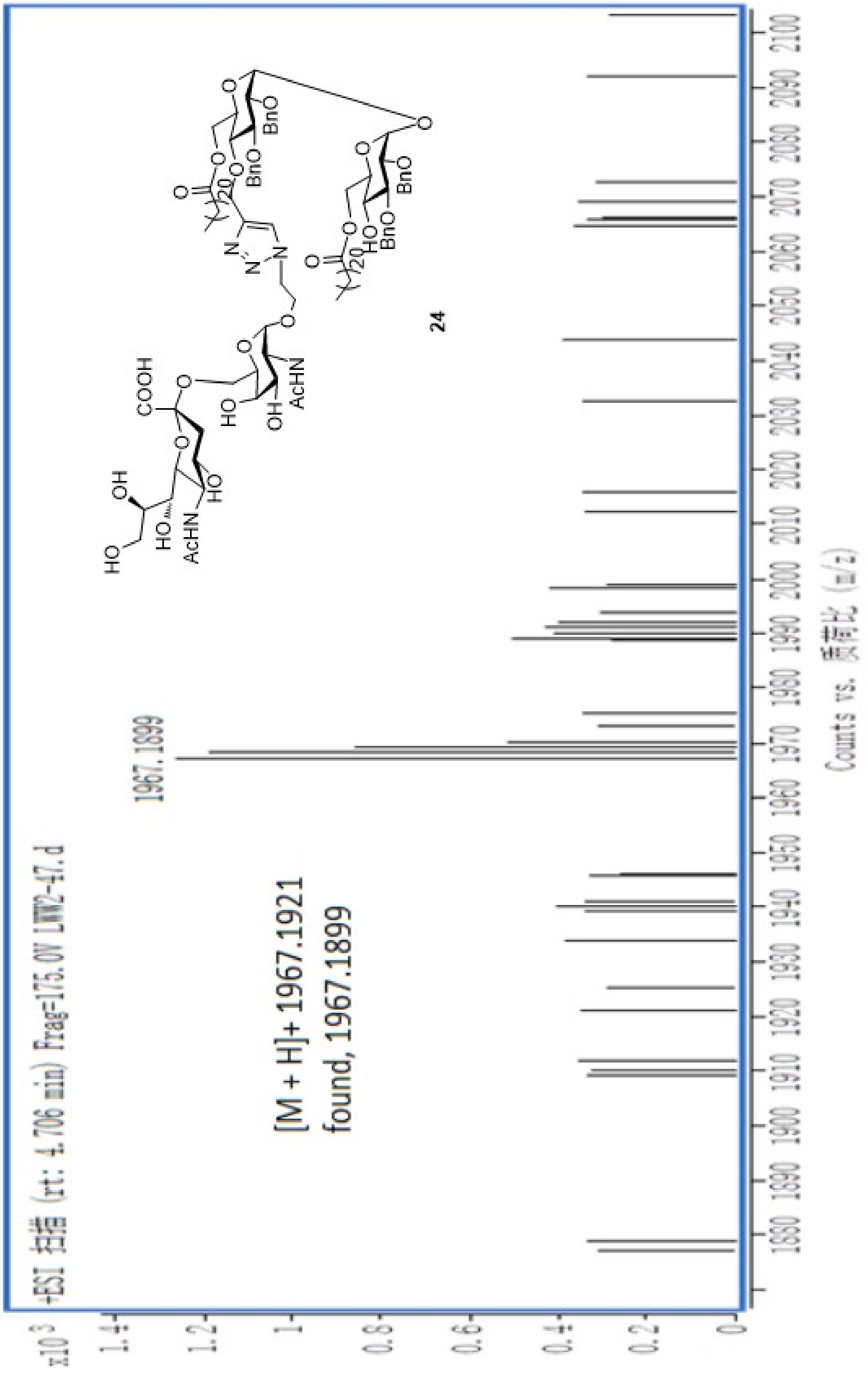

HR-ESI-MS spectrum of conjugate **24**

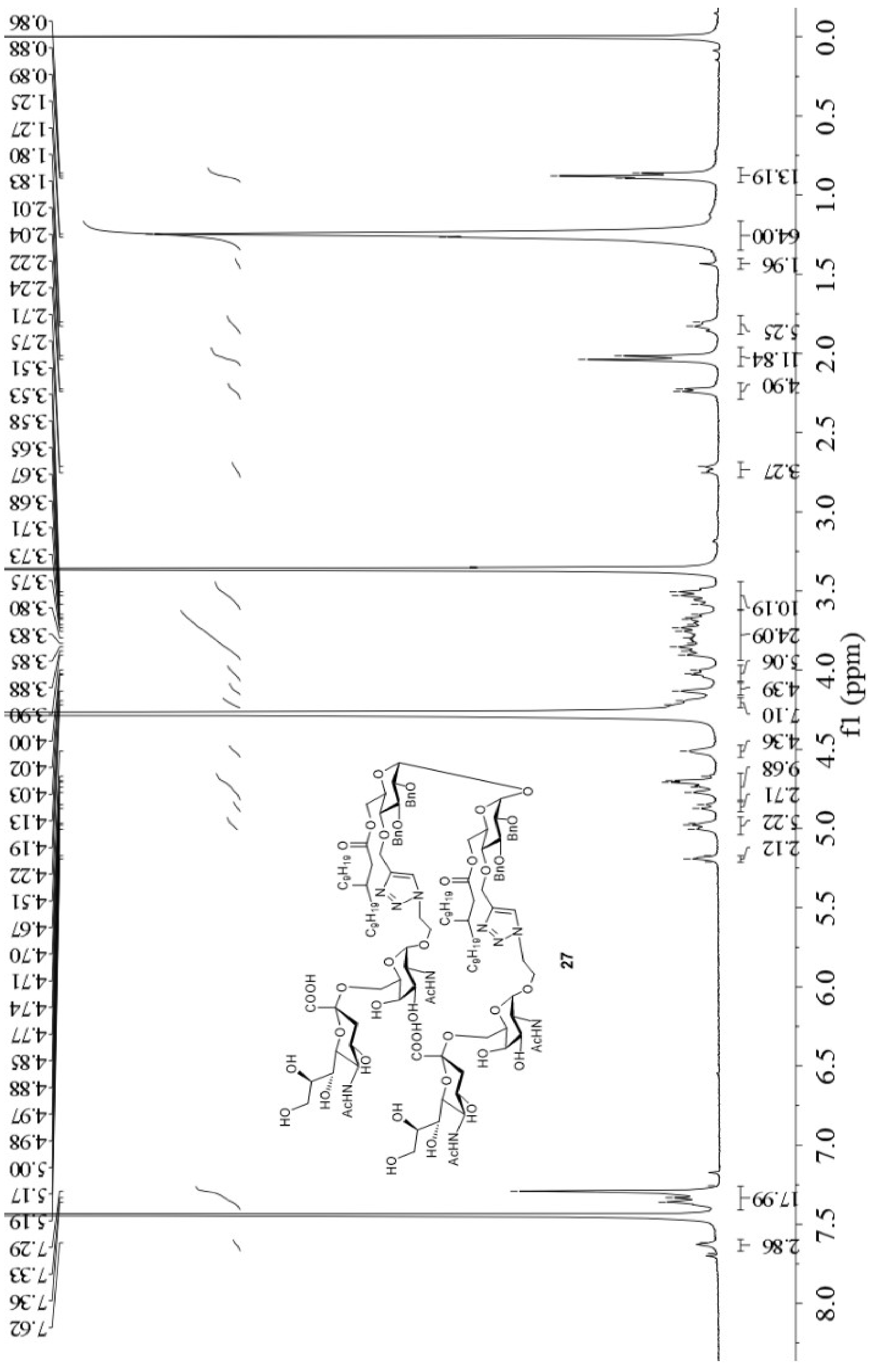

^{1}H NMR Spectrum of compound **27** (MeOD: $CDCl_3$ = 1:6; 400 MHz)

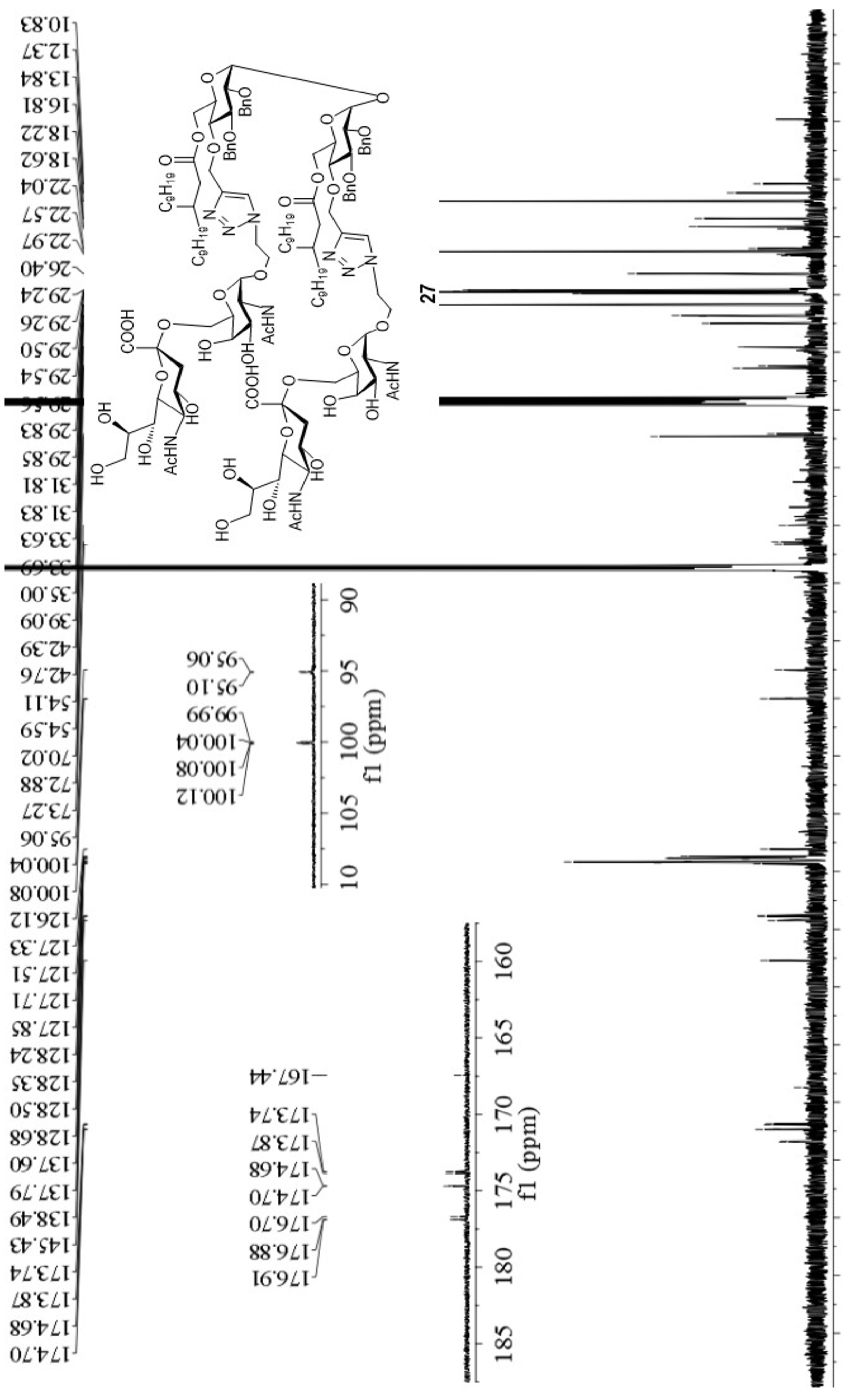

^{13}C NMR Spectrum of compound **27** (MeOD: $CDCl_3$ = 1:6; 400 MHz)

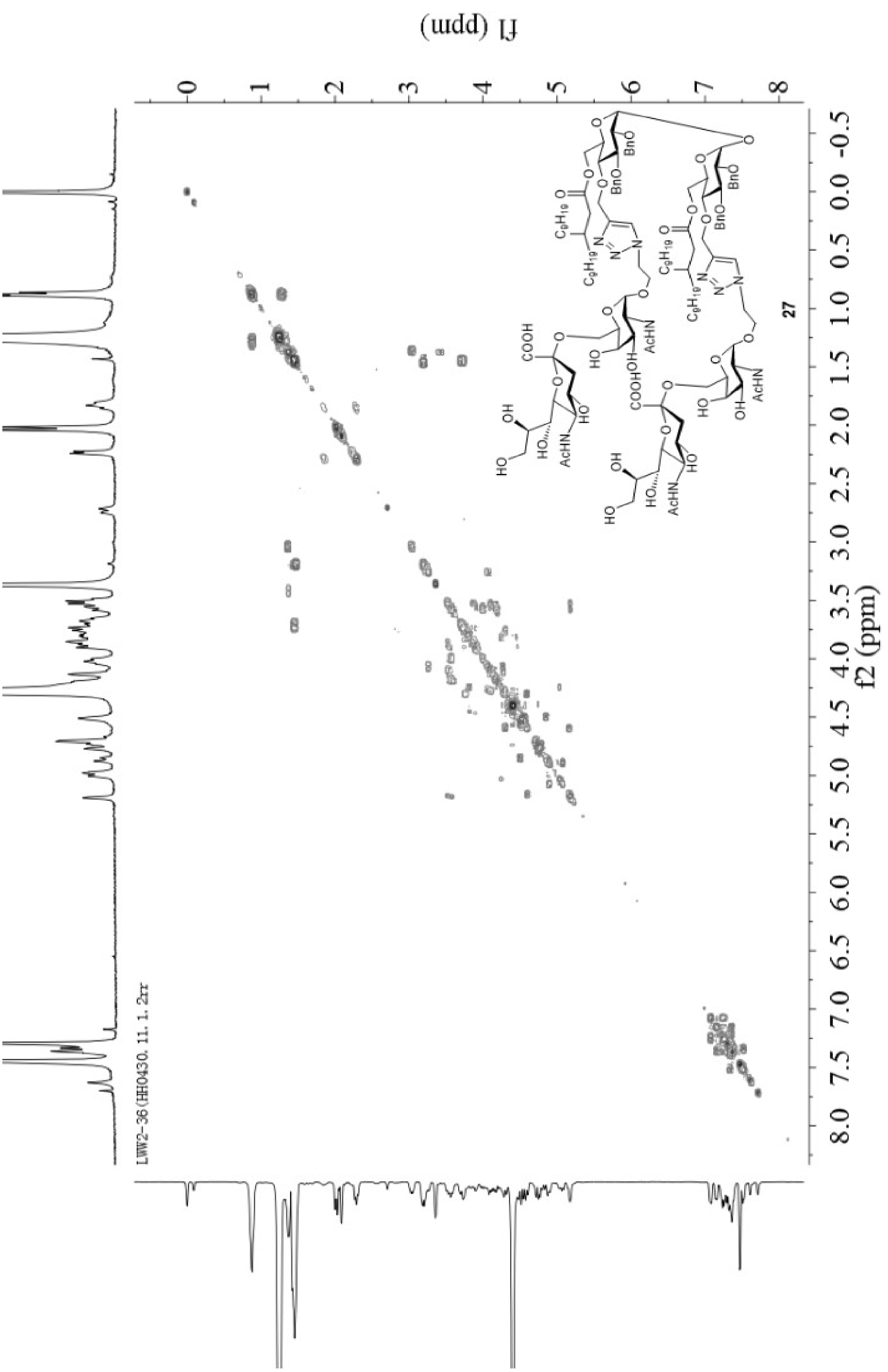

^{1}H-^{1}H COSY NMR Spectrum of compound **27** (MeOD: $CDCl_3$ = 1:6; 400 MHz)

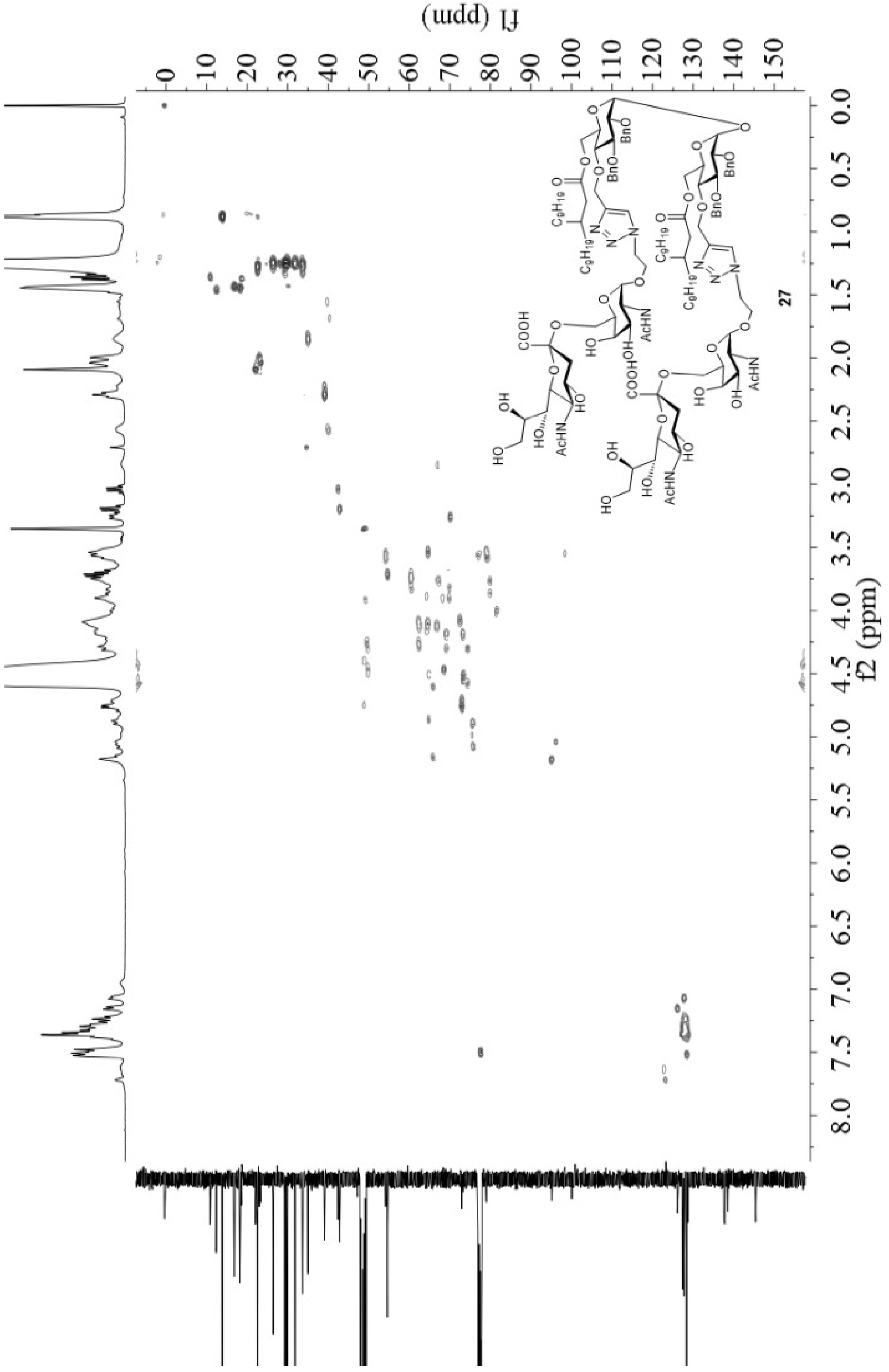

HSQC NMR Spectrum of compound **27** (MeOD: $CDCl_3$ = 1:6; 400 MHz)

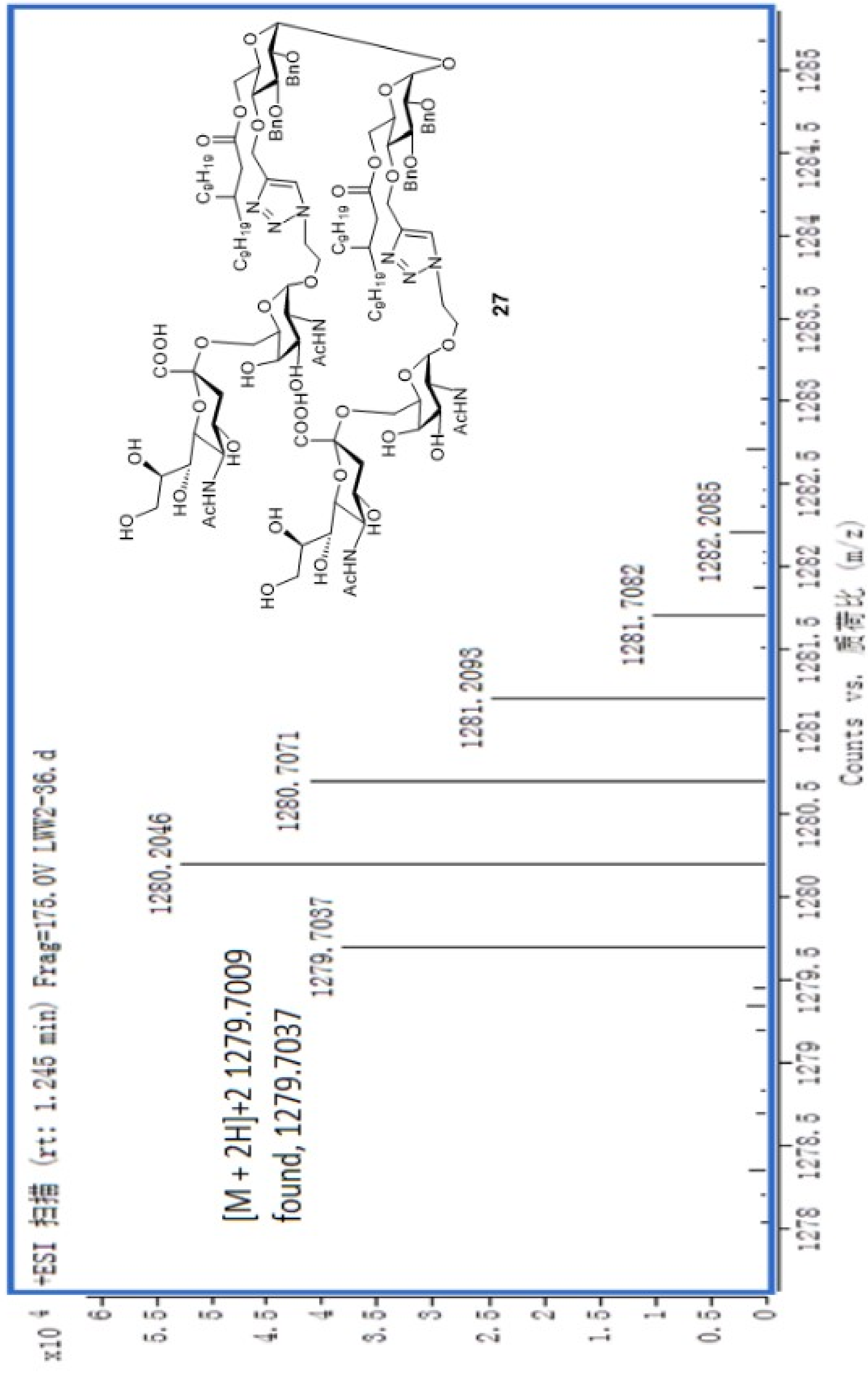

HR-ESI-MS spectrum of conjugate **27**

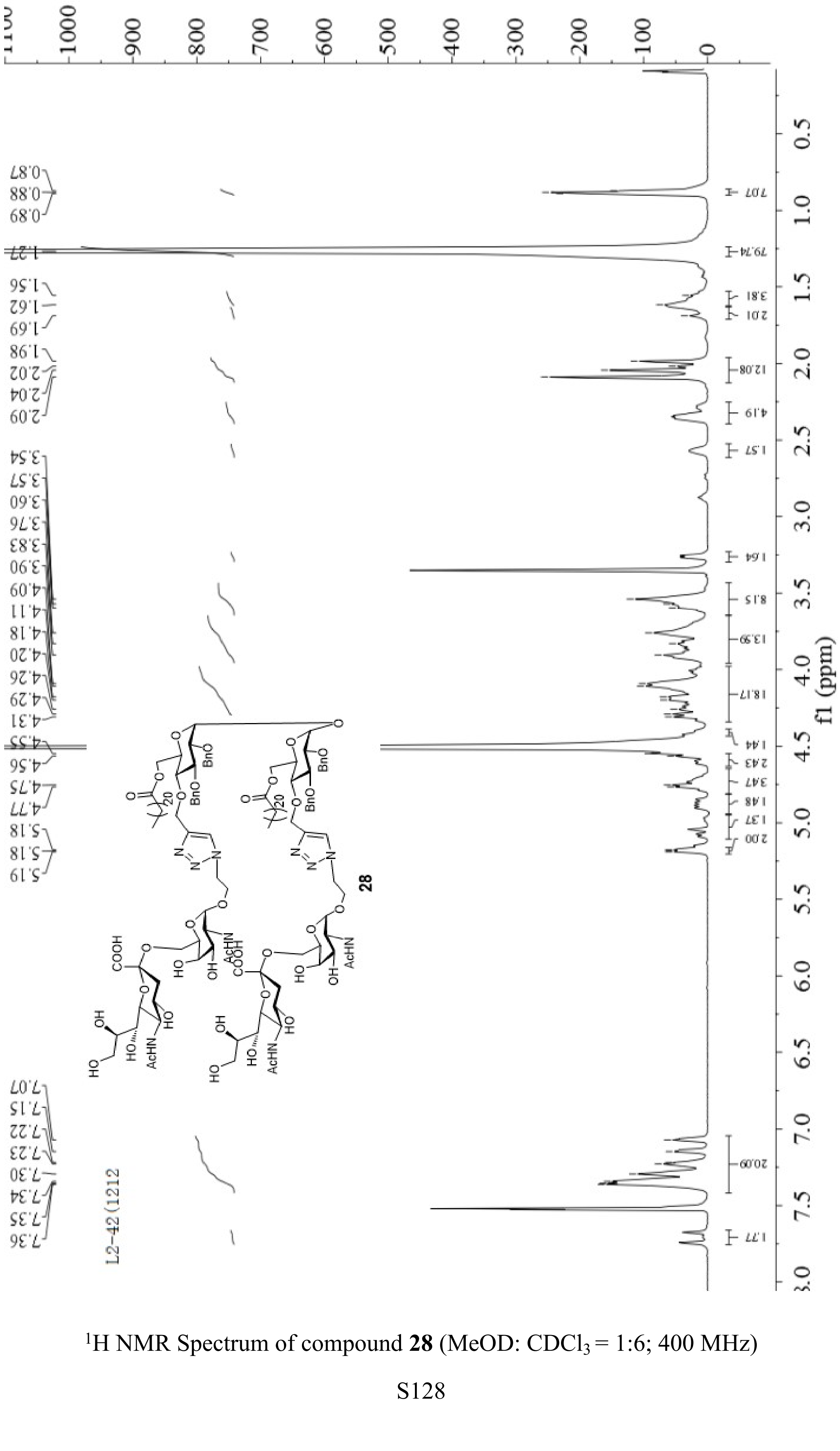

^{1}H NMR Spectrum of compound **28** (MeOD: $CDCl_3$ = 1:6; 400 MHz)

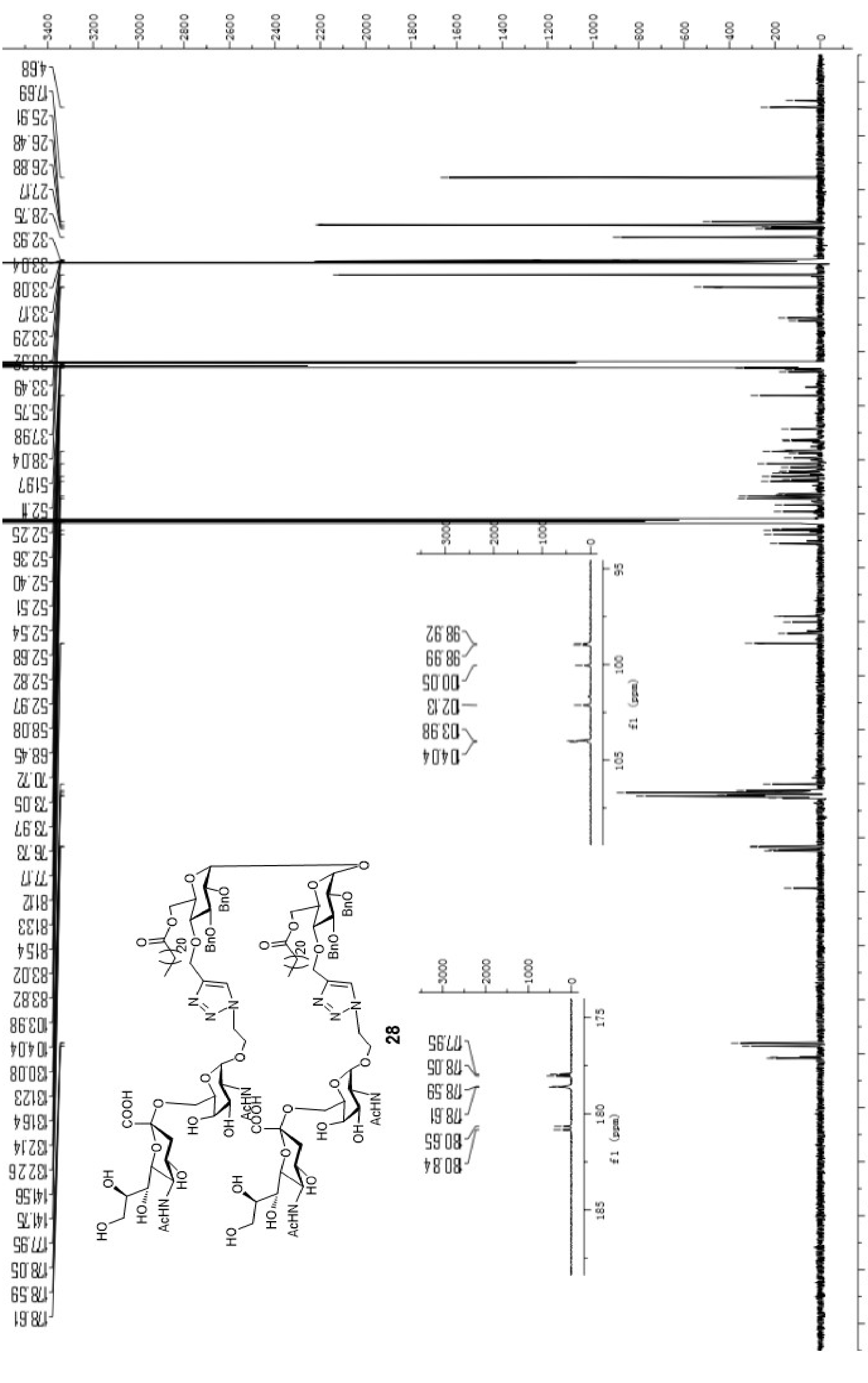

^{13}C NMR Spectrum of compound **28** (MeOD: $CDCl_3$ = 1:6; 400 MHz)

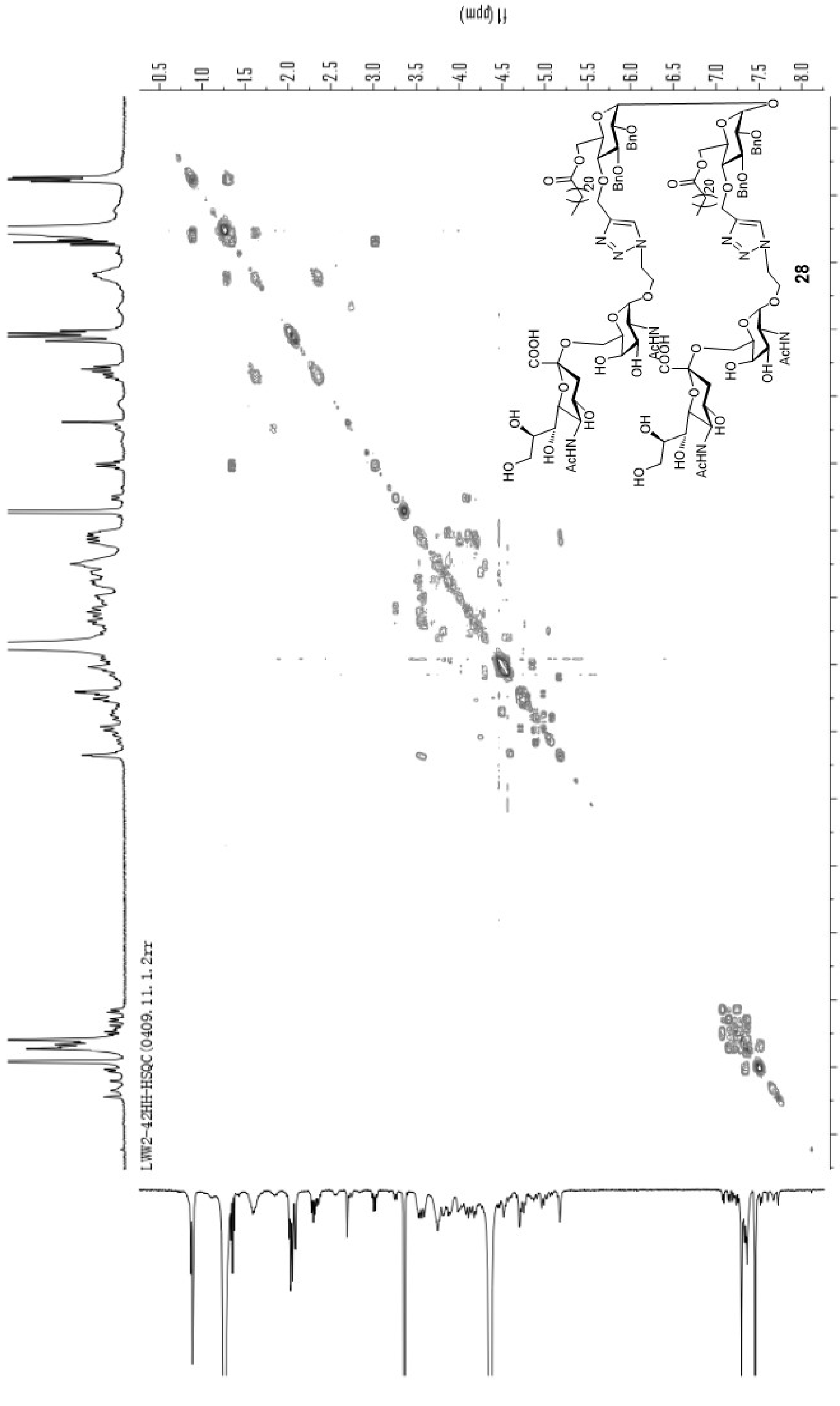

^{1}H-^{1}H COSY NMR Spectrum of compound **28** (MeOD: $CDCl_3$ = 1:6; 400 MHz)

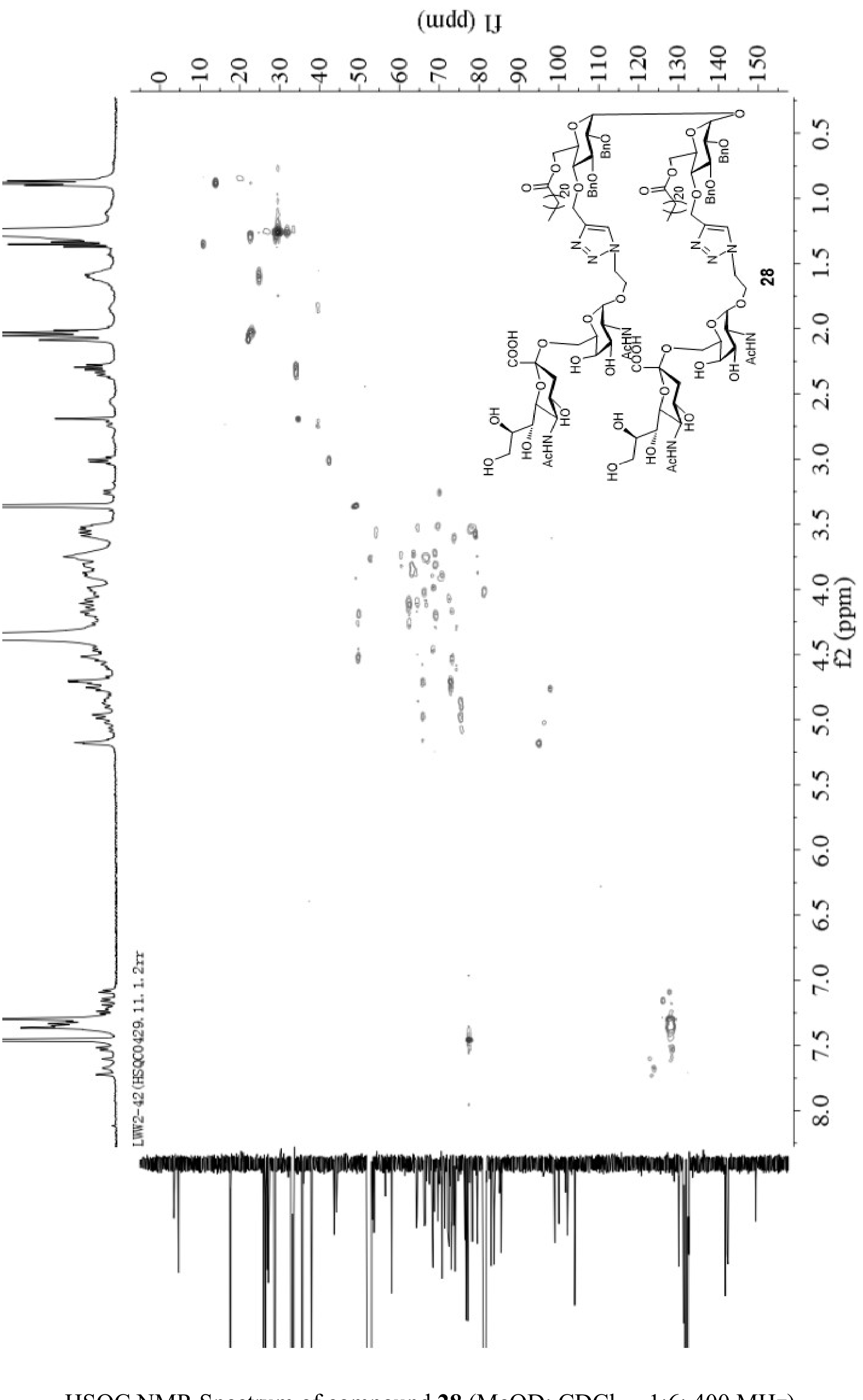

HSQC NMR Spectrum of compound **28** (MeOD: $CDCl_3$ = 1:6; 400 MHz)

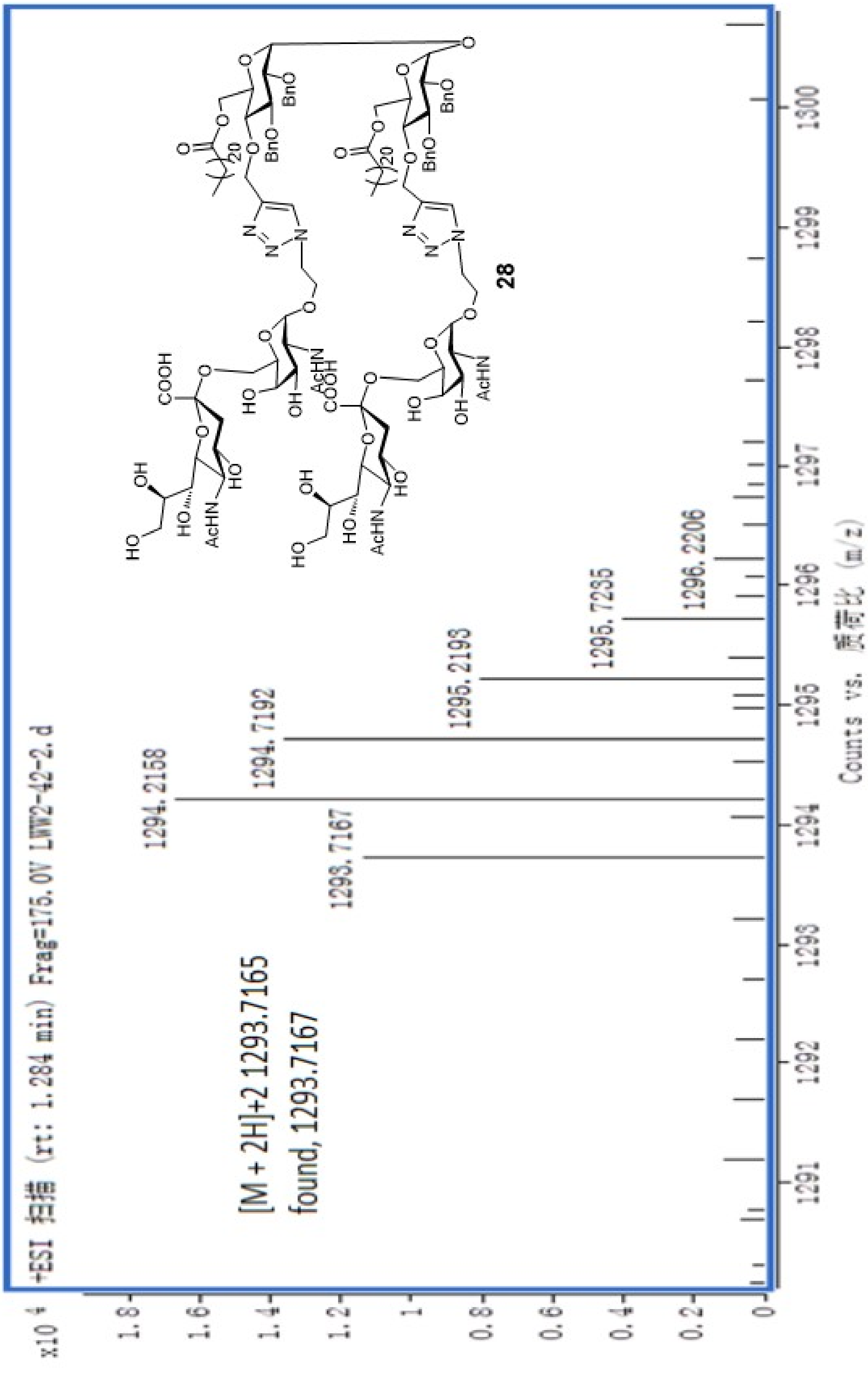

HR-ESI-MS spectrum of conjugate **28**

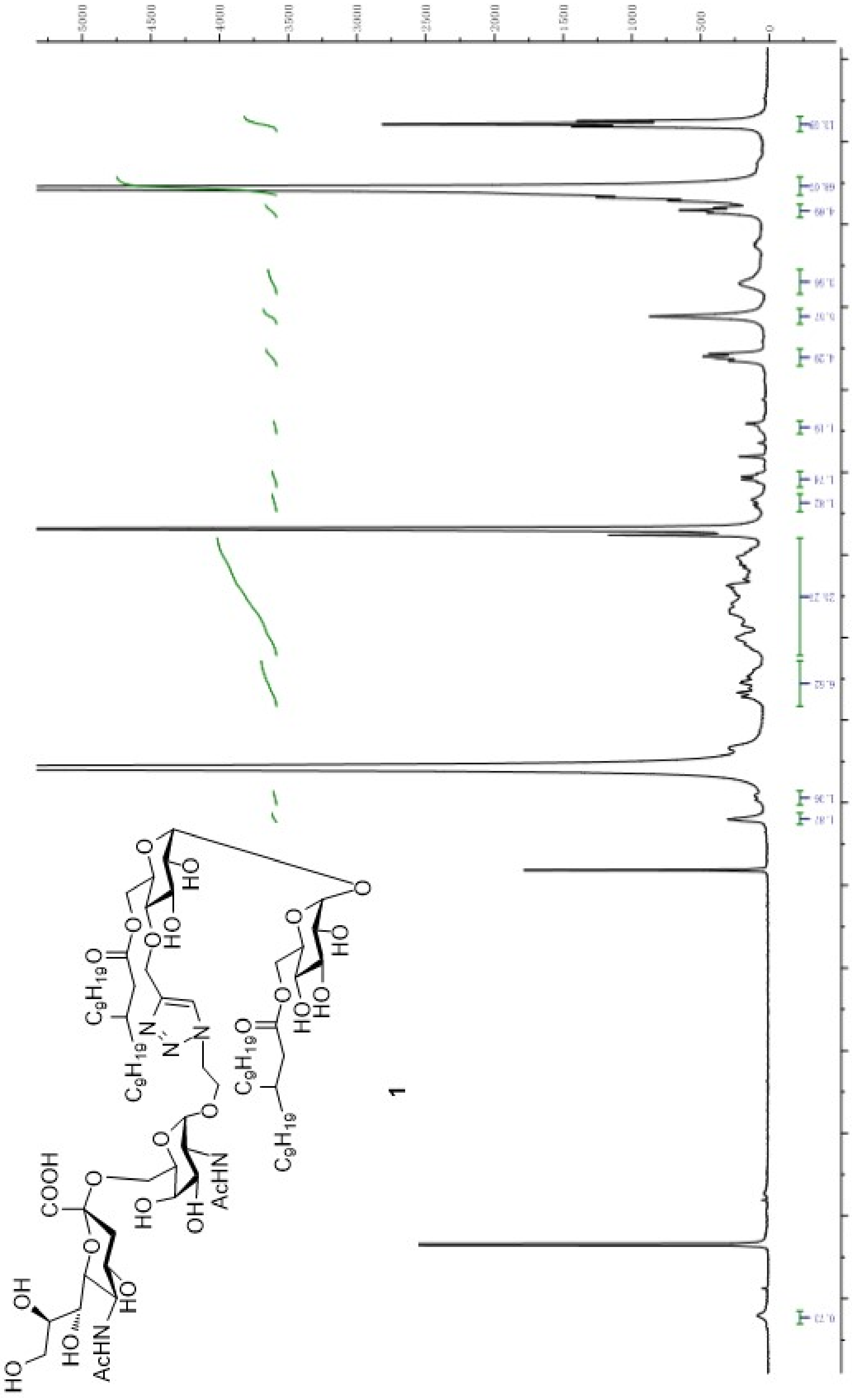

^{1}H NMR Spectrum of compound **1** (CD_3OD-$CDCl_3$=1：4, 400 MHz)

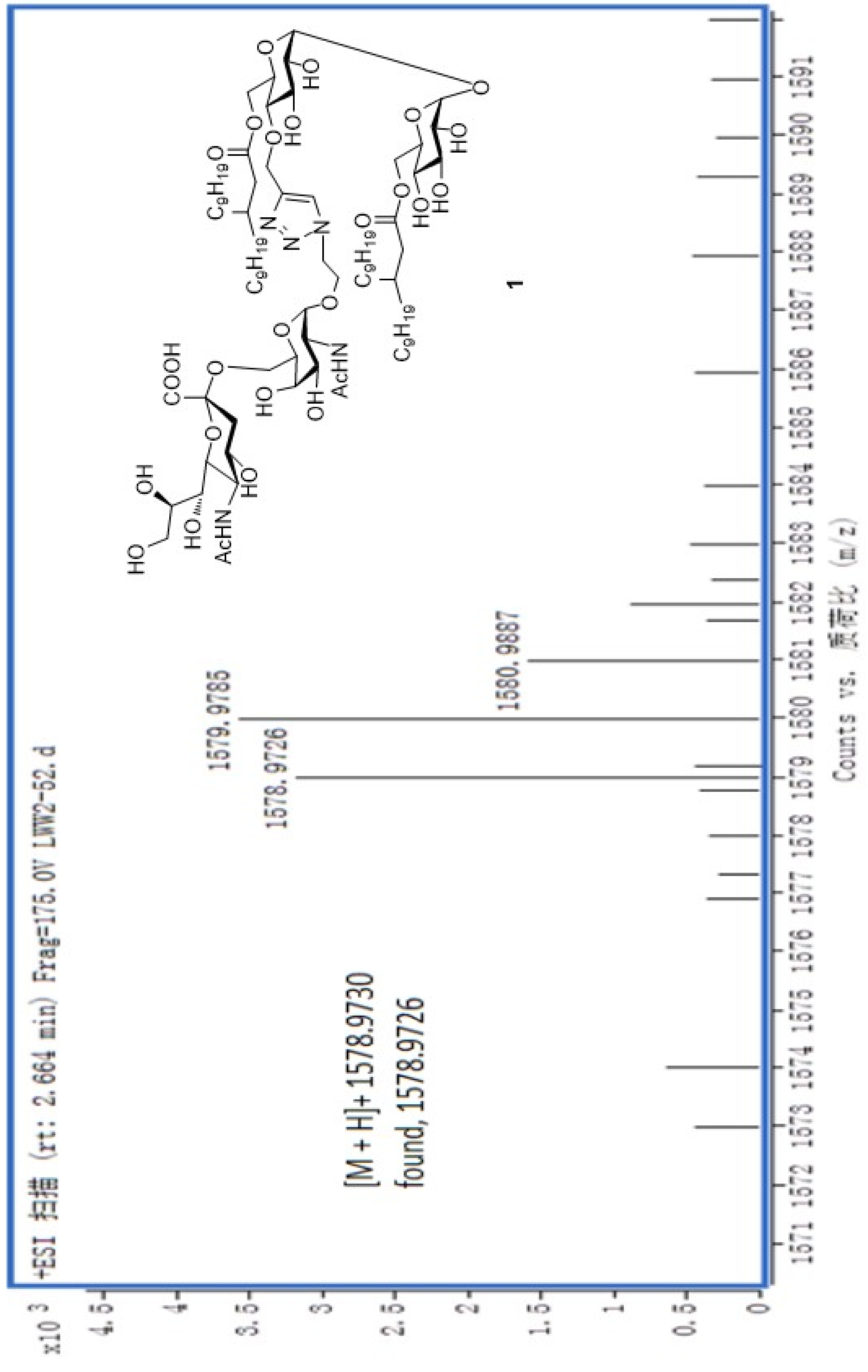

HR-ESI-MS spectrum of conjugate **1**

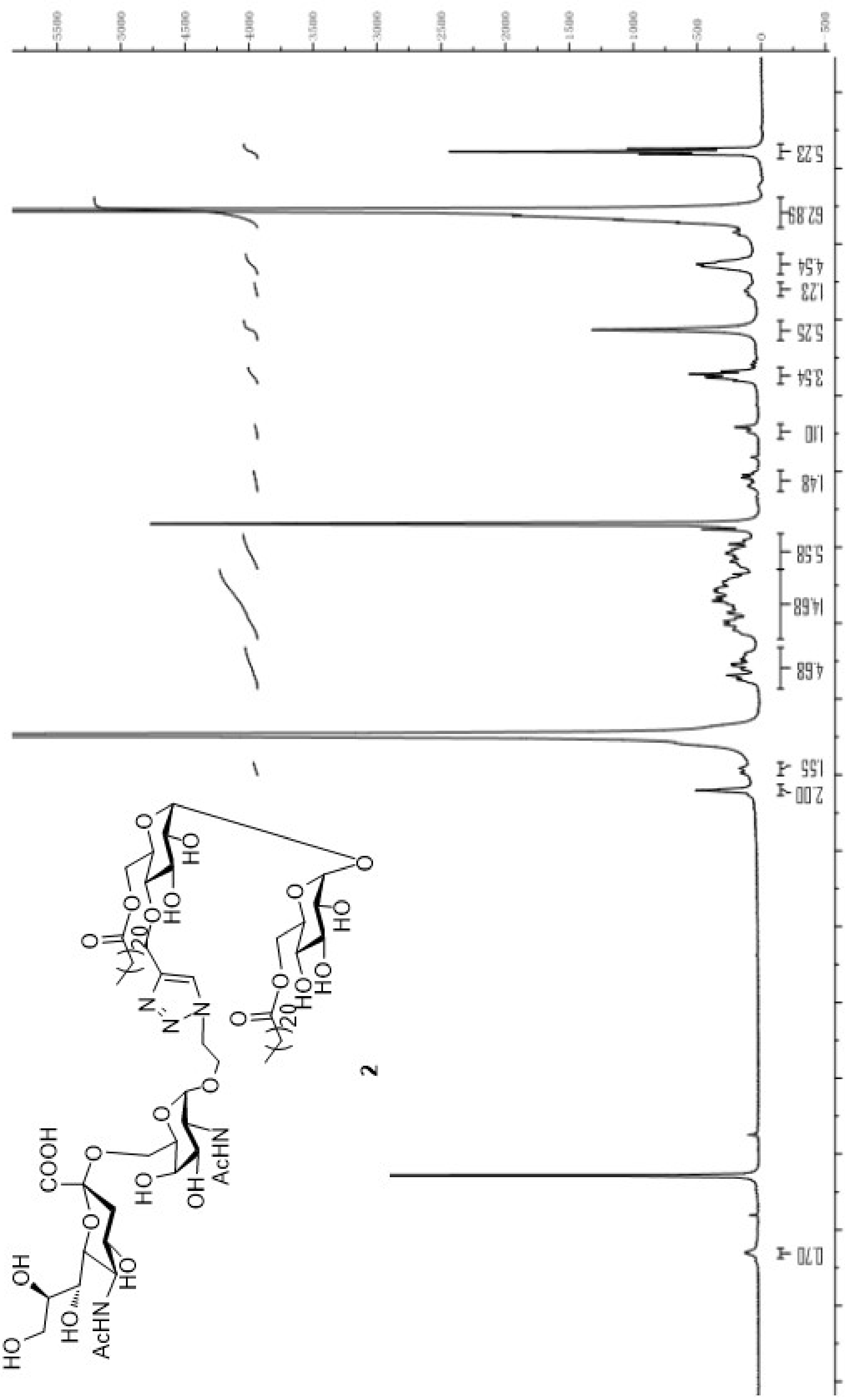

^{1}H NMR Spectrum of compound **2** (CD_3OD-$CDCl_3$=1：4, 400 MHz)

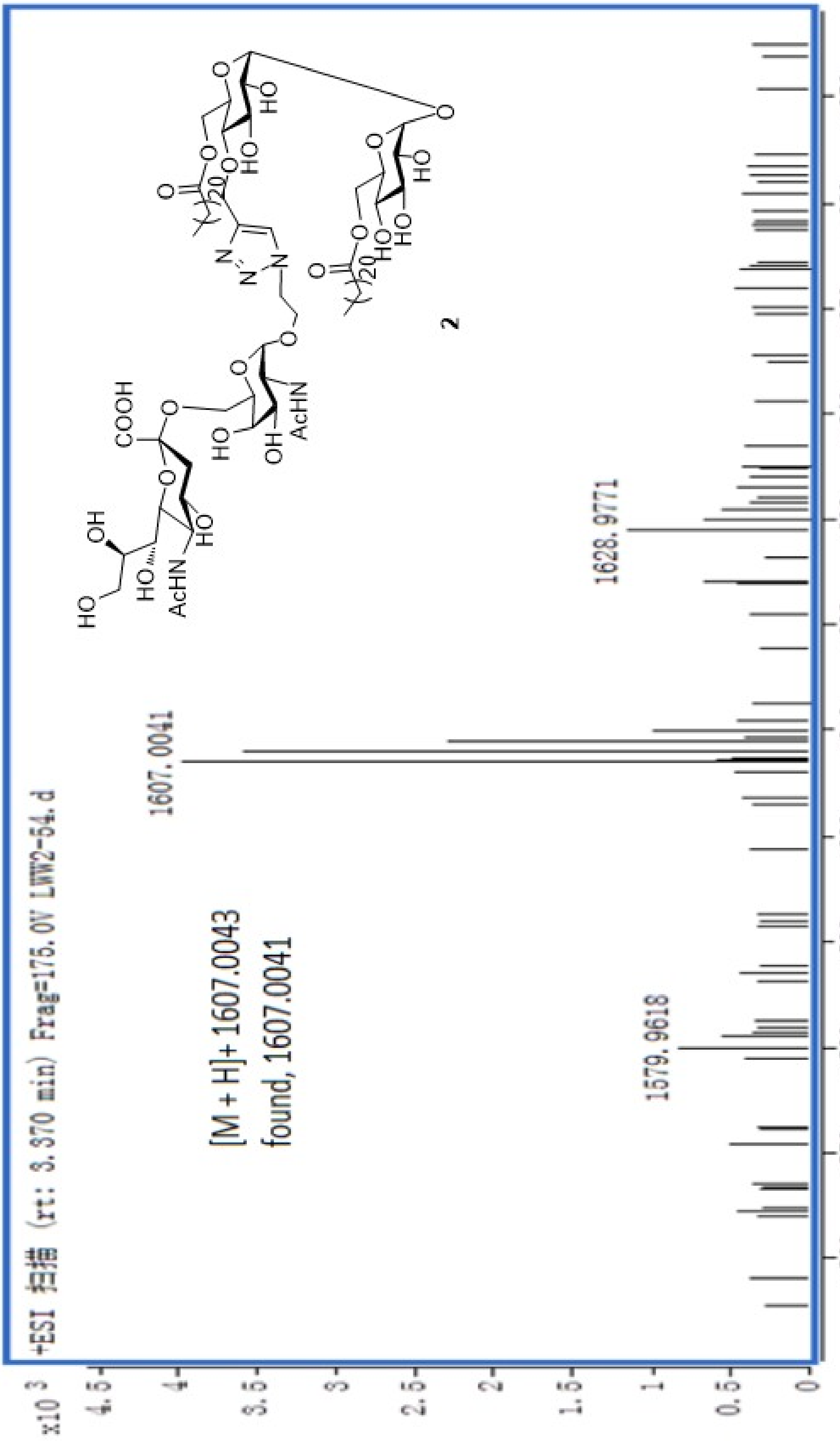

HR-ESI-MS spectrum of conjugate **2**

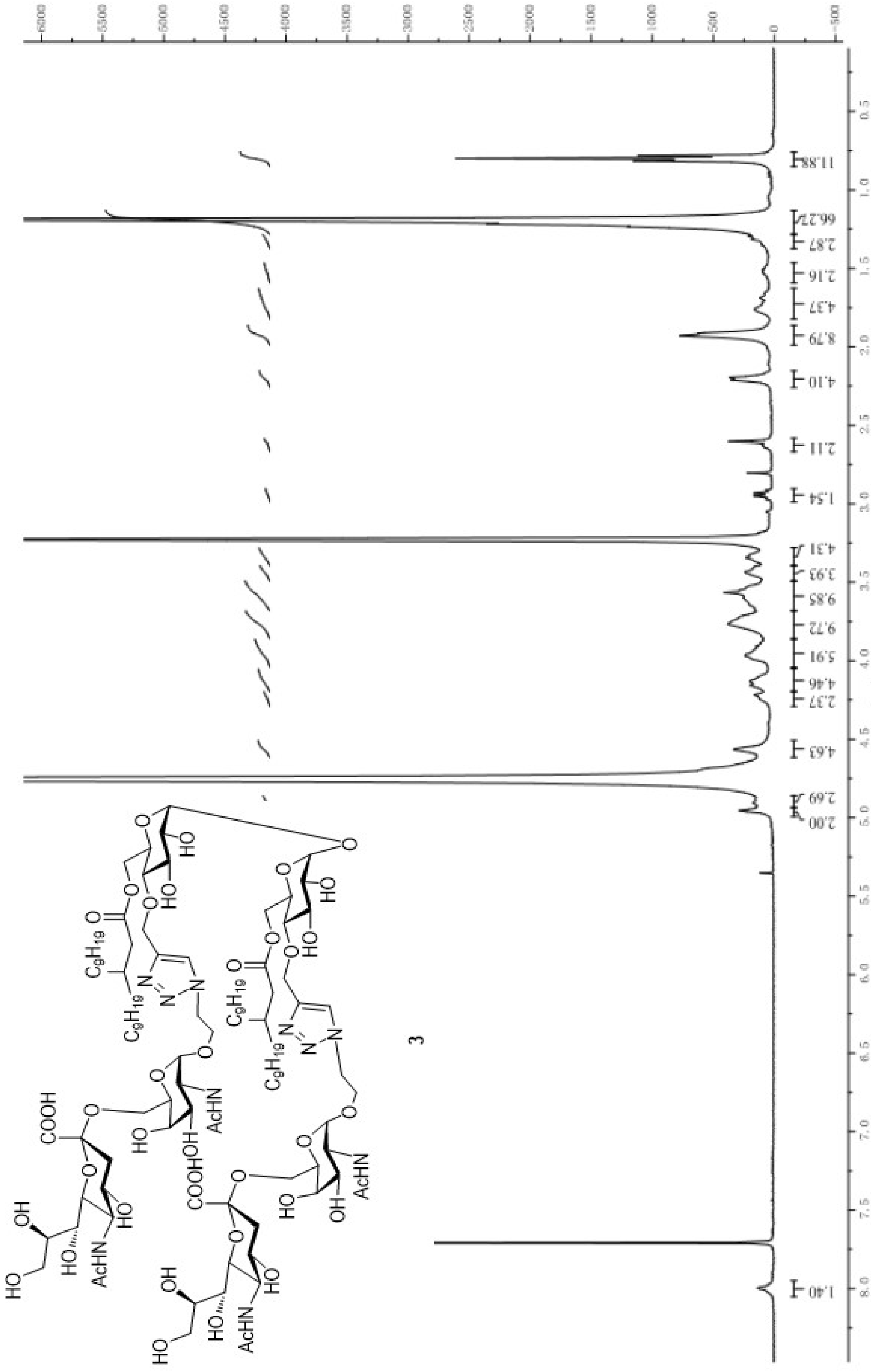

^{1}H NMR Spectrum of compound **3** (CD_3OD-$CDCl_3$=1：4, 400 MHz)

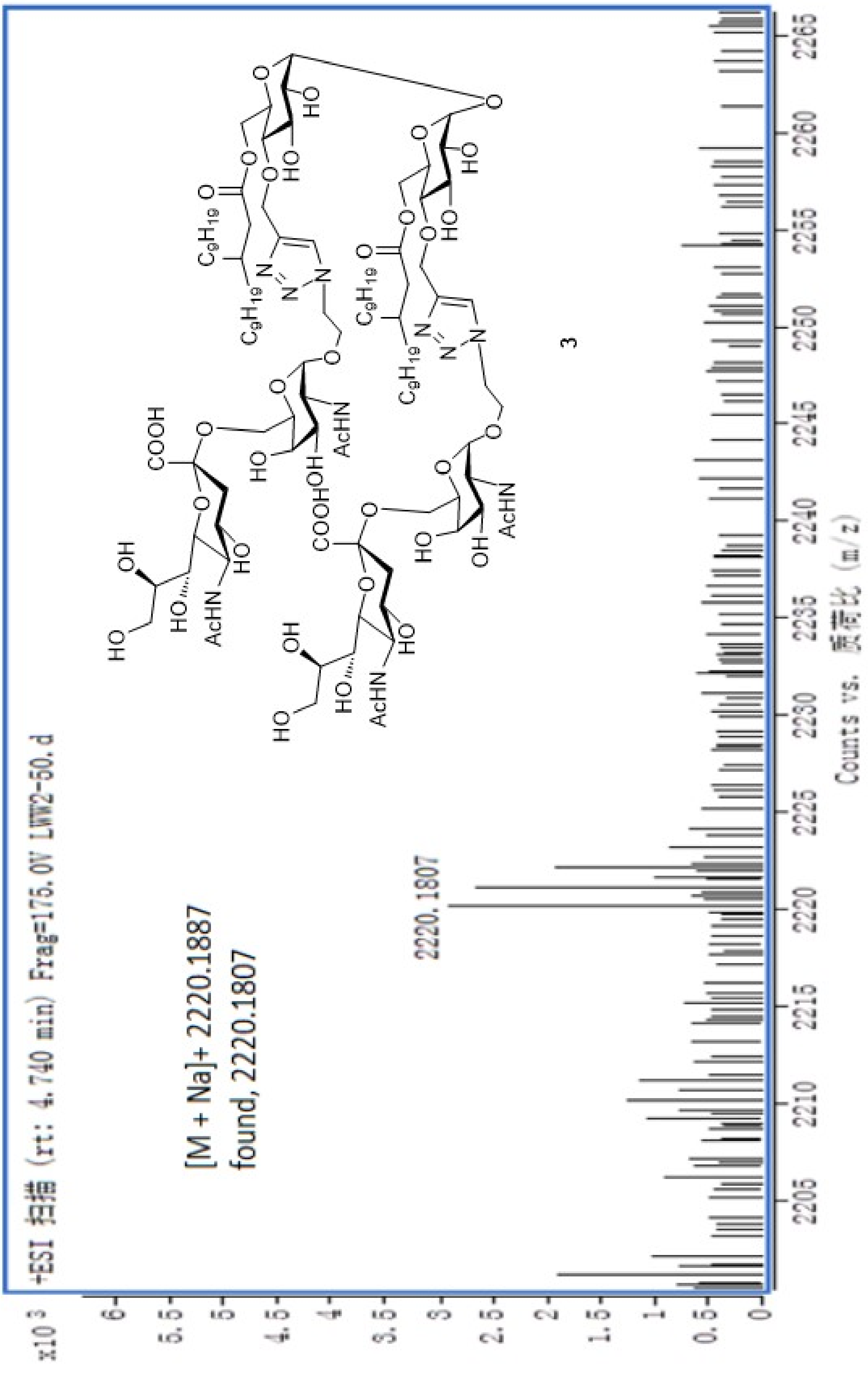

HR-ESI-MS spectrum of conjugate **3**

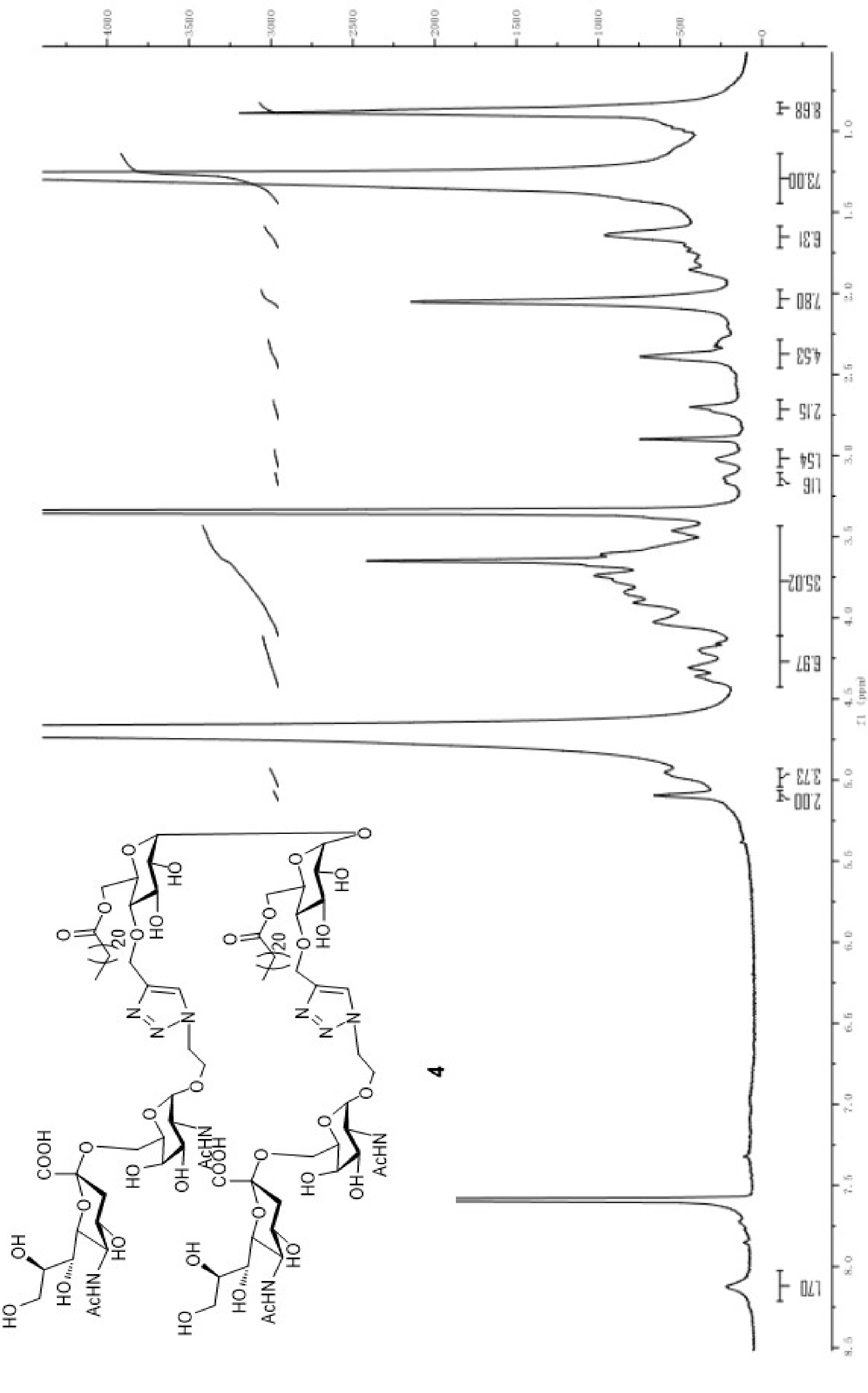

^{1}H NMR Spectrum of compound **4** (CD_3OD-$CDCl_3$=1：4, 400 MHz)

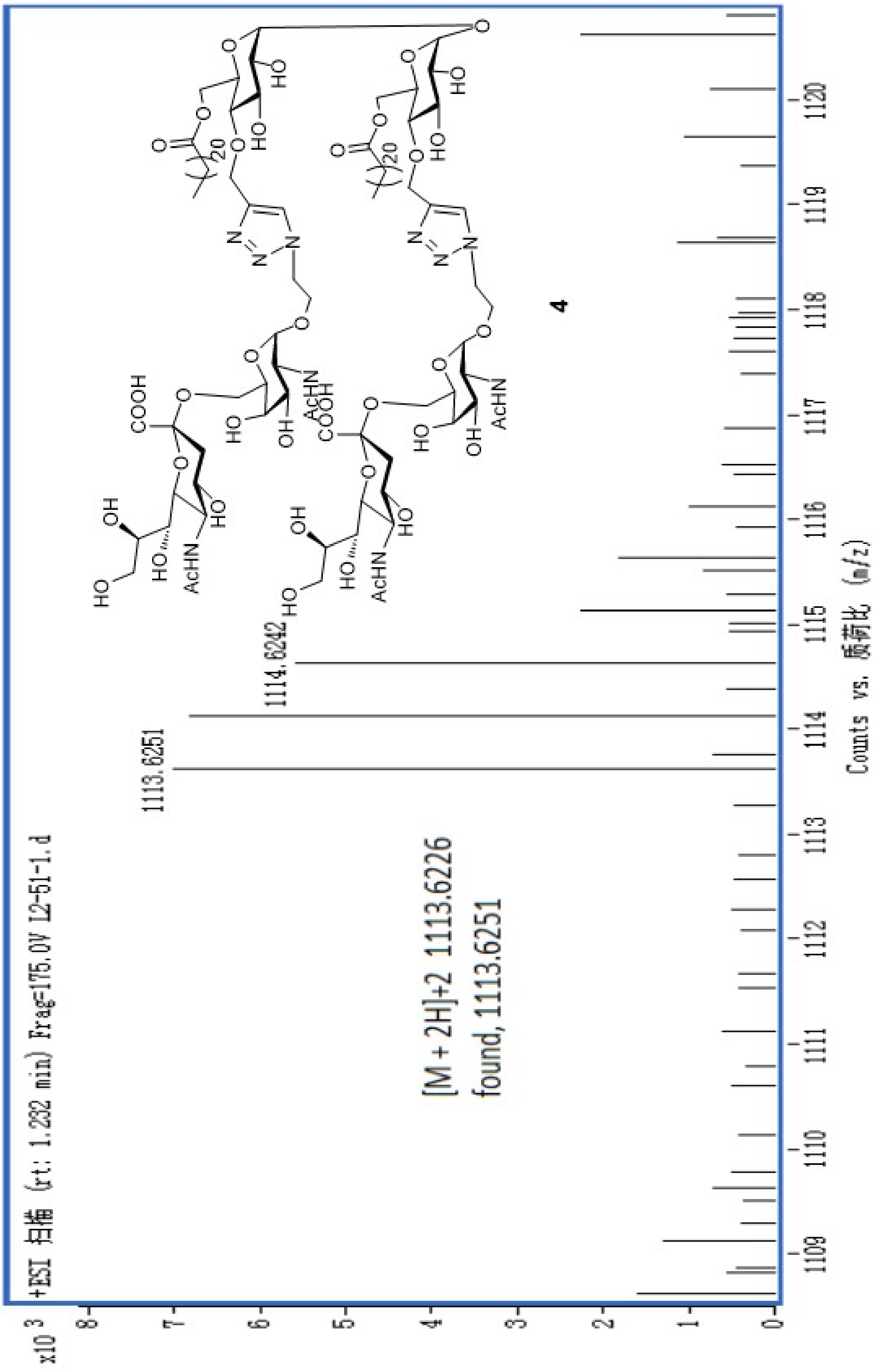

HR-ESI-MS spectrum of conjugate **4**

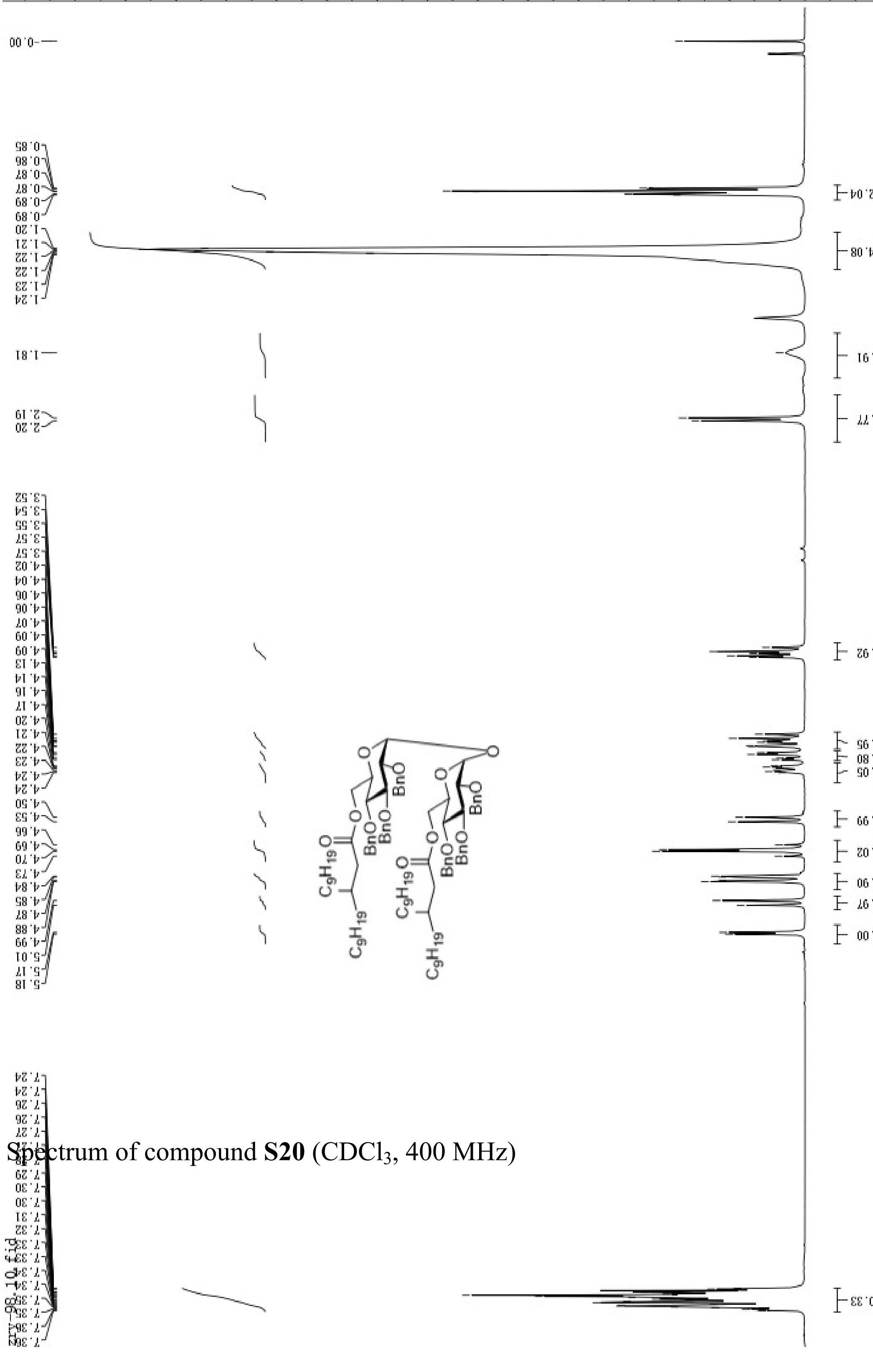

^{1}H NMR Spectrum of compound **S20** ($CDCl_3$, 400 MHz)

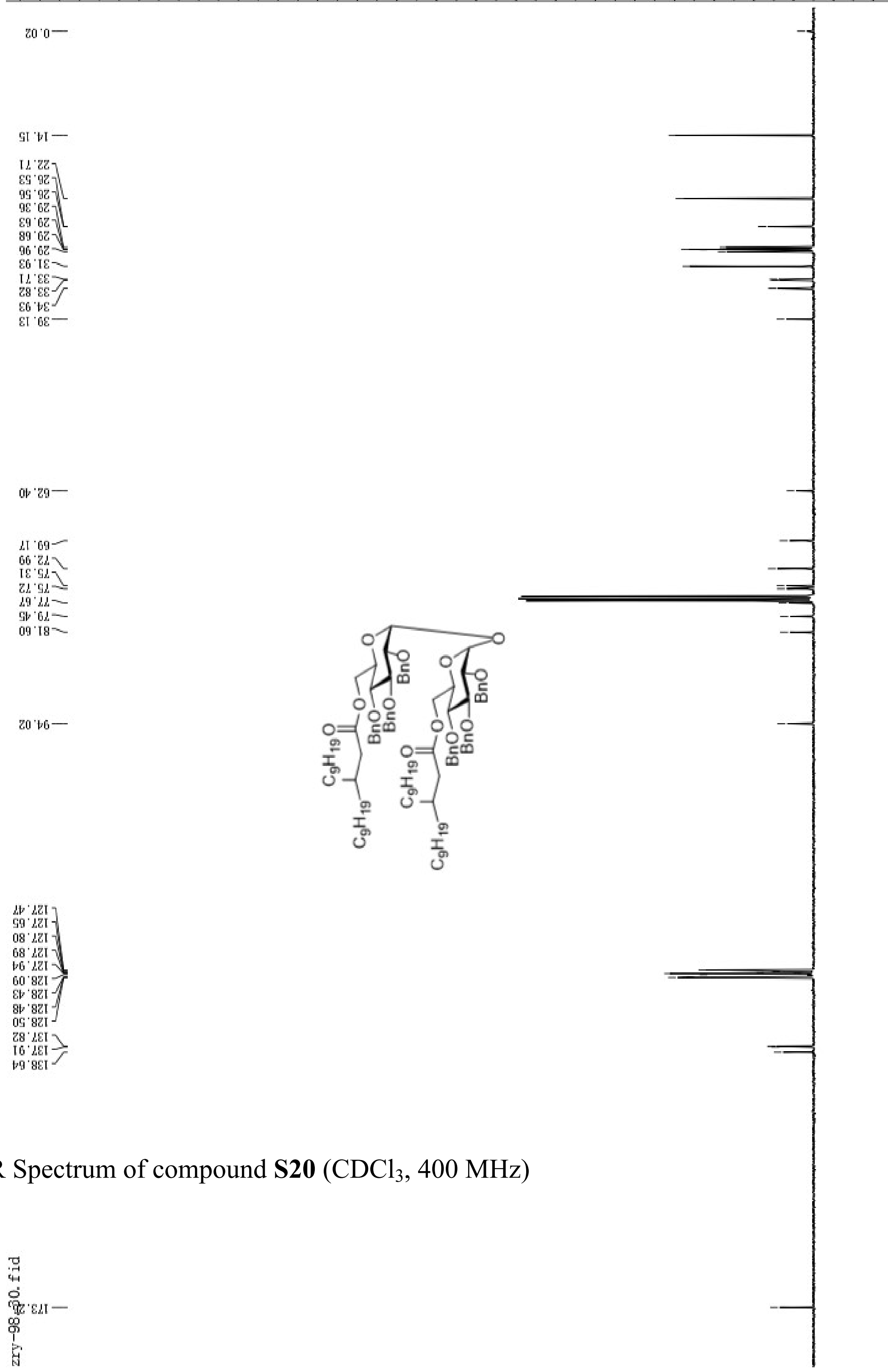

^{13}C NMR Spectrum of compound **S20** ($CDCl_3$, 400 MHz)

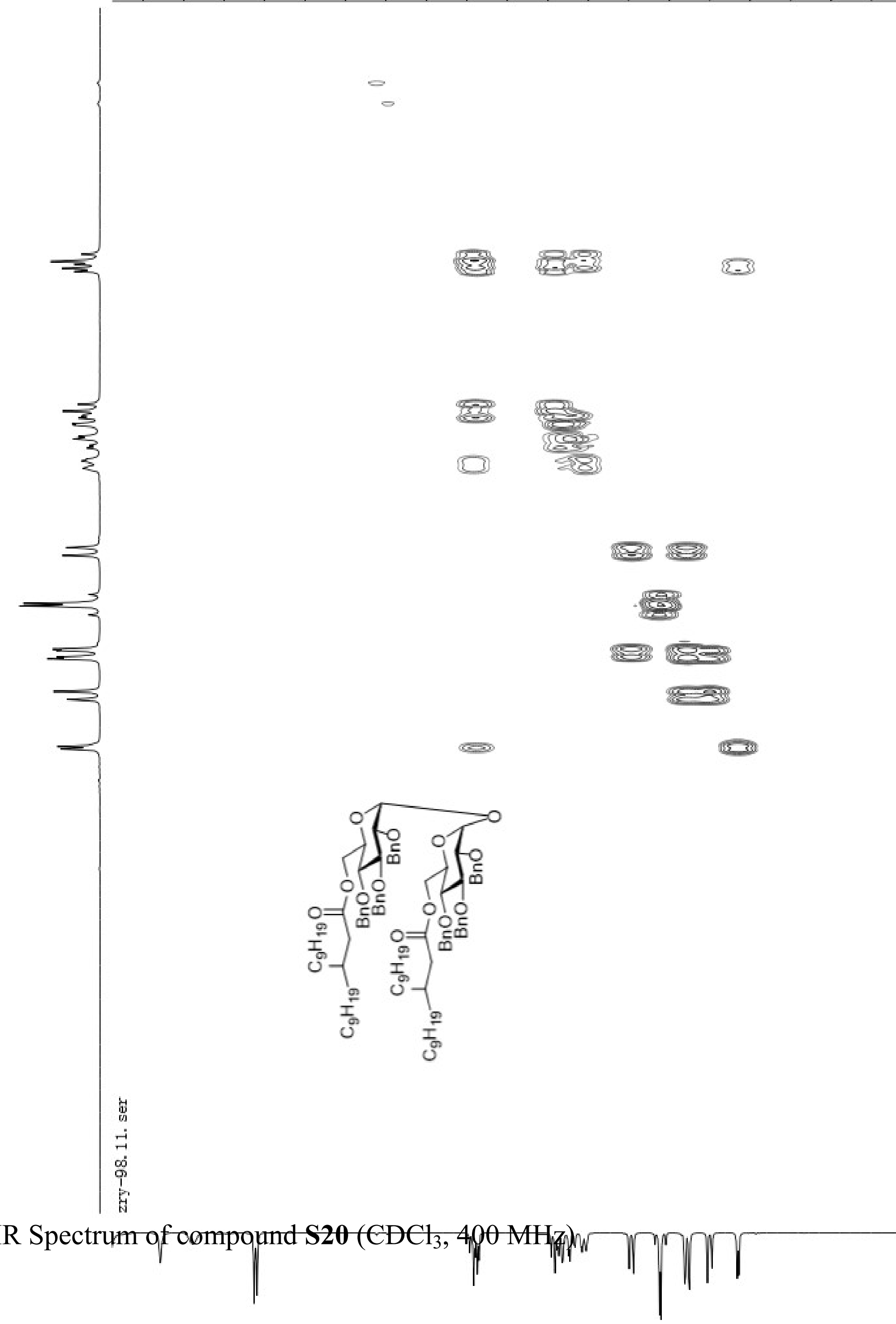

^{1}H-^{1}H COSY NMR Spectrum of compound **S20** ($CDCl_3$, 400 MHz)

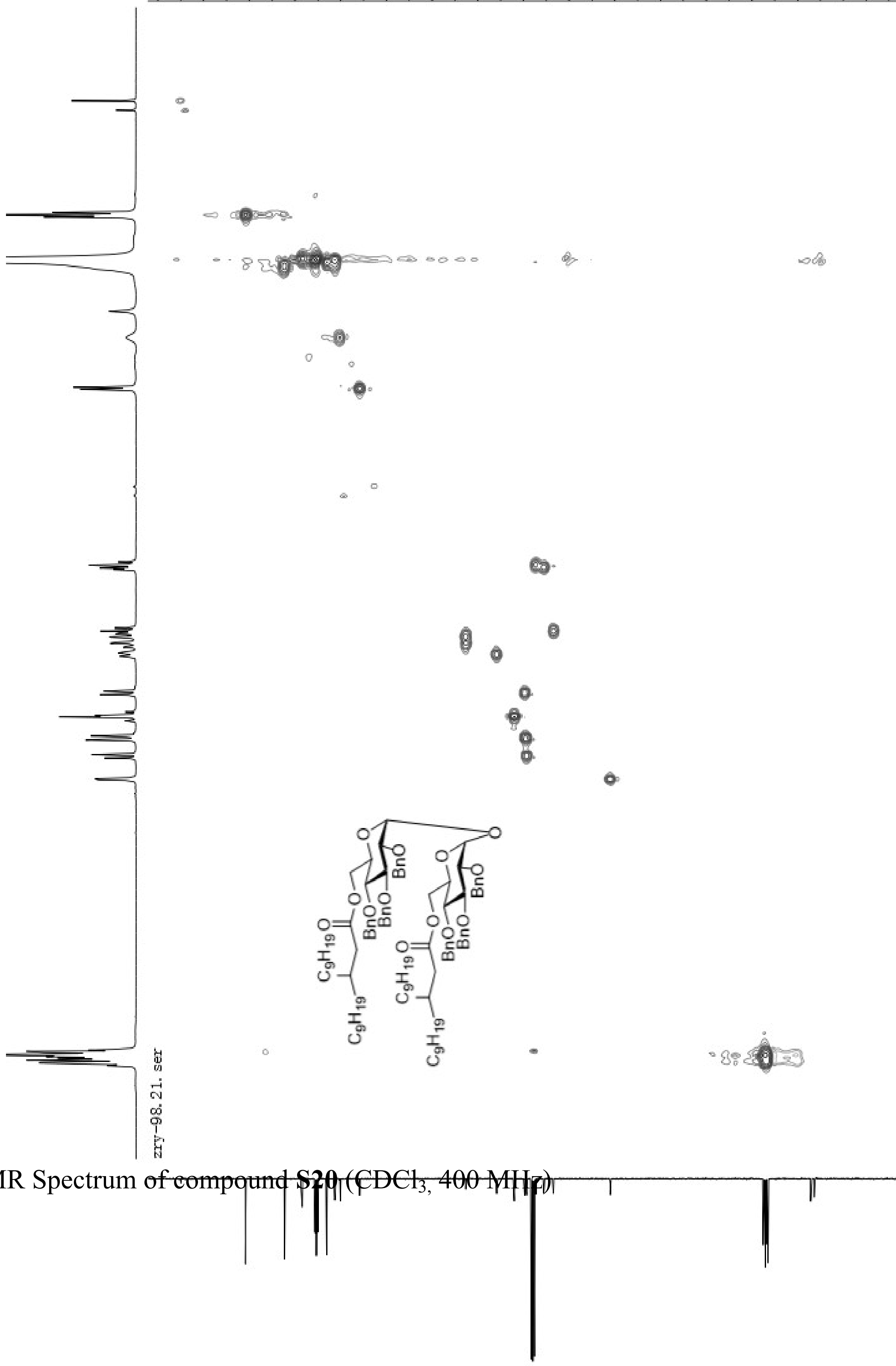

HSQC NMR Spectrum of compound **S20** ($CDCl_3$, 400 MHz)

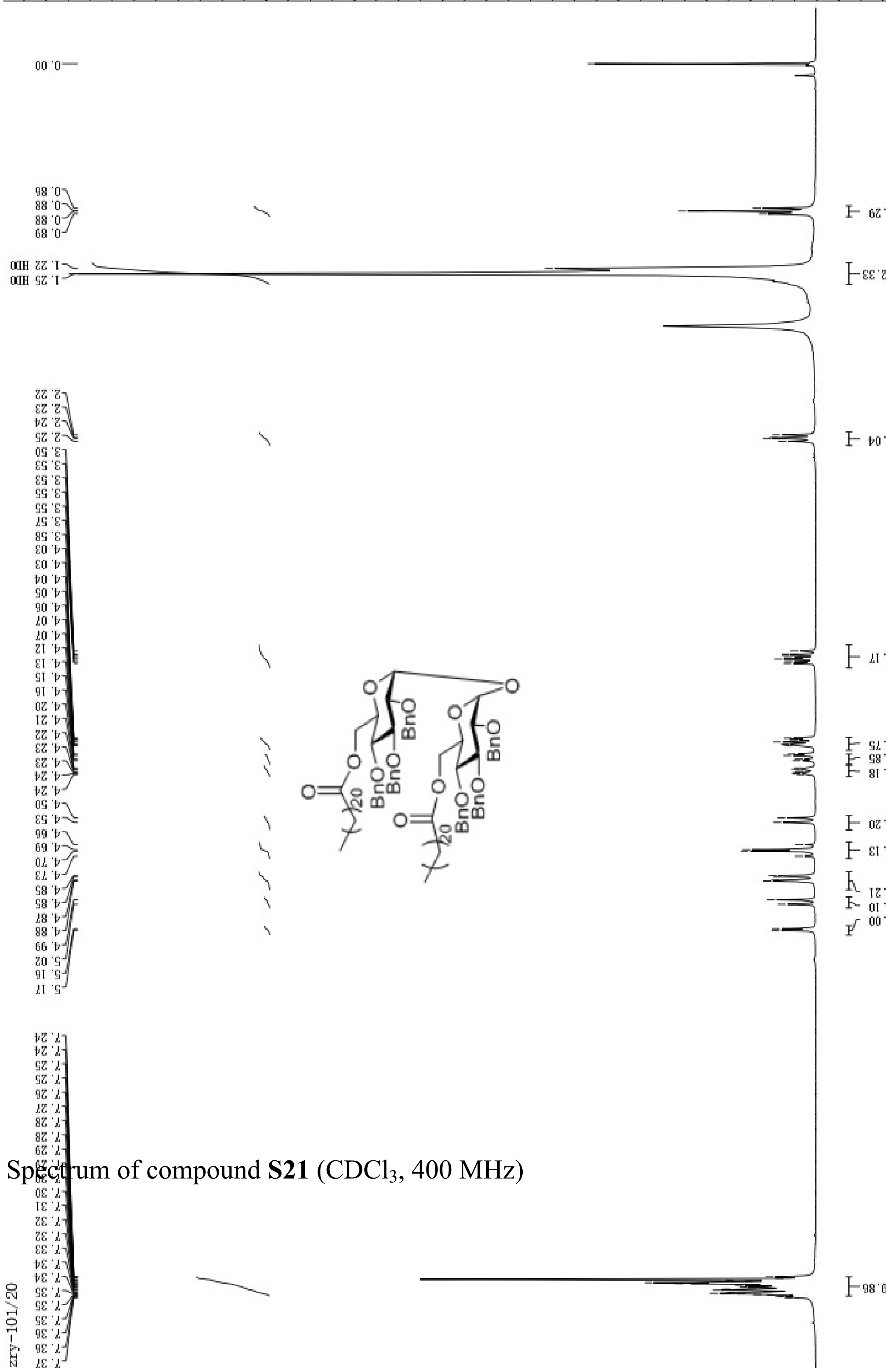

^{1}H NMR Spectrum of compound **S21** ($CDCl_3$, 400 MHz)

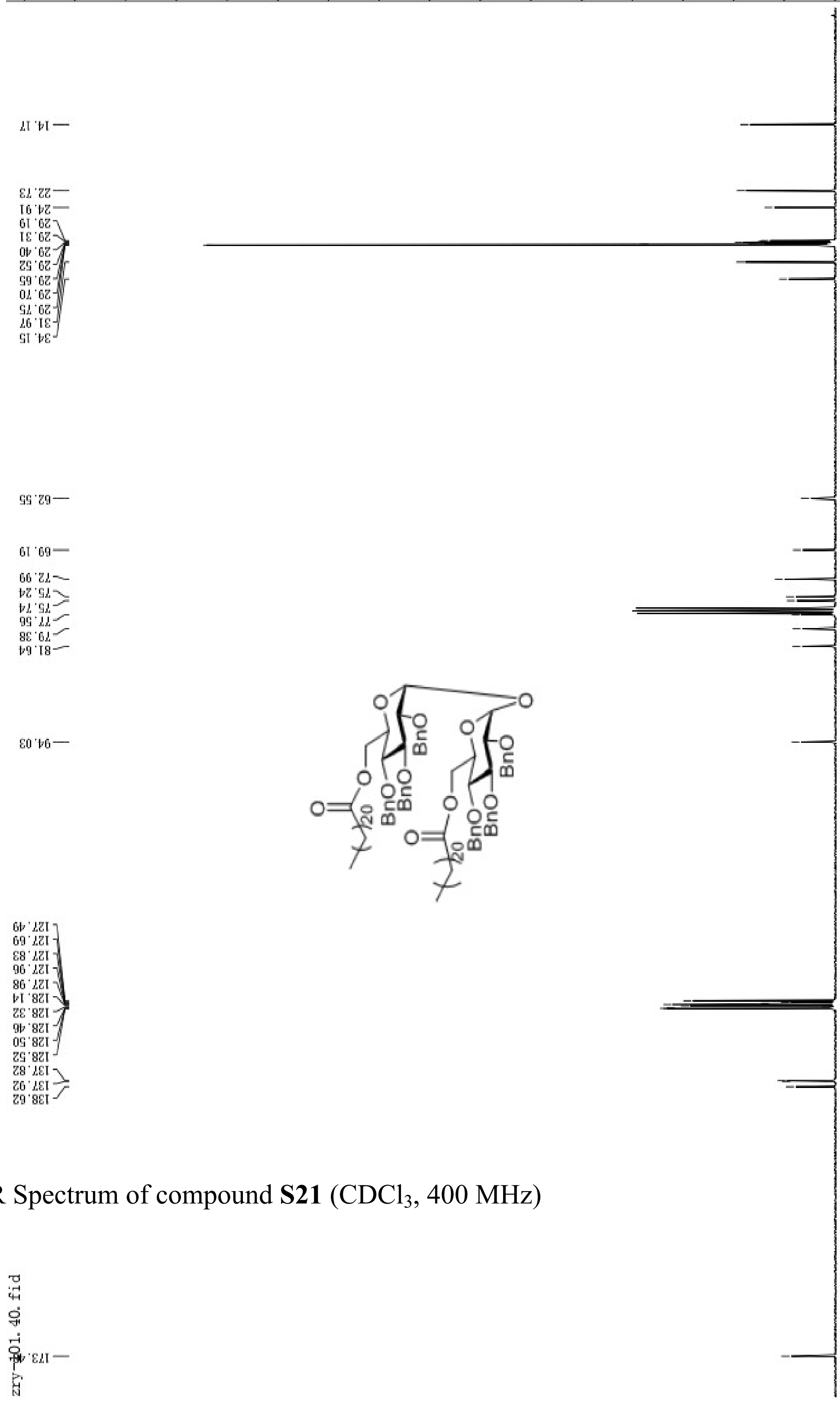

^{13}C NMR Spectrum of compound **S21** ($CDCl_3$, 400 MHz)

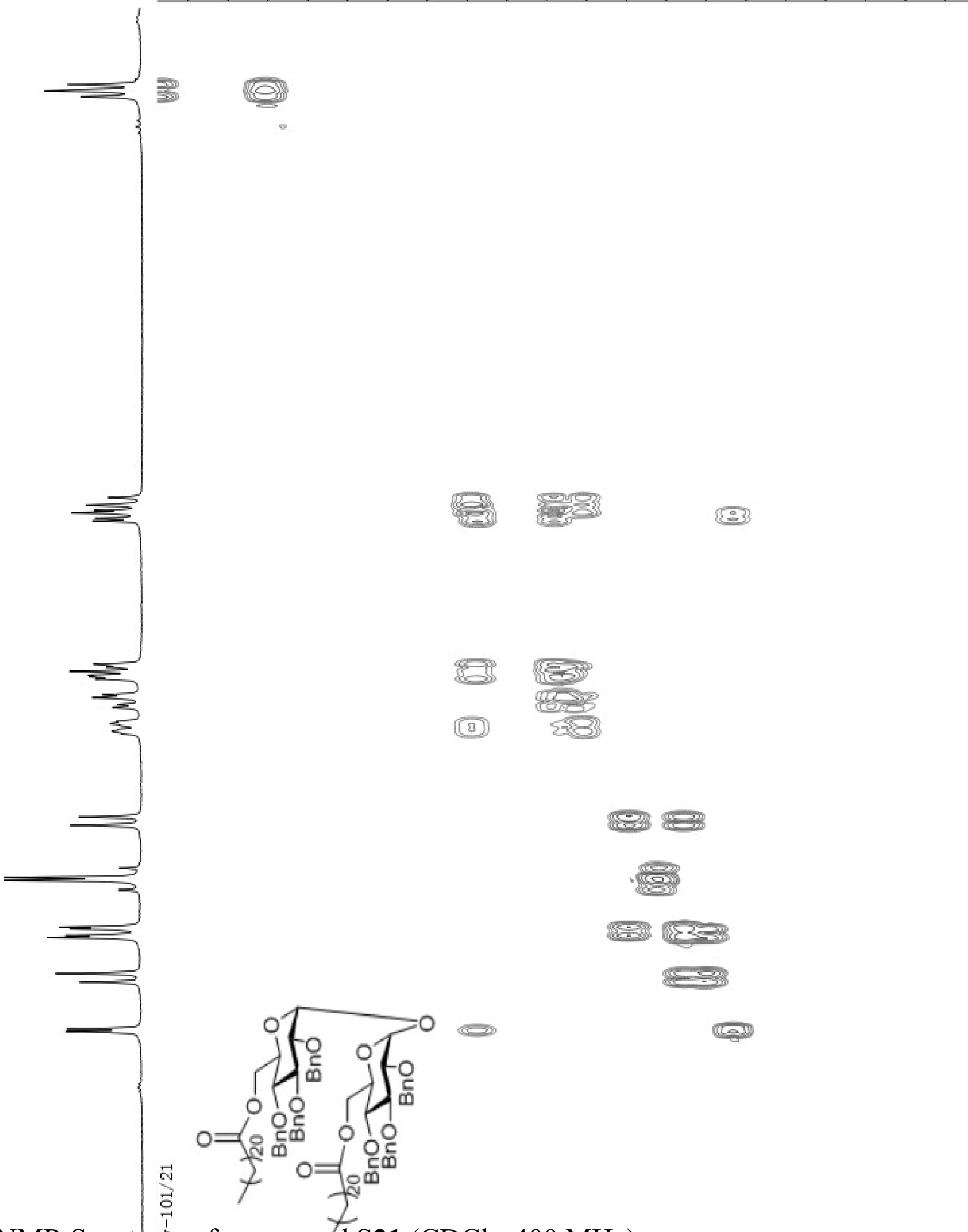

^{1}H-^{1}H COSY NMR Spectrum of compound **S21** ($CDCl_3$, 400 MHz)

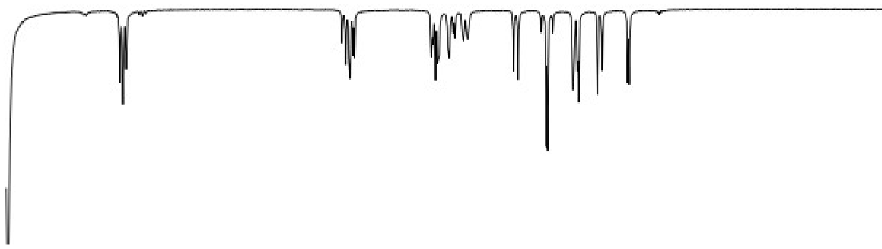

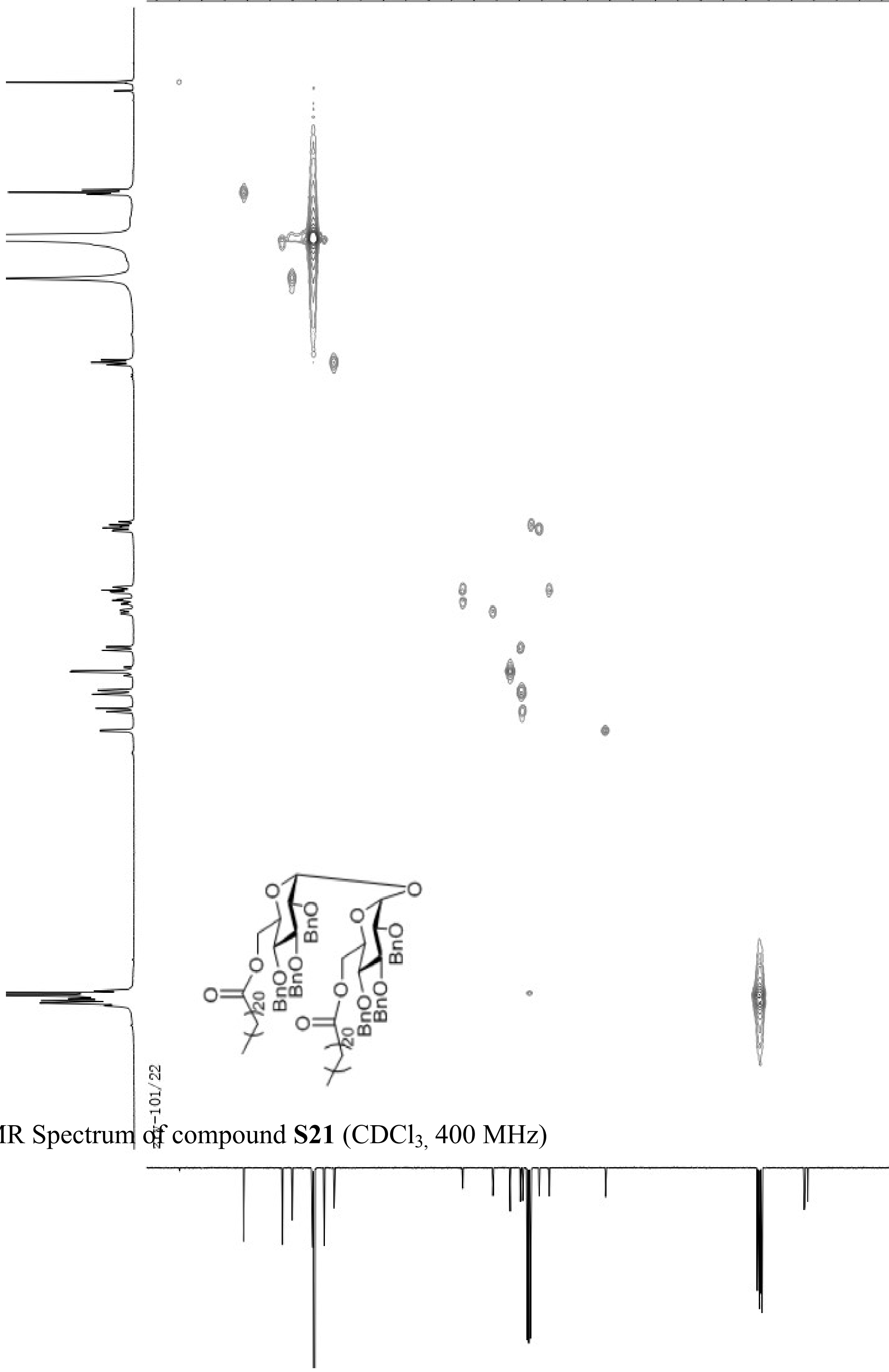

HSQC NMR Spectrum of compound **S21** ($CDCl_3$, 400 MHz)

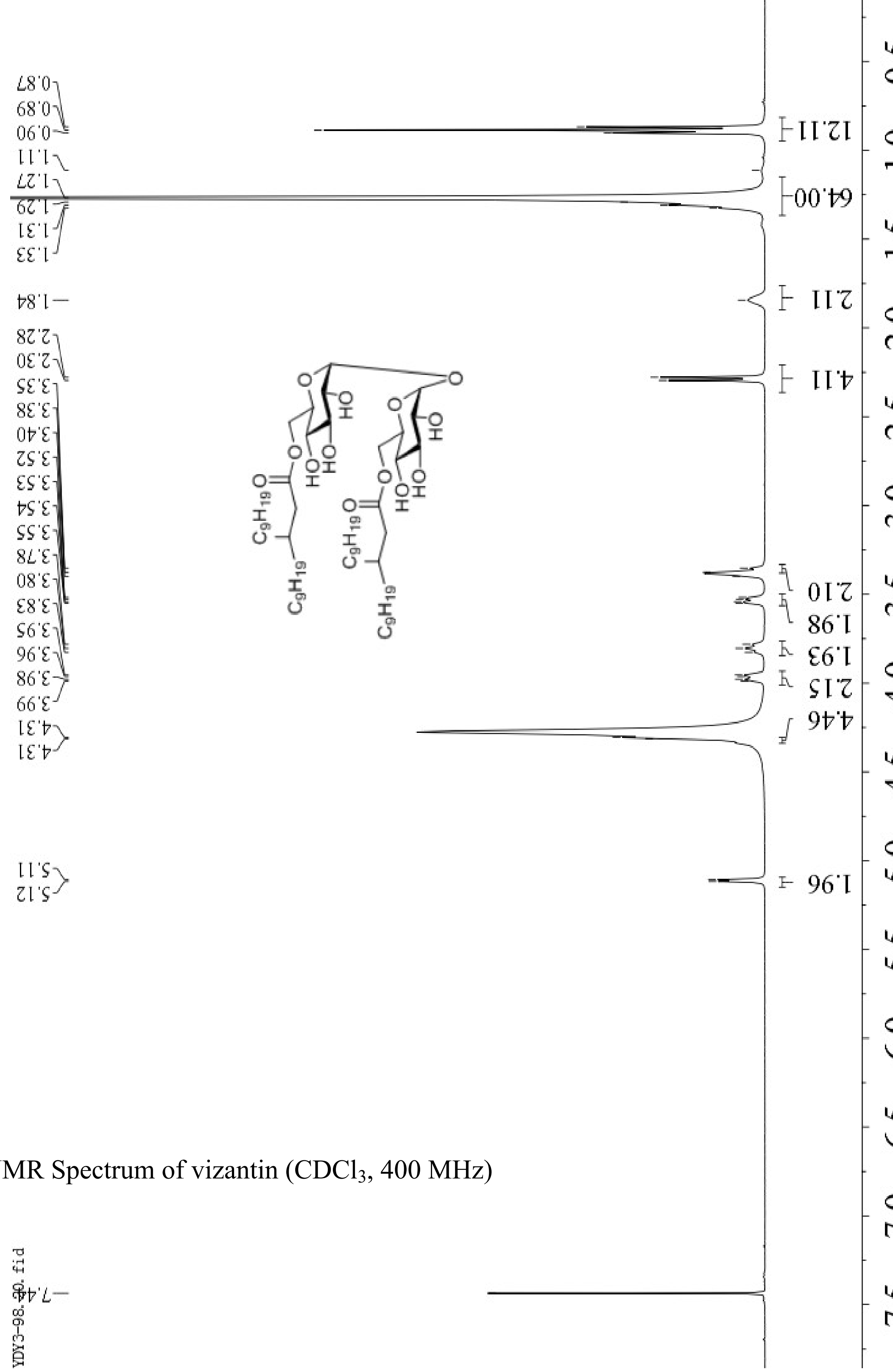

^{1}H NMR Spectrum of vizantin ($CDCl_3$, 400 MHz)

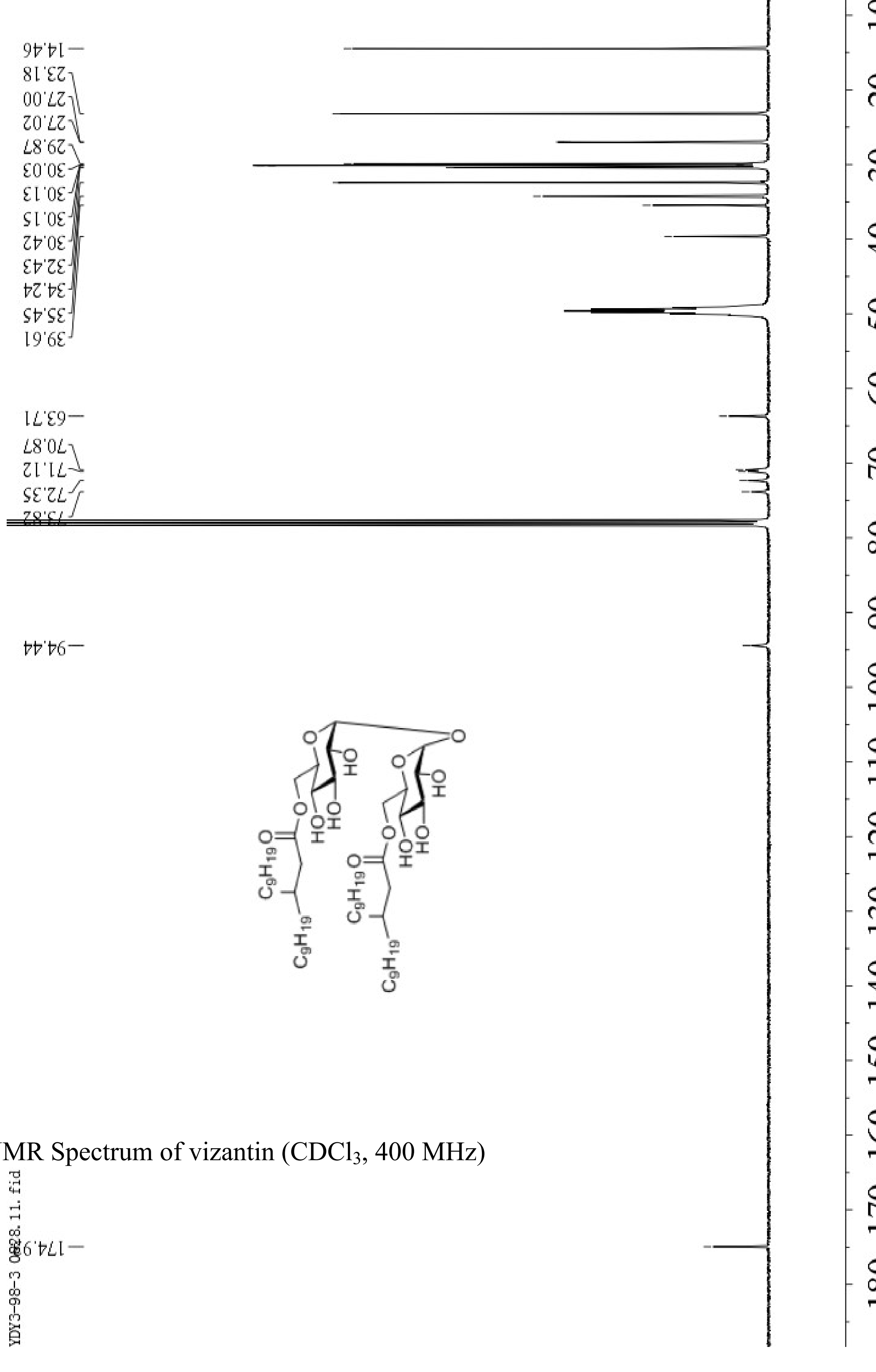

^{13}C NMR Spectrum of vizantin ($CDCl_3$, 400 MHz)

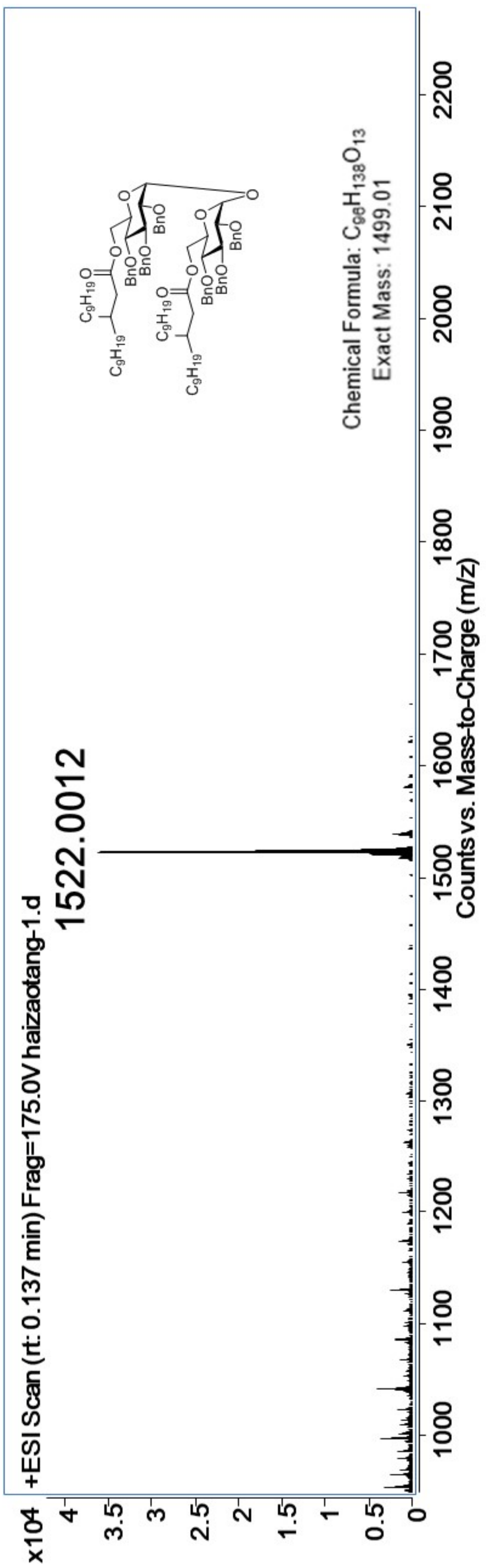

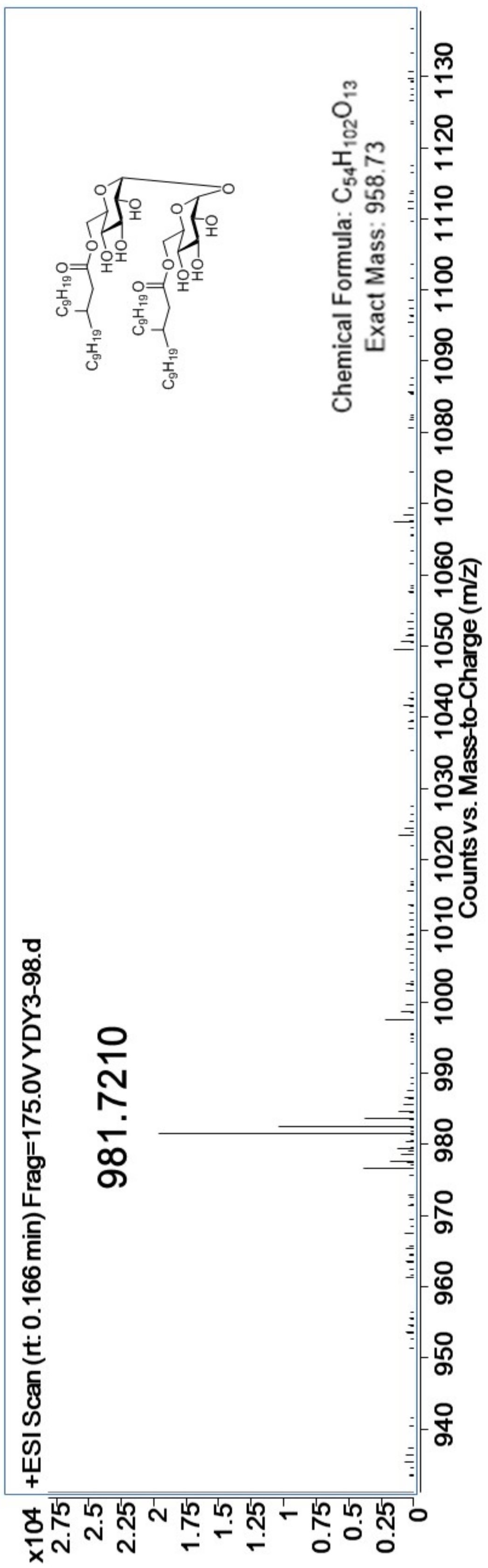

HRMS spectrum of **S20** and vizantin.

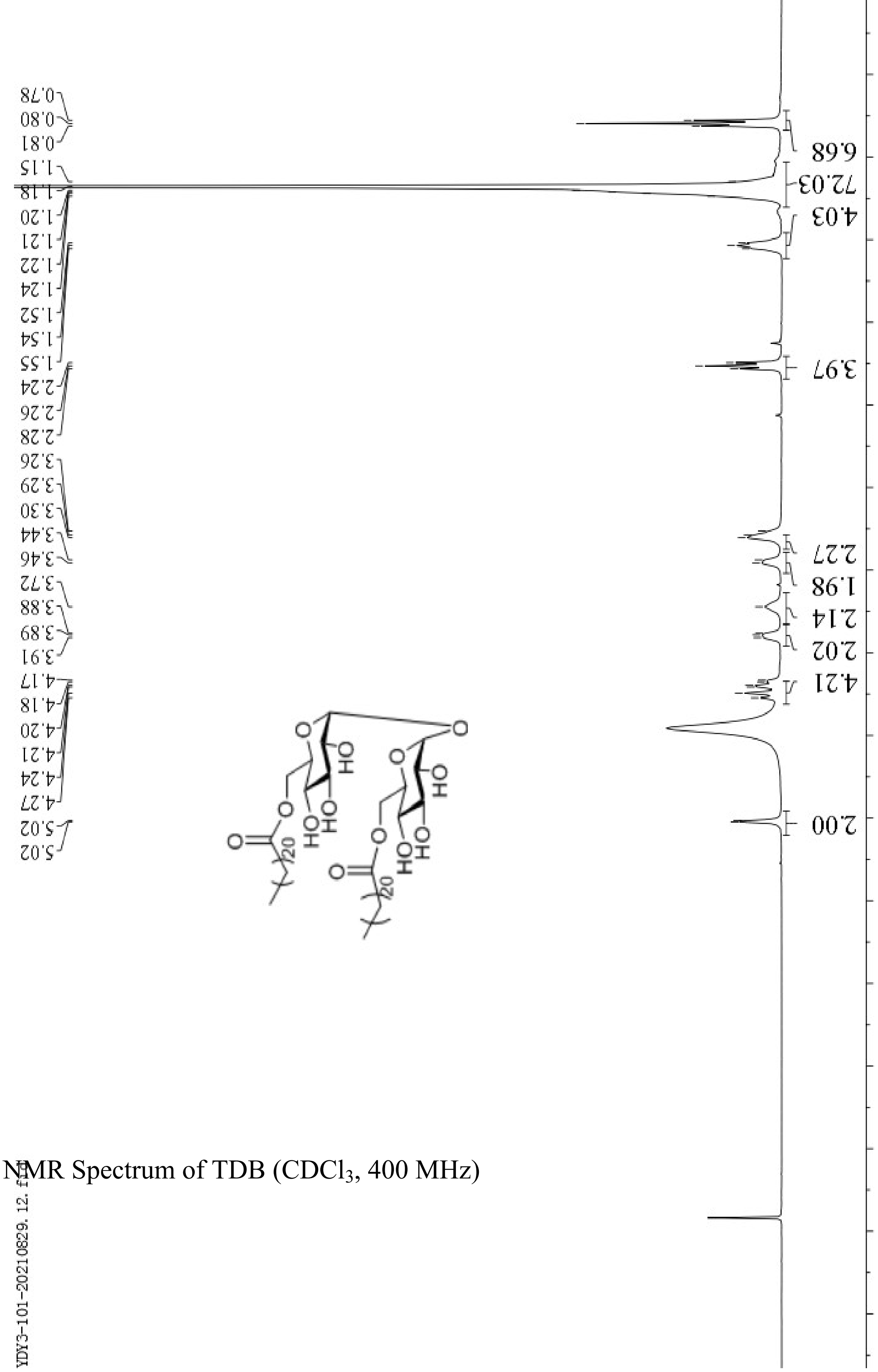

^{1}H NMR Spectrum of TDB ($CDCl_3$, 400 MHz)

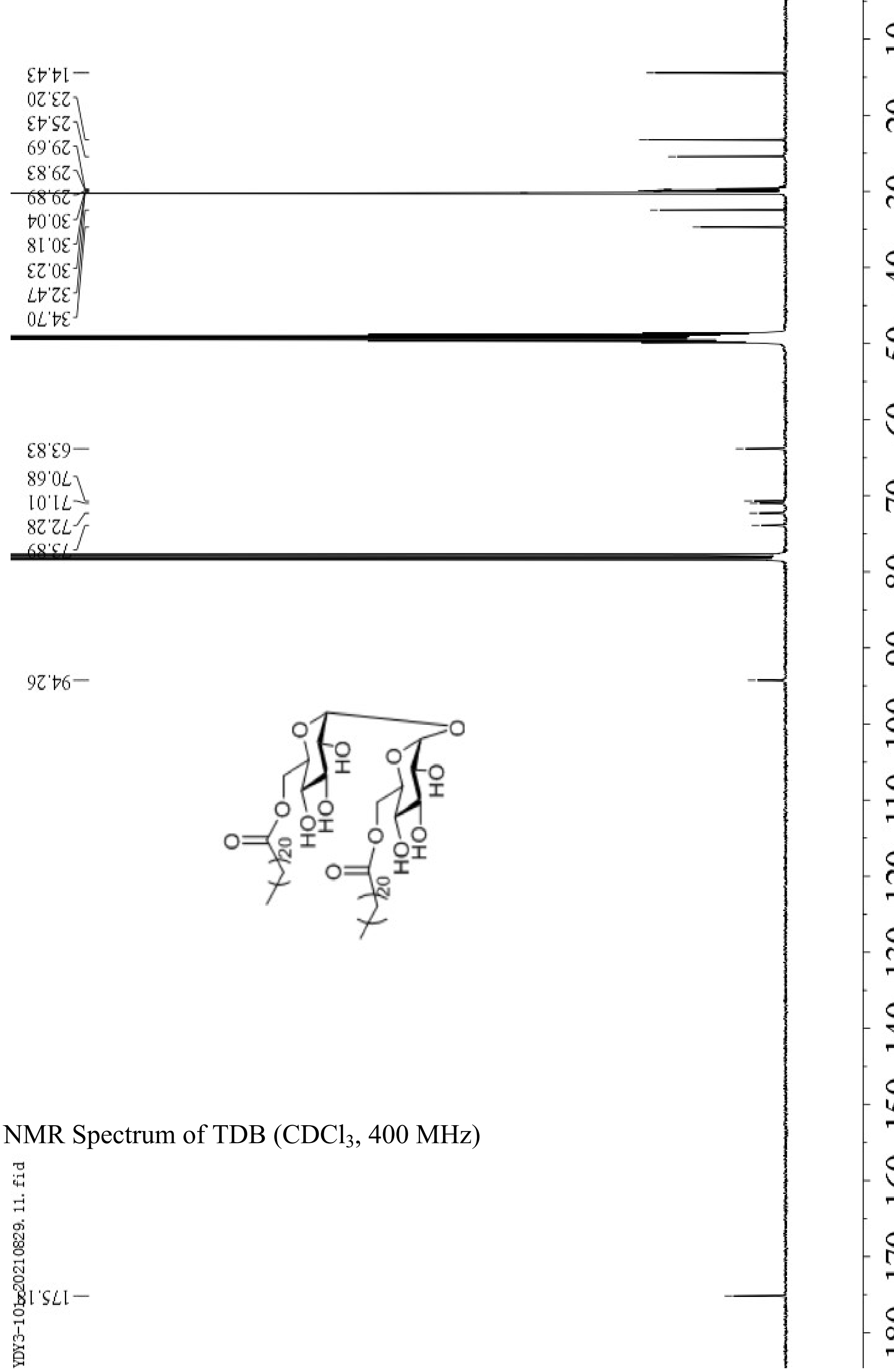

^{13}C NMR Spectrum of TDB ($CDCl_3$, 400 MHz)

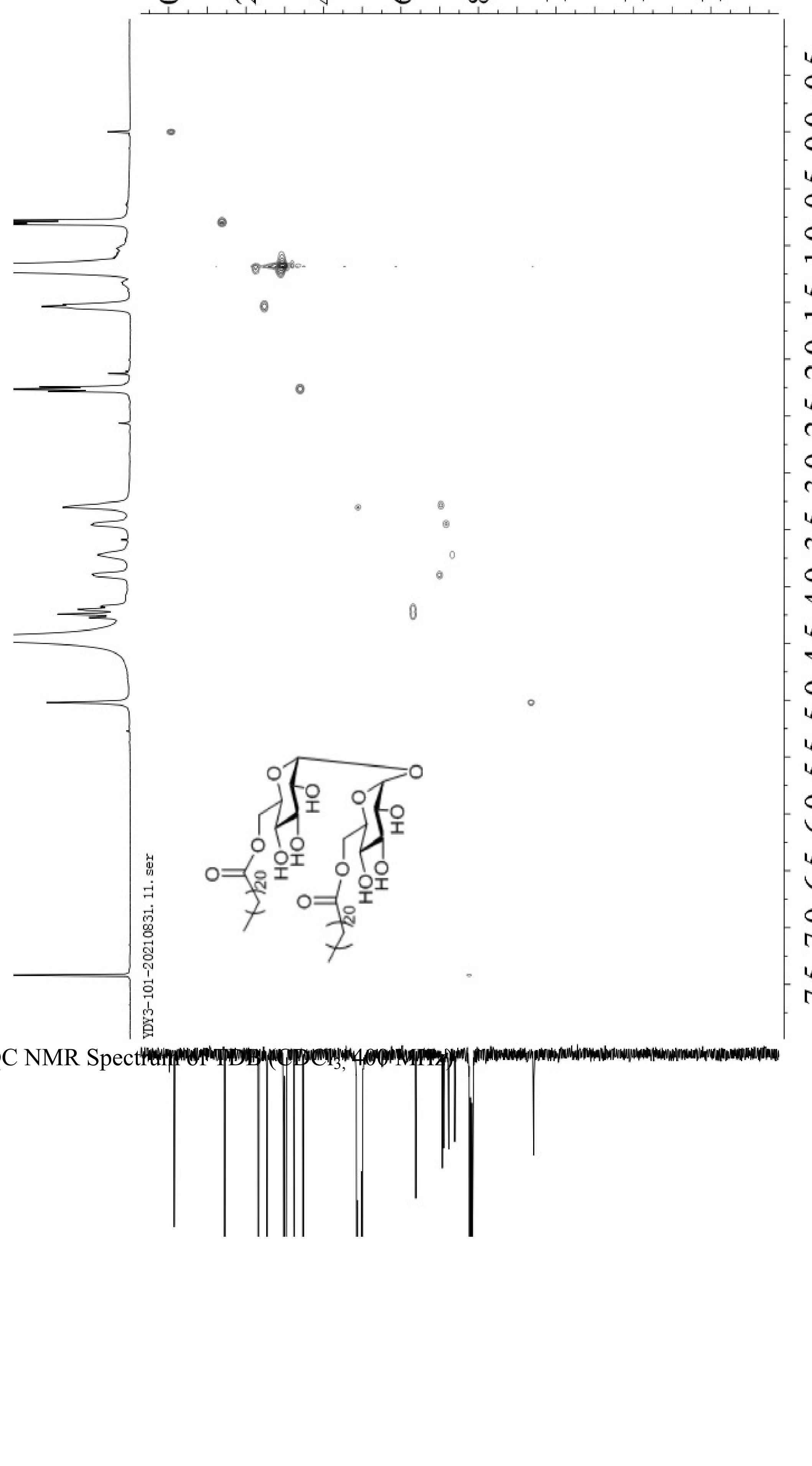

HSQC NMR Spectrum of TDE ($CDCl_3$, 400 MHz)

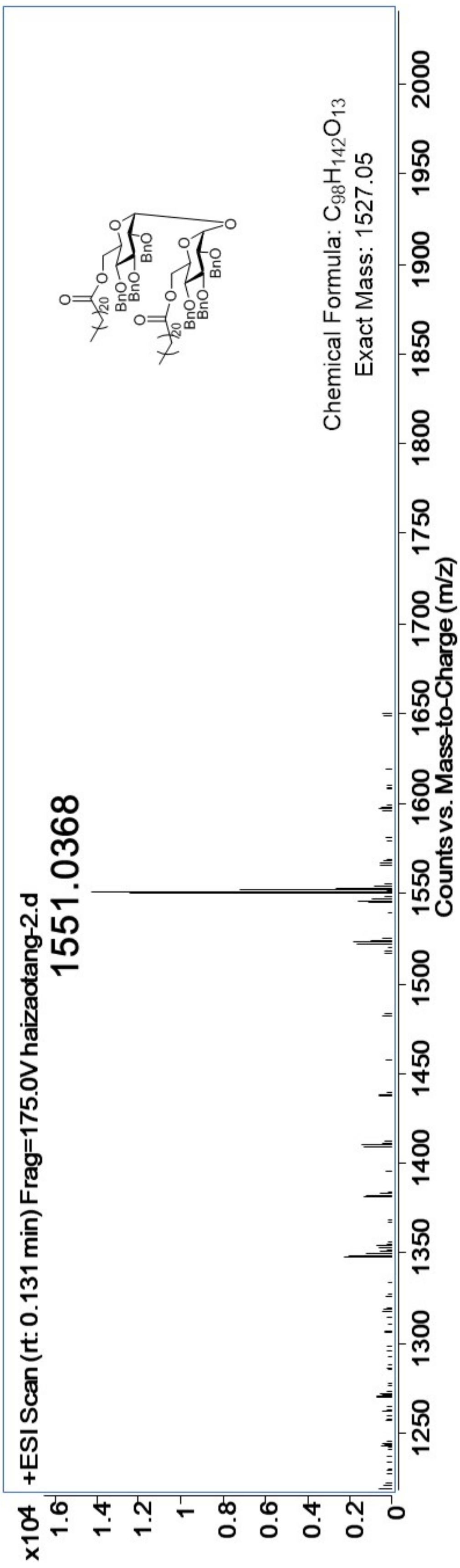

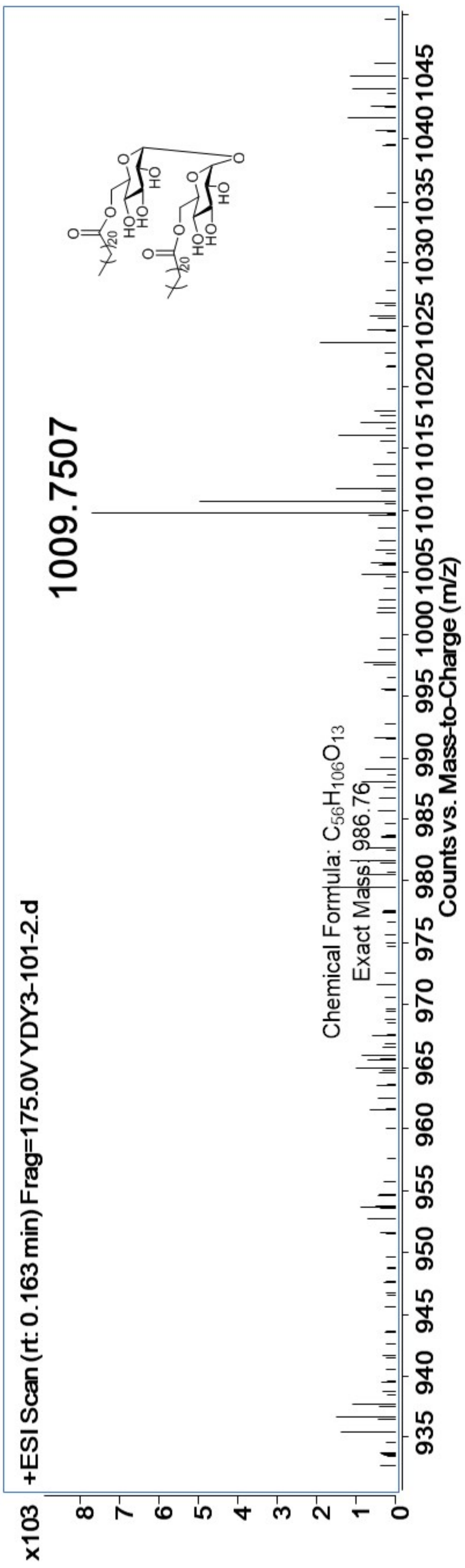

HRMS spectrum of **S21** and TDB.
